# Biodiversity in marine invertebrate responses to acute warming revealed by a comparative multi‐omics approach

**DOI:** 10.1111/gcb.13357

**Published:** 2016-06-17

**Authors:** Melody S. Clark, Ulf Sommer, Jaspreet K. Sihra, Michael A. S. Thorne, Simon A. Morley, Michelle King, Mark R. Viant, Lloyd S. Peck

**Affiliations:** ^1^ British Antarctic Survey Natural Environment Research Council High Cross, Madingley Road Cambridge CB3 0ET UK; ^2^ NERC Biomolecular Analysis Facility – Metabolomics Node (NBAF‐B) School of Biosciences University of Birmingham Edgbaston Birmingham B15 2TT UK

**Keywords:** ^1^H NMR, anaerobic end products, biodiversity, ecosystem, heat shock response, LC‐MS, macrophysiology, marine invertebrate, metabolomics, transcriptomics

## Abstract

Understanding species' responses to environmental change underpins our abilities to make predictions on future biodiversity under any range of scenarios. In spite of the huge biodiversity in most ecosystems, a model species approach is often taken in environmental studies. To date, we still do not know how many species we need to study to input into models and inform on ecosystem‐level responses to change. In this study, we tested current paradigms on factors setting thermal limits by investigating the acute warming response of six Antarctic marine invertebrates: a crustacean *Paraceradocus miersi*, a brachiopod *Liothyrella uva*, two bivalve molluscs, *Laternula elliptica*,* Aequiyoldia eightsii*, a gastropod mollusc *Marseniopsis mollis* and an echinoderm *Cucumaria georgiana*. Each species was warmed at the rate of 1 °C h^−1^ and taken to the same physiological end point (just prior to heat coma). Their molecular responses were evaluated using complementary metabolomics and transcriptomics approaches with the aim of discovering the underlying mechanisms of their resilience or sensitivity to warming. The responses were species‐specific; only two showed accumulation of anaerobic end products and three exhibited the classical heat shock response with expression of HSP70 transcripts. These diverse cellular measures did not directly correlate with resilience to heat stress and suggested that each species may have a different critical point of failure. Thus, one unifying molecular mechanism underpinning response to warming could not be assigned, and no overarching paradigm was supported. This biodiversity in response makes future ecosystems predictions extremely challenging, as we clearly need to develop a macrophysiology‐type approach to cellular evaluations of the environmental stress response, studying a range of well‐rationalized members from different community levels and of different phylogenetic origins rather than extrapolating from one or two arbitrary model species.

## Introduction

Accurately predicting climate change effects at the ecosystem level is complex and fraught with difficulty. However, such knowledge is critical to understanding future biodiversity and the impact on ecosystem services. As it is impossible to acquire a detailed knowledge of the responses of all species within an ecosystem to a given environmental perturbation, a tractable approach is to evaluate a series of candidate species to understand the underlying mechanisms (Pörtner, 2010; Peck, 2011). Identifying the factors that lie behind the sensitivity or resilience of a range of species to changing conditions enables the extrapolation of those results to other less well‐characterized species (i.e. applying a macrophysiological approach) and improves our abilities to predict the ecosystem‐level consequences of climate change (Chown & Gaston, 2016). However, the challenge with this approach is identifying the number and choice of species that must be investigated to predict the behaviour of the ecosystem and developing robust experimental protocols to inform on relative sensitivities (Buckley & Kingsolver, 2012; Chown & Gaston, 2016).

One approach to this problem is to use experimental manipulation of thermal tolerances using ramping assays. These are proving effective at predicting future thermal tolerances, even though these are very short term relative to the rate of climate change (Peck *et al*., 2009; Terblanche *et al*., 2011). Importantly, they provide an estimate of the relative sensitivities of different species to a particular environmental stress (Peck *et al*., 2009; Buckley & Kingsolver, 2012). These types of experiments are particularly useful for those species where long‐term husbandry is not known, which compromises the ability to successfully perform long‐term acclimations. In previous thermal ramping trials using Antarctic marine invertebrates, more active animals survived to higher temperatures than sessile species (Peck *et al*., 2009). Those thermal trials used a warming rate of 1 °C day^−1^ and 1 °C per 3 days. However, subsequent experiments using a more rapid warming rate of 1 °C h^−1^ and long‐term acclimation trials suggested that this rule was not universal (Peck and Morley, pers. obs.). Clearly, more detailed evaluations are required to understand the mechanisms underlying both acute and chronic responses to environmental change. Can we, for example, group species into different ‘types’ of stress response (or different levels of stress response) to empower the predictions of the response of biodiversity to climate change with the ultimate goal of accurately defining future ecosystem structure and stability? Whilst Krogh (1929) is largely remembered for advocating the use of model organisms, it is often forgotten that he also emphasized the need to study diversity (Chown & Gaston, 2016). Indeed, previous analyses examining cellular responses to environmental change, even in the same species, have been confounded by variability in external factors, making it difficult to dissect species’ cellular responses from experimental variability and identify whether there are any truly universal biomarkers to environmental change (cf. Clark *et al*., 2016).

Approaches which seek to provide unifying concepts to explain responses to environmental stress include identification of a conserved cellular stress proteome (Kültz, 2005), the accumulation of toxic oxidized proteins (Powell *et al*., 2005) and oxygen‐ and capacity‐limited thermal tolerance at the level of whole‐animal physiology (Pörtner, 2010). In the latter, warming produces an increase in metabolism leading to a mismatch between oxygen demand and supply, which if maintained leads to time‐limited thermal tolerance, reduced aerobic scope and loss of performance levels. The initial period of passive heat resistance can see the activation of a number of cellular pathways [cf. stress proteome (Kültz, 2005)] including the up‐regulation of antioxidant defences and the heat shock response [which has often been proposed as a universal response to stress (Gross, 2004)]. At higher temperatures, survival becomes time limited and is characterized by the transfer to anaerobic metabolism. In the marine environment, exposure to warming is also accompanied by a reduction in the solubility and concentration of oxygen (by 40% between 0 and 15 °C) and therefore potentially results in functional hypoxia, exacerbating oxygen demand (Abele & Puntarulo, 2004). The ability to tolerate the accumulation of anaerobic end products has been associated with resilience to environmental stress (De Zwaan & Eertman, 1996), and in addition, increased ambient oxygen has been proven to increase resistance to warming under a variety of experimental ramping protocols (Weatherly, 1970; Mark *et al*., 2002; Pörtner *et al*., 2006; Peck *et al*., 2007). However, these ramping experiments were conducted at what would be considered intermediate rates of experimental warming with a 1 °C increase every 12 h (Mark *et al*., 2002; Pörtner *et al*., 2006) and there is uncertainty as to whether oxygen limitation can explain the reduction in performance levels over all rates of warming and timescales (Peck *et al*., 2009).

This study expands on previous physiological evaluations of thermal tolerances (Peck *et al*., 2009) with a multi‐omics approach comprising nuclear magnetic resonance (NMR) spectroscopy‐based metabolomics and RNA‐Seq transcriptomics. These provide complementary approaches to discover the molecular mechanisms underlying Antarctic species sensitivities to warming. Transcriptomics can provide specific insights into the potential response of the animal (especially if highly conserved gene pathways are up‐regulated). However, these transcripts may not necessarily be translated into active end products, which is where metabolomics studies provide essential additional information (Hines *et al*., 2007; Viant, 2007). Whilst the difficulties of metabolite identification often limit the interpretation of metabolomics data, especially from nonmodel species, metabolites do represent functional molecules which can be predictive of animal physiology (Viant, 2007). In particular, metabolomics is very effective at identifying anaerobic end products (Hochachka & Somero, 2002), which are one of the foci of this study, as these would be expected to be produced as the animals transfer from passive heat resistance into anaerobic metabolism. As molecular information in Antarctic species is limited, utilizing these two ‘omics techniques in a comparative approach leverages the advantages of both techniques, enhancing abilities to decipher responses to warming within an ecological context (Viant, 2007).

In this study, we took six diverse Antarctic marine invertebrates with different locomotory habits and subjected them to rapid rates of warming (1 °C h^−1^). These comprised a highly active crustacean *Paraceradocus miersi*, a sessile‐attached brachiopod *Liothyrella uva*, two infaunal bivalve molluscs, *Laternula elliptica*,* Aequiyoldia eightsii*, (previously known as (*Yoldia eightsii*) a locomotory gastropod mollusc *Marseniopsis mollis* and an attached echinoderm *Cucumaria georgiana* (previously known as *Cucumaria attenuata*). These are common species, all widely distributed around the Antarctic (Dell, 1972; Foster, 1974; Coleman, 1989; O'Loughlin *et al*., 2011), and their thermal limits have been previously tested at both 1 °C day^−1^ and 1 °C 3 days^−1^ (Peck *et al*., 2009). In addition, we already have valuable background information on the ecology and physiology of these species with *L. elliptica* particularly well studied. It is the largest Antarctic marine bivalve contributing the highest ecological biomass and plays a significant role in benthopelagic coupling (Ralph & Maxwell, 1977; Ahn, 1993). Growth rates and more detailed analyses of responses to temperature have been carried out in several of the species: *L. elliptica* (cf. Peck *et al*., 2002); *L. uva* (cf. Peck *et al*., 1997); *M. mollis* (Peck *et al*., 2006); and *A. eightsii* (Peck & Bullough, 1993; Abele *et al*., 2001). In addition, *P. miersi* is one of the largest Antarctic amphipod and has proven a useful model to examine nerve conductance related to temperature (Young *et al*., 2006) and *M. mollis* has been particularly studied in relation to chemical defence (McClintock *et al*., 1992). These are all highly abundant species in the near‐shore marine fauna around the Rothera Research base, and therefore, any change in their biomass would significantly impact on ecosystem functioning.

The aim of this series of experiments was to test to what extent the current unifying physiological and cellular concepts applied to warming responses and whether they were underpinned by unifying molecular mechanisms. Rapid warming was chosen specifically to test acute responses, to identify whether survival correlated with activity levels and test the hypothesis that resilience correlated with the ability to tolerate anaerobic end products as a result of the transfer to anaerobic metabolism. NMR metabolomics was applied to all species to identify the biodiversity of the significantly changing metabolites, including anaerobic end products, with a subsequent LC‐MS study on *A. eightsii*, to provide added sensitivity and greater insight into the response of the most resilient species. These data provided intriguing results, and therefore, complementary transcriptomic sequencing was subsequently conducted to further identify the biodiversity in the response of biochemical pathways as a result of the warming experiment.

## Materials and methods

### Animal collection

The six species used in experimental work (*P. miersi*,* L. uva*,* L. elliptica*,* A. eightsii*,* M. mollis* and *C. georgiana*) were chosen because they demonstrated a wide range of thermal tolerances in their upper lethal temperatures (ULT) at a warming rate of 1 °C h^−1^ (Peck, pers. obs.) (Table 1). The animals were collected at Rothera Research Station, Adelaide Island, Antarctic Peninsula (67°34′07″S, 68°07′30″W), by SCUBA divers during the austral summer at depths of 10–15 m. Animals were acclimated in the flow‐through aquarium facility at Rothera for 2 weeks prior to the experiment, and the control animals (*n* = 10 for each species) were maintained in the same system. Temperature in the control tanks (500 l) was maintained at external ambient sea temperatures using flow‐through water pumped directly from the sea at rates in excess of 20 l min^−1^. The whole aquarium system was held in a controlled temperature room where air temperatures were set at 0.5–1.0 °C.

**Table 1:** Details of species, thermal tolerances and sampling temperatures. Thermal tolerance was based on upper lethal temperature trials conducted at a warming rate of 1 °C h^−1^ (based on Peck, pers. obs.). With the exception of *Paraceradocus miersi*, animals were sampled across a temperature range depending on responsiveness

Species	Taxa	Thermal tolerance	Temperature range of animals sampling (°C)	Mean temperature animals sampled at (°C)
*Aequiyoldia eightsii*	Bivalve mollusc	Very high	24.8–25.8	25.0
*Laternula elliptica*	Bivalve mollusc	High	17.8–23.9	19.9
*Liothyrella uva*	Brachiopod	Intermediate	17.3–19.6	18.1
*Cucumaria georgiana*	Echinoderm	Intermediate	14.7–16.0	15.2
*Paraceradocus miersi*	Crustacean	Intermediate	15.1	15.1
*Marseniopsis mollis*	Gastropod mollusc	Low	11.6–13.0	12.0

### Thermal trials

**Figure 1:**
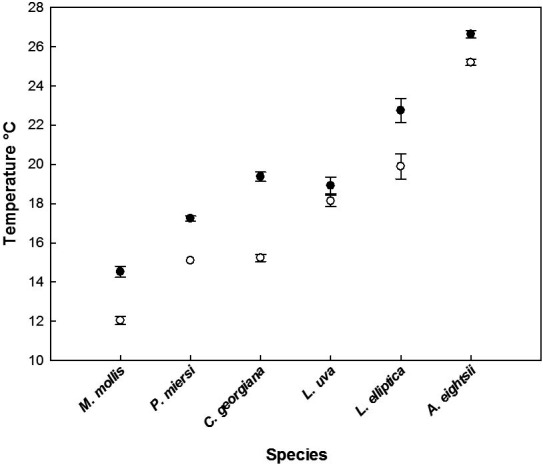
Upper lethal temperatures at a warming rate of 1 °C h^−1^ (Peck, pers. obs.) compared with sampling temperatures in this experiment.

All species were collected from the same site, tested in the same week (to negate temporal and seasonal effects) and subjected to the same ramping of temperature, with each animal being sampled when they were just below the level of being unresponsive and below their ULT, using ULT trials conducted in 2012 as a guideline (Peck, pers. comm.) (Fig. 1). This ensured that they were still metabolically active. Thus, as far as possible, each species was subjected to the same level of heat stress to provide directly comparable data. Animals (*n* = 10) were placed in 75‐l internal volume tanks with hollow walls, through which water was pumped from a temperature‐controlled unit. One tank was used for the ten animals. The whole system was placed inside a temperature‐controlled room and the system held at 0 °C. After transfer of the animals to the experimental system, they were warmed at a rate of 1 °C h^−1^. Animals were checked every 30 mins and were sampled when they responded poorly to tactile stimuli, such as touching or prodding with a blunt seeker (*L. elliptica*) or appropriate behavioural stimuli such as movement of antennae (*M. mollis*), retraction of tentacles (*C. georgiana*), failure to rapidly close the shell (*A. eightsii* and *L. uva*) or movement of parapodia (*P. miersi*).

### Animal and tissue sampling

Immediately following the thermal treatment, the animals were measured [wet weights (±0.001 g) where appropriate, length and height of shell (±0.01 mm)] before being snap frozen in liquid nitrogen. The animals were either frozen whole or the tissues were dissected out prior to freezing as detailed. The majority of samples comprised tissue mixes with a high proportion of muscular tissue: *P. miersi*: whole animal was frozen, with the tissue sample taken from one of the abdominal segments incorporating a pair of pleopods; *L. uva*: the lophophore was dissected out, but not used in the analyses as it is highly calcified, the rest of the animal tissue was mixed and used for the NMR and RNA‐Seq; *L. elliptica*: five tissues were sampled: siphon, mantle, foot, gill and digestive gland (all five were used in the NMR, but only mantle and gill in the RNA‐Seq); *A. eightsii*: the foot of the animal was dissected out and stored separately. The remainder of the animal was used as a mix for the NMR samples; *M. mollis*: only foot muscle was taken and *C. georgiana*: the whole animal was frozen, but the end portion of the animal close to the cloaca (cross‐section of muscular body wall) was taken for NMR and RNA‐Seq. A set of controls kept at 0 °C (*n* = 10 for each species) was sampled at the same time. Tissues were stored at −80 °C until analysed. The thermal tolerance trial had to be repeated for *P. miersi*, as the RNA had degraded in the samples used for the metabolomics extractions. The same protocol was strictly followed in this repeat trial.

### Metabolite extraction protocol

Approximately 50–100 mg of frozen tissue was used for each extraction. Samples were homogenized in a Precellys bead‐based homogenizer (Stretton Scientific, Stretton, UK) using ceramic beads (Stretton Scientific) as described by Wu *et al*. (2008) with the following modifications. In general, tissues were subjected to 2 × 10 s homogenization at 6400 rpm, but where tissues were particularly recalcitrant, for example *C*. *georgiana* and *M*. *mollis*, three rounds of 2 × 10 s homogenization were used. When the homogenates were removed from the Precellys tubes to clean 1.8‐ml glass vials, an additional wash step was added. Instead of adding 4 μl mg^−1^ chloroform directly to the sample in the glass vials, 4 μl mg^−1^ chloroform and 2 μl mg^−1^ of double‐distilled water were first added to the Precellys tubes and vortexed lightly before adding to the previous homogenate in the glass vials to ensure that all homogenized tissue was carried over for the next step. Both polar and nonpolar fractions were removed into separate 1.8‐ml glass vials using a Hamilton syringe and stored at −80 °C before further processing, although only the polar fraction was used for the following analyses. The polar metabolites were then transferred to a 1.5‐ml microcentrifuge tube. Each metabolite sample was dried using a centrifugal concentrator (Thermo Savant, Holbrook, NY, USA) and stored at −80 °C.

### NMR spectroscopy of tissue extracts

The dried polar extracts were resuspended in sodium phosphate buffer [0.1 m in 10% D_2_O and 90% H_2_O, pH 7.0, containing 0.5 mm sodium 3‐trimethylsilyl‐2,2,3,3,‐d_4_‐propionate (TMSP: chemical shift standard)]. The tissue extracts were analysed on the DRX‐500 NMR spectrometer (Bruker Biospin, Coventry, UK) equipped with a cryoprobe and operated at 500.18 MHz (at 300 K). One‐dimensional (1‐D) ^1^H NMR spectra were obtained using excitation sculpting for water suppression and a 8.4 μs (60°) pulse, 6 kHz spectral width and a 2.5 s relaxation delay with water presaturation. A total of 64 transients were collected into 16 348 data points, requiring a 4.5‐min acquisition time. The data sets were zero‐filled to 32 768 points, before line broadenings of 0.5 Hz were applied prior to Fourier transformation. To maximize metabolite discrimination, two‐dimensional (2‐D) ^1^H J‐resolved (JRES) NMR spectra were also acquired for each sample using eight transients per increment, for eight increments, which were collected into 16 384 data points with spectral widths of 6 kHz in F2 (chemical shift axis) and 50 kHz in F1 (spin–spin coupling constant axis). A 4.0‐s relaxation delay was employed resulting in a total acquisition time of 24 min. The data sets were zero‐filled in F1; the F2 dimension was then multiplied by a SEM window function using 0.5 Hz line broadening whilst the F1 dimension was multiplied by a sine‐bell window function, all prior to Fourier transformation. The JRES spectra were tilted by 45°, symmetrized about F1 and calibrated using TopSpin (Bruker Biospin). The data were exported as the 1‐D skyline projections of JRES spectra (pJRES) and converted to a format for multivariate analysis using custom‐written prometab software in matlab (version 7.1; The MathsWorks, Natick, MA, USA; Viant *et al*., 2003).

### NMR spectral processing

The 220 pJRES spectra were split according to control and treated tissue of each species. The 220 2‐D JRES NMR spectra were processed and skyline projection pJRES spectra were calculated in the matlab programming environment using nmrlab software. The pJRES spectra were baseline corrected, unwanted spectral regions (containing TMSP, water and a signal between *δ*_H_ 2.21–2.26) were excluded, and spectra were binned to a bin width of 0.005 ppm, normalized and g‐log transformed using the lambda parameter 1e‐7. The variables were mean centred prior to principal component analysis (PCA). All comparisons were subjected to both PCA and partial least squares discriminant analysis (PLS‐DA). For identification and quantitation of specific metabolites, peak areas were fitted and measured using the Chenomx software and normalized to the TMSP reference peak within each sample.

### LC‐MS metabolomics of tissue samples

To obtain greater detail on the metabolite profile of the most thermally resilient species, a limited trial of LC‐MS was carried out on the *A. eightsii* sample aliquots (rest of animal – minus foot). The polar extract aliquots designated for MS for 10 samples each of AeRC (control) and AeRT (heat treated) were first used to pool a QC sample from all of them, then dried down and applied to UHPLC‐MS on a 100 × 2.1 mm Thermo Hypersil Gold column with a flow rate of 400 μl min^−1^. The 15‐min programme on the Ultimate 3000 UHPLC system contained a gradient from 100% A (0.1% formic acid in water) to 100% B (0.1% formic acid in methanol). The LTQ‐FT Ultra mass spectrometer acquired data in positive ion mode from *m*/*z* 100–1000 at 50 000 resolution and in centroid mode. To avoid salt contamination, the first 30 s of the LC run was diverted away from the mass spectrometer. Additional MS/MS data were acquired on the same mass spectrometer.

### LC‐MS spectral processing

Data were processed by converting the Thermo .raw files into netcdf (.cdf) format using Thermo's XCalibur software; then an R script containing XCMS and CAMERA command (Brown *et al*., 2009; Dunn *et al*., 2011) was run to create an output in form of a csv file, containing an intensity matrix, *m*/*z* values, retention times and other information. A Taverna‐based workflow was used to match the output against the Manchester Metabolite Library (MMD; Brown *et al*., 2009), and it was also inserted into the standard MATLAB‐based Birmingham DIMS workflow (Kirwan *et al*., 2014) for alignment and sample filtering. Further in‐house matlab scripts were used for subsequent matrix processing: normalization using the PQN algorithm, filling missing values using KNN with *k* = 5, and, for multivariate statistics, g‐log transformation. Further annotation of metabolites was carried out by searching the extended peaklist against the KEGG (http://www.genome.jp/kegg/pathway.html) and LipidMap databases (LIPID MAPS Lipidomics Gateway, http://www.lipidmaps.org), using the MIPack package (Weber & Viant, 2010).

### Statistical analysis of metabolomics data

Multivariate analyses (PCA and PLS‐DA) were performed using the Eigenvector PLS Toolbox. For PCA, the resulting PCs were tested for significant separations between control and treated tissues using an in‐house matlab script applying anova and a Tukey test (PC scores test). PLS‐DA models were created in parallel and tested using an in‐house matlab script applying cross‐validation using a venetian blinds method (permutation testing). The bins used in the model were then reduced by forward selection of variables to the optimum for a robust separation of classes based on variable importance for projection (VIP) scores. The optimized models were again permutation tested. For NMR data, for both PCA and PLS‐DA models identification of metabolites in the 25 highest PC loadings for each significant PC, and those with forward‐selected VIP values, respectively, a two‐tailed *t*‐test was performed on the peak areas of these metabolites. The *P* values were adjusted for multiple testing using a 5% false discovery rate [FDR, after Benjamini & Hochberg (1995)] correction (*q* values). For LC‐MS data, univariate statistics were carried out on the whole normalized matrix after filling missing values in form of a two‐tailed *t*‐test with a 5% FDR correction (*q* values). At the same time, this in‐house script determined fold changes.

### RNA extraction

RNA was extracted from *n* = 5 for each set of control and treated animals using TRI reagent (Bioline, London, UK) and purified on RNeasy mini columns (Qiagen, Hilden, Germany) according to manufacturer's instructions. RNA was quantified using an Agilent Technologies Tape Station 2200. These were the same animals (with the exception of *P. miersi*) that had been used in the metabolomics analyses to provide a directly comparable analysis. The RNAs were subjected to RNA‐Seq on an Illumina Hi‐Seq 2000 at The Genome Analysis Centre (TGAC), Norwich.

### Transcriptomic analysis

The raw Illumina reads were assembled using SOAP denovo (soap.genomics.org.cn) with transcripts >500 bp used for both producing the backbone sequence database and also for mapping differential expression patterns. Selection for differential expression was carried out using a stringent twofold expression‐level difference, and the use of a linear model in Bayseq (Hardcastle & Kelly, 2010) using a model drawing on replicate individuals (Guo *et al*., 2013). A further adjustment was made for multiple testing (Benjamini & Hochberg, 1995) with an FDR cut‐off set at 0.01. Only mappings where both paired‐end reads mapped to the same contig were used to generate expression levels and calculate significance of expression. Contigs were searched for sequence similarity using Blast (Altschul *et al*., 1997) against the GenBank nonredundant database (Benson *et al*., 2007) with a threshold score of <1e^−10^. All annotations were manually verified, where possible and the putative annotations binned into generic cellular pathways.

### Data submission

All sequence data were submitted to the NCBI Short‐Read Archive (SRA) with the accession numbers: *P. miersi*: SRP071221; *L. elliptica*: SRP071174; *L. uva*: SRP071176; *A. eightsii*: SRP071173; *M. mollis*: SRP071177; *C. georgiana*: SRP070764. All metabolomic profiles have been submitted to METABOLITE and can be viewed at http://www.ebi.ac.uk/metabolights/MTBLS336.

## Results

Each species was warmed at a rate of 1 °C h^−1^ and sampled within a very small temperature window at between 0.8 and 4.1 °C below their ULT, as previously determined in experiments performed 2 years earlier (Peck, pers. comm.) (Fig. 1; Table 1). The relative thermal sensitivity of each species in this experiment was in the same order as previously identified in the ULT trials (Fig. 1): *A. eightsii* was the most tolerant species sampled at a mean temperature of 25.0 °C, followed by *L. elliptica* (19.9 °C), *L. uva* (18.1 °C), *C. georgiana* (15.2 °C) and *P. miersi* (15.1 °C), with *M. mollis* having the lowest thermal tolerance, sampled at 12.0 °C (Fig. 1; Table 1). This order was different to that previously obtained under the different ramping regimes of 1 °C day^−1^ and 1 °C 3 days^−1^ (Peck *et al*., 2009) where more active animals survived to higher temperatures than sessile ones. In this experiment, *P. miersi*, the most active species, and the locomotory gastropod *M. mollis* were the most thermally sensitive with the infaunal bivalves and sessile‐attached brachiopod, the most resilient. There was no significant difference in size of animals between the control and treated groups for any of the species (Table S1), and all animals were reproductively mature.

### NMR metabolomics

**Figure 2:**
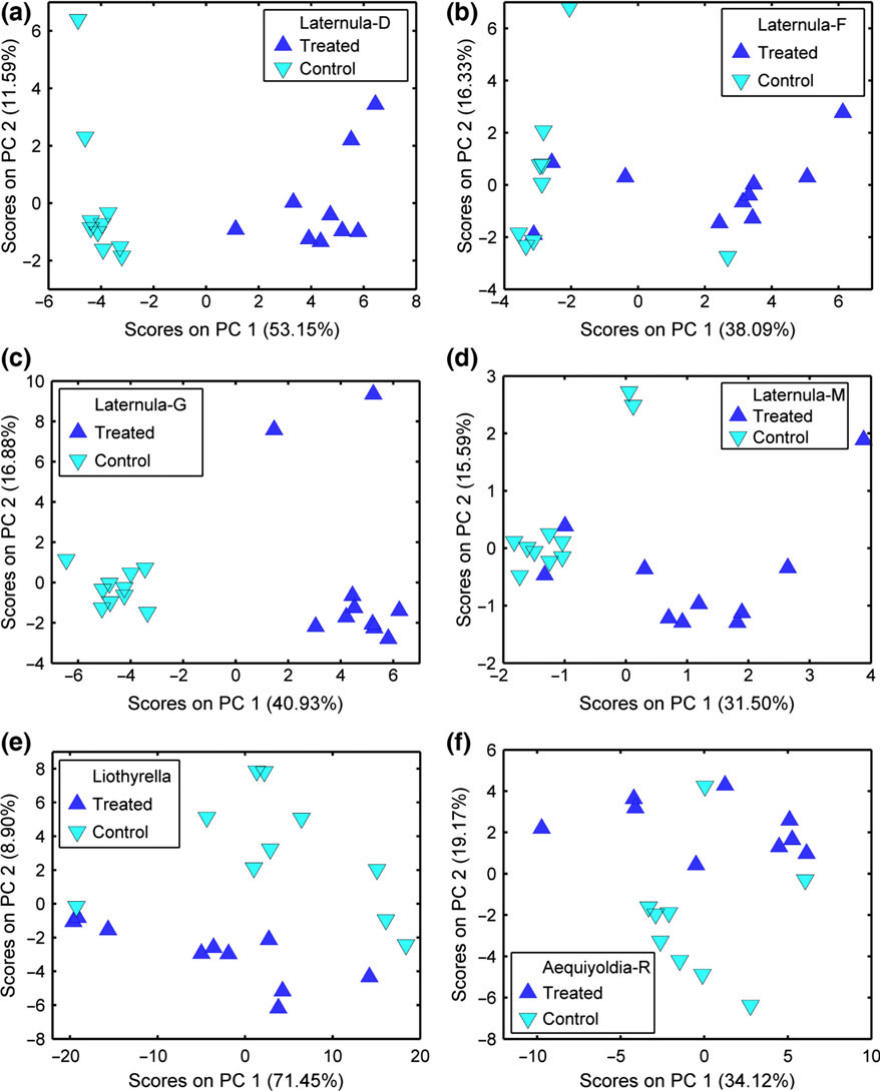
Principal component analysis (PCA) plots showing separation of control and treated groups for *Laternula elliptica* (a) mantle; (b) foot; (c) gill; (d) digestive gland; (e) *Liothyrella uva* (whole‐animal tissue mix); (f) *Aequiyoldia eightsii* (whole‐animal tissue mix).

The PCA analysis showed that there were PC scores that gave significant separations between the control and treated samples for all tissues of *L. elliptica* (siphon, mantle, foot, gill and digestive gland), whole‐animal tissue mix of *L. uva* and whole‐animal tissue mix of *A. eightsii* (Fig. 2). PC scores did not show any significant separations for the foot tissue of *A. eightsii* or the whole‐animal tissue mixes extracted from *P. miersi* and *C. georgiana* or the foot of *M. mollis*. Further analysis identifying metabolites from the top 25 loading plots and adjustment for multiple testing verified these results with the exception that separation was no longer statistically significant for one tissue from *L. elliptica* (siphon) (Data S1). Virtually identical results were produced with the PLS‐DA analysis when adjusted for multiple testing, except that PLS‐DA analysis forced a separation for the *A. eightsii* foot samples (Table 2).

**Table 2:** NMR‐based metabolomics: fold change in metabolite concentration for identified significant principal component analysis (PCA) and PLSDA results (*P* < 0.05) for the three more thermally resilient species

	Acetone	Alanine	Aspartate	Dimethyl amine	Dimethyl sulfone	Lactate	Succinate	Valine
*Laternula elliptica*								
Mantle						1.42	14.63	1.60
Foot							25.84	
Gill	0.61	2.45	0.55			4.07	94.73	3.84
Digestive gland						2.13	72.91	2.53
*Liothyrella uva*					0.16			
*Aequiyoldia eightsii*								
Foot		0.69						
Whole animal			0.53	0.51			7.91	

From the PLS‐DA results, between 11 and 21 metabolites were putatively annotated across all species (the exception being *C. georgiana* where only 1 was identified) (Data S1). These were tested for their significance using VIP scores for the full data set, with significant separation identified only for signals annotated as acetone, alanine, aspartate, dimethyl amine, dimethyl sulphone, lactate, succinate and valine in three species (Table 2). When the results were constrained to those metabolites showing a fold change greater than one, only *L. elliptica* and *A. eightsii* were retained, with the significant metabolites identified as arginine, lactate, succinate and valine (Table 2). There were many metabolites which were not identified due to weak signal intensity or overlapping complex signals and also due to problems identifying metabolites in nonmodel species. To investigate these ‘unknown’ metabolites, advanced chromatographic separations and NMR techniques would be required. These results correlated with the thermal tolerance of the species chosen, with only significant separation of metabolites identified between control and treated animals for the three most thermally resilient species (Table 3).

**Table 3:** Summary of combined metabolite and transcriptomic data sets, including the detail on the cellular pathways up‐regulated in response to animals being warmed at 1 °C per h, based on putatively annotated transcripts from RNA‐Seq experiments

Response	Species					
	*Mm*	*Pm*	*Cg*	*Lu*	*Le*	*Ae*
Physiological tolerance	Low	Intermediate	Intermediate	Intermediate	High	Very high
Tissue type	Foot	Mix	Mix	Mix	Gill	Mix
Number of transcripts up‐regulated	32	32	34	279	1871	79
Number of metabolites significantly changed	0	0	0	0	3	1
Heat shock response		X		X	X	
Anaerobic end products					X	X
Number of major pathways identified	2	2	4	8	7	5
Detail on pathways identified						
Heat shock response		X		X	X	
Neurotransmission		X				
Respiratory electron transport chain	X		X			
Apoptosis	X		X	X	X	X
Cytoskeleton			X	X	X	X
CYP450 detoxification			X	X	X	
Protein degradation				X	X	X
Immune response				X	X	X
DNA damage and repair				X		
Cell signalling and ion transport				X	X	X

X denotes a positive result. Species symbols: *Mm*,* Marseniopsis mollis*;* Pm*,* Paraceradocus miersi*;* Ca*,* Cucumaria georgiana*;* Lu*,* Liothyrella uva*;* Le*,* Laternula elliptica*;* Ae*,* Aequiyoldia eightsii*. Only the *Laternula elliptica* gill tissue results are presented here, as the mantle tissue did not show any significantly up‐regulated transcripts.

### Transcriptomics

The limited results (in terms of NMR identifications of anaerobic end products in only three of the six species) were intriguing and somewhat unexpected, given our prior hypothesis that resilience was related to the ability to tolerate anaerobic end products. Thus, it was decided to further investigate the response to warming on the same samples using a complementary transcriptomics approach. It was only possible to perform expression analyses on a more limited range of tissues and therefore where multiple tissues for a species were investigated using NMR, only one tissue type was chosen for *A. eightsii* (whole‐animal mixed tissue, as this produced a metabolomics result) and two tissues for *L. elliptica* [gill, as this showed the highest succinate levels and mantle as this tissue has been extensively investigated in similar transcriptomic experiments (cf. Truebano *et al*., 2010; Clark *et al*., 2010, 2016)]. The number of transcripts up‐regulated in each species when treated animals were compared with controls varied dramatically (Table 3). At the lowest end, *M. mollis* and *P. miersi* both showed 32 up‐regulated transcripts, followed by *C. georgiana* with 34, then *A. eightsii* with 79, *L. uva* with 279 and finally *L. elliptica* gill with 1871 (Tables S2–S7, respectively). The latter result in gill was in complete contrast to the transcriptomic results in *L. elliptica* mantle tissue, which showed no differential expression of transcripts between experimental and control animals and therefore the results of the mantle sequencing were not included in Tables 2 and 3. When the annotations produced from Blast sequence similarity searching of the up‐regulated transcripts were analysed in more detail, the putative functions could be assigned to a variety of pathways (Table 3). None of the species showed the same response to warming. Only three species demonstrated a heat shock response (*P. miersi, L. uva* and *L. elliptica*), with up‐regulation of members of the 70 kDa heat shock protein (HSP70) family. *P. miersi* showed major up‐regulation of putative transcripts involved in neurotransmission, whilst *M. mollis* and *C. georgiana* were the only species to express transcripts involved in the mitochondrial electron transport chain. In general, the least resilient species showed not only a lower number of up‐regulated transcripts, but also less diversity of response, in terms of the putatively identified cellular pathways (Table 3).

### LC‐MS metabolomics on *A. eightsii*

After the processing of the metabolomics and transcriptomics data, LC‐MS was applied for a deeper inspection of the metabolomic response of the most resilient species. Processing of the LC‐MS data resulted in a peak list and data matrix of 1470 signals. Both PCA and PLS‐DA analysis produced separation of treated and control samples (Data S2). The forward selection of variables by VIP score to obtain an optimized PLS‐DA model resulted in 26 signals, whilst the *t*‐test resulted in 25 signals with significant *q* values (*P* values after FDR correction), with 14 of these signals found on both lists. Of these seven were annotated as being likely versions of the same molecule, succinate, which is significantly up‐regulated in the treated samples and corresponds to the NMR results (Table 4). Also leucine or isoleucine and a signal annotated as *O*‐propanoylcarnitine were up‐regulated and on both lists, whilst several probably inorganic signals decreased. The forward‐selected variables also contained a compound annotated as *O*‐butanoylcarnitine and also tryptophan retained a significant *q* value (both increased). The rest of these compounds including a putative peptide and a very polar sulphur‐containing ion could not be reliably annotated.

**Table 4:** LC‐MS‐based metabolomics: fold change in metabolite concentration for safely annotated significantly changed metabolites (PLS‐DA and *t*‐test) of *Aequiyoldia eightsii* (whole animal, not the foot)

Annotation	Fold change
Leucine or isoleucine	2.4
Succinate	2.4–12.8^a^
Tryptophan	2.1
O‐propanoylcarnitine	1.7
O‐butanoylcarnitine	2.0^b^

a Range of fold changes for different adduct forms of succinic acid.

b Important only in multivariate statistics.

## Discussion

These data demonstrate, for the first time, the wide diversity in response to warming in six Antarctic marine species. All species were collected from the same site, tested in the same week and subjected to the same ramping of temperature. Thus, as far as possible, each species was subjected to the same level of heat stress to provide directly comparable data. The difference between the mean sampling temperature compared to the mean ULT varied with species, but was not unexpected as ULTs can vary annually depending on conditions such as food supply and long‐term habitat temperatures (Morley *et al*., 2012). In this experiment, the sampling temperature erred on the cautious side so that the upper lethal limit was not reached.

Previous studies comparing the stress response of different species have been hampered by variance in the experimental protocol, either the methodology of application of the stress, different stresses or timings (cf. Clark *et al*., 2016 and discussion therein). Therefore, it has previously been difficult to differentiate between treatment effect and species‐specific response. These factors have made the identification of the diversity of response exhibited between species difficult to achieve.

The major paradigm around species inability to cope with changed conditions, specifically to resist warming, is based on oxygen limitation (Pörtner, 2010). In fact, exposure to critical warming, as in the rapid rates here, is accompanied by a considerable reduction in the solubility and concentration of oxygen and therefore functional hypoxia. This would exacerbate the problems from warming, with induced increases in oxygen demand (Abele & Puntarulo, 2004). From this paradigm of oxygen limitation, it was expected that as each species approached their critical temperature, they would switch to anaerobic respiration which could be demonstrated by the accumulation of anaerobic end products (cf. Pörtner, 2010) and tolerance of such products has been associated with enhanced survival (De Zwaan & Eertman, 1996). A range of anaerobic end products has been documented in invertebrates, aside from the widely observed lactate, several of which are common metabolites, such as succinate, that can be detected by NMR (Hochachka & Somero, 2002). Previous analyses have shown the presence of anaerobic end products in invertebrate muscle tissue (reviewed in De Zwaan & Eertman, 1996) and with the exception of *L. elliptica* which was dissected into separate tissues, the samples from other species were tissue mixes, comprising a significant proportion of muscle. This use of muscle‐dominated tissue mixes maximizes the chances of detecting anaerobic end products and also significantly reduces the possibility of observing tissue‐specific responses. This is more difficult to achieve with larger species which have more defined tissues and are easier to dissect, such as demonstrated by *L. elliptica*, but with these animals, it is equally important to evaluate a range of different tissues to understand the whole‐animal responses (cf. Clark *et al*., 2016).

What was very surprising was that only three of the species (which happened to be the most resilient ones) showed significant differences observed in separation of metabolites between control and treated animals. Hence, only half of the species studied showed a response to acute warming in their metabolite profiles. Although a range of metabolites were identified in the NMR study across all the species, only alanine, lactate, succinate and valine were significantly up‐regulated in the NMR study in *L. elliptica* and *A. eightsii*, the first three metabolites being classic end products of anaerobic respiration in marine bivalves (Table 2) (De Zwaan & Eertman, 1996; Hochachka & Somero, 2002). Whilst valine has been shown to be produced in response to both anoxia and heat stress in other species, the exact reason is unknown (Mayer *et al*., 1990; Kaplan *et al*., 2004; Podrabsky *et al*., 2007). In general, any animal exposed to environmental stress has shown a perturbation of the amino acid pool. It has been suggested that this may be due to either the possible degradation of proteins, a decrease in consumption of amino acids associated with the arrest of protein synthesis, balancing intracellular osmolarity or to support secondary metabolite production as part of an immune defence mechanism (Kaplan *et al*., 2004; Podrabsky *et al*., 2007; Ellis *et al*., 2014). In *A. eightsii*, these data were later supplemented with an LC‐MS study (Table 4), which showed the accumulation of succinate (validating the NMR result) (Tables 2 and 3), along with two free amino acids (leucine/isoleucine and tryptophan) and *O*‐propanoyl carnitine. The putative detection of *O*‐propanoyl carnitine was intriguing, as it has not been identified in metabolite studies of invertebrates before. It is involved in the carnitine pathway which plays an important role in fatty acid and energy metabolism. Whilst it may be present in *A. eightsii* as an intermediate of this pathway, it has also been demonstrated to induce an antioxidant defence against the lipid peroxidation of membranes in mammals (Sayed‐Ahmed *et al*., 2001).

It is perhaps pertinent that the two species which showed accumulation of alanine, lactate and succinate are bivalve molluscs that regularly experience periods of hypoxia. Therefore, they are habituated to shutting down their metabolism in times of stress and tolerating increased levels of anaerobic end products (cf. De Zwaan & Eertman, 1996). For example, *L. elliptica* shows a seasonal pattern of long periods of siphon closure in the winter when its phytoplankton food source is in scarce supply (Morley *et al*., 2007). This may be the reason for the more limited metabolomic response in the siphon and mantle and the lack of a significant transcriptomic response in the mantle, with these organs being shut down and cellular energy concentrated in the more vital organs. *A. eightsii* is a bioturbating deposit feeder that carries out vertical feeding migrations in the sediment. It is completely buried whilst feeding and thus regularly experiences hypoxic conditions (Davenport, 1988). The metabolomics results represent a conundrum. It was expected that anaerobic end products would accumulate in all species, not just the two bivalves. Therefore, to try and decipher the mechanisms underlying the response to rapid warming further, it was decided to extend this study by examining the gene expression profiles of exactly the same tissue samples studied by metabolomics. The gene expression data identified an even more diverse range of species‐specific responses.

*Marseniopsis mollis* was the most thermally sensitive species and essentially lacked a transcriptomic response (Table 3). The putative annotations almost solely comprised of enzymes in the electron transport chain (NADH dehydrogenase and cytochrome c) (Table S2), indicating that the animals were respiring aerobically. Hence, there was almost a complete absence of a recognizable cellular stress response (cf. Kültz, 2005).

*Paraceradocus miersi* was the second most thermally sensitive species in this experiment. Surprisingly, all the experimental animals succumbed at exactly the same temperature. Blast sequence similarity searching produced many matches to InterPro annotated domains, several of which revealed putative connections with neuronal functions, for example contig 4656385 (structural integrity of nerve terminals), contig 4630105 (sodium symporter with neurotransmitter activity) and contig 4676103 (synaptojanin involved in synaptic vesicle recovery) amongst others (Table S3). Whilst these annotations were tentative, given the evolutionary distance between *P. miersi* and the vertebrate‐centric data, the fact that many domain matches had putative neuronal connections added weight to the hypothesis that neuronal collapse may be the reason for failure of this species at 15.1 °C and the potential reason why all the animals failed at the same temperature. A test of the thermal characteristics of neuronal conduction in *P. miersi* indicated an upper thermal block in conduction at 21.5 °C (Young *et al*., 2006). This is considerably higher than the ULT in the current experiment, but the conduction test was run at the even faster rate of warming of 1 °C every 4 min. If the animals were warmed at the same rate, it is entirely possible that they would have achieved this temperature, even though whole animals tend to fail at lower temperatures than their individual biochemical pathways (Pörtner *et al*., 2006). A particularly interesting discovery in *P. miersi* was the up‐regulation of an HSP70 transcript (Table S3). Previous analyses of heat shock genes in this species failed to reveal a heat shock response, but the analyses were limited to a handful of HSP70 transcripts obtained via degenerate PCR and not the transcriptome‐led approach here (Clark *et al*., 2008a). The HSP70 transcript identified here did not correspond to any gene fragments previously isolated and thus brings the total of HSP70 genes identified in *P. miersi* to 5, which is approaching a similar level to that of other highly active Antarctic crustaceans, krill *Euphausia superba* and ice krill *Euphasia crystallorophias* (Cascella *et al*., 2015).

The last more thermally sensitive species was the sea cucumber, *C. georgiana*. Similar to *M. mollis*, there was an impoverished transcriptomic response with evidence of up‐regulation of enzymes in the electron transport chain (NADH dehydrogenase and cytochrome c) and aerobic respiration (Table 3). However, there were also additional annotations to the cytoskeleton (contig 7122771), detoxification (CYP450, contig 7122369), apoptosis (contigs 7140781, 7152303 and 7099238) and tropomyosin with the stabilization of stress fibres (contigs 7090962 and 7129591) (Table S4), which indicated that this species did invoke a type of stress response. There was no heat shock response (HSR).

This lack of an HSR was not entirely unexpected, because although the HSR is often the first line of defence to increased temperature (Parsell & Lindquist, 1993), Antarctic marine species often fail to exhibit such a response (reviewed in Clark & Peck, 2009), which has been suggested to be the result of adaptation to millions of years adaptation to life in the cold. Whilst the lack of a response in the notothenioid fish has been shown to be the result of a mutation in the promoter of the inducible form of HSP70 (Buckley *et al*., 2004), in the other species, including those investigated here, it is more likely that this particular stress did not induce an HSR or the up‐regulation of classical stress proteins, such as antioxidants. It is entirely possible that under different stresses, either an HSR or a more complex stress response would be produced, as shown previously, in both the limpet *Nacella concinna* and the clam *L. elliptica* (Clark & Peck, 2009). As additional evidence is the fact that previous analyses of the HSR of *P. miersi* (previously known as *Paraceradocus gibber*) failed to identify the HSP70 genes and HSR response (Clark *et al*., 2008a) which was evident here. Clearly, more extensive analyses over different ranges of temperatures would be required to investigate whether the species described here, which fail to exhibit such a response, really do lack an HSR.

Transcriptome analysis of the three more thermally resilient species revealed a much more comprehensive response, suggesting that the capability to respond at the molecular level may underlie tolerance (Table 3) (Tables S5–S7). This situation was very similar to gene expression analyses previously conducted on *L. elliptica* comparing the hypoxic response of young and older animals. The younger animals were far more resilient with the putative transcript annotations, indicating a more active molecular response to environmental change (Clark *et al*., 2013, 2016). In other studies, more tolerant species have also tended to produce a more active response against cellular damage (Dong *et al*., 2008; Lockwood *et al*., 2010; Hasanuzzaman *et al*., 2013). Of the three resilient species, only two, *L. uva* and *L. elliptica* showed an HSR (Tables S6 and S7). An HSR had previously been described in *L. elliptica* (Clark *et al*., 2008b), but it is the first time that the HSR has been identified in *L. uva*. The other pathways identified in the up‐regulated transcripts of these three species were a mixture of different pathways (Tables 3 and S5–S7). The presence of transcripts putatively involved in cell signalling and ion transport indicates that all three resilient species were still metabolically active and not shutting down their metabolism in response to the heat (Podrabsky *et al*., 2007). These processes, along with protein synthesis, are some of the most energetically expensive for the cell and the first to be shut down when conditions become difficult (Buttgereit & Brand, 1995). So why did these animals fail?

It was interesting to note that in none of the species was there evidence of the classical response to reactive oxygen species (ROS) damage, with no transcripts showing sequence similarity to superoxide dismutase (SOD), catalase and the glutathione enzymes. This was unexpected as these are often present in the ‘resting’ transcriptome of Antarctic species, which is thought to be a response to life in the hyperoxygenated waters of the Southern Ocean and the increased potential for damage from ROS (Abele & Puntarulo, 2004; Chen *et al*., 2008; Clark *et al*., 2010, 2011). In this respect, there may be clues from previous work in Antarctic bivalves, in which measurements were made of the antioxidant enzyme SOD and also the pro‐oxidative product malondialdehyde (MDA). The latter is one of the first products of lipid peroxidation in the cell, which acts as a signalling system promoting cell death or cell survival and is often used as a biomarker of oxidative stress (Ayala *et al*., 2014). Whilst MDA levels increased in response to environmental stress in *A. eightsii* and *Adamussium colbecki* indicating that oxidative stress was occurring, SOD levels decreased (Regoli *et al*., 1997; Abele *et al*., 2001). It was suggested that Antarctic bivalves had maximized the activity of their antioxidant system at low temperatures to combat ROS damage associated with living in highly oxygenated freezing waters with a trade‐off of the thermal stability of its antioxidant system (Abele *et al*., 2001). In other systems, thermal denaturation (both warm and cold) of enzymes, such as succinate dehydrogenase, the immediate downstream enzyme of succinate in the Krebs cycle, has been proposed for the accumulation of succinate as a stress metabolite (Van Den Thillart & Smit, 1984; Michaud *et al*., 2008). Hence, these data provide examples of species‐specific enhanced sensitivity of critical enzymes which directly impact on organism physiology and survival. Antarctic marine species have evolved in isolation for the past 10–14 Myr (Clarke & Crame, 1992), but there are questions as to whether their proteins are completely adapted to the cold (Peck, 2016). The diversity shown here in response to acute warming may well reflect differential thermal sensitivity of critical enzyme systems. If this hypothesis is true, then the question arises as to how representative are arbitrarily selected sentinel species?

In conclusion, this experiment which treated six species of Antarctic marine invertebrate to the same acute stress, with each species taken to the same physiological end point (just prior to heat coma), is the first to dissect the species‐specific nature of the thermal stress response from the variability in response obtained from different experiments in nonmodel organisms, which occurs even when performed on the same species under similar conditions. It also demonstrated the utility of laboratory experimentation in identifying the biodiversity of responses. Of the six species, only two showed accumulation of anaerobic end products and three exhibited the classical HSR with expression of HSP70 transcripts, but these did not directly align with resilience to heat stress. This diversity in response does not align with current unifying concepts of thermal limits, which seek to explain species responses in a changing world. Whilst this experiment specifically targeted invertebrates with different known thermal tolerances and this resulted in a wide phylogenetic mix of species, future experiments should also investigate the responses of closely related species to identify whether there are taxa‐specific constraints on the response, or indeed, whether this biodiversity in response holds at different rates of temperature change (e.g. long‐term chronic challenge). However, as stated earlier, this may not be possible in all species due to a lack of knowledge in animal husbandry. Therefore, acute ramping experiments provide essential data on differential sensitivities over different rates of temperature change (Peck *et al*., 2009; Terblanche *et al*., 2011). These data clearly emphasize the need for a more macrophysiology‐type approach to understanding the cellular stress response and integration of these data to inform on the ecosystem consequences on future biodiversity (Buckley & Kingsolver, 2012; Chown & Gaston, 2016). Initially, such studies should concentrate on the highly abundant species (such as those described here) which contribute significant biomass to the marine ecosystem. Any changes in the population densities of these species will significantly impact on ecosystem balance and functioning, shaping future biodiversity in our oceans.

## Supporting information


**Table S1.** Morphometrics of control and treated animals.

**Table S2.** Transcripts up‐regulated in *Marseniopsis mollis* in response to acute thermal stress.

**Table S3.** Transcripts up‐regulated in *Paraceradocus miersi* in response to acute thermal stress.

**Table S4.** Transcripts up‐regulated in *Cucumaria georgiana* in response to acute thermal stress.

**Table S5.** Transcripts up‐regulated in *Aequiyoldia eightsii* in response to acute thermal stress.

**Table S6.** Transcripts up‐regulated in *Liothyrella uva* in response to acute thermal stress.

**Table S7.** Transcripts up‐regulated in *Laternula elliptica* gill in response to acute thermal stress

**Data S1.** Detail on PCA and PLS‐DA analyses.

**Data S2.** LC‐MS statistics and signal annotations.
