# Supplementary material for: Biodiversity in marine invertebrate responses to acute warming revealed by a comparative multi‐omics approach
Source: Glob Chang Biol. 2016 Jun 17;23(1):318–30. doi: 10.1111/gcb.13357 (PMC6849730; doi:10.1111/gcb.13357)
Supplement: Supplementary file 1 — Table S1. Morphometrics of control and treated animals. [file GCB-23-318-s001.pdf]

**Organism**	**Morphometrics**			**t-test**
	**Metric**	**Measurement ± SE mean**		
		**Controls**	**Treated**	
Cucumaria georgiana	Wet weight (g)	5.28 ± 0.53	6.59 ± 0.87	T = -1.29, P = 0.22, DF = 14
Laternula elliptica	Shell length (mm)	66.80 ± 3.72	67.77 ± 2.93	T = -0.20, P = 0.84, DF = 17
	Wet weight (g)	112.90 ± 18.40	109.70 ± 17.40	T = 0.13, P = 0.90, DF = 17
Liothyrella uva	Shell length (mm)	33.77 ± 1.24	32.42 ± 1.33	T = 0.74, P = 0.47, DF = 17
	Shell height (mm)	22.80 ± 0.98	23.47 ± 0.98	T = -0.48, P = 0.64, DF = 17
	Shell width (mm)	17.97 ± 1.40	17.59 ± 1.00	T = 0.22, P = 0.83, DF = 16
	Wet weight (g)	8.13 ± 1.00	7.18 ± 1.10	T = 0.61, P = 0.55, DF = 17
Marseniopsis mollis	Wet weight (g)	57.46 ± 5.20	51.42 ± 7.70	T = 0.65, P = 0.53, DF = 15
Paraceradocus miersi (M)	Wet weight (g)	0.96 ± 0.13	0.84 ± 1.14	T = 0.62, P = 0.54, DF = 17
Paraceradocus miersi (T)	Wet weight (g)	0.73±0.09	0.91±0.17	T=-0.98, P=0.359, DF=7
Aequiyoldia eightsii	Shell length (mm)	22.15 ± 3.19	22.09 ± 2.83	T = 0.04, P = 0.97, DF = 17
	Wet weight (g)	1.57 ± 0.75	1.59 ± 0.62	T = -0.05, P = 0.96, DF = 17

Supplementary Table S1: Morphometrics of control and treated animals. *Paraceradocus miersi (M)* indicates data for animals used in the metabolomics, *Paraceradocus miersi (T)* indicates animals used in the repeated transcriptomics experiment.
