# Supplementary material for: Biodiversity in marine invertebrate responses to acute warming revealed by a comparative multi‐omics approach
Source: Glob Chang Biol. 2016 Jun 17;23(1):318–30. doi: 10.1111/gcb.13357 (PMC6849730; doi:10.1111/gcb.13357)
Supplement: Supplementary file 2 — Table S2. Transcripts up‐regulated in Marseniopsis mollis in response to acute thermal stress. [file GCB-23-318-s002.pdf]

**Supplementary Table S2: Transcripts up-regulated in *M. mollis* in response to acute thermal stress**
Transcripts with annotations below 10-10 or no annotation not show

| contig | accession | evalue | description |
|---|---|---|---|
| 3630953 | gi\|171473594\|ref\|YP_001798462.1\| | 1.03378e-11 | NADH dehydrogenase subunit 4 [Trigoniophthalmus alternatus |
| 3617737 | gi\|134154087\|gb\|ABO64423.1\| | 1.15342e-51 | cytochrome oxidase subunit II [Drosophila polychaeta |
| 3644629 | gi\|339256140\|ref\|XP_003370555.1\| | 1.22604e-17 | conserved hypothetical protein [Trichinella spirali |
| 3641567 | gi\|520993079\|gb\|AGP25531.1\| | 2.34947e-99 | cytochrome c oxidase subunit I, partial (mitochondrion) [Yoldia eights |
| 3647671 | gi\|403334965\|gb\|EJY66653.1\| | 2.55368e-17 | hypothetical protein OXYTRI_13058 [Oxytricha trifalla |
| 3634107 | gi\|552808617\|ref\|XP_005842765.1\| | 2.78382e-16 | hypothetical protein CHLNCDRAFT_28884, partial [Chlorella variabili |
| 3645261 | gi\|405953564\|gb\|EKC21201.1\| | 2.81835e-17 | PC4 and SFRS1-interacting protein [Crassostrea gigas |
| 3640393 | gi\|357488221\|ref\|XP_003614398.1\| | 3.35634e-23 | hypothetical protein MTR_5g051170 [Medicago truncatula |
| 3628633 | gi\|321445829\|gb\|EFX60776.1\| | 3.45987e-19 | hypothetical protein DAPPUDRAFT_70655 [Daphnia pulex |
| 3623053 | gi\|403333060\|gb\|EJY65597.1\| | 3.48224e-16 | hypothetical protein OXYTRI_14248 [Oxytricha trifalla |
| 3619001 | gi\|403334966\|gb\|EJY66654.1\| | 4.26078e-12 | hypothetical protein OXYTRI_13059 [Oxytricha trifalla |
| 3619215 | gi\|575418853\|gb\|ETX03503.1\| | 4.55226e-14 | hypothetical protein ETSY1_47025 [Candidatus Entotheonella sp. TSY |
| 3616295 | gi\|678336953\|emb\|CDW75723.1\| | 4.7713e-20 | UNKNOWN [Stylonychia lemnae] |
| 3652985 | gi\|169794155\|ref\|YP_001718392.1\| | 7.98483e-93 | NADH dehydrogenase subunit 5 [Loxocorone alla |
| 3654985 | gi\|678336953\|emb\|CDW75723.1\| | 8.48339e-16 | UNKNOWN [Stylonychia lemnae] |
| 3646451 | gi\|575669575\|ref\|YP_009000282.1\| | 8.8836e-41 | NADH dehydrogenase subunit 1 (mitochondrion) [Semisulcospira libertina |
| 3624907 | gi\|676426511\|ref\|XP_009044647.1\| | 9.73762e-12 | hypothetical protein LOTGIDRAFT_202939 [Lottia gigantea |
| 3631355 | gi\|678336953\|emb\|CDW75723.1\| | 9.84052e-26 | UNKNOWN [Stylonychia lemnae] |
