# Supplementary material for: Biodiversity in marine invertebrate responses to acute warming revealed by a comparative multi‐omics approach
Source: Glob Chang Biol. 2016 Jun 17;23(1):318–30. doi: 10.1111/gcb.13357 (PMC6849730; doi:10.1111/gcb.13357)
Supplement: Supplementary file 3 — Table S3. Transcripts up‐regulated in Paraceradocus miersi in response to acute thermal stress. [file GCB-23-318-s003.pdf]

Supplementary Table S3: Transcripts up-regulated in *P. miersi* in response to acute thermal stress

Transcripts with annotations below 10-10 or no annotation not shown

contig	accession	evalue	description
4677701	gi|372001233|gb|AEX65806.1|	0	heat shock protein 70, partial [Eulimnogammarus vittatus]
4678967	gi|321471223|gb|EFX82196.1|	1.00381e-110	hypothetical protein DAPPUDRAFT_316814 [Daphnia pulex]
4629119	gi|350401510|ref|XP_003486177.1|	1.01514e-31	PREDICTED: protein bicaudal C-like [Bombus impatiens]
4652751	gi|321450057|gb|EFX62229.1|	1.28663e-67	hypothetical protein DAPPUDRAFT_301743 [Daphnia pulex]
4676103	gi|676442190|ref|XP_009049729.1|	1.37396e-30	hypothetical protein LOTGIDRAFT_173709 [Lottia gigantea]
4656385	gi|665805636|ref|XP_008551020.1|	1.3993e-57	PREDICTED: Niemann-Pick C1 protein isoform X3 [Microplitis demolitor]
4679161	gi|110555466|gb|ABG75717.1|	1.63108e-108	hemolectin [Callinectes sapidus]
4633759	gi|21428670|gb|AAM49995.1|	2.03638e-26	RE27784p [Drosophila melanogaster]
4630105	gi|533176937|ref|XP_005401781.1|	2.21385e-21	PREDICTED: sodium- and chloride-dependent glycine transporter 2 isoform X1 [Chinchilla lanigera]
4665417	gi|166014131|gb|ABY77968.1|	3.65853e-62	tetraspanin-like protein CD9 [Fenneropenaeus chinensis]
4671503	gi|260813850|ref|XP_002601629.1|	5.342e-80	hypothetical protein BRAFLDRAFT_85797 [Branchiostoma floridae]
4626877	gi|429535130|emb|CCE73651.2|	5.65099e-21	chymotrypsin-like proteinase [Scylla paramamosain]
4639185	gi|390351605|ref|XP_003727690.1|	6.59864e-11	PREDICTED: RNA-directed DNA polymerase from mobile element jockey-like [Strongylocentrotus purpuratus]
4656043	gi|307175374|gb|EFN65393.1|	8.36444e-55	Sodium- and chloride-dependent glycine transporter 2 [Camponotus floridanus]
