# Supplementary material for: Biodiversity in marine invertebrate responses to acute warming revealed by a comparative multi‐omics approach
Source: Glob Chang Biol. 2016 Jun 17;23(1):318–30. doi: 10.1111/gcb.13357 (PMC6849730; doi:10.1111/gcb.13357)
Supplement: Supplementary file 4 — Table S4. Transcripts up‐regulated in Cucumaria georgiana in response to acute thermal stress. [file GCB-23-318-s004.pdf]

Supplementary Table S4: Transcripts up-regulated in *C. georgiana* in response to acute thermal stress
Transcripts with annotations below 10-10 or no annotation not showr

contig	accession	evalue	description
7091818	gi|134154087|gb|ABO64423.1|	1.2254e-51	cytochrome oxidase subunit II [Drosophila polychaeta]
7077600	gi|225690875|gb|ACO06120.1|	1.31687e-35	cytochrome b (mitochondrion) [Cephalonomia gallicola]
7092320	gi|288904167|ref|YP_003434434.1|	2.20719e-48	NADH dehydrogenase subunit 5 (mitochondrion) [Chaetoderma nitidulum]
7104742	gi|308745877|ref|YP_003934366.1|	6.27042e-36	cytochrome c oxidase subunit III (mitochondrion) [Ceraesignum maximum]
7139327	gi|359422034|ref|YP_004935449.1|	3.79073e-119	cytochrome c oxidase subunit I (mitochondrion) [Bathyteuthis abyssicola
7140781	gi|390343190|ref|XP_783312.3|	4.11794e-63	PREDICTED: protein yippee-like 2-like [Strongylocentrotus purpuratus]
7099238	gi|390344140|ref|XP_784504.3|	6.64594e-87	PREDICTED: UDP-N-acetylglucosamine-peptide N-acetylglucosaminyltransferase 110 kDa subunit-like [Strongylocentrotus purpuratus]
7122369	gi|390344149|ref|XP_003726054.1|	1.05985e-43	PREDICTED: cholesterol 24-hydroxylase-like isoform 1 [Strongylocentrotus purpuratus
7126995	gi|405952839|gb|EKC20601.1|	1.56901e-22	Zinc finger SWIM domain-containing protein 2 [Crassostrea gigas]
7090962	gi|42559558|sp|O97192.1|TPM_HELAS	2.56782e-41	RecName: Full=Tropomyosin; AltName: Allergen=Hel as 1 [Helix aspersa
7129591	gi|524864520|ref|XP_005089095.1|	6.92997e-40	PREDICTED: tropomyosin-2-like isoform X3 [Aplysia californica]
7102378	gi|524916452|ref|XP_005113003.1|	2.86283e-27	PREDICTED: elongation factor 1-alpha-like isoform X1 [Aplysia californica]
7152303	gi|528947524|ref|XP_005205837.1|	4.61986e-12	PREDICTED: GTPase, IMAP family member 7 isoform X1 [Bos taurus
7122771	gi|637266065|ref|XP_008102340.1|	6.35691e-20	PREDICTED: actin, cytoplasmic 2 [Anolis carolinensis]
7144523	gi|669026851|ref|YP_009048973.1|	2.75027e-61	NADH dehydrogenase subunit 4 (mitochondrion) [Pomacea canaliculata]
