# Supplementary material for: Biodiversity in marine invertebrate responses to acute warming revealed by a comparative multi‐omics approach
Source: Glob Chang Biol. 2016 Jun 17;23(1):318–30. doi: 10.1111/gcb.13357 (PMC6849730; doi:10.1111/gcb.13357)
Supplement: Supplementary file 5 — Table S5. Transcripts up‐regulated in Aequiyoldia eightsii in response to acute thermal stress. [file GCB-23-318-s005.pdf]

**Supplementary Table S5: Transcripts up-regulated in *A. eightsii* in response to acute thermal stress**
Transcripts with annotations below 10-10 or no annotation not shown

| contig | accession | evalue | description |
|---|---|---|---|
| 3229352 | gi\|676480191\|ref\|XP_009061984.1\| | 0 | hypothetical protein LOTGIDRAFT_107431 [Lottia gigantea] |
| 3229638 | gi\|676449138\|ref\|XP_009051958.1\| | 0 | hypothetical protein LOTGIDRAFT_231552 [Lottia gigantea] |
| 3193627 | gi\|405973606\|gb\|EKC38308.1\| | 1.03E-63 | Early growth response protein 3 [Crassostrea gigas] |
| 3228146 | gi\|405973606\|gb\|EKC38308.1\| | 1.03E-38 | Early growth response protein 3 [Crassostrea gigas] |
| 3202936 | gi\|425703136\|gb\|AFX93746.1\| | 1.05E-19 | ferritin 4 [Ruditapes decussatus] |
| 3207387 | gi\|405952714\|gb\|EKC20493.1\| | 1.07E-18 | Neuropeptide FF receptor 2 [Crassostrea gigas] |
| 3227674 | gi\|291242079\|ref\|XP_002740936.1\| | 1.16E-44 | PREDICTED: protein mab-21-like 3-like [Saccoglossus kowalevskii] |
| 3229656 | gi\|676469281\|ref\|XP_009058481.1\| | 1.23E-68 | hypothetical protein LOTGIDRAFT_163713 [Lottia gigantea] |
| 3225406 | gi\|524914545\|ref\|XP_005112086.1\| | 1.31E-24 | PREDICTED: potassium channel regulatory protein-like [Aplysia californica] |
| 3228566 | gi\|405969876\|gb\|EKC34821.1\| | 1.37E-108 | hypothetical protein CGI_10022523 [Crassostrea gigas] |
| 3193775 | gi\|325504493\|emb\|CBX41746.1\| | 1.89E-25 | putative C1q domain containing protein MgC1q97 [Mytilus galloprovincialis] |
| 3213727 | gi\|405958346\|gb\|EKC24482.1\| | 2.06E-25 | Collagen alpha-6(VI) chain [Crassostrea gigas] |
| 3227926 | gi\|524907917\|ref\|XP_005109069.1\| | 2.30E-11 | PREDICTED: uncharacterized protein LOC101853971 [Aplysia californica] |
| 3224270 | gi\|676452023\|ref\|XP_009052898.1\| | 2.46E-131 | hypothetical protein LOTGIDRAFT_159961 [Lottia gigantea] |
| 3209431 | gi\|460002040\|gb\|AGH06131.1\| | 2.50E-29 | galectin [Tegillarca granosa] |
| 3221226 | gi\|676485147\|ref\|XP_009063567.1\| | 2.56E-107 | hypothetical protein LOTGIDRAFT_130278, partial [Lottia gigantea] |
| 3219866 | gi\|405964142\|gb\|EKC29659.1\| | 2.58E-29 | Agrin [Crassostrea gigas] |
| 3215001 | gi\|405957479\|gb\|EKC23686.1\| | 2.99E-38 | Tolloid-like protein 2 [Crassostrea gigas] |
| 3223286 | gi\|676437499\|ref\|XP_009048224.1\| | 3.15E-32 | hypothetical protein LOTGIDRAFT_172798 [Lottia gigantea] |
| 3208366 | gi\|260830611\|ref\|XP_002610254.1\| | 3.54E-25 | hypothetical protein BRAFLDRAFT_126823 [Branchiostoma floridae] |
| 3197966 | gi\|676436230\|ref\|XP_009047814.1\| | 3.57E-39 | hypothetical protein LOTGIDRAFT_200320 [Lottia gigantea] |
| 3216853 | gi\|405960265\|gb\|EKC26205.1\| | 3.92E-31 | Interferon regulatory factor 8 [Crassostrea gigas] |
| 3228386 | gi\|405977942\|gb\|EKC42366.1\| | 4.49E-125 | Penicillin-binding protein 4 [Crassostrea gigas] |
| 3220794 | gi\|443720568\|gb\|ELU10262.1\| | 5.28E-81 | hypothetical protein CAPTEDRAFT_156752 [Capitella teleta] |
| 3226352 | gi\|291242079\|ref\|XP_002740936.1\| | 5.42E-56 | PREDICTED: protein mab-21-like 3-like [Saccoglossus kowalevskii] |
| 3212753 | gi\|524907562\|ref\|XP_005108897.1\| | 5.88E-62 | PREDICTED: AN1-type zinc finger protein 2B-like [Aplysia californica] |
| 3220070 | gi\|405956943\|gb\|EKC23185.1\| | 5.93E-74 | hypothetical protein CGI_10017349 [Crassostrea gigas] |
| 3201220 | gi\|405976178\|gb\|EKC40694.1\| | 6.34E-56 | DBH-like monooxygenase protein 1 [Crassostrea gigas] |
| 3228170 | gi\|405970925\|gb\|EKC35788.1\| | 6.41E-89 | Melatonin receptor type 1B [Crassostrea gigas] |
| 3228792 | gi\|443696912\|gb\|ELT97520.1\| | 6.59E-13 | hypothetical protein CAPTEDRAFT_213147 [Capitella teleta] |
| 3221418 | gi\|676440832\|ref\|XP_009049302.1\| | 6.96E-36 | hypothetical protein LOTGIDRAFT_203644 [Lottia gigantea] |
| 3227234 | gi\|676471285\|ref\|XP_009059127.1\| | 7.71E-59 | hypothetical protein LOTGIDRAFT_233878 [Lottia gigantea] |
| 3229376 | gi\|554802113\|gb\|AGZ03662.1\| | 8.04E-61 | inhibitor of nuclear factor-kappaB protein [Haliotis rufescens] |
| 3229210 | gi\|405970232\|gb\|EKC35160.1\| | 8.57E-41 | Tumor necrosis factor ligand superfamily member 10 [Crassostrea gigas] |
| 3219916 | gi\|405970348\|gb\|EKC35262.1\| | 8.60E-17 | Caprin-2 [Crassostrea gigas] |
| 3197128 | gi\|390336919\|ref\|XP_785416.3\| | 9.31E-16 | PREDICTED: uncharacterized protein LOC580251 [Strongylocentrotus purpuratus] |
| 3185561 | gi\|405976185\|gb\|EKC40701.1\| | 9.41E-26 | Deleted in malignant brain tumors 1 protein [Crassostrea gigas] |
| 3228728 | gi\|405945264\|gb\|EKC17247.1\| | 9.62E-57 | Kelch-like protein 24 [Crassostrea gigas] |
| 3201776 | gi\|585697861\|ref\|XP_006822461.1\| | 9.89E-12 | PREDICTED: neurogenic locus notch homolog protein 1-like [Saccoglossus kowalevskii] |
