# Supplementary material for: Biodiversity in marine invertebrate responses to acute warming revealed by a comparative multi‐omics approach
Source: Glob Chang Biol. 2016 Jun 17;23(1):318–30. doi: 10.1111/gcb.13357 (PMC6849730; doi:10.1111/gcb.13357)
Supplement: Supplementary file 6 — Table S6. Transcripts up‐regulated in Liothyrella uva in response to acute thermal stress. [file GCB-23-318-s006.pdf]

Supplementary Table S6: Transcripts up-regulated in *L. uva* in response to acute thermal stress
Transcripts with annotations below 10-10 or no annotation not showr

contig	accession	evalue	description
6046018	gi|675875616|ref|XP_009023368.1|	3.90582e-65	hypothetical protein HELRODRAFT_101612 [Helobdella robusta]
6077945	gi|524867963|ref|XP_005090788.1|	0	PREDICTED: E3 ubiquitin-protein ligase HECTD1-like [Aplysia californica]
6075303	gi|443684487|gb|ELT88415.1|	0	hypothetical protein CAPTEDRAFT_172215 [Capitella teleta]
6057954	gi|443730298|gb|ELU15864.1|	1.00968e-50	hypothetical protein CAPTEDRAFT_201069 [Capitella teleta]
6038540	gi|585706264|ref|XP_006823786.1|	1.00986e-64	PREDICTED: mitogen-activated protein kinase kinase kinase 13-A-like [Saccoglossus kowalevskii
6078451	gi|676459477|ref|XP_009055313.1|	1.01915e-22	hypothetical protein LOTGIDRAFT_104988 [Lottia gigantea]
6046528	gi|260781198|ref|XP_002585708.1|	1.03245e-12	hypothetical protein BRAFLDRAFT_257729 [Branchiostoma floridae]
6070485	gi|321458955|gb|EFX70014.1|	1.03957e-44	hypothetical protein DAPPUDRAFT_328543 [Daphnia pulex]
6051080	gi|241813188|ref|XP_002414634.1|	1.07693e-27	serine/threonine protein kinase TAO1, putative [Ixodes scapularis
6056712	gi|405957817|gb|EKC23997.1|	1.10287e-59	hypothetical protein CGI_10014240, partial [Crassostrea gigas]
6079479	gi|291222691|ref|XP_002731347.1|	1.1081e-39	PREDICTED: uncharacterized protein LOC100377009 [Saccoglossus kowalevskii]
6077063	gi|524892837|ref|XP_005102943.1|	1.11672e-21	PREDICTED: receptor-type tyrosine-protein phosphatase T-like [Aplysia californica
6057690	gi|443709725|gb|ELU04274.1|	1.12444e-54	hypothetical protein CAPTEDRAFT_105698 [Capitella teleta]
6073749	gi|260834587|ref|XP_002612291.1|	1.1262e-46	hypothetical protein BRAFLDRAFT_122528 [Branchiostoma floridae]
6052830	gi|524908162|ref|XP_005109187.1|	1.15459e-78	PREDICTED: protein gustavus-like [Aplysia californica]
6045142	gi|530583059|ref|XP_005285366.1|	1.19694e-79	PREDICTED: E3 ubiquitin-protein ligase HECTD1 isoform X1 [Chrysemys picta bellii]
6051922	gi|390334808|ref|XP_787986.3|	1.21225e-29	PREDICTED: protein dispatched homolog 1-like [Strongylocentrotus purpuratus]
6058666	gi|597744941|ref|XP_007234516.1|	1.23062e-67	PREDICTED: helicase ARIP4-like [Astyanax mexicanus
6064610	gi|524883127|ref|XP_005098197.1|	1.23339e-41	PREDICTED: uncharacterized protein LOC101848352 [Aplysia californica]
6078499	gi|157108670|ref|XP_001650338.1|	1.26137e-20	toll [Aedes aegypti]
6042074	gi|642940388|ref|XP_008200541.1|	1.265e-14	PREDICTED: chitin deacetylase 5 isoform X2 [Tribolium castaneum]
6074349	gi|676456145|ref|XP_009054232.1|	1.2931e-117	hypothetical protein LOTGIDRAFT_160793 [Lottia gigantea]
6058202	gi|676451606|ref|XP_009052761.1|	1.30488e-13	hypothetical protein LOTGIDRAFT_159800 [Lottia gigantea]
6064440	gi|524866574|ref|XP_005090108.1|	1.31277e-27	PREDICTED: ubiquitin-protein ligase E3C-like [Aplysia californica]
6062904	gi|676477317|ref|XP_009061075.1|	1.32276e-50	hypothetical protein LOTGIDRAFT_165810 [Lottia gigantea]
6053084	gi|405975554|gb|EKC40113.1|	1.32716e-58	Polypeptide N-acetylgalactosaminyltransferase 1 [Crassostrea gigas
6040824	gi|260791285|ref|XP_002590670.1|	1.35576e-32	hypothetical protein BRAFLDRAFT_125550 [Branchiostoma floridae]
6071415	gi|443734989|gb|ELU18844.1|	1.36225e-18	hypothetical protein CAPTEDRAFT_226756 [Capitella teleta]
6046826	gi|405969748|gb|EKC34701.1|	1.36882e-36	MAGUK p55 subfamily member 5 [Crassostrea gigas]
6061268	gi|524867963|ref|XP_005090788.1|	1.38658e-22	PREDICTED: E3 ubiquitin-protein ligase HECTD1-like [Aplysia californica]
6076007	gi|389568425|gb|AFK84936.1|	1.41501e-166	heat shock protein 70 [Lineidae sp. TWL-2008]
6058862	gi|443683057|gb|ELT87432.1|	1.4914e-20	hypothetical protein CAPTEDRAFT_190001 [Capitella teleta]
6045814	gi|602668029|ref|XP_007439569.1|	1.61489e-39	PREDICTED: transmembrane protein 144 isoform X1 [Python bivittatus]
6071501	gi|585688595|ref|XP_006820707.1|	1.64037e-104	PREDICTED: uncharacterized protein LOC100366770 [Saccoglossus kowalevskii]
6057756	gi|443722201|gb|ELU11164.1|	1.6478e-45	hypothetical protein CAPTEDRAFT_119245 [Capitella teleta]
6040578	gi|70663484|emb|CAJ15141.1|	1.69921e-59	PL10b protein [Platynereis dumerilii]
6069195	gi|676776898|gb|KFP04398.1|	1.72957e-14	Ankyrin repeat and SOCS box protein 8, partial [Calypte anna
6061194	gi|676492991|ref|XP_009066101.1|	1.73381e-57	hypothetical protein LOTGIDRAFT_133948, partial [Lottia gigantea]
6071125	gi|4160308|emb|CAA12274.1|	1.80237e-15	wee1-like kinase [Platynereis dumerilii]
6078085	gi|676495320|ref|XP_009066852.1|	1.81005e-151	hypothetical protein LOTGIDRAFT_91560, partial [Lottia gigantea]
6055748	gi|443696719|gb|ELT97356.1|	1.82441e-35	hypothetical protein CAPTEDRAFT_185181 [Capitella teleta]
6057384	gi|405961790|gb|EKC27535.1|	1.8809e-78	Tyrosine-protein phosphatase non-receptor type 9 [Crassostrea gigas
6066847	gi|524879637|ref|XP_005096492.1|	1.89561e-46	PREDICTED: epidermal growth factor receptor-like, partial [Aplysia californica
6039856	gi|405961156|gb|EKC27001.1|	1.90598e-26	Myosin-XVI [Crassostrea gigas]
6061224	gi|443697824|gb|ELT98122.1|	1.91695e-56	hypothetical protein CAPTEDRAFT_225958 [Capitella teleta]
6054374	gi|443721639|gb|ELU10878.1|	1.92028e-23	hypothetical protein CAPTEDRAFT_223355 [Capitella teleta]
6039438	gi|676474303|ref|XP_009060103.1|	1.92627e-15	hypothetical protein LOTGIDRAFT_154137 [Lottia gigantea]
6042736	gi|557007101|ref|XP_006004935.1|	1.94654e-15	PREDICTED: E3 UFM1-protein ligase 1 [Latimeria chalumnae]
6072489	gi|291240099|ref|XP_002739960.1|	1.99186e-63	PREDICTED: ATP-binding cassette sub-family A member 3-like [Saccoglossus kowalevskii
6044106	gi|524892005|ref|XP_005102534.1|	2.00752e-84	PREDICTED: E3 ubiquitin-protein ligase MIB1-like [Aplysia californica]
6063062	gi|657781905|ref|XP_008318572.1|	2.01349e-14	PREDICTED: leucine-rich repeats and immunoglobulin-like domains protein 1 [Cynoglossus semilaevis]

6042078	gi|556966325|ref|XP_005992564.1|	2.08512e-17	PREDICTED: dual specificity protein phosphatase 1-B-like [Latimeria chalumnae]
6056066	gi|405965787|gb|EKC31141.1|	2.11109e-39	hypothetical protein CGI_10028774 [Crassostrea gigas]
6049042	gi|443693755|gb|ELT95042.1|	2.11698e-30	hypothetical protein CAPTEDRAFT_126822, partial [Capitella teleta]
6045988	gi|78706948|ref|NP_001027279.1|	2.11891e-23	Cadherin-N, isoform J [Drosophila melanogaster]
6075377	gi|390370892|ref|XP_798214.3|	2.14763e-12	PREDICTED: uncharacterized protein LOC593651, partial [Strongylocentrotus purpuratus]
6062488	gi|260790829|ref|XP_002590443.1|	2.17335e-82	hypothetical protein BRAFLDRAFT_62750 [Branchiostoma floridae]
6068843	gi|390361028|ref|XP_003729826.1|	2.18749e-38	PREDICTED: uncharacterized protein LOC100888806 [Strongylocentrotus purpuratus]
6058612	gi|676428711|ref|XP_009045364.1|	2.20269e-16	hypothetical protein LOTGIDRAFT_156479 [Lottia gigantea]
6067791	gi|197091707|gb|ACH42086.1|	2.21371e-101	predicted phosphoinositide 4-kinase beta [Crassostrea gigas]
6076379	gi|677983493|ref|XP_009074024.1|	2.23254e-13	PREDICTED: cytochrome P450 2D14 [Acanthisitta chloris]
6044120	gi|55728292|emb|CAH90891.1|	2.24026e-51	hypothetical protein [Pongo abelii]
6071765	gi|676439421|ref|XP_009048847.1|	2.24354e-16	hypothetical protein LOTGIDRAFT_158021 [Lottia gigantea]
6049868	gi|543372262|ref|XP_005529352.1|	2.26085e-51	PREDICTED: acidic mammalian chitinase-like [Pseudopodoces humilis]
6050272	gi|632951519|ref|XP_007891337.1|	2.28302e-30	PREDICTED: G-protein coupled receptor 98 [Callorhinchus milii]
6045138	gi|390366572|ref|XP_780926.3|	2.3687e-27	PREDICTED: deleted in malignant brain tumors 1 protein-like [Strongylocentrotus purpuratus]
6077577	gi|585646687|ref|XP_006811776.1|	2.45697e-107	PREDICTED: neurogenic locus notch homolog protein 1-like [Saccoglossus kowalevskii
6077015	gi|405977508|gb|EKC41951.1|	2.46724e-21	Protein slit [Crassostrea gigas]
6047656	gi|260823384|ref|XP_002604163.1|	2.49218e-73	hypothetical protein BRAFLDRAFT_119756 [Branchiostoma floridae]
6065217	gi|291235010|ref|XP_002737439.1|	2.54201e-106	PREDICTED: cullin-5-like [Saccoglossus kowalevskii]
6073053	gi|405965659|gb|EKC31021.1|	2.56277e-77	Pleckstrin-like protein domain-containing family A member 6 [Crassostrea gigas
6049860	gi|466025882|ref|XP_004273203.1|	2.60481e-23	PREDICTED: cadherin-23 isoform 1 [Orcinus orca]
6070757	gi|260813989|ref|XP_002601698.1|	2.63775e-104	hypothetical protein BRAFLDRAFT_94578 [Branchiostoma floridae]
6061770	gi|676489406|ref|XP_009064924.1|	2.65163e-77	hypothetical protein LOTGIDRAFT_132462 [Lottia gigantea]
6041756	gi|196012188|ref|XP_002115957.1|	2.89016e-51	hypothetical protein TRIADDRAFT_29967 [Trichoplax adhaerens]
6041222	gi|607359654|gb|EZA54007.1|	2.90438e-43	Signal recognition particle receptor subunit alpha-like protein [Cerapachys biroi]
6074729	gi|405971677|gb|EKC36500.1|	2.93234e-88	Myosin-IXa [Crassostrea gigas]
6069567	gi|405952069|gb|EKC19921.1|	3.00951e-30	Leucine zipper putative tumor suppressor 2-like protein [Crassostrea gigas
6077359	gi|662183513|ref|XP_008487855.1|	3.15083e-27	PREDICTED: uncharacterized protein LOC103524604 [Diaphorina citri]
6058728	gi|578399327|gb|AHI16242.1|	3.15862e-39	spondin [Platynereis dumerilii]
6039926	gi|675869428|ref|XP_009020274.1|	3.22856e-50	hypothetical protein HELRODRAFT_81481, partial [Helobdella robusta]
6050510	gi|443700702|gb|ELT99546.1|	3.28903e-37	hypothetical protein CAPTEDRAFT_222769 [Capitella teleta]
6043490	gi|156351203|ref|XP_001622406.1|	3.38085e-31	hypothetical protein NEMVEDRAFT_v1g141380 [Nematostella vectensis]
6074907	gi|443724638|gb|ELU12542.1|	3.38724e-116	hypothetical protein CAPTEDRAFT_156898 [Capitella teleta]
6069435	gi|585660883|ref|XP_006816466.1|	3.46852e-95	PREDICTED: unconventional myosin-XVI-like, partial [Saccoglossus kowalevskii
6075821	gi|557321011|ref|XP_006033630.1|	3.48769e-61	PREDICTED: collagen alpha-5(IV) chain [Alligator sinensis]
6074949	gi|432889245|ref|XP_004075183.1|	3.64597e-118	PREDICTED: multiple epidermal growth factor-like domains protein 10-like [Oryzias latipes
6068105	gi|641483972|gb|AIA66467.1|	3.95319e-13	toll2 [Hyriopsis cumingii]
6056122	gi|405969351|gb|EKC34327.1|	3.99985e-22	Fidgetin-like protein 1 [Crassostrea gigas]
6077267	gi|390369149|ref|XP_791996.3|	4.06067e-27	PREDICTED: toll-like receptor 3-like, partial [Strongylocentrotus purpuratus]
6051372	gi|405966360|gb|EKC31655.1|	4.24112e-80	Chloride channel protein 2 [Crassostrea gigas]
6048168	gi|625257020|ref|XP_007619877.1|	4.26078e-12	PREDICTED: chondroadherin-like protein isoform X2 [Cricetulus griseus]
6058556	gi|148227411|ref|NP_001080141.1|	4.28549e-97	glycine dehydrogenase (decarboxylating) [Xenopus laevis]
6042292	gi|657583425|ref|XP_008295821.1|	4.34774e-15	PREDICTED: sperm flagellar protein 1 [Stegastes partitus]
6043304	gi|443732187|gb|ELU17015.1|	4.43606e-15	hypothetical protein CAPTEDRAFT_175610 [Capitella teleta]
6068851	gi|676472743|ref|XP_009059589.1|	4.5401e-52	hypothetical protein LOTGIDRAFT_234185 [Lottia gigantea]
6055454	gi|524893527|ref|XP_005103276.1|	4.55473e-55	PREDICTED: serine/threonine-protein kinase MRCK alpha-like isoform X7 [Aplysia californica]
6044322	gi|524899942|ref|XP_005106397.1|	4.56663e-12	PREDICTED: uncharacterized protein LOC101845642 [Aplysia californica]
6058606	gi|443699253|gb|ELT98834.1|	4.86176e-48	hypothetical protein CAPTEDRAFT_226944 [Capitella teleta]
6071097	gi|637376116|ref|XP_008122950.1|	4.86299e-45	PREDICTED: B-cell lymphoma 3 protein-like [Anolis carolinensis]
6064038	gi|676488256|ref|XP_009064558.1|	4.89434e-43	hypothetical protein LOTGIDRAFT_236112 [Lottia gigantea]
6046350	gi|607366279|gb|EZA60450.1|	4.98692e-28	Tyrosine-protein kinase Src42A [Cerapachys biroi]
6073329	gi|443691684|gb|ELT93466.1|	5.05735e-12	hypothetical protein CAPTEDRAFT_198011 [Capitella teleta]
6049396	gi|675374960|gb|KFM67862.1|	5.11378e-48	Inhibitor of nuclear factor kappa-B kinase subunit beta, partial [Stegodyphus mimosarum
6068251	gi|260782360|ref|XP_002586256.1|	5.18401e-45	hypothetical protein BRAFLDRAFT_109340 [Branchiostoma floridae]
6061856	gi|676449926|ref|XP_009052212.1|	5.31626e-86	hypothetical protein LOTGIDRAFT_143498 [Lottia gigantea]

6055988	gi|307209790|gb|EFN86595.1|	5.3189e-75	Uncharacterized protein KIAA1109 [Harpegnathos saltator
6057388	gi|504246634|ref|WP_014433736.1|	5.36245e-33	alpha-amylase [Caldilinea aerophila]
6056276	gi|405960504|gb|EKC26425.1|	5.42842e-51	Serine/threonine-protein kinase SIK2 [Crassostrea gigas
6061688	gi|405963486|gb|EKC29055.1|	5.53809e-35	Inter-alpha-trypsin inhibitor heavy chain H3 [Crassostrea gigas]
6065419	gi|676448722|ref|XP_009051824.1|	5.55501e-85	hypothetical protein LOTGIDRAFT_54044, partial [Lottia gigantea]
6057990	gi|528481343|ref|XP_005172123.1|	5.57901e-25	PREDICTED: uncharacterized protein LOC101882198 [Danio rerio]
6045162	gi|524870892|ref|XP_005092226.1|	5.83431e-26	PREDICTED: uncharacterized protein LOC101846194 isoform X1 [Aplysia californica]
6071817	gi|676454892|ref|XP_009053828.1|	6.05862e-78	hypothetical protein LOTGIDRAFT_116962 [Lottia gigantea]
6074577	gi|630025408|ref|XP_007835454.1|	6.12458e-14	hypothetical protein PFICI_08682 [Pestalotiopsis fici W106-1]
6038682	gi|597871181|gb|EYC20560.1|	6.1694e-38	hypothetical protein Y032_0021g277 [Ancylostoma ceylanicum]
6068351	gi|585715375|ref|XP_006825324.1|	6.17855e-38	PREDICTED: VWFA and cache domain-containing protein 1-like [Saccoglossus kowalevskii
6050212	gi|705689746|ref|XP_010121809.1|	6.37443e-57	PREDICTED: MAGUK p55 subfamily member 5, partial [Chlamydotis macqueenii]
6045684	gi|443720895|gb|ELU10447.1|	6.39469e-12	hypothetical protein CAPTEDRAFT_226528 [Capitella teleta]
6078893	gi|405967696|gb|EKC32830.1|	6.48238e-45	Tetratricopeptide repeat protein 28 [Crassostrea gigas
6074879	gi|405969876|gb|EKC34821.1|	6.70928e-48	hypothetical protein CGI_10022523 [Crassostrea gigas]
6070929	gi|156374121|ref|XP_001629657.1|	6.95713e-20	predicted protein [Nematostella vectensis]
6056688	gi|405971703|gb|EKC36526.1|	7.00475e-14	Nuclear receptor ROR-beta [Crassostrea gigas
6063988	gi|405976336|gb|EKC40848.1|	7.10081e-111	hypothetical protein CGI_10026535 [Crassostrea gigas]
6072849	gi|557277978|ref|XP_006022065.1|	7.15773e-24	PREDICTED: Hermansky-Pudlak syndrome 5 protein isoform X2 [Alligator sinensis]
6056662	gi|646702053|gb|KDR11489.1|	7.18185e-43	Methionine aminopeptidase 1D, mitochondrial [Zootermopsis nevadensis
6077851	gi|524891035|ref|XP_005102056.1|	7.53619e-22	PREDICTED: tyrosine-protein phosphatase non-receptor type 21-like [Aplysia californica
6045034	gi|572259897|ref|XP_006608279.1|	7.86704e-31	PREDICTED: disintegrin and metalloproteinase domain-containing protein 10-like [Apis dorsata
6070923	gi|675379273|gb|KFM72175.1|	7.90479e-40	putative ribonuclease, partial [Stegodyphus mimosarum]
6041092	gi|556735675|ref|XP_005963445.1|	7.90938e-41	PREDICTED: cytosolic phospholipase A2 [Pantholops hodgsonii]
6054552	gi|524868195|ref|XP_005090902.1|	7.91259e-25	PREDICTED: yorkie homolog isoform X1 [Aplysia californica
6057996	gi|646695040|gb|KDR08214.1|	8.1456e-101	Serine/threonine-protein kinase LATS1 [Zootermopsis nevadensis
6066311	gi|524908624|ref|XP_005109405.1|	8.14757e-103	PREDICTED: serine/threonine-protein phosphatase 4 regulatory subunit 3A-like isoform X2 [Aplysia californica
6039562	gi|405978349|gb|EKC42749.1|	8.30597e-14	Glycine dehydrogenase [decarboxylating], mitochondrial [Crassostrea gigas
6042578	gi|585647647|ref|XP_006814078.1|	8.40442e-35	PREDICTED: rho GTPase-activating protein 21-like [Saccoglossus kowalevskii]
6055070	gi|676438854|ref|XP_009048666.1|	8.40955e-30	hypothetical protein LOTGIDRAFT_157823 [Lottia gigantea]
6070871	gi|405967934|gb|EKC33048.1|	9.04424e-36	Atrial natriuretic peptide-converting enzyme [Crassostrea gigas
6052820	gi|432910540|ref|XP_004078404.1|	9.57746e-33	PREDICTED: Friend leukemia integration 1 transcription factor-like [Oryzias latipes
6055040	gi|524868989|ref|XP_005091292.1|	9.77393e-87	PREDICTED: DNA topoisomerase 1-like [Aplysia californica]
6075281	gi|524917297|ref|XP_005113408.1|	9.81898e-143	PREDICTED: mitogen-activated protein kinase kinase kinase MLT-like, partial [Aplysia californica
6039166	gi|405970510|gb|EKC35408.1|	9.87009e-20	Eukaryotic translation initiation factor 2-alpha kinase 3 [Crassostrea gigas
