# Supplementary material for: Biodiversity in marine invertebrate responses to acute warming revealed by a comparative multi‐omics approach
Source: Glob Chang Biol. 2016 Jun 17;23(1):318–30. doi: 10.1111/gcb.13357 (PMC6849730; doi:10.1111/gcb.13357)
Supplement: Supplementary file 7 — Table S7. Transcripts up‐regulated in Laternula elliptica gill in response to acute thermal stress [file GCB-23-318-s007.pdf]

Supplementary Table S7: Transcripts up-regulated in *L. elliptica* gill in response to acute thermal stress
Transcripts with annotations below 10-10 or no annotation not show

contig	accession	evalue	description
9652084	gi|676429795|ref|XP_009045717.1|	0	hypothetical protein LOTGIDRAFT_137098, partial [Lottia gigantea]
9650112	gi|468860726|gb|AGH32327.1|	0	heat shock protein 70 [Cellana toreuma
9653216	gi|405971274|gb|EKC36120.1|	0	RIC1-like protein [Crassostrea gigas]
9651816	gi|405958381|gb|EKC24514.1|	0	B-box type zinc finger protein ncl-1 [Crassostrea gigas
9644882	gi|405953136|gb|EKC20853.1|	0	Transformation/transcription domain-associated protein [Crassostrea gigas]
9648626	gi|405978038|gb|EKC42455.1|	0	Sodium/hydrogen exchanger 2 [Crassostrea gigas
9648872	gi|405956907|gb|EKC23150.1|	0	StAR-related lipid transfer protein 13 [Crassostrea gigas]
9646784	gi|405977580|gb|EKC42023.1|	0	Uridine-cytidine kinase-like 1 [Crassostrea gigas]
9653406	gi|405964979|gb|EKC30412.1|	0	Mitogen-activated protein kinase kinase kinase 9 [Crassostrea gigas
9648076	gi|676447469|ref|XP_009051417.1|	0	hypothetical protein LOTGIDRAFT_208735 [Lottia gigantea
9652946	gi|558214655|ref|XP_006134465.1|	0	PREDICTED: hephaestin-like protein 1 isoform X1 [Pelodiscus sinensis]>gi|558214667|ref|XP_006134467.1| PREDICTED: hephaestin-like protein 1 isoform X3 [Pelodiscus sinensi
9648144	gi|405966994|gb|EKC32211.1|	0	Kelch-like protein 18 [Crassostrea gigas]
9649986	gi|676478926|ref|XP_009061580.1|	0	hypothetical protein LOTGIDRAFT_127385 [Lottia gigantea]
9653142	gi|405963660|gb|EKC29216.1|	0	Nucleolar protein 4 [Crassostrea gigas]
9645734	gi|15150336|gb|AAK85400.1|AF399934_1	0	RNA helicase p47 [Spisula solidissima
9650932	gi|676429098|ref|XP_009045489.1|	0	hypothetical protein LOTGIDRAFT_212187 [Lottia gigantea
9653342	gi|676420698|ref|XP_009043703.1|	0	hypothetical protein LOTGIDRAFT_208096 [Lottia gigantea]
9648632	gi|405975644|gb|EKC40198.1|	0	ATP-binding cassette sub-family F member 3 [Crassostrea gigas
9653026	gi|405962975|gb|EKC28596.1|	0	Protein TANC2 [Crassostrea gigas]
9647852	gi|405971243|gb|EKC36089.1|	0	transport protein Sec31A [Crassostrea gigas]
9653040	gi|676461891|ref|XP_009056090.1|	0	hypothetical protein LOTGIDRAFT_232790 [Lottia gigantea
9642336	gi|676492384|ref|XP_009065899.1|	0	hypothetical protein LOTGIDRAFT_133427 [Lottia gigantea
9651348	gi|405953091|gb|EKC20815.1|	0	Integrator complex subunit 3 [Crassostrea gigas
9650344	gi|676482608|ref|XP_009062757.1|	0	hypothetical protein LOTGIDRAFT_179365 [Lottia gigantea]
9650780	gi|524891868|ref|XP_005102466.1|	0	PREDICTED: microtubule-associated protein futsch-like [Aplysia californica
9652554	gi|405962385|gb|EKC28072.1|	0	Hippocampus abundant transcript 1 protein [Crassostrea gigas
9649676	gi|524908597|ref|XP_005109392.1|	0	PREDICTED: LOW QUALITY PROTEIN: diacylglycerol kinase theta [Aplysia californica
9651322	gi|405964508|gb|EKC29984.1|	0	Serine/threonine-protein kinase tousled-like 1 [Crassostrea gigas
9653390	gi|405975449|gb|EKC40013.1|	0	Hemicentin-1 [Crassostrea gigas]
9647292	gi|443722438|gb|ELU11307.1|	0	hypothetical protein CAPTEDRAFT_179015 [Capitella teleta
9649466	gi|405971153|gb|EKC36006.1|	0	Cytoskeleton-associated protein 5 [Crassostrea gigas]
9653420	gi|675366580|gb|KFM59482.1|	0	Ankyrin repeat domain-containing protein 50, partial [Stegodyphus mimosaru
9643302	gi|676426143|ref|XP_009044529.1|	0	hypothetical protein LOTGIDRAFT_109410, partial [Lottia gigantea
9651712	gi|676467881|ref|XP_009058031.1|	0	hypothetical protein LOTGIDRAFT_182605, partial [Lottia gigantea
9650096	gi|405950200|gb|EKC18201.1|	0	Tyrosine-protein kinase Src42A [Crassostrea gigas]
9651244	gi|405956937|gb|EKC23179.1|	0	C-myc promoter-binding protein [Crassostrea gigas
9652442	gi|405968860|gb|EKC33889.1|	0	Phosphatidylinositol 3-kinase regulatory subunit alpha [Crassostrea gigas
9652316	gi|676461082|ref|XP_009055830.1|	0	hypothetical protein LOTGIDRAFT_119149, partial [Lottia gigantea
9650536	gi|676477122|ref|XP_009061010.1|	0	hypothetical protein LOTGIDRAFT_165736 [Lottia gigantea]
9648460	gi|676458565|ref|XP_009055017.1|	0	hypothetical protein LOTGIDRAFT_215642 [Lottia gigantea]
9648106	gi|405951632|gb|EKC19529.1|	0	TBC1 domain family member 15 [Crassostrea gigas
9650010	gi|405974168|gb|EKC38836.1|	0	Titin [Crassostrea gigas]
9653332	gi|405953139|gb|EKC20855.1|	0	Double-stranded RNA-specific adenosine deaminase [Crassostrea gigas]
9644300	gi|405970480|gb|EKC35379.1|	0	Glycogen synthase kinase-3 beta [Crassostrea gigas]
9648170	gi|524868922|ref|XP_005091259.1|	0	PREDICTED: AP-1 complex subunit gamma-1-like isoform X3 [Aplysia californica
9646938	gi|405971891|gb|EKC36694.1|	0	Protein ariadne-2 [Crassostrea gigas]
9653196	gi|405953550|gb|EKC21191.1|	0	NADPH--cytochrome P450 reductase [Crassostrea gigas]
9652262	gi|405974123|gb|EKC38791.1|	0	Exportin-6 [Crassostrea gigas]
9652458	gi|405973316|gb|EKC38036.1|	0	Lysine-specific demethylase 5A [Crassostrea gigas]
9645538	gi|443692870|gb|ELT94375.1|	0	hypothetical protein CAPTEDRAFT_102987 [Capitella teleta
9650482	gi|405976544|gb|EKC41046.1|	0	LIM domain kinase 2 [Crassostrea gigas]
9651264	gi|405972811|gb|EKC37559.1|	0	Condensin complex subunit 1 [Crassostrea gigas
9651826	gi|405968127|gb|EKC33227.1|	0	Neurofibromin [Crassostrea gigas]
9653560	gi|405950928|gb|EKC18883.1|	0	HEAT repeat-containing protein 5B [Crassostrea gigas]
9650942	gi|676434671|ref|XP_009047303.1|	0	hypothetical protein LOTGIDRAFT_212681 [Lottia gigantea]
9650530	gi|405968683|gb|EKC33730.1|	0	DNA replication licensing factor MCM3 [Crassostrea gigas]
9648570	gi|676476730|ref|XP_009060887.1|	0	hypothetical protein LOTGIDRAFT_179097 [Lottia gigantea
9653350	gi|405965623|gb|EKC30985.1|	0	Serine/threonine-protein kinase N2 [Crassostrea gigas]
9651048	gi|405976026|gb|EKC40550.1|	0	Serine/threonine-protein kinase/endoribonuclease ire-1 [Crassostrea gigas
9647868	gi|676489369|ref|XP_009064912.1|	0	hypothetical protein LOTGIDRAFT_132259, partial [Lottia gigantea]
9650444	gi|524895996|ref|XP_005104477.1|	0	PREDICTED: neuralized-like protein 4-like [Aplysia californica
9651614	gi|676492026|ref|XP_009065788.1|	0	hypothetical protein LOTGIDRAFT_169225 [Lottia gigantea
9650262	gi|676430885|ref|XP_009046073.1|	0	hypothetical protein LOTGIDRAFT_137613 [Lottia gigantea
9650720	gi|524865526|ref|XP_005089592.1|	0	PREDICTED: dedicator of cytokinesis protein 7-like isoform X4 [Aplysia californica
9650926	gi|676453053|ref|XP_009053231.1|	0	hypothetical protein LOTGIDRAFT_116192 [Lottia gigantea
9650194	gi|405958520|gb|EKC24642.1|	0	Putative protein tag-53 [Crassostrea gigas]

9649138	gi|676448457|ref|XP_009051738.1|	0	hypothetical protein LOTGIDRAFT_153005 [Lottia gigantea]
9648074	gi|405967551|gb|EKC32699.1|	0	Double-stranded RNA-specific editase 1 [Crassostrea gigas]
9653436	gi|405974491|gb|EKC39131.1|	0	hypothetical protein CGI_10011150 [Crassostrea gigas]
9650148	gi|405971529|gb|EKC36364.1|	0	NMDA receptor-regulated protein 1 [Crassostrea gigas]
9653372	gi|405967141|gb|EKC32341.1|	0	Ubiquitin carboxyl-terminal hydrolase 32 [Crassostrea gigas
9649706	gi|676493744|ref|XP_009066337.1|	0	hypothetical protein LOTGIDRAFT_169795 [Lottia gigantea]
9652890	gi|405973705|gb|EKC38401.1|	0	hypothetical protein CGI_10002987 [Crassostrea gigas]
9649450	gi|405959110|gb|EKC25175.1|	0	MBT domain-containing protein 1 [Crassostrea gigas
9645368	gi|405959793|gb|EKC25785.1|	0	ATP-dependent RNA helicase A-like protein [Crassostrea gigas
9652588	gi|405963040|gb|EKC28649.1|	0	hypothetical protein CGI_10025169 [Crassostrea gigas]
9650048	gi|676427450|ref|XP_009044951.1|	0	hypothetical protein LOTGIDRAFT_136271, partial [Lottia gigantea]
9651912	gi|676427732|ref|XP_009045043.1|	0	hypothetical protein LOTGIDRAFT_237433 [Lottia gigantea
9652844	gi|405954517|gb|EKC21934.1|	0	Brefeldin A-inhibited guanine nucleotide-exchange protein 1 [Crassostrea gigas
9652234	gi|405975064|gb|EKC39660.1|	0	Pleckstrin-like protein domain-containing family G member 5 [Crassostrea gigas
9648010	gi|405969005|gb|EKC34020.1|	0	Tyrosine 3-monooxygenase [Crassostrea gigas]
9653548	gi|405954181|gb|EKC21693.1|	0	hypothetical protein CGI_10003467 [Crassostrea gigas]
9637768	gi|676463849|ref|XP_009056733.1|	0	hypothetical protein LOTGIDRAFT_178640 [Lottia gigantea]
9651938	gi|405977754|gb|EKC42188.1|	0	Cullin-1 [Crassostrea gigas]
9635270	gi|324029111|gb|ADY16711.1|	0	arrestin [Argopecten irradians]
9652818	gi|26000552|gb|AAN75454.1|	0	Na-dependent Cl/HCO3 exchanger [Doryteuthis pealeii
9642646	gi|676490577|ref|XP_009065315.1|	0	hypothetical protein LOTGIDRAFT_211133 [Lottia gigantea]
9639408	gi|524902720|ref|XP_005107588.1|	0	PREDICTED: protein argonaute-2-like isoform X3 [Aplysia californica
9644632	gi|405975835|gb|EKC40377.1|	0	NAD(P) transhydrogenase, mitochondrial [Crassostrea gigas]
9653510	gi|524912739|ref|XP_005111210.1|	0	PREDICTED: sterol regulatory element-binding protein cleavage-activating protein-like [Aplysia californic
9647408	gi|405958063|gb|EKC24227.1|	0	Putative pre-mRNA-splicing factor ATP-dependent RNA helicase DHX15 [Crassostrea gigas
9648712	gi|524914499|ref|XP_005112064.1|	0	PREDICTED: hydroxymethylglutaryl-CoA synthase, cytoplasmic-like isoform X1 [Aplysia californic
9653082	gi|676474182|ref|XP_009060061.1|	0	hypothetical protein LOTGIDRAFT_106770, partial [Lottia gigantea
9652774	gi|524902505|ref|XP_005107498.1|	0	PREDICTED: rab GTPase-binding effector protein 1-like isoform X2 [Aplysia californica
9650692	gi|676459643|ref|XP_009055367.1|	0	hypothetical protein LOTGIDRAFT_209276 [Lottia gigantea]
9653380	gi|405970535|gb|EKC35431.1|	0	Histone-lysine N-methyltransferase MLL3 [Crassostrea gigas]
9652356	gi|405976339|gb|EKC40851.1|	0	Transmembrane protein C9orf5 [Crassostrea gigas]
9653444	gi|676454661|ref|XP_009053754.1|	0	hypothetical protein LOTGIDRAFT_144613 [Lottia gigantea]
9650346	gi|405951345|gb|EKC19266.1|	0	hypothetical protein CGI_10009041 [Crassostrea gigas]
9640078	gi|405953739|gb|EKC21342.1|	0	Regulator of nonsense transcripts 1 [Crassostrea gigas]
9652202	gi|405964613|gb|EKC30078.1|	0	G-protein coupled receptor 64 [Crassostrea gigas]
9640780	gi|524886501|ref|XP_005099844.1|	0	PREDICTED: cytoplasmic dynein 1 heavy chain 1-like isoform X1 [Aplysia californica
9649542	gi|405960321|gb|EKC26252.1|	0	E3 ubiquitin-protein ligase MIB2 [Crassostrea gigas
9650460	gi|676482024|ref|XP_009062564.1|	0	hypothetical protein LOTGIDRAFT_194758, partial [Lottia gigantea]
9653036	gi|443708151|gb|ELU03406.1|	0	hypothetical protein CAPTEDRAFT_168454 [Capitella teleta
9640782	gi|676485111|ref|XP_009063558.1|	0	hypothetical protein LOTGIDRAFT_221280 [Lottia gigantea]
9652916	gi|405965855|gb|EKC31204.1|	0	Muscle, skeletal receptor tyrosine protein kinase [Crassostrea gigas
9651958	gi|676445936|ref|XP_009050915.1|	0	hypothetical protein LOTGIDRAFT_206222 [Lottia gigantea]
9653336	gi|524907650|ref|XP_005108941.1|	0	PREDICTED: ATPase family AAA domain-containing protein 2B-like isoform X3 [Aplysia californica
9653362	gi|405952143|gb|EKC19988.1|	0	OTU domain-containing protein 7B [Crassostrea gigas
9653502	gi|676471444|ref|XP_009059181.1|	0	hypothetical protein LOTGIDRAFT_164410 [Lottia gigantea
9653426	gi|405956902|gb|EKC23145.1|	0	Glutamate receptor, ionotropic kainate 3, partial [Crassostrea gigas
9651660	gi|119351137|gb|ABL63470.1|	0	plasma membrane calcium ATPase [Pinctada fucata
9652932	gi|676489777|ref|XP_009065050.1|	0	hypothetical protein LOTGIDRAFT_108137 [Lottia gigantea]
9643258	gi|29378335|gb|AAO83849.1|AF484092_1	0	neural-specific syntaxin-binding protein 1 [Lymnaea stagnalis
9650794	gi|405970767|gb|EKC35643.1|	0	hypothetical protein CGI_10020334 [Crassostrea gigas]
9646774	gi|524891524|ref|XP_005102295.1|	0	PREDICTED: sterol O-acyltransferase 1-like isoform X3 [Aplysia californica]
9653528	gi|405975363|gb|EKC39929.1|	0	Lethal(3)malignant brain tumor-like 4 protein [Crassostrea gigas
9642714	gi|676452029|ref|XP_009052900.1|	0	hypothetical protein LOTGIDRAFT_214584 [Lottia gigantea]
9650058	gi|405975951|gb|EKC40479.1|	0	Zinc finger FYVE domain-containing protein 9 [Crassostrea gigas
9645488	gi|405950922|gb|EKC18877.1|	0	Metastasis-associated protein MTA1 [Crassostrea gigas]
9653250	gi|405969825|gb|EKC34773.1|	0	E3 ubiquitin-protein ligase HUWE1 [Crassostrea gigas
9643304	gi|405956993|gb|EKC23232.1|	0	Sorting nexin-8 [Crassostrea gigas]
9648864	gi|405957797|gb|EKC23980.1|	0	Ankyrin repeat domain-containing protein 17 [Crassostrea gigas
9652506	gi|676447292|ref|XP_009051360.1|	0	hypothetical protein LOTGIDRAFT_228176 [Lottia gigantea]
9645182	gi|421952591|gb|AFX71644.1|	0	G protein alpha subunit [Perinereis aibuhitensis
9653370	gi|405960496|gb|EKC26417.1|	0	Tyrosine-protein kinase Abl [Crassostrea gigas]
9652978	gi|405969557|gb|EKC34521.1|	0	TRS85-like protein [Crassostrea gigas]
9652106	gi|405963872|gb|EKC29404.1|	0	CCR4-NOT transcription complex subunit 1 [Crassostrea gigas]
9648112	gi|676421154|ref|XP_009043778.1|	0	hypothetical protein LOTGIDRAFT_227892 [Lottia gigantea]
9651080	gi|405960053|gb|EKC26005.1|	0	Frizzled-10-A [Crassostrea gigas]
9646490	gi|676472655|ref|XP_009059560.1|	0	hypothetical protein LOTGIDRAFT_124718 [Lottia gigantea]
9653126	gi|405959863|gb|EKC25846.1|	0	Tyrosine-protein phosphatase non-receptor type 14 [Crassostrea gigas]
9653544	gi|524883368|ref|XP_005098316.1|	0	PREDICTED: uncharacterized protein LOC101856179 [Aplysia californica]
9652388	gi|676480847|ref|XP_009062192.1|	0	hypothetical protein LOTGIDRAFT_154747 [Lottia gigantea]
9652944	gi|405971824|gb|EKC36634.1|	0	Fatty acyl-CoA reductase 1 [Crassostrea gigas]

9649266	gi|524879721|ref|XP_005096532.1|		0	PREDICTED: E3 ubiquitin-protein ligase TRAF7-like isoform X2 [Aplysia californica
9647384	gi|676485567|ref|XP_009063697.1|		0	hypothetical protein LOTGIDRAFT_210797 [Lottia gigantea
9651504	gi|443716615|gb|ELU08049.1|		0	hypothetical protein CAPTEDRAFT_20343 [Capitella teleta
9643400	gi|405949971|gb|EKC17981.1|		0	Putative phospholipid-transporting ATPase IIB [Crassostrea gigas
9648190	gi|125415|sp|P21613.1|KINH_DORPE		0	RecName: Full=Kinesin heavy chain [Doryteuthis pealeii
9639570	gi|405965007|gb|EKC30438.1|		0	DNA-directed RNA polymerase II subunit RPB1 [Crassostrea gigas
9652374	gi|405950968|gb|EKC18920.1|		0	WD repeat-containing protein 7 [Crassostrea gigas]
9648220	gi|405964798|gb|EKC30244.1|		0	Coatomer subunit beta [Crassostrea gigas]
9653146	gi|405960766|gb|EKC26649.1|		0	Multidrug resistance-associated protein 4 [Crassostrea gigas]
9650406	gi|405968487|gb|EKC33554.1|		0	E3 ubiquitin-protein ligase UBR5 [Crassostrea gigas
9653088	gi|405955267|gb|EKC22446.1|		0	Kinesin-like protein KIF17 [Crassostrea gigas]
9653450	gi|524892005|ref|XP_005102534.1|		0	PREDICTED: E3 ubiquitin-protein ligase MIB1-like [Aplysia californica
9647734	gi|524866556|ref|XP_005090099.1|		0	PREDICTED: non-specific lipid-transfer protein-like isoform X1 [Aplysia californica
9653168	gi|405969488|gb|EKC34457.1|		0	hypothetical protein CGI_10012737 [Crassostrea gigas]
9653530	gi|676456145|ref|XP_009054232.1|		0	hypothetical protein LOTGIDRAFT_160793 [Lottia gigantea]
9648184	gi|524870022|ref|XP_005091797.1|		0	PREDICTED: formin-like protein CG32138-like isoform X3 [Aplysia californica
9648156	gi|405965002|gb|EKC30433.1|		0	Suppressor of fused-like protein [Crassostrea gigas]
9652864	gi|405954105|gb|EKC21632.1|		0	hypothetical protein CGI_10003599 [Crassostrea gigas]
9651068	gi|405952425|gb|EKC20239.1|		0	Baculoviral IAP repeat-containing protein 6 [Crassostrea gigas
9652534	gi|405977192|gb|EKC41654.1|		0	Putative E3 ubiquitin-protein ligase MYCBP2 [Crassostrea gigas
9649108	gi|524866025|ref|XP_005089837.1|		0	PREDICTED: dynactin subunit 1-like isoform X5 [Aplysia californica
9650000	gi|405967745|gb|EKC32876.1|		0	G-protein-signaling modulator 2 [Crassostrea gigas
9652900	gi|676493869|ref|XP_009066377.1|		0	hypothetical protein LOTGIDRAFT_236919 [Lottia gigantea]
9639160	gi|676420120|ref|XP_009043632.1|		0	hypothetical protein LOTGIDRAFT_151884 [Lottia gigantea]
9652672	gi|524883489|ref|XP_005098372.1|		0	PREDICTED: type II inositol 1,4,5-trisphosphate 5-phosphatase-like isoform X2 [Aplysia californica
9650676	gi|524876259|ref|XP_005094848.1|		0	PREDICTED: tyrosine-protein phosphatase non-receptor type 9-like isoform X2 [Aplysia californica
9647640	gi|405959093|gb|EKC25161.1|		0	Neurotrypsin [Crassostrea gigas]
9645168	gi|676423450|ref|XP_009044105.1|		0	hypothetical protein LOTGIDRAFT_208557 [Lottia gigantea]
9652094	gi|676483328|ref|XP_009062989.1|		0	hypothetical protein LOTGIDRAFT_129137 [Lottia gigantea]
9650788	gi|405977192|gb|EKC41654.1|		0	Putative E3 ubiquitin-protein ligase MYCBP2 [Crassostrea gigas
9646012	gi|405966159|gb|EKC31472.1|		0	Nuclear cap-binding protein subunit 1 [Crassostrea gigas
9648300	gi|405966143|gb|EKC31461.1|		0	DnaJ-like protein subfamily C member 13 [Crassostrea gigas
9653554	gi|405971799|gb|EKC36611.1|		0	Enhancer of mRNA-decapping protein 4 [Crassostrea gigas
9652504	gi|405976591|gb|EKC41092.1|		0	bicaudal D-like protein 2 [Crassostrea gigas]
9653392	gi|405966604|gb|EKC31867.1|		0	Protein dopey-2 [Crassostrea gigas]
9647678	gi|405950585|gb|EKC18563.1|		0	WD repeat-containing protein 26 [Crassostrea gigas]
9652650	gi|405973235|gb|EKC37959.1|		0	Protocadherin Fat 1 [Crassostrea gigas]
9648432	gi|405977605|gb|EKC42047.1|		0	Cullin-3-B [Crassostrea gigas]
9650736	gi|405964470|gb|EKC29952.1|		0	DmX-like protein 2 [Crassostrea gigas]
9649298	gi|524892499|ref|XP_005102777.1|		0	PREDICTED: WD repeat-containing protein 20-like [Aplysia californica
9651102	gi|405951298|gb|EKC19223.1|		0	Cullin-2 [Crassostrea gigas]
9649486	gi|524866560|ref|XP_005090101.1|		0	PREDICTED: nipped-B-like protein B-like isoform X1 [Aplysia californica
9651380	gi|405977906|gb|EKC42333.1|		0	Non-lysosomal glucosylceramidase [Crassostrea gigas]
9641394	gi|524884255|ref|XP_005098749.1|		0	PREDICTED: probable S-acyltransferase At2g14255-like [Aplysia californica
9645854	gi|405964762|gb|EKC30211.1|		0	UPF0505 protein C16orf62-like protein [Crassostrea gigas]
9648284	gi|7524150|gb|AAD34642.2|AF154109_1		0	E3 ubiquitin-protein ligase [Mya arenaria
9653476	gi|676457963|ref|XP_009054821.1|		0	hypothetical protein LOTGIDRAFT_215534 [Lottia gigantea]
9649926	gi|676427982|ref|XP_009045125.1|		0	hypothetical protein LOTGIDRAFT_207546 [Lottia gigantea
9652100	gi|676440086|ref|XP_009049061.1|		0	hypothetical protein LOTGIDRAFT_225973 [Lottia gigantea
9598481	gi|524892063|ref|XP_005102563.1|	1.00378e-93		PREDICTED: importin-7-like [Aplysia californica
9602084	gi|676489652|ref|XP_009065009.1|	1.00525e-38		hypothetical protein LOTGIDRAFT_207292 [Lottia gigantea]
9638872	gi|405969882|gb|EKC34827.1|	1.00712e-172		Importin-7 [Crassostrea gigas]
9622556	gi|405976491|gb|EKC40996.1|	1.01255e-25		PRKC apoptosis WT1 regulator protein [Crassostrea gigas]
9639594	gi|676460774|ref|XP_009055731.1|	1.01297e-143		hypothetical protein LOTGIDRAFT_232605 [Lottia gigantea]
9613792	gi|405965297|gb|EKC30679.1|	1.01645e-57		YTH domain family protein 1 [Crassostrea gigas
9615452	gi|676468171|ref|XP_009058123.1|	1.0196e-60		hypothetical protein LOTGIDRAFT_228850 [Lottia gigantea]
9585375	gi|556965032|ref|XP_005992169.1|	1.01986e-15		PREDICTED: probable peptidyl-tRNA hydrolase [Latimeria chalumnae
9643930	gi|676430966|ref|XP_009046099.1|	1.02021e-132		hypothetical protein LOTGIDRAFT_137839 [Lottia gigantea
9645530	gi|405950089|gb|EKC18095.1|	1.02148e-133		Alpha-(1,3)-fucosyltransferase 10 [Crassostrea gigas]
9642684	gi|405952069|gb|EKC19921.1|	1.02301e-86		Leucine zipper putative tumor suppressor 2-like protein [Crassostrea gigas
9626116	gi|524881876|ref|XP_005097586.1|	1.0236e-18		PREDICTED: solute carrier family 12 member 9-like [Aplysia californica
9624484	gi|405978367|gb|EKC42766.1|	1.02486e-44		CREB-binding protein [Crassostrea gigas]
9644022	gi|676431058|ref|XP_009046126.1|	1.02499e-23		hypothetical protein LOTGIDRAFT_171688 [Lottia gigantea]
9634202	gi|676439874|ref|XP_009048992.1|	1.02508e-114		hypothetical protein LOTGIDRAFT_61540, partial [Lottia gigantea]
9595269	gi|405945173|gb|EKC17185.1|	1.02623e-28		hypothetical protein CGI_10002027 [Crassostrea gigas]
9640160	gi|676441129|ref|XP_009049397.1|	1.0307e-66		hypothetical protein LOTGIDRAFT_230885 [Lottia gigantea]
9575800	gi|405950309|gb|EKC18305.1|	1.03483e-16		hypothetical protein CGI_10013720 [Crassostrea gigas]
9586333	gi|676421803|ref|XP_009043862.1|	1.03962e-23		hypothetical protein LOTGIDRAFT_149165 [Lottia gigantea]
9633264	gi|405952662|gb|EKC20447.1|	1.04311e-156		C-jun-amino-terminal kinase-interacting protein 4 [Crassostrea gigas
9589387	gi|405951037|gb|EKC18984.1|	1.04336e-53		Calsyntenin-1 [Crassostrea gigas]

9613822	gi|676455086|ref|XP_009053891.1|	1.047e-70	hypothetical protein LOTGIDRAFT_116873 [Lottia gigantea]
9650038	gi|676477934|ref|XP_009061266.1|	1.04895e-142	hypothetical protein LOTGIDRAFT_126794 [Lottia gigantea]
9650582	gi|405963591|gb|EKC29153.1|	1.0525e-31	hypothetical protein CGI_10024275 [Crassostrea gigas]
9609270	gi|676437147|ref|XP_009048109.1|	1.05404e-66	hypothetical protein LOTGIDRAFT_139785 [Lottia gigantea]
9620502	gi|405958252|gb|EKC24397.1|	1.05662e-85	Myosin-Ic [Crassostrea gigas]
9615204	gi|676440953|ref|XP_009049340.1|	1.05696e-17	hypothetical protein LOTGIDRAFT_230852 [Lottia gigantea]
9651338	gi|405966671|gb|EKC31928.1|	1.05737e-72	Lateral signaling target protein 2-like protein [Crassostrea gigas
9631948	gi|405967289|gb|EKC32469.1|	1.05765e-41	Metal regulatory transcription factor 1 [Crassostrea gigas]
9646186	gi|524881528|ref|XP_005097415.1|	1.05787e-64	PREDICTED: uncharacterized protein LOC101848656 isoform X1 [Aplysia californica]
9624122	gi|405972638|gb|EKC37398.1|	1.06285e-17	Protein AMBP [Crassostrea gigas]
9645076	gi|676490644|ref|XP_009065337.1|	1.062e-127	hypothetical protein LOTGIDRAFT_155546 [Lottia gigantea
9574304	gi|405964786|gb|EKC30232.1|	1.06375e-45	F-box/WD repeat-containing protein 9 [Crassostrea gigas]
9637982	gi|405966980|gb|EKC32200.1|	1.06423e-62	Cell division control protein 42-like protein [Crassostrea gigas
9582197	gi|260808666|ref|XP_002599128.1|	1.0654e-11	hypothetical protein BRAFLDRAFT_122979 [Branchiostoma floridae
9630826	gi|405978882|gb|EKC43241.1|	1.06981e-91	hypothetical protein CGI_10013463 [Crassostrea gigas]
9648256	gi|405954002|gb|EKC21552.1|	1.07415e-15	hypothetical protein CGI_10003779 [Crassostrea gigas]
9645752	gi|339251812|ref|XP_003372928.1|	1.07615e-13	hypothetical protein Tsp_10483 [Trichinella spiralis]
9644890	gi|405963794|gb|EKC29340.1|	1.07636e-39	Genetic suppressor element 1 [Crassostrea gigas]
9645818	gi|405968000|gb|EKC33109.1|	1.07672e-146	Ubiquitin carboxyl-terminal hydrolase 8 [Crassostrea gigas
9640296	gi|391329773|ref|XP_003739342.1|	1.07763e-55	PREDICTED: metabotropic glutamate receptor-like [Metaseiulus occidentalis
9648218	gi|676465212|ref|XP_009057171.1|	1.07853e-108	hypothetical protein LOTGIDRAFT_121393 [Lottia gigantea]
9642436	gi|676483760|ref|XP_009063128.1|	1.07888e-72	hypothetical protein LOTGIDRAFT_220842 [Lottia gigantea]
9634090	gi|676454834|ref|XP_009053810.1|	1.08195e-140	hypothetical protein LOTGIDRAFT_232003 [Lottia gigantea]
9647992	gi|405960594|gb|EKC26506.1|	1.08554e-12	hypothetical protein CGI_10002601 [Crassostrea gigas]
9649212	gi|524905969|ref|XP_005108340.1|	1.09087e-53	PREDICTED: 2',3'-cyclic-nucleotide 3'-phosphodiesterase-like isoform X1 [Aplysia californica]
9649908	gi|676439884|ref|XP_009048995.1|	1.09523e-176	hypothetical protein LOTGIDRAFT_173279 [Lottia gigantea]
9597823	gi|405957286|gb|EKC23509.1|	1.09527e-84	Vacuolar protein sorting-associated protein 16-like protein [Crassostrea gigas
9642040	gi|405964299|gb|EKC29799.1|	1.0956e-69	Transmembrane protein 64 [Crassostrea gigas]
9648234	gi|676435916|ref|XP_009047712.1|	1.09619e-52	hypothetical protein LOTGIDRAFT_225295 [Lottia gigantea]
9649642	gi|405952142|gb|EKC19987.1|	1.1038e-48	hypothetical protein CGI_10007164 [Crassostrea gigas]
9617564	gi|405969694|gb|EKC34648.1|	1.10431e-97	Serine/threonine-protein phosphatase 4 regulatory subunit 1 [Crassostrea gigas
9644034	gi|405952881|gb|EKC20639.1|	1.10728e-102	Low-density lipoprotein receptor-related protein 5 [Crassostrea gigas
9569790	gi|405975785|gb|EKC40331.1|	1.11626e-55	Filamin-A [Crassostrea gigas]
9572190	gi|405972762|gb|EKC37512.1|	1.11711e-18	Organic cation transporter-like protein [Crassostrea gigas]
9638332	gi|405951713|gb|EKC19603.1|	1.11828e-67	Nuclear factor erythroid 2-related factor 2 [Crassostrea gigas]
9579946	gi|443722677|gb|ELU11437.1|	1.11959e-26	hypothetical protein CAPTEDRAFT_221587 [Capitella teleta
9649088	gi|405950692|gb|EKC18663.1|	1.11964e-34	Bcl-2-like protein 13 [Crassostrea gigas]
9584805	gi|24583258|ref|NP_609357.2|	1.12312e-67	Niemann-Pick type C-1a, isoform A [Drosophila melanogaster]
9577946	gi|270011525|gb|EFA07973.1|	1.12808e-18	hypothetical protein TcasGA2_TC005555 [Tribolium castaneum
9602138	gi|524895733|ref|XP_005104348.1|	1.12901e-98	PREDICTED: ER lumen protein retaining receptor 2-like isoform X1 [Aplysia californica
9638290	gi|390332699|ref|XP_785809.3|	1.13162e-11	PREDICTED: uncharacterized protein LOC580672 [Strongylocentrotus purpuratus
9638006	gi|405950617|gb|EKC18593.1|	1.13177e-80	Regulator of microtubule dynamics protein 1 [Crassostrea gigas
9621938	gi|405951983|gb|EKC19845.1|	1.13376e-13	Serine/threonine-protein phosphatase 1 regulatory subunit 10 [Crassostrea gigas
9651470	gi|74039697|gb|AAZ94880.1|	1.1338e-122	ecto-nucleosidase triphosphate diphosphohydrolase 7 [Xenopus laevis
9642292	gi|405977691|gb|EKC42127.1|	1.13391e-106	Solute carrier family 22 member 6-A [Crassostrea gigas
9590033	gi|405951241|gb|EKC19171.1|	1.13396e-63	Neurobeachin-like protein 2 [Crassostrea gigas]
9640084	gi|524890649|ref|XP_005101868.1|	1.13501e-65	PREDICTED: toll-like receptor 13-like [Aplysia californica
9636278	gi|676468340|ref|XP_009058178.1|	1.14079e-127	hypothetical protein LOTGIDRAFT_205708 [Lottia gigantea
9633524	gi|405959296|gb|EKC25348.1|	1.14148e-94	ATP-binding cassette transporter sub-family C member 9 [Crassostrea gigas
9640068	gi|405959093|gb|EKC25161.1|	1.15296e-126	Neurotrypsin [Crassostrea gigas]
9652130	gi|405963290|gb|EKC28877.1|	1.1552e-46	hypothetical protein CGI_10009223 [Crassostrea gigas]
9613642	gi|524880698|ref|XP_005097010.1|	1.15646e-37	PREDICTED: protein enabled homolog [Aplysia californica
9644946	gi|443721783|gb|ELU10963.1|	1.15822e-142	hypothetical protein CAPTEDRAFT_109432 [Capitella teleta
9612530	gi|405958470|gb|EKC24597.1|	1.15973e-34	Papilin [Crassostrea gigas]
9568348	gi|642932900|ref|XP_008197178.1|	1.1608e-44	PREDICTED: carboxypeptidase M isoform X1 [Tribolium castaneum
9595109	gi|405969968|gb|EKC34911.1|	1.16331e-56	Circularly permutated Ras protein 1 [Crassostrea gigas
9621878	gi|405977508|gb|EKC41951.1|	1.16513e-34	Protein slit [Crassostrea gigas]
9639794	gi|676479272|ref|XP_009061697.1|	1.16853e-139	hypothetical protein LOTGIDRAFT_127673 [Lottia gigantea]
9616010	gi|676463356|ref|XP_009056571.1|	1.16984e-80	hypothetical protein LOTGIDRAFT_162365 [Lottia gigantea]
9573446	gi|524910197|ref|XP_005109980.1|	1.17099e-68	PREDICTED: alpha-N-acetylgalactosaminidase-like isoform X1 [Aplysia californica
9645826	gi|405971475|gb|EKC36310.1|	1.17464e-76	Fatty acid 2-hydroxylase [Crassostrea gigas]
9651738	gi|405957595|gb|EKC23796.1|	1.17589e-162	Uncharacterized protein C4orf8 [Crassostrea gigas]
9622370	gi|524889255|ref|XP_005101188.1|	1.18392e-34	PREDICTED: uncharacterized protein LOC101853466 isoform X1 [Aplysia californica]
9652994	gi|405960875|gb|EKC26749.1|	1.18861e-176	hypothetical protein CGI_10028288 [Crassostrea gigas]
9607364	gi|260816533|ref|XP_002603025.1|	1.18994e-59	hypothetical protein BRAFLDRAFT_59438 [Branchiostoma floridae]
9625574	gi|405962407|gb|EKC28088.1|	1.19121e-112	Transmembrane 9 superfamily member 1 [Crassostrea gigas
9623950	gi|676444217|ref|XP_009050357.1|	1.19299e-53	hypothetical protein LOTGIDRAFT_231137 [Lottia gigantea]
9583241	gi|504139160|ref|XP_004582033.1|	1.19485e-42	PREDICTED: sortilin isoform X2 [Ochotona princeps
9633538	gi|697000185|ref|XP_009566178.1|	1.1978e-43	PREDICTED: cell division control protein 42 homolog isoform X1 [Cuculus canorus
9640952	gi|405951336|gb|EKC19257.1|	1.19898e-25	Potassium voltage-gated channel protein Shaw [Crassostrea gigas

9652030	gi|405958078|gb|EKC24241.1|	1.2063e-40	Ubiquitin-associated protein 1 [Crassostrea gigas]
9648050	gi|676427674|ref|XP_009045024.1|	1.21091e-112	hypothetical protein LOTGIDRAFT_170919 [Lottia gigantea]
9633626	gi|405968017|gb|EKC33125.1|	1.2148e-128	Adenosine monophosphate-protein transferase FICD [Crassostrea gigas]
9639250	gi|405960153|gb|EKC26098.1|	1.21621e-40	Neural/ectodermal development factor IMP-L2 [Crassostrea gigas
9589667	gi|405977168|gb|EKC41631.1|	1.21827e-94	Signal recognition particle 54 kDa protein [Crassostrea gigas
9619958	gi|405967409|gb|EKC32573.1|	1.21848e-110	Vacuolar protein sorting-associated protein 35, partial [Crassostrea gigas
9573810	gi|543747494|ref|XP_005514505.1|	1.22024e-41	PREDICTED: LOW QUALITY PROTEIN: thymidine kinase 2, mitochondrial [Columba livi
9642832	gi|524915917|ref|XP_005112745.1|	1.22539e-63	PREDICTED: tRNA pseudouridine(38/39) synthase-like [Aplysia californica
9639422	gi|405977285|gb|EKC41744.1|	1.22583e-133	E3 ubiquitin-protein ligase Siah1 [Crassostrea gigas
9619778	gi|195050907|ref|XP_001992993.1|	1.22984e-78	GH13340 [Drosophila grimshawi]
9648030	gi|676447469|ref|XP_009051417.1|	1.23006e-173	hypothetical protein LOTGIDRAFT_208735 [Lottia gigantea
9633954	gi|524887879|ref|XP_005100522.1|	1.23429e-128	PREDICTED: striatin-3-like isoform X3 [Aplysia californica]
9644526	gi|676468640|ref|XP_009058274.1|	1.237e-165	hypothetical protein LOTGIDRAFT_122405 [Lottia gigantea
9626238	gi|405955808|gb|EKC22769.1|	1.23971e-48	B-Raf proto-oncogene serine/threonine-protein kinase [Crassostrea gigas]
9650256	gi|676491687|ref|XP_009065676.1|	1.24197e-106	hypothetical protein LOTGIDRAFT_169123 [Lottia gigantea]
9619484	gi|676445630|ref|XP_009050816.1|	1.24752e-38	hypothetical protein LOTGIDRAFT_174238 [Lottia gigantea]
9641728	gi|524876850|ref|XP_005095134.1|	1.24902e-142	PREDICTED: signal recognition particle subunit SRP68-like [Aplysia californica
9648992	gi|524897288|ref|XP_005105107.1|	1.25262e-70	PREDICTED: probable protein phosphatase CG10417-like [Aplysia californica
9595479	gi|676469255|ref|XP_009058473.1|	1.25739e-18	hypothetical protein LOTGIDRAFT_163705 [Lottia gigantea
9575946	gi|554850409|ref|XP_005935473.1|	1.25914e-30	PREDICTED: cleavage and polyadenylation specificity factor subunit 2-like [Haplochromis burtor
9600818	gi|676427493|ref|XP_009044965.1|	1.26034e-30	hypothetical protein LOTGIDRAFT_136300, partial [Lottia gigantea
9571386	gi|524864001|ref|XP_005088839.1|	1.26341e-54	PREDICTED: parafibromin-like isoform X2 [Aplysia californica
9639490	gi|676460695|ref|XP_009055708.1|	1.26567e-37	hypothetical protein LOTGIDRAFT_232589 [Lottia gigantea]
9646446	gi|676491726|ref|XP_009065688.1|	1.2656e-97	hypothetical protein LOTGIDRAFT_236498 [Lottia gigantea]
9635518	gi|405978104|gb|EKC42518.1|	1.2662e-38	Afadin [Crassostrea gigas]
9613550	gi|405974895|gb|EKC39507.1|	1.2727e-52	Son of sevenless-like protein 2 [Crassostrea gigas]
9652034	gi|405977111|gb|EKC41577.1|	1.27281e-90	Cytochrome P450 2J6 [Crassostrea gigas]
9646316	gi|676490571|ref|XP_009065313.1|	1.27459e-150	hypothetical protein LOTGIDRAFT_211131 [Lottia gigantea]
9646748	gi|405957663|gb|EKC23860.1|	1.27584e-28	BAT2 domain-containing protein 1 [Crassostrea gigas
9595205	gi|291236175|ref|XP_002738015.1|	1.28321e-63	PREDICTED: thymus-specific serine protease-like [Saccoglossus kowalevskii
9594139	gi|524865310|ref|XP_005089485.1|	1.28588e-52	PREDICTED: synaptobrevin homolog YKT6-like [Aplysia californica
9626690	gi|405956937|gb|EKC23179.1|	1.28891e-101	C-myc promoter-binding protein [Crassostrea gigas
9589313	gi|675366163|gb|KFM59065.1|	1.2903e-11	Nuclear pore complex protein Nup98-Nup96, partial [Stegodyphus mimosarur
9652246	gi|524878182|ref|XP_005095780.1|	1.2911e-125	PREDICTED: large neutral amino acids transporter small subunit 1-like isoform X1 [Aplysia californic
9651804	gi|676450212|ref|XP_009052305.1|	1.29331e-100	hypothetical protein LOTGIDRAFT_231644 [Lottia gigantea
9586415	gi|405957935|gb|EKC24111.1|	1.29694e-66	hypothetical protein CGI_10016657 [Crassostrea gigas]
9584273	gi|405975573|gb|EKC40131.1|	1.29722e-68	LAG1 longevity assurance-like protein 6 [Crassostrea gigas
9652714	gi|291235369|ref|XP_002737617.1|	1.30569e-49	PREDICTED: uncharacterized family 31 glucosidase KIAA1161-like [Saccoglossus kowalevski
9602492	gi|676463991|ref|XP_009056779.1|	1.30682e-54	hypothetical protein LOTGIDRAFT_233079 [Lottia gigantea]
9580228	gi|675858342|ref|XP_009014731.1|	1.30729e-67	hypothetical protein HELRODRAFT_76326 [Helobdella robusta]
9645150	gi|405966238|gb|EKC31545.1|	1.30904e-117	Cytokine receptor-like factor 3 [Crassostrea gigas]
9649028	gi|625228533|ref|XP_007653432.1|	1.31404e-35	PREDICTED: LOW QUALITY PROTEIN: extracellular calcium-sensing receptor-like isoform X1, partial [Cricetulus griseu
9608676	gi|431923345|gb|AGA94627.1|	1.32398e-45	insulin-related peptide receptor [Pinctada fucata
9571212	gi|405965593|gb|EKC30956.1|	1.323e-19	E3 ubiquitin-protein ligase HECTD1 [Crassostrea gigas
9646068	gi|676443642|ref|XP_009050181.1|	1.32706e-59	hypothetical protein LOTGIDRAFT_238844 [Lottia gigantea
9600024	gi|607357226|gb|EZA51692.1|	1.32984e-24	Acetylcholine receptor subunit alpha-like protein [Cerapachys biro
9639316	gi|405953739|gb|EKC21342.1|	1.33164e-87	Regulator of nonsense transcripts 1 [Crassostrea gigas]
9591279	gi|405953739|gb|EKC21342.1|	1.33488e-91	Regulator of nonsense transcripts 1 [Crassostrea gigas]
9643386	gi|556969164|ref|XP_005993418.1|	1.33496e-12	PREDICTED: TGF-beta-activated kinase 1 and MAP3K7-binding protein 2 [Latimeria chalumna
9645284	gi|405970126|gb|EKC35058.1|	1.33617e-77	Transcriptional-regulating factor 1 [Crassostrea gigas]
9639530	gi|405974511|gb|EKC39150.1|	1.3391e-87	Calpain-9 [Crassostrea gigas]
9628338	gi|405951139|gb|EKC19078.1|	1.34507e-24	Low-density lipoprotein receptor-related protein 1B [Crassostrea gigas
9584689	gi|585642963|ref|XP_006811269.1|	1.34529e-20	PREDICTED: uncharacterized protein LOC102810221 [Saccoglossus kowalevskii
9652284	gi|405972705|gb|EKC37459.1|	1.34619e-13	hypothetical protein CGI_10011439 [Crassostrea gigas]
9595777	gi|405966133|gb|EKC31451.1|	1.3473e-68	Rap guanine nucleotide exchange factor 1 [Crassostrea gigas
9595573	gi|524881876|ref|XP_005097586.1|	1.3473e-68	PREDICTED: solute carrier family 12 member 9-like [Aplysia californica
9579614	gi|242023566|ref|XP_002432203.1|	1.35283e-64	glycylpeptide N-tetRadecanoyltransferase, putative [Pediculus humanus corpori
9584409	gi|548342653|ref|XP_005723813.1|	1.35709e-105	PREDICTED: serine/threonine-protein phosphatase PP1-beta catalytic subunit-like isoform X2 [Pundamilia nyerere
9632098	gi|676461082|ref|XP_009055830.1|	1.35878e-137	hypothetical protein LOTGIDRAFT_119149, partial [Lottia gigantea]
9638748	gi|524879420|ref|XP_005096386.1|	1.36167e-153	PREDICTED: mitogen-activated protein kinase kinase kinase kinase 3-like [Aplysia californica
9569182	gi|744457|prf||2014371A	1.36804e-53	kinesin
9639650	gi|405976887|gb|EKC41365.1|	1.36907e-140	Tyrosine-protein phosphatase non-receptor type 4 [Crassostrea gigas
9646408	gi|405950242|gb|EKC18241.1|	1.37057e-43	Glycoprotein 3-alpha-L-fucosyltransferase A [Crassostrea gigas]
9584665	gi|571514070|ref|XP_006568759.1|	1.38893e-25	PREDICTED: insulin-like receptor-like isoform X3 [Apis mellifera
9586991	gi|524903658|ref|XP_005108024.1|	1.38989e-15	PREDICTED: mediator of RNA polymerase II transcription subunit 15a-like isoform X2 [Aplysia californica
9593743	gi|676444702|ref|XP_009050514.1|	1.3903e-67	hypothetical protein LOTGIDRAFT_114108, partial [Lottia gigantea
9574040	gi|405973205|gb|EKC37931.1|	1.3929e-45	hypothetical protein CGI_10009705 [Crassostrea gigas]
9613152	gi|524871019|ref|XP_005092286.1|	1.39586e-43	PREDICTED: nose resistant to fluoxetine protein 6-like [Aplysia californica
9652090	gi|405969040|gb|EKC34051.1|	1.39642e-169	hypothetical protein CGI_10013747 [Crassostrea gigas]
9635734	gi|405961931|gb|EKC27662.1|	1.39709e-61	hypothetical protein CGI_10001842 [Crassostrea gigas]

9601338	gi|405949971|gb|EKC17981.1|	1.39766e-69	Putative phospholipid-transporting ATPase IIB [Crassostrea gigas
9641964	gi|585679353|ref|XP_006819500.1|	1.397e-24	PREDICTED: nipped-B-like protein-like isoform X1 [Saccoglossus kowalevski
9624262	gi|405973516|gb|EKC38224.1|	1.40429e-118	Spectrin alpha chain [Crassostrea gigas]
9633110	gi|665804686|ref|XP_008550500.1|	1.40507e-12	PREDICTED: serine/arginine repetitive matrix protein 2-like [Microplitis demolito
9620396	gi|511843304|ref|XP_004746100.1|	1.40583e-21	PREDICTED: collagen alpha-1(IV) chain isoform X2 [Mustela putorius furc
9643144	gi|405966259|gb|EKC31566.1|	1.40644e-131	WD repeat-containing protein 47 [Crassostrea gigas]
9627594	gi|524906359|ref|XP_005108493.1|	1.41798e-119	PREDICTED: flotillin-2-like [Aplysia californica
9626426	gi|524870130|ref|XP_005091851.1|	1.4185e-68	PREDICTED: trithorax group protein osa-like [Aplysia californica
9587491	gi|405951802|gb|EKC19683.1|	1.41884e-15	MKL/myocardin-like protein 2 [Crassostrea gigas]
9641128	gi|405976244|gb|EKC40757.1|	1.42312e-151	Neutral ceramidase B [Crassostrea gigas]
9618704	gi|524866201|ref|XP_005089924.1|	1.42558e-63	PREDICTED: reelin-like [Aplysia californica
9575442	gi|260821041|ref|XP_002605842.1|	1.42734e-58	hypothetical protein BRAFLDRAFT_84327 [Branchiostoma floridae]
9638838	gi|405962526|gb|EKC28192.1|	1.43164e-110	Suppressor of hairless-like protein [Crassostrea gigas]
9587093	gi|405974202|gb|EKC38865.1|	1.43165e-28	Nose resistant to fluoxetine protein 6 [Crassostrea gigas]
9620040	gi|405957422|gb|EKC23633.1|	1.43269e-127	Phosphatidylinositol 4-kinase type 2-beta [Crassostrea gigas
9629010	gi|405962823|gb|EKC28466.1|	1.43446e-90	transport protein Sec16A [Crassostrea gigas]
9649252	gi|405974070|gb|EKC38740.1|	1.43998e-146	Guanine nucleotide-binding protein subunit alpha-12 [Crassostrea gigas
9651754	gi|405950972|gb|EKC18923.1|	1.44628e-144	hypothetical protein CGI_10010397 [Crassostrea gigas]
9647990	gi|557013308|ref|XP_006007195.1|	1.44688e-57	PREDICTED: mTERF domain-containing protein 1, mitochondrial [Latimeria chalumna
9590883	gi|405967412|gb|EKC32576.1|	1.45191e-82	RING finger protein 31 [Crassostrea gigas]
9642290	gi|676484440|ref|XP_009063347.1|	1.4545e-53	hypothetical protein LOTGIDRAFT_129853 [Lottia gigantea]
9647602	gi|405968904|gb|EKC33930.1|	1.45609e-139	hypothetical protein CGI_10017863 [Crassostrea gigas]
9607456	gi|676477105|ref|XP_009061006.1|	1.45798e-49	hypothetical protein LOTGIDRAFT_126248 [Lottia gigantea]
9649976	gi|676441158|ref|XP_009049406.1|	1.45904e-128	hypothetical protein LOTGIDRAFT_113008 [Lottia gigantea
9633020	gi|405974324|gb|EKC38980.1|	1.46677e-94	Large neutral amino acids transporter small subunit 2 [Crassostrea gigas
9587831	gi|676429386|ref|XP_009045583.1|	1.46827e-12	hypothetical protein LOTGIDRAFT_237555 [Lottia gigantea]
9650818	gi|676433551|ref|XP_009046937.1|	1.47065e-145	hypothetical protein LOTGIDRAFT_138558 [Lottia gigantea]
9648880	gi|676431199|ref|XP_009046172.1|	1.4736e-55	hypothetical protein LOTGIDRAFT_171725 [Lottia gigantea
9636196	gi|405977230|gb|EKC41689.1|	1.48619e-119	Amidophosphoribosyltransferase [Crassostrea gigas]
9646152	gi|676425933|ref|XP_009044462.1|	1.48644e-42	hypothetical protein LOTGIDRAFT_156198 [Lottia gigantea]
9634614	gi|390338994|ref|XP_003724902.1|	1.48682e-36	PREDICTED: uncharacterized protein LOC100891763 [Strongylocentrotus purpuratus
9625778	gi|676456339|ref|XP_009054292.1|	1.48719e-62	hypothetical protein LOTGIDRAFT_160862 [Lottia gigantea]
9645342	gi|541047454|gb|ERG86195.1|	1.48857e-60	low-density lipoprotein receptor-related protein 1 [Ascaris suum
9605290	gi|405954301|gb|EKC21781.1|	1.48932e-104	MAP kinase-activated protein kinase 2 [Crassostrea gigas]
9608228	gi|405968811|gb|EKC33840.1|	1.49354e-49	FYVE, RhoGEF and PH domain-containing protein 1 [Crassostrea gigas
9629570	gi|676473324|ref|XP_009059781.1|	1.49451e-151	hypothetical protein LOTGIDRAFT_218809 [Lottia gigantea]
9616580	gi|676476535|ref|XP_009060824.1|	1.49813e-99	hypothetical protein LOTGIDRAFT_219163 [Lottia gigantea]
9640506	gi|676428381|ref|XP_009045257.1|	1.50165e-89	hypothetical protein LOTGIDRAFT_110501 [Lottia gigantea]
9650176	gi|405973033|gb|EKC37770.1|	1.5038e-104	Monocarboxylate transporter 12 [Crassostrea gigas]
9622078	gi|676431009|ref|XP_009046110.1|	1.50416e-74	hypothetical protein LOTGIDRAFT_224600 [Lottia gigantea]
9643334	gi|524895937|ref|XP_005104448.1|	1.50473e-48	PREDICTED: uncharacterized protein LOC101854514 isoform X1 [Aplysia californica]
9647108	gi|405977797|gb|EKC42231.1|	1.505e-133	hypothetical protein CGI_10028030 [Crassostrea gigas]
9652502	gi|676461795|ref|XP_009056058.1|	1.50763e-47	hypothetical protein LOTGIDRAFT_232765 [Lottia gigantea]
9622736	gi|524903013|ref|XP_005107725.1|	1.50955e-29	PREDICTED: uncharacterized protein LOC101855213 isoform X1 [Aplysia californica]
9638644	gi|405950083|gb|EKC18089.1|	1.51005e-112	Solute carrier family 17 member 9 [Crassostrea gigas]
9652156	gi|405961309|gb|EKC27135.1|	1.51007e-62	Nuclear receptor subfamily 1 group D member 2 [Crassostrea gigas
9622028	gi|524895348|ref|XP_005104159.1|	1.51115e-58	PREDICTED: apoptosis-stimulating of p53 protein 2-like isoform X3 [Aplysia californica
9649200	gi|405965836|gb|EKC31185.1|	1.51542e-63	hypothetical protein CGI_10020761 [Crassostrea gigas]
9634448	gi|405958387|gb|EKC24520.1|	1.51579e-49	Autophagy-related protein 9A [Crassostrea gigas
9624050	gi|572430684|gb|AHF51978.1|	1.51705e-56	serum response factor [Crassostrea hongkongensis]
9612242	gi|405950969|gb|EKC18921.1|	1.51872e-103	Ras-related protein Rab-2 [Crassostrea gigas]
9617150	gi|405973691|gb|EKC38388.1|	1.5196e-115	Cytohesin-1 [Crassostrea gigas]
9592229	gi|524901899|ref|XP_005107353.1|	1.52074e-50	PREDICTED: serine/threonine-protein kinase TBK1-like [Aplysia californica
9650360	gi|405966685|gb|EKC31939.1|	1.52078e-35	Poly [ADP-ribose] polymerase 12 [Crassostrea gigas]
9636192	gi|405972029|gb|EKC36826.1|	1.52106e-39	hypothetical protein CGI_10022778 [Crassostrea gigas]
9643094	gi|676429386|ref|XP_009045583.1|	1.5256e-69	hypothetical protein LOTGIDRAFT_237555 [Lottia gigantea]
9641238	gi|405975501|gb|EKC40060.1|	1.52582e-52	RING finger protein 11 [Crassostrea gigas]
9625016	gi|676433472|ref|XP_009046911.1|	1.52927e-96	hypothetical protein LOTGIDRAFT_172080 [Lottia gigantea
9595083	gi|405951633|gb|EKC19530.1|	1.52994e-32	Calcipressin-1 [Crassostrea gigas]
9599891	gi|676488256|ref|XP_009064558.1|	1.53315e-57	hypothetical protein LOTGIDRAFT_236112 [Lottia gigantea
9651552	gi|405968283|gb|EKC33365.1|	1.5359e-50	Liver carboxylesterase 22 [Crassostrea gigas]
9570484	gi|405946057|gb|EKC17554.1|	1.54121e-68	Talin-2 [Crassostrea gigas]
9642148	gi|85687486|gb|ABC73693.1|	1.55786e-31	Toll receptor [Azumapecten farreri]
9631370	gi|405961841|gb|EKC27585.1|	1.57239e-90	Baculoviral IAP repeat-containing protein 7 [Crassostrea gigas
9584369	gi|383859690|ref|XP_003705325.1|	1.57813e-45	PREDICTED: protein numb-like isoform 1 [Megachile rotundata
9650400	gi|443732773|gb|ELU17364.1|	1.57964e-32	hypothetical protein CAPTEDRAFT_154290 [Capitella teleta
9622530	gi|431923345|gb|AGA94627.1|	1.58166e-71	insulin-related peptide receptor [Pinctada fucata
9649624	gi|524891182|ref|XP_005102127.1|	1.58285e-172	PREDICTED: carboxy-terminal kinesin 2-like isoform X2 [Aplysia californica
9643042	gi|405968945|gb|EKC33968.1|	1.59543e-18	Rho GTPase-activating protein 20 [Crassostrea gigas]
9653164	gi|641483972|gb|AIA66467.1|	1.5955e-174	toll2 [Hyriopsis cumingii

9634400	gi|524894036|ref|XP_005103525.1|	1.60081e-91	PREDICTED: mitochondrial folate transporter/carrier-like isoform X2 [Aplysia californica
9618502	gi|156360566|ref|XP_001625098.1|	1.60171e-91	predicted protein [Nematostella vectensis]
9648726	gi|524915526|ref|XP_005112560.1|	1.60805e-38	PREDICTED: protein Tob1-like isoform X1 [Aplysia californica]
9631358	gi|676489560|ref|XP_009064979.1|	1.60841e-127	hypothetical protein LOTGIDRAFT_179669 [Lottia gigantea
9634856	gi|524866641|ref|XP_005090139.1|	1.60855e-30	PREDICTED: uncharacterized protein LOC101853362 isoform X1 [Aplysia californica]
9623774	gi|405967412|gb|EKC32576.1|	1.60864e-58	RING finger protein 31 [Crassostrea gigas]
9591229	gi|405962198|gb|EKC27899.1|	1.62024e-49	Malignant fibrous histiocytoma-amplified sequence 1 [Crassostrea gigas
9635956	gi|405962937|gb|EKC28565.1|	1.62077e-158	Mitogen-activated protein kinase kinase kinase 13-B [Crassostrea gigas
9625162	gi|524910650|ref|XP_005110200.1|	1.62316e-146	PREDICTED: AP-1 complex subunit beta-1-like [Aplysia californica
9614376	gi|405966825|gb|EKC32062.1|	1.62421e-87	Hepatocyte growth factor-regulated tyrosine kinase substrate [Crassostrea gigas
9650786	gi|260825265|ref|XP_002607587.1|	1.62586e-11	hypothetical protein BRAFLDRAFT_71465 [Branchiostoma floridae]
9649440	gi|405963381|gb|EKC28958.1|	1.63038e-121	Cytoplasmic protein NCK2 [Crassostrea gigas]
9642764	gi|676482308|ref|XP_009062656.1|	1.63138e-100	hypothetical protein LOTGIDRAFT_128636, partial [Lottia gigantea]
9627954	gi|405951874|gb|EKC19747.1|	1.63319e-115	Serine/threonine-protein kinase 38-like protein [Crassostrea gigas
9652178	gi|563425681|gb|AHB59649.1|	1.63336e-165	activin receptor type II [Haliotis rufescens]
9644934	gi|405957436|gb|EKC23646.1|	1.63501e-104	Forkhead box protein O [Crassostrea gigas]
9650690	gi|405952358|gb|EKC20180.1|	1.64349e-40	Calpain-15 [Crassostrea gigas]
9625700	gi|93277256|gb|ABF06445.1|	1.64428e-61	AE-like protein [Doryteuthis pealeii
9642450	gi|405963184|gb|EKC28781.1|	1.64438e-12	hypothetical protein CGI_10018076 [Crassostrea gigas]
9572466	gi|676436391|ref|XP_009047867.1|	1.64964e-46	hypothetical protein LOTGIDRAFT_112005, partial [Lottia gigantea
9601326	gi|91083669|ref|XP_968061.1|	1.651e-70	PREDICTED: transmembrane 9 superfamily member 4 [Tribolium castaneum
9645110	gi|676474657|ref|XP_009060215.1|	1.65867e-96	hypothetical protein LOTGIDRAFT_229077 [Lottia gigantea
9628394	gi|405950190|gb|EKC18192.1|	1.66178e-123	Vinculin [Crassostrea gigas]
9645998	gi|405967368|gb|EKC32540.1|	1.67403e-54	80 kDa MCM3-associated protein [Crassostrea gigas]
9638284	gi|405952719|gb|EKC20497.1|	1.67537e-39	hypothetical protein CGI_10005970 [Crassostrea gigas]
9640470	gi|731513191|ref|XP_010599754.1|	1.68745e-32	PREDICTED: zinc finger protein 420-like isoform X3 [Loxodonta africana
9569946	gi|524870815|ref|XP_005092188.1|	1.68749e-11	PREDICTED: uncharacterized protein LOC101858164 [Aplysia californica]
9629492	gi|676463600|ref|XP_009056648.1|	1.69926e-147	hypothetical protein LOTGIDRAFT_120690 [Lottia gigantea]
9637234	gi|524900500|ref|XP_005106670.1|	1.7027e-158	PREDICTED: mannose-1-phosphate guanyltransferase beta-like isoform X1 [Aplysia californica]
9574396	gi|676448719|ref|XP_009051823.1|	1.7062e-27	hypothetical protein LOTGIDRAFT_53531, partial [Lottia gigantea]
9612732	gi|524886501|ref|XP_005099844.1|	1.71127e-110	PREDICTED: cytoplasmic dynein 1 heavy chain 1-like isoform X1 [Aplysia californica
9627258	gi|733910726|ref|XP_010718009.1|	1.71452e-85	PREDICTED: LOW QUALITY PROTEIN: poly(A)-specific ribonuclease PARN [Meleagris gallopavo
9601450	gi|524865865|ref|XP_005089758.1|	1.72845e-27	PREDICTED: heterogeneous nuclear ribonucleoprotein M-like isoform X1 [Aplysia californica
9641660	gi|524879091|ref|XP_005096225.1|	1.7284e-160	PREDICTED: E3 ubiquitin-protein ligase HERC2-like [Aplysia californica
9652380	gi|405966855|gb|EKC32090.1|	1.73112e-32	hypothetical protein CGI_10013456 [Crassostrea gigas]
9578030	gi|405976015|gb|EKC40539.1|	1.73233e-35	hypothetical protein CGI_10025593 [Crassostrea gigas]
9596319	gi|405958262|gb|EKC24407.1|	1.73274e-63	ATP-binding cassette sub-family B member 10, mitochondrial [Crassostrea gigas
9634434	gi|321456065|gb|EFX67182.1|	1.73429e-45	hypothetical protein DAPPUDRAFT_302185 [Daphnia pulex
9590993	gi|405960743|gb|EKC26631.1|	1.7351e-43	NEDD4-like E3 ubiquitin-protein ligase WWP1 [Crassostrea gigas]
9580020	gi|405966344|gb|EKC31640.1|	1.73532e-11	hypothetical protein CGI_10009152 [Crassostrea gigas]
9585317	gi|676445967|ref|XP_009050925.1|	1.73559e-23	hypothetical protein LOTGIDRAFT_231336 [Lottia gigantea
9600654	gi|676468340|ref|XP_009058178.1|	1.73838e-72	hypothetical protein LOTGIDRAFT_205708 [Lottia gigantea]
9647140	gi|443712986|gb|ELU06028.1|	1.74248e-97	hypothetical protein CAPTEDRAFT_168270 [Capitella teleta
9642624	gi|524883893|ref|XP_005098572.1|	1.74292e-78	PREDICTED: chondroitin sulfate synthase 1-like isoform X1 [Aplysia californica]
9643248	gi|260821125|ref|XP_002605884.1|	1.74299e-105	hypothetical protein BRAFLDRAFT_87444 [Branchiostoma floridae
9636860	gi|657557756|ref|XP_008283016.1|	1.74669e-22	PREDICTED: RNA-binding protein 26 [Stegastes partitus
9652756	gi|405952454|gb|EKC20264.1|	1.74863e-126	Epidermal growth factor receptor substrate 15-like 1 [Crassostrea gigas
9643674	gi|524912790|ref|XP_005111233.1|	1.75223e-52	PREDICTED: steroid 17-alpha-hydroxylase/17,20 lyase-like isoform X1 [Aplysia californica]
9620424	gi|405971270|gb|EKC36116.1|	1.76195e-48	Putative ferric-chelate reductase 1 [Crassostrea gigas]
9618534	gi|675859086|ref|XP_009015103.1|	1.76271e-106	hypothetical protein HELRODRAFT_98900 [Helobdella robusta]
9632360	gi|405950248|gb|EKC18247.1|	1.7832e-44	Activated CDC42 kinase 1 [Crassostrea gigas]
9619146	gi|405965007|gb|EKC30438.1|	1.78588e-122	DNA-directed RNA polymerase II subunit RPB1 [Crassostrea gigas
9649632	gi|405960504|gb|EKC26425.1|	1.78647e-107	Serine/threonine-protein kinase SIK2 [Crassostrea gigas]
9640520	gi|524895992|ref|XP_005104475.1|	1.79356e-167	PREDICTED: cleavage and polyadenylation specificity factor subunit 2-like [Aplysia californica
9606668	gi|443728139|gb|ELU14613.1|	1.7952e-52	hypothetical protein CAPTEDRAFT_180567 [Capitella teleta
9582965	gi|697002990|ref|XP_009567643.1|	1.79793e-14	PREDICTED: inter-alpha-trypsin inhibitor heavy chain H3-like [Cuculus canorus
9648718	gi|676455452|ref|XP_009054010.1|	1.80198e-98	hypothetical protein LOTGIDRAFT_160668 [Lottia gigantea
9595285	gi|45361293|ref|NP_989224.1|	1.80727e-33	mitochondrial trifunctional protein, alpha subunit [Xenopus (Silurana) tropicali
9625542	gi|405957834|gb|EKC24014.1|	1.80917e-52	hypothetical protein CGI_10014257 [Crassostrea gigas]
9643814	gi|405960038|gb|EKC25990.1|	1.81255e-73	Protein FAM60A [Crassostrea gigas]
9612088	gi|405957316|gb|EKC23537.1|	1.81493e-64	Protein rolling stone [Crassostrea gigas]
9650394	gi|524882362|ref|XP_005097825.1|	1.8198e-113	PREDICTED: rho GTPase-activating protein 35-like [Aplysia californica
9637074	gi|676441129|ref|XP_009049397.1|	1.82443e-19	hypothetical protein LOTGIDRAFT_230885 [Lottia gigantea]
9606992	gi|405953842|gb|EKC21423.1|	1.83033e-28	Circularly permutated Ras protein 1 [Crassostrea gigas
9642648	gi|676495692|ref|XP_009066970.1|	1.83642e-128	hypothetical protein LOTGIDRAFT_170085 [Lottia gigantea]
9649746	gi|524915919|ref|XP_005112746.1|	1.83659e-51	PREDICTED: serine/arginine repetitive matrix protein 2-like [Aplysia californica
9652096	gi|676488041|ref|XP_009064488.1|	1.84762e-129	hypothetical protein LOTGIDRAFT_168358 [Lottia gigantea]
9601112	gi|405967483|gb|EKC32638.1|	1.85528e-13	Cadherin EGF LAG seven-pass G-type receptor 3 [Crassostrea gigas]
9616540	gi|405958132|gb|EKC24290.1|	1.86131e-41	SH3 and PX domain-containing protein 2B [Crassostrea gigas
9607724	gi|405971038|gb|EKC35895.1|	1.86154e-12	Nuclear hormone receptor E75 [Crassostrea gigas]

9622880	gi|676495552|ref|XP_009066929.1|	1.86313e-136	hypothetical protein LOTGIDRAFT_134650 [Lottia gigantea]
9603070	gi|524900452|ref|XP_005106646.1|	1.8662e-72	PREDICTED: cysteine-rich with EGF-like domain protein 2-A-like isoform X1 [Aplysia californica
9636086	gi|405962051|gb|EKC27764.1|	1.86772e-146	Importin subunit alpha-3 [Crassostrea gigas
9650538	gi|675374257|gb|KFM67159.1|	1.8692e-97	Zinc finger homeobox protein 4, partial [Stegodyphus mimosarum
9648162	gi|162568970|gb|ABY19404.1|	1.86969e-36	PclA [Pasteuria ramosa]
9649042	gi|573890846|ref|XP_006633183.1|	1.87966e-74	PREDICTED: ankyrin repeat and sterile alpha motif domain-containing protein 1B-like [Lepisosteus oculatu
9640890	gi|321470189|gb|EFX81166.1|	1.88485e-63	hypothetical protein DAPPUDRAFT_102733 [Daphnia pulex
9645790	gi|676429519|ref|XP_009045626.1|	1.89078e-151	hypothetical protein LOTGIDRAFT_137063 [Lottia gigantea
9642244	gi|578896918|gb|AHI17298.1|	1.90162e-37	interleukine-1 receptor-associated kinase -a [Mytilus galloprovincialis
9641690	gi|405950330|gb|EKC18325.1|	1.90213e-175	Copper-transporting ATPase 1 [Crassostrea gigas]
9612206	gi|405962611|gb|EKC28269.1|	1.90448e-13	Leucine-rich repeat-containing protein 8D [Crassostrea gigas]
9645330	gi|676426856|ref|XP_009044758.1|	1.90501e-15	hypothetical protein LOTGIDRAFT_237326 [Lottia gigantea
9628908	gi|405969995|gb|EKC34935.1|	1.90855e-26	Choline-phosphate cytidylyltransferase B [Crassostrea gigas
9650880	gi|676429866|ref|XP_009045740.1|	1.91105e-177	hypothetical protein LOTGIDRAFT_224309 [Lottia gigantea
9615472	gi|676431094|ref|XP_009046138.1|	1.91398e-75	hypothetical protein LOTGIDRAFT_171701 [Lottia gigantea]
9644044	gi|676472988|ref|XP_009059669.1|	1.91521e-54	hypothetical protein LOTGIDRAFT_125059, partial [Lottia gigantea]
9584445	gi|568256755|gb|ETN65200.1|	1.91575e-67	hypothetical protein AND_003032 [Anopheles darling
9586709	gi|524877923|ref|XP_005095656.1|	1.91707e-57	PREDICTED: N-acetylglucosamine-1-phosphodiester alpha-N-acetylglucosaminidase-like isoform X2 [Aplysia californica
9621848	gi|405973316|gb|EKC38036.1|	1.91831e-13	Lysine-specific demethylase 5A [Crassostrea gigas]
9640940	gi|405968361|gb|EKC33438.1|	1.92205e-124	phosphatase Slingshot-like protein 2 [Crassostrea gigas
9647824	gi|405950899|gb|EKC18855.1|	1.9308e-163	Neuronal PAS domain-containing protein 4 [Crassostrea gigas
9647848	gi|405956446|gb|EKC23049.1|	1.93207e-54	hypothetical protein CGI_10000765 [Crassostrea gigas]
9646460	gi|676480629|ref|XP_009062122.1|	1.93451e-130	hypothetical protein LOTGIDRAFT_210561 [Lottia gigantea]
9637566	gi|405957485|gb|EKC23692.1|	1.93512e-125	Golgi-specific brefeldin A-resistance guanine nucleotide exchange factor 1 [Crassostrea gigas
9610832	gi|405966685|gb|EKC31939.1|	1.94724e-15	Poly [ADP-ribose] polymerase 12 [Crassostrea gigas]
9623900	gi|405973025|gb|EKC37762.1|	1.94827e-88	Coatomer subunit delta [Crassostrea gigas]
9646204	gi|676428881|ref|XP_009045419.1|	1.95021e-151	hypothetical protein LOTGIDRAFT_185322 [Lottia gigantea]
9636390	gi|405950935|gb|EKC18889.1|	1.95071e-135	Wee1-like protein kinase [Crassostrea gigas]
9652678	gi|405950780|gb|EKC18744.1|	1.95428e-130	Forkhead box protein N3 [Crassostrea gigas]
9642454	gi|676490897|ref|XP_009065419.1|	1.95495e-106	hypothetical protein LOTGIDRAFT_168884 [Lottia gigantea]
9639286	gi|524872372|ref|XP_005092948.1|	1.9589e-22	PREDICTED: RING-H2 finger protein ATL3-like [Aplysia californica
9643636	gi|449272743|gb|EMC82497.1|	1.95992e-11	hypothetical protein A306_09513, partial [Columba livia
9626370	gi|405958647|gb|EKC24756.1|	1.96108e-102	Ectoine hydroxylase [Crassostrea gigas]
9569500	gi|524864538|ref|XP_005089104.1|	1.96127e-68	PREDICTED: tyrosine-protein kinase CSK-like [Aplysia californica
9644572	gi|443692967|gb|ELT94444.1|	1.96839e-70	hypothetical protein CAPTEDRAFT_171777 [Capitella teleta
9652450	gi|524911398|ref|XP_005110563.1|	1.97227e-100	PREDICTED: endonuclease/exonuclease/phosphatase family domain-containing protein 1-like [Aplysia californic
9586901	gi|676420817|ref|XP_009043729.1|	1.97467e-70	hypothetical protein LOTGIDRAFT_227857 [Lottia gigantea
9618762	gi|524865579|ref|XP_005089618.1|	1.97544e-89	PREDICTED: ADP-ribosylation factor-like protein 5B-like isoform X1 [Aplysia californica]
9634068	gi|405960073|gb|EKC26023.1|	1.97636e-134	Peroxisome assembly factor 2 [Crassostrea gigas]
9638580	gi|405975249|gb|EKC39830.1|	1.97676e-104	Disco-interacting protein 2-like protein C [Crassostrea gigas]
9603094	gi|405950190|gb|EKC18192.1|	1.98004e-98	Vinculin [Crassostrea gigas]
9641776	gi|405970874|gb|EKC35741.1|	1.98027e-164	Protein kinase C iota type [Crassostrea gigas]
9641892	gi|405961102|gb|EKC26956.1|	1.98213e-71	Alpha-1,6-mannosyl-glycoprotein 2-beta-N-acetylglucosaminyltransferase [Crassostrea gigas
9633960	gi|405975684|gb|EKC40234.1|	1.98433e-102	HEAT repeat-containing protein 7A [Crassostrea gigas]
9585645	gi|91093137|ref|XP_969665.1|	1.99038e-11	PREDICTED: breast cancer anti-estrogen resistance protein 1 isoform X1 [Tribolium castaneum
9651648	gi|405964783|gb|EKC30230.1|	1.99142e-29	Cyclin-T2 [Crassostrea gigas]
9633504	gi|585678960|ref|XP_006819429.1|	1.99275e-14	PREDICTED: nuclear factor erythroid 2-related factor 1-like isoform X2 [Saccoglossus kowalevskii
9636808	gi|405972624|gb|EKC37384.1|	1.99729e-119	PAP-associated domain-containing protein 5 [Crassostrea gigas
9638882	gi|676458562|ref|XP_009055016.1|	1.99768e-112	hypothetical protein LOTGIDRAFT_215641 [Lottia gigantea]
9649470	gi|524872858|ref|XP_005093184.1|	2.00131e-119	PREDICTED: ankyrin repeat and IBR domain-containing protein 1-like [Aplysia californica
9644958	gi|585675225|ref|XP_006818739.1|	2.00331e-94	PREDICTED: stromal interaction molecule 1-like [Saccoglossus kowalevski
9635372	gi|632933446|gb|AHZ44497.1|	2.00516e-145	transforming growth factor activated kinase-1 [Mytilus galloprovincialis
9607726	gi|405977917|gb|EKC42344.1|	2.00633e-99	Peroxisomal multifunctional enzyme type 2 [Crassostrea gigas
9622410	gi|405960400|gb|EKC26326.1|	2.01011e-50	BTB/POZ domain-containing protein 7 [Crassostrea gigas
9573316	gi|443712494|gb|ELU05786.1|	2.01655e-44	hypothetical protein CAPTEDRAFT_221385, partial [Capitella teleta
9583537	gi|576701272|gb|EUB64791.1|	2.02466e-29	Tripartite motif-containing protein [Echinococcus granulosus
9636820	gi|676469874|ref|XP_009058673.1|	2.02998e-63	hypothetical protein LOTGIDRAFT_217780, partial [Lottia gigantea]
9644586	gi|676495131|ref|XP_009066789.1|	2.03225e-75	hypothetical protein LOTGIDRAFT_223226 [Lottia gigantea
9643420	gi|405971020|gb|EKC35878.1|	2.0358e-53	Sodium-dependent glucose transporter 1 [Crassostrea gigas
9632064	gi|443688390|gb|ELT91094.1|	2.03777e-101	hypothetical protein CAPTEDRAFT_227278 [Capitella teleta
9628586	gi|405951405|gb|EKC19320.1|	2.04095e-12	hypothetical protein CGI_10008806 [Crassostrea gigas]
9651000	gi|405959192|gb|EKC25253.1|	2.04619e-110	Forkhead box protein P1 [Crassostrea gigas]
9631014	gi|233142080|gb|ACQ91095.1|	2.04851e-58	complement factor B-like protein [Ruditapes decussatus
9641244	gi|405962873|gb|EKC28512.1|	2.05631e-174	Filamin-C [Crassostrea gigas]
9650984	gi|405974827|gb|EKC39440.1|	2.05695e-86	A disintegrin and metalloproteinase with thrombospondin motifs 2 [Crassostrea giga
9602002	gi|443712494|gb|ELU05786.1|	2.06176e-44	hypothetical protein CAPTEDRAFT_221385, partial [Capitella teleta
9630266	gi|676462533|ref|XP_009056299.1|	2.06672e-39	hypothetical protein LOTGIDRAFT_232832 [Lottia gigantea
9639962	gi|405960113|gb|EKC26060.1|	2.07467e-144	Ubiquitin carboxyl-terminal hydrolase 15 [Crassostrea gigas
9611108	gi|676496714|ref|XP_009067305.1|	2.07475e-65	hypothetical protein LOTGIDRAFT_223620 [Lottia gigantea]
9586765	gi|366999322|ref|XP_003684397.1|	2.07691e-11	hypothetical protein TPHA_0B02910 [Tetrapisispora phaffii CBS 4417

9651624	gi|405963516|gb|EKC29081.1|	2.08062e-87	Ras-responsive element-binding protein 1, partial [Crassostrea gigas
9632130	gi|405966142|gb|EKC31460.1|	2.08773e-37	TBC1 domain family member 4 [Crassostrea gigas
9633806	gi|676427774|ref|XP_009045057.1|	2.08944e-136	hypothetical protein LOTGIDRAFT_136505 [Lottia gigantea]
9614482	gi|676430487|ref|XP_009045942.1|	2.08988e-58	hypothetical protein LOTGIDRAFT_237715 [Lottia gigantea
9586435	gi|405977912|gb|EKC42339.1|	2.0899e-32	Proton-coupled folate transporter [Crassostrea gigas]
9637358	gi|405952290|gb|EKC20120.1|	2.09214e-39	Armadillo repeat protein deleted in velo-cardio-facial syndrome-like protein [Crassostrea gigas
9649306	gi|524864042|ref|XP_005088859.1|	2.09855e-20	PREDICTED: kelch-like protein 20-like [Aplysia californica
9596601	gi|524869257|ref|XP_005091424.1|	2.09857e-93	PREDICTED: YTH domain family protein 1-like [Aplysia californica
9624624	gi|405967758|gb|EKC32889.1|	2.09982e-13	hypothetical protein CGI_10024451 [Crassostrea gigas]
9640106	gi|405966964|gb|EKC32184.1|	2.10119e-128	Putative oxidoreductase yteT [Crassostrea gigas]
9638806	gi|443696892|gb|ELT97507.1|	2.1019e-37	hypothetical protein CAPTEDRAFT_221464 [Capitella teleta
9633698	gi|524878007|ref|XP_005095696.1|	2.10263e-96	PREDICTED: transcription factor Sp4-like [Aplysia californica
9634986	gi|405969382|gb|EKC34356.1|	2.10661e-38	Nuclear receptor coactivator 4 [Crassostrea gigas]
9649394	gi|405962502|gb|EKC28171.1|	2.10691e-28	hypothetical protein CGI_10014339 [Crassostrea gigas]
9635970	gi|676494045|ref|XP_009066434.1|	2.10814e-57	hypothetical protein LOTGIDRAFT_108839 [Lottia gigantea]
9648774	gi|405951037|gb|EKC18984.1|	2.10862e-147	Calsyntenin-1 [Crassostrea gigas]
9632666	gi|405974709|gb|EKC39334.1|	2.12231e-29	Body wall muscle protein HR-29 [Crassostrea gigas
9634204	gi|405953727|gb|EKC21332.1|	2.12261e-136	Protein FAM46A [Crassostrea gigas]
9633416	gi|405977349|gb|EKC41806.1|	2.12299e-141	E3 ubiquitin-protein ligase NEDD4 [Crassostrea gigas
9648606	gi|405967974|gb|EKC33083.1|	2.12437e-67	Protein regulator of cytokinesis 1 [Crassostrea gigas]
9633196	gi|405971234|gb|EKC36081.1|	2.12465e-101	Metastasis suppressor protein 1 [Crassostrea gigas]
9570184	gi|443692103|gb|ELT93776.1|	2.12974e-14	hypothetical protein CAPTEDRAFT_190955 [Capitella teleta
9638568	gi|405961919|gb|EKC27652.1|	2.13167e-74	Oxysterol-binding protein-related protein 6 [Crassostrea gigas
9621562	gi|390334560|ref|XP_790502.3|	2.13201e-81	PREDICTED: protein SDA1 homolog [Strongylocentrotus purpuratus
9586627	gi|405950549|gb|EKC18530.1|	2.13437e-32	GRAM domain-containing protein 1B [Crassostrea gigas
9645072	gi|405953791|gb|EKC21383.1|	2.13607e-19	Dual specificity tyrosine-phosphorylation-regulated kinase 1A [Crassostrea gigas
9613296	gi|524879283|ref|XP_005096319.1|	2.13857e-68	PREDICTED: partitioning defective 6 homolog gamma-like isoform X2 [Aplysia californica
9650104	gi|524874689|ref|XP_005094079.1|	2.14073e-119	PREDICTED: uncharacterized protein LOC101851335 isoform X1 [Aplysia californica]
9649640	gi|405977235|gb|EKC41694.1|	2.14366e-169	Polycomb protein SCMH1 [Crassostrea gigas]
9621826	gi|676493255|ref|XP_009066181.1|	2.14451e-89	hypothetical protein LOTGIDRAFT_133766 [Lottia gigantea]
9636950	gi|584074659|ref|XP_006757563.1|	2.15014e-12	PREDICTED: zinc finger protein 154 [Myotis davidii
9621446	gi|405951407|gb|EKC19322.1|	2.15189e-49	Putative Bcl-2-like protein antagonist/killer 2 [Crassostrea gigas
9592601	gi|524911574|ref|XP_005110648.1|	2.1556e-60	PREDICTED: zinc finger protein ZPR1-like [Aplysia californica
9649126	gi|405957288|gb|EKC23511.1|	2.16457e-70	hypothetical protein CGI_10006130 [Crassostrea gigas]
9620984	gi|676487578|ref|XP_009064342.1|	2.16509e-86	hypothetical protein LOTGIDRAFT_196190 [Lottia gigantea
9645084	gi|405971352|gb|EKC36193.1|	2.17133e-88	Disks large-like protein 1 [Crassostrea gigas]
9649360	gi|405975951|gb|EKC40479.1|	2.17623e-17	Zinc finger FYVE domain-containing protein 9 [Crassostrea gigas
9616486	gi|524886831|ref|XP_005100007.1|	2.17839e-74	PREDICTED: uncharacterized protein LOC101855261 [Aplysia californica]
9632928	gi|405953770|gb|EKC21366.1|	2.17982e-90	hypothetical protein CGI_10004021 [Crassostrea gigas]
9597415	gi|405966788|gb|EKC32027.1|	2.18251e-69	hypothetical protein CGI_10004788 [Crassostrea gigas]
9646728	gi|405959418|gb|EKC25460.1|	2.18444e-121	Zinc finger homeobox protein 3 [Crassostrea gigas
9636480	gi|405970492|gb|EKC35390.1|	2.19236e-94	OTU domain-containing protein 5-B [Crassostrea gigas
9648518	gi|405951892|gb|EKC19763.1|	2.19252e-149	hypothetical protein CGI_10007665 [Crassostrea gigas]
9615814	gi|405973144|gb|EKC37874.1|	2.19601e-95	Importin subunit beta-1 [Crassostrea gigas
9607056	gi|405950151|gb|EKC18154.1|	2.20651e-74	Kelch-like ECH-associated protein 1 [Crassostrea gigas]
9631034	gi|405964922|gb|EKC30361.1|	2.20904e-28	Protein sidekick [Crassostrea gigas]
9620510	gi|91087863|ref|XP_968982.1|	2.21342e-67	PREDICTED: phosphatidylinositol transfer protein alpha isoform isoform X1 [Tribolium castaneum
9572416	gi|405957387|gb|EKC23601.1|	2.21409e-67	Delta-aminolevulinic acid dehydratase [Crassostrea gigas
9603888	gi|405976869|gb|EKC41348.1|	2.2193e-28	GTP-binding protein GEM [Crassostrea gigas
9614398	gi|405973624|gb|EKC38326.1|	2.2208e-44	Muscle M-line assembly protein unc-89 [Crassostrea gigas
9631652	gi|405958250|gb|EKC24395.1|	2.22491e-84	E3 ubiquitin-protein ligase MIB2 [Crassostrea gigas
9597069	gi|676465868|ref|XP_009057383.1|	2.23303e-50	hypothetical protein LOTGIDRAFT_233329 [Lottia gigantea]
9651288	gi|405959772|gb|EKC25766.1|	2.23557e-115	Fibroblast growth factor receptor-like 1 [Crassostrea gigas]
9650390	gi|405963423|gb|EKC28997.1|	2.24009e-87	Williams-Beuren syndrome chromosomal region 14 protein [Crassostrea gigas
9584597	gi|524895425|ref|XP_005104195.1|	2.26301e-68	PREDICTED: copine-3-like isoform X1 [Aplysia californica]
9581919	gi|67084071|gb|AAY66970.1|	2.27837e-46	secreted protein [Ixodes scapularis]
9639050	gi|405976812|gb|EKC41296.1|	2.28381e-140	SWI/SNF-related matrix-associated actin-dependent regulator of chromatin subfamily A member 5 [Crassostrea gigas
9607000	gi|405950583|gb|EKC18561.1|	2.28868e-63	Fermitin family-like protein 2 [Crassostrea gigas
9621180	gi|405971038|gb|EKC35895.1|	2.29085e-35	Nuclear hormone receptor E75 [Crassostrea gigas]
9637150	gi|405971243|gb|EKC36089.1|	2.29191e-38	transport protein Sec31A [Crassostrea gigas]
9625954	gi|154551045|gb|ABS83556.1|	2.29477e-87	ABCB/p-glycoprotein-like protein [Mytilus californianus
9593083	gi|405977099|gb|EKC41565.1|	2.29772e-70	hypothetical protein CGI_10022116 [Crassostrea gigas]
9647836	gi|405973063|gb|EKC37798.1|	2.29796e-23	hypothetical protein CGI_10017558 [Crassostrea gigas]
9625848	gi|405972681|gb|EKC37437.1|	2.29973e-55	hypothetical protein CGI_10019335 [Crassostrea gigas]
9647522	gi|405954309|gb|EKC21787.1|	2.30241e-153	Constitutive coactivator of PPAR-gamma-like protein 1-like protein [Crassostrea gigas
9640892	gi|405971427|gb|EKC36266.1|	2.30407e-61	Follistatin-A [Crassostrea gigas]
9632180	gi|405973540|gb|EKC38248.1|	2.31898e-20	Metal transporter CNNM2 [Crassostrea gigas]
9642296	gi|405976869|gb|EKC41348.1|	2.32753e-43	GTP-binding protein GEM [Crassostrea gigas
9648026	gi|405975449|gb|EKC40013.1|	2.33599e-148	Hemicentin-1 [Crassostrea gigas]
9627200	gi|405962952|gb|EKC28577.1|	2.33884e-50	hypothetical protein CGI_10011784 [Crassostrea gigas]

9635562	gi|405965593|gb|EKC30956.1|	2.34405e-101	E3 ubiquitin-protein ligase HECTD1 [Crassostrea gigas
9576488	gi|133505845|ref|NP_035331.3|	2.35752e-45	tyrosine-protein phosphatase non-receptor type 1 [Mus musculus
9610500	gi|734546053|gb|KHN74625.1|	2.36384e-37	Collagen alpha-2(IV) chain [Toxocara canis
9631068	gi|405961082|gb|EKC26936.1|	2.36435e-139	Cell division protein kinase 9 [Crassostrea gigas
9584625	gi|307197051|gb|EFN78423.1|	2.36916e-25	hypothetical protein EAI_13692 [Harpegnathos saltator
9596829	gi|524879355|ref|XP_005096354.1|	2.36924e-76	PREDICTED: NEDD8-activating enzyme E1 regulatory subunit-like [Aplysia californica
9651354	gi|405964149|gb|EKC29666.1|	2.37285e-173	Putative nuclear hormone receptor HR3 [Crassostrea gigas]
9608744	gi|405962873|gb|EKC28512.1|	2.37401e-103	Filamin-C [Crassostrea gigas]
9652998	gi|405957301|gb|EKC23523.1|	2.38467e-84	Nucleolar pre-ribosomal-associated protein 1 [Crassostrea gigas]
9648304	gi|405951392|gb|EKC19308.1|	2.38516e-140	E3 ubiquitin-protein ligase RNF19A [Crassostrea gigas
9639228	gi|524894514|ref|XP_005103754.1|	2.38885e-129	PREDICTED: ubiquitin-conjugating enzyme E2 Q1-like [Aplysia californica
9630400	gi|524871619|ref|XP_005092581.1|	2.39011e-19	PREDICTED: trithorax group protein osa-like [Aplysia californica
9610166	gi|260815098|ref|XP_002602311.1|	2.39082e-74	hypothetical protein BRAFLDRAFT_127318 [Branchiostoma floridae]
9640534	gi|405975054|gb|EKC39650.1|	2.39187e-95	transport protein Sec24B [Crassostrea gigas]
9612164	gi|405978120|gb|EKC42534.1|	2.39246e-32	Putative helicase with zinc finger domain [Crassostrea gigas
9642218	gi|405962918|gb|EKC28548.1|	2.3956e-149	Y amino acid transporter 2 [Crassostrea gigas]
9648660	gi|676450929|ref|XP_009052539.1|	2.39658e-135	hypothetical protein LOTGIDRAFT_143634 [Lottia gigantea]
9631218	gi|676493826|ref|XP_009066363.1|	2.40051e-107	hypothetical protein LOTGIDRAFT_223151 [Lottia gigantea]
9648854	gi|405957661|gb|EKC23858.1|	2.40124e-90	Large proline-rich protein BAT3 [Crassostrea gigas]
9582839	gi|83318391|gb|AAI08485.1|	2.41313e-35	TCERG1 protein [Xenopus laevis]
9649836	gi|405949988|gb|EKC17997.1|	2.41638e-67	strawberry notch-like protein 1 [Crassostrea gigas]
9618236	gi|405965865|gb|EKC31214.1|	2.42211e-15	Proteasome subunit beta type-8 [Crassostrea gigas]
9618240	gi|242022816|ref|XP_002431834.1|	2.42641e-124	guanine nucleotide-binding protein G, putative [Pediculus humanus corpor
9603694	gi|573893895|ref|XP_006634699.1|	2.42663e-19	PREDICTED: putative ferric-chelate reductase 1-like [Lepisosteus oculatus
9631592	gi|405971408|gb|EKC36247.1|	2.42725e-14	Aftiphilin [Crassostrea gigas]
9621200	gi|405956277|gb|EKC22996.1|	2.43059e-61	Nuclear distribution protein nudE-like protein 1 [Crassostrea gigas
9635696	gi|405959219|gb|EKC25276.1|	2.43201e-106	Mitogen-activated protein kinase kinase kinase kinase 3, partial [Crassostrea gigas
9651546	gi|405955429|gb|EKC22551.1|	2.44643e-56	Max-binding protein MNT [Crassostrea gigas]
9644076	gi|524868914|ref|XP_005091255.1|	2.45185e-134	PREDICTED: protein phosphatase 1 regulatory inhibitor subunit 16B-like isoform X5 [Aplysia californic
9652488	gi|405953548|gb|EKC21189.1|	2.45454e-58	Nuclear receptor corepressor 1 [Crassostrea gigas]
9623460	gi|676488858|ref|XP_009064751.1|	2.46696e-27	hypothetical protein LOTGIDRAFT_236217 [Lottia gigantea
9600116	gi|524901669|ref|XP_005107239.1|	2.46757e-79	PREDICTED: hypoxia up-regulated protein 1-like [Aplysia californica
9644020	gi|405952826|gb|EKC20589.1|	2.4725e-94	PR domain zinc finger protein 16 [Crassostrea gigas
9631798	gi|676480483|ref|XP_009062074.1|	2.47415e-83	hypothetical protein LOTGIDRAFT_107273 [Lottia gigantea]
9633738	gi|576703312|gb|AHH32929.1|	2.48376e-97	myostatin [Sinonovacula constricta
9601768	gi|573906081|ref|XP_006640761.1|	2.4855e-90	PREDICTED: 78 kDa glucose-regulated protein-like [Lepisosteus oculatus
9583021	gi|676449479|ref|XP_009052067.1|	2.503e-24	hypothetical protein LOTGIDRAFT_115014 [Lottia gigantea]
9604234	gi|524879203|ref|XP_005096279.1|	2.50411e-72	PREDICTED: protein DD3-3-like [Aplysia californica]
9639766	gi|405977199|gb|EKC41661.1|	2.51111e-33	capicua-like protein [Crassostrea gigas]
9590935	gi|195434699|ref|XP_002065340.1|	2.51712e-26	GK15396 [Drosophila willistoni] >gi|194161425|gb|EDW76326.1| GK15396 [Drosophila williston
9633822	gi|405963090|gb|EKC28694.1|	2.5261e-57	AP-1 complex subunit gamma-1 [Crassostrea gigas
9590465	gi|676445955|ref|XP_009050921.1|	2.5262e-63	hypothetical protein LOTGIDRAFT_231331 [Lottia gigantea]
9640420	gi|676429718|ref|XP_009045692.1|	2.52779e-121	hypothetical protein LOTGIDRAFT_137203, partial [Lottia gigantea
9652062	gi|602698599|ref|XP_007461323.1|	2.54026e-38	PREDICTED: nuclear pore complex protein Nup153 isoform X1 [Lipotes vexillifer
9651930	gi|405955290|gb|EKC22460.1|	2.54393e-160	Follistatin-related protein 5 [Crassostrea gigas
9589923	gi|405977593|gb|EKC42036.1|	2.55566e-23	Phosphoinositide 3-kinase adapter protein 1 [Crassostrea gigas
9624194	gi|676447846|ref|XP_009051542.1|	2.55786e-96	hypothetical protein LOTGIDRAFT_152778, partial [Lottia gigantea]
9602282	gi|676454949|ref|XP_009053847.1|	2.56227e-34	hypothetical protein LOTGIDRAFT_232036 [Lottia gigantea
9613416	gi|685835123|emb|CEF70044.1|	2.57035e-45	Collagen alpha-1(IV) chain [Strongyloides ratt
9653364	gi|493211562|ref|WP_006196496.1|	2.57903e-123	hypothetical protein [Nodularia spumigena
9643406	gi|405968820|gb|EKC33849.1|	2.57987e-157	Cysteine sulfinic acid decarboxylase [Crassostrea gigas]
9602956	gi|676494786|ref|XP_009066677.1|	2.58034e-58	hypothetical protein LOTGIDRAFT_108875 [Lottia gigantea
9620924	gi|405958629|gb|EKC24738.1|	2.58138e-55	Lysine-specific histone demethylase 1 [Crassostrea gigas]
9611616	gi|405957831|gb|EKC24011.1|	2.5823e-39	DnaJ-like protein subfamily C member 22 [Crassostrea gigas
9636722	gi|585684442|ref|XP_006812581.1|	2.58568e-18	PREDICTED: tyrosine-protein phosphatase non-receptor type 13-like [Saccoglossus kowalevski
9612802	gi|676458651|ref|XP_009055045.1|	2.58699e-66	hypothetical protein LOTGIDRAFT_161404 [Lottia gigantea]
9643300	gi|405957186|gb|EKC23416.1|	2.59251e-117	RNA-binding protein Nova-1 [Crassostrea gigas]
9648704	gi|524897891|ref|XP_005105401.1|	2.60206e-97	PREDICTED: microtubule-associated serine/threonine-protein kinase 2-like [Aplysia californica
9632808	gi|405978814|gb|EKC43175.1|	2.60271e-35	SLAIN motif-containing protein 2 [Crassostrea gigas
9647348	gi|405974083|gb|EKC38753.1|	2.60333e-40	Latrophilin-3 [Crassostrea gigas]
9603938	gi|676494997|ref|XP_009066746.1|	2.6034e-53	hypothetical protein LOTGIDRAFT_197880 [Lottia gigantea] >gi|556093902|gb|ESO82555.1| hypothetical protein LOTGIDRAFT_197880 [Lottia gigante
9649040	gi|405962714|gb|EKC28363.1|	2.60466e-100	Cysteine/serine-rich nuclear protein 3 [Crassostrea gigas]
9569102	gi|556955671|ref|XP_005989263.1|	2.6101e-12	PREDICTED: breast cancer metastasis-suppressor 1-like protein [Latimeria chalumnae
9647470	gi|676429522|ref|XP_009045627.1|	2.61697e-48	hypothetical protein LOTGIDRAFT_171182 [Lottia gigantea]
9635400	gi|675883162|ref|XP_009027083.1|	2.62632e-20	hypothetical protein HELRODRAFT_87621, partial [Helobdella robusta]
9615662	gi|676494474|ref|XP_009066575.1|	2.62916e-16	hypothetical protein LOTGIDRAFT_229805 [Lottia gigantea
9639150	gi|405958108|gb|EKC24268.1|	2.63013e-128	Metalloreductase STEAP2 [Crassostrea gigas]
9588963	gi|405962519|gb|EKC28185.1|	2.63157e-17	Tripartite motif-containing protein 2 [Crassostrea gigas]
9649240	gi|501299315|dbj|BAN21488.1|	2.63179e-23	conserved hypothetical protein, partial [Riptortus pedestris
9600644	gi|405963605|gb|EKC29167.1|	2.64019e-12	hypothetical protein CGI_10024290 [Crassostrea gigas]

9636514	gi|405951872|gb|EKC19745.1|	2.64438e-23	Nuclear receptor ROR-gamma [Crassostrea gigas]
9652796	gi|405968869|gb|EKC33898.1|	2.64937e-130	Rho GTPase-activating protein 21 [Crassostrea gigas]
9621284	gi|443719184|gb|ELU09458.1|	2.6749e-76	hypothetical protein CAPTEDRAFT_21383 [Capitella teleta
9651358	gi|405957301|gb|EKC23523.1|	2.67883e-100	Nucleolar pre-ribosomal-associated protein 1 [Crassostrea gigas]
9649106	gi|676432840|ref|XP_009046705.1|	2.68208e-129	hypothetical protein LOTGIDRAFT_62027, partial [Lottia gigantea]
9617734	gi|405975403|gb|EKC39969.1|	2.68371e-59	Ubiquitin-conjugating enzyme E2 Q2 [Crassostrea gigas
9585221	gi|641789842|ref|XP_008176691.1|	2.68384e-16	PREDICTED: integrin beta-6 isoform X2 [Chrysemys picta belli
9647808	gi|405969343|gb|EKC34319.1|	2.68944e-56	Glucocorticoid-induced transcript 1 protein [Crassostrea gigas]
9648548	gi|405951802|gb|EKC19683.1|	2.69383e-38	MKL/myocardin-like protein 2 [Crassostrea gigas]
9607828	gi|676449052|ref|XP_009051930.1|	2.69572e-43	hypothetical protein LOTGIDRAFT_214161 [Lottia gigantea]
9645694	gi|676470191|ref|XP_009058773.1|	2.71468e-150	hypothetical protein LOTGIDRAFT_123182, partial [Lottia gigantea
9624716	gi|405966044|gb|EKC31369.1|	2.72975e-29	Collagen alpha-1(XII) chain [Crassostrea gigas
9619246	gi|161110488|gb|ABX57736.1|	2.72978e-70	TFG beta signaling pathway factor [Pinctada fucata
9653292	gi|405962019|gb|EKC27734.1|	2.74281e-135	Vitamin D3 receptor B [Crassostrea gigas]
9638426	gi|443694236|gb|ELT95429.1|	2.74728e-90	hypothetical protein CAPTEDRAFT_160825 [Capitella teleta
9624778	gi|524912061|ref|XP_005110884.1|	2.76009e-106	PREDICTED: ral GTPase-activating protein subunit beta-like isoform X2 [Aplysia californica
9600440	gi|405964907|gb|EKC30346.1|	2.76645e-30	Mediator of RNA polymerase II transcription subunit 26 [Crassostrea gigas
9582191	gi|676454834|ref|XP_009053810.1|	2.7778e-44	hypothetical protein LOTGIDRAFT_232003 [Lottia gigantea
9636852	gi|405969278|gb|EKC34259.1|	2.79358e-129	Putative ATP-dependent RNA helicase an3 [Crassostrea gigas
9594119	gi|405953450|gb|EKC21110.1|	2.79401e-23	Nuclear factor interleukin-3-regulated protein [Crassostrea gigas
9611650	gi|405967390|gb|EKC32555.1|	2.79574e-33	Protein SPEC3 [Crassostrea gigas]
9634484	gi|522214818|ref|WP_020721930.1|	2.79612e-27	hypothetical protein [Acidobacteriaceae bacterium KBS 96
9632466	gi|348591352|emb|CAX46411.2|	2.80107e-98	ABCB/P-glycoprotein-like protein [Mytilus galloprovincialis
9618360	gi|524880771|ref|XP_005097044.1|	2.80205e-88	PREDICTED: palmitoyltransferase ZDHHC7-like [Aplysia californica
9640272	gi|443734840|gb|ELU18696.1|	2.80504e-80	hypothetical protein CAPTEDRAFT_147210 [Capitella teleta
9640788	gi|675372583|gb|KFM65485.1|	2.80566e-59	Protein white, partial [Stegodyphus mimosarum
9632006	gi|405950619|gb|EKC18595.1|	2.8057e-34	RNA-binding protein Raly [Crassostrea gigas
9574904	gi|405964786|gb|EKC30232.1|	2.81237e-30	F-box/WD repeat-containing protein 9 [Crassostrea gigas]
9636678	gi|443733929|gb|ELU18108.1|	2.8193e-65	hypothetical protein CAPTEDRAFT_219441 [Capitella teleta
9651420	gi|405976456|gb|EKC40962.1|	2.82093e-174	Zinc finger MIZ domain-containing protein 1 [Crassostrea gigas
9642556	gi|676479573|ref|XP_009061787.1|	2.82325e-129	hypothetical protein LOTGIDRAFT_127745 [Lottia gigantea]
9652358	gi|676429147|ref|XP_009045505.1|	2.82397e-35	hypothetical protein LOTGIDRAFT_110160 [Lottia gigantea
9642622	gi|405959211|gb|EKC25270.1|	2.84636e-113	Enhancer of mRNA-decapping protein 3 [Crassostrea gigas
9653482	gi|676448280|ref|XP_009051681.1|	2.84789e-52	hypothetical protein LOTGIDRAFT_152942 [Lottia gigantea]
9604654	gi|405971818|gb|EKC36629.1|	2.85086e-76	Myosin-Id [Crassostrea gigas]
9637266	gi|405957589|gb|EKC23790.1|	2.85252e-105	Active breakpoint cluster region-related protein [Crassostrea gigas
9606406	gi|405950771|gb|EKC18736.1|	2.85663e-18	E3 ubiquitin-protein ligase TRIM33 [Crassostrea gigas
9630038	gi|405974458|gb|EKC39101.1|	2.87961e-25	E3 ubiquitin-protein ligase MIB2 [Crassostrea gigas
9652782	gi|405950029|gb|EKC18037.1|	2.88557e-161	hypothetical protein CGI_10016161 [Crassostrea gigas]
9649284	gi|524865134|ref|XP_005089399.1|	2.88686e-179	PREDICTED: WD repeat-containing protein 11-like [Aplysia californica
9611778	gi|676436230|ref|XP_009047814.1|	2.89314e-30	hypothetical protein LOTGIDRAFT_200320 [Lottia gigantea]
9652820	gi|524898093|ref|XP_005105500.1|	2.89655e-84	PREDICTED: uncharacterized protein LOC101856598 isoform X1 [Aplysia californica]
9585961	gi|529009643|ref|XP_005226003.1|	2.896e-58	PREDICTED: N(4)-(beta-N-acetylglucosaminyl)-L-asparaginase isoform X2 [Bos taurus]
9651136	gi|405950801|gb|EKC18764.1|	2.91121e-144	La-related protein 1 [Crassostrea gigas]
9573218	gi|524906986|ref|XP_005108636.1|	2.91865e-35	PREDICTED: MAGUK p55 subfamily member 2-like [Aplysia californica
9596041	gi|443732701|gb|ELU17328.1|	2.93425e-31	hypothetical protein CAPTEDRAFT_219555 [Capitella teleta
9594901	gi|405973235|gb|EKC37959.1|	2.93526e-28	Protocadherin Fat 1 [Crassostrea gigas]
9579786	gi|405976142|gb|EKC40661.1|	2.93948e-35	ATP-binding cassette sub-family A member 5 [Crassostrea gigas
9613714	gi|676474873|ref|XP_009060285.1|	2.94086e-41	hypothetical protein LOTGIDRAFT_106056, partial [Lottia gigantea]
9645958	gi|676436494|ref|XP_009047901.1|	2.94664e-51	hypothetical protein LOTGIDRAFT_157441 [Lottia gigantea]
9585037	gi|405959257|gb|EKC25312.1|	2.95558e-68	Sodium- and chloride-dependent glycine transporter 2 [Crassostrea gigas
9639914	gi|405959608|gb|EKC25623.1|	2.963e-167	Importin subunit alpha-7 [Crassostrea gigas
9623218	gi|405968127|gb|EKC33227.1|	2.9717e-49	Neurofibromin [Crassostrea gigas]
9621668	gi|405970915|gb|EKC35778.1|	2.97857e-107	Serine/threonine-protein phosphatase 2A 55 kDa regulatory subunit B alpha isoform [Crassostrea gigas
9641590	gi|405962370|gb|EKC28059.1|	2.98117e-96	hypothetical protein CGI_10012981 [Crassostrea gigas]
9649396	gi|405970743|gb|EKC35619.1|	2.98645e-91	Krueppel-like factor 15 [Crassostrea gigas]
9635710	gi|405952903|gb|EKC20658.1|	2.98675e-88	Putative guanine nucleotide exchange factor MCF2L2 [Crassostrea gigas
9629468	gi|405965483|gb|EKC30852.1|	2.98721e-43	Dihydrolipoyl dehydrogenase, mitochondrial [Crassostrea gigas
9640462	gi|405960743|gb|EKC26631.1|	2.99084e-130	NEDD4-like E3 ubiquitin-protein ligase WWP1 [Crassostrea gigas]
9642396	gi|676458639|ref|XP_009055041.1|	2.99145e-123	hypothetical protein LOTGIDRAFT_232454 [Lottia gigantea
9634514	gi|405971019|gb|EKC35877.1|	2.99271e-138	Acetolactate synthase-like protein [Crassostrea gigas]
9648384	gi|405977901|gb|EKC42328.1|	2.99802e-74	Inositol 1,4,5-triphosphate receptor-interacting protein [Crassostrea gigas
9652940	gi|676474303|ref|XP_009060103.1|	2e-27	hypothetical protein LOTGIDRAFT_154137 [Lottia gigantea
9622926	gi|524870168|ref|XP_005091870.1|	3.00923e-94	PREDICTED: PDZ domain-containing protein GIPC1-like [Aplysia californica
9573462	gi|585663816|ref|XP_006811905.1|	3.01954e-24	PREDICTED: geranylgeranyl transferase type-2 subunit alpha-like [Saccoglossus kowalevsk
9642966	gi|676482569|ref|XP_009062745.1|	3.0319e-94	hypothetical protein LOTGIDRAFT_220533 [Lottia gigantea]
9620828	gi|405967103|gb|EKC32304.1|	3.04223e-64	Choline dehydrogenase, mitochondrial [Crassostrea gigas
9640348	gi|524903041|ref|XP_005107738.1|	3.04231e-159	PREDICTED: EH domain-containing protein 3-like [Aplysia californica
9611010	gi|405973584|gb|EKC38289.1|	3.05746e-109	Glycylpeptide N-tetradecanoyltransferase 2 [Crassostrea gigas]
9635418	gi|676456230|ref|XP_009054259.1|	3.0605e-85	hypothetical protein LOTGIDRAFT_188902 [Lottia gigantea

9630878	gi|405970510|gb|EKC35408.1|	3.06422e-30	Eukaryotic translation initiation factor 2-alpha kinase 3 [Crassostrea gigas
9623742	gi|524886402|ref|XP_005099797.1|	3.0655e-110	PREDICTED: uncharacterized protein LOC101846429 [Aplysia californica]
9619682	gi|405965622|gb|EKC30984.1|	3.06562e-45	ATP-binding cassette sub-family G member 1 [Crassostrea gigas
9607934	gi|676478491|ref|XP_009061445.1|	3.07002e-55	hypothetical protein LOTGIDRAFT_92759, partial [Lottia gigantea]
9599423	gi|405974720|gb|EKC39344.1|	3.07074e-26	Transforming growth factor-beta receptor-associated protein 1 [Crassostrea gigas]
9647786	gi|524878979|ref|XP_005096169.1|	3.07675e-44	PREDICTED: uncharacterized protein LOC101856336 [Aplysia californica]
9645552	gi|676431482|ref|XP_009046266.1|	3.07743e-138	hypothetical protein LOTGIDRAFT_110843 [Lottia gigantea]
9632530	gi|443710038|gb|ELU04419.1|	3.08145e-105	hypothetical protein CAPTEDRAFT_173048 [Capitella teleta
9638988	gi|524889738|ref|XP_005101423.1|	3.08245e-12	PREDICTED: FYVE, RhoGEF and PH domain-containing protein 1-like isoform X2 [Aplysia californica
9637028	gi|602634276|ref|XP_007424011.1|	3.10479e-11	PREDICTED: inverted formin-2-like [Python bivittatus
9641958	gi|405951555|gb|EKC19458.1|	3.10499e-32	Breast cancer anti-estrogen resistance protein 1 [Crassostrea gigas]
9634876	gi|676432760|ref|XP_009046679.1|	3.11752e-50	hypothetical protein LOTGIDRAFT_237886 [Lottia gigantea]
9637092	gi|602705747|ref|XP_007447264.1|	3.11923e-11	PREDICTED: collagen alpha-1(IX) chain isoform X1 [Lipotes vexillifer
9646506	gi|405957765|gb|EKC23951.1|	3.12696e-104	PHD finger protein 12 [Crassostrea gigas]
9636368	gi|405952904|gb|EKC20659.1|	3.13611e-109	Guanine nucleotide exchange factor DBS [Crassostrea gigas
9640384	gi|676461454|ref|XP_009055949.1|	3.14058e-63	hypothetical protein LOTGIDRAFT_189885 [Lottia gigantea
9641888	gi|405959405|gb|EKC25450.1|	3.14258e-93	SH2 domain-containing protein 3C [Crassostrea gigas
9637776	gi|405973281|gb|EKC38004.1|	3.14649e-91	Drebrin-like protein [Crassostrea gigas]
9638216	gi|676493030|ref|XP_009066114.1|	3.15867e-139	hypothetical protein LOTGIDRAFT_169573 [Lottia gigantea
9615568	gi|676484295|ref|XP_009063300.1|	3.16218e-70	hypothetical protein LOTGIDRAFT_129863 [Lottia gigantea]
9643146	gi|405973160|gb|EKC37890.1|	3.16242e-99	Sushi, von Willebrand factor type A, EGF and pentraxin domain-containing protein 1 [Crassostrea giga
9647856	gi|676474897|ref|XP_009060293.1|	3.16258e-81	hypothetical protein LOTGIDRAFT_106318 [Lottia gigantea]
9617580	gi|405957979|gb|EKC24153.1|	3.16282e-68	Diacylglycerol O-acyltransferase 1 [Crassostrea gigas]
9631560	gi|676480191|ref|XP_009061984.1|	3.16837e-131	hypothetical protein LOTGIDRAFT_107431 [Lottia gigantea]
9647802	gi|524915635|ref|XP_005112611.1|	3.1692e-57	PREDICTED: probable exonuclease mut-7 homolog [Aplysia californica
9630962	gi|260832388|ref|XP_002611139.1|	3.17834e-19	hypothetical protein BRAFLDRAFT_88462 [Branchiostoma floridae]
9648326	gi|405949970|gb|EKC17980.1|	3.18087e-76	Tetratricopeptide repeat protein 5 [Crassostrea gigas]
9650432	gi|405966133|gb|EKC31451.1|	3.18584e-49	Rap guanine nucleotide exchange factor 1 [Crassostrea gigas
9648912	gi|291222299|ref|XP_002731141.1|	3.19233e-159	PREDICTED: high affinity cationic amino acid transporter 1-like [Saccoglossus kowalevski
9584419	gi|405953233|gb|EKC20935.1|	3.20202e-22	Disintegrin and metalloproteinase domain-containing protein 10 [Crassostrea gigas
9643774	gi|524886975|ref|XP_005100077.1|	3.20688e-62	PREDICTED: vacuolar protein sorting-associated protein 37A-like [Aplysia californica
9580668	gi|524882530|ref|XP_005097908.1|	3.21162e-45	PREDICTED: protein sel-1 homolog 1-like isoform X1 [Aplysia californica
9628786	gi|524875783|ref|XP_005094615.1|	3.21966e-42	PREDICTED: trafficking protein particle complex subunit 9-like isoform X2 [Aplysia californica
9632980	gi|405962302|gb|EKC27994.1|	3.23171e-17	hypothetical protein CGI_10017316 [Crassostrea gigas]
9624794	gi|405951518|gb|EKC19424.1|	3.23205e-22	RalBP1-associated Eps domain-containing protein 1 [Crassostrea gigas
9627248	gi|405966280|gb|EKC31587.1|	3.23345e-84	Transmembrane protein 86A [Crassostrea gigas]
9611512	gi|524874196|ref|XP_005093837.1|	3.23645e-66	PREDICTED: protein canopy homolog 3-like [Aplysia californica
9579366	gi|443691007|gb|ELT92991.1|	3.24342e-50	hypothetical protein CAPTEDRAFT_98851 [Capitella teleta
9602204	gi|524871732|ref|XP_005092637.1|	3.2488e-21	PREDICTED: collagen alpha-1(XI) chain-like [Aplysia californica
9631024	gi|405970869|gb|EKC35736.1|	3.25536e-72	Arf-GAP with coiled-coil, ANK repeat and PH domain-containing protein 2 [Crassostrea gigas
9624846	gi|161110488|gb|ABX57736.1|	3.26177e-131	TFG beta signaling pathway factor [Pinctada fucata
9616778	gi|524890041|ref|XP_005101570.1|	3.2634e-94	PREDICTED: protein NDRG3-like isoform X3 [Aplysia californica]
9645992	gi|676473619|ref|XP_009059877.1|	3.27288e-159	hypothetical protein LOTGIDRAFT_218886 [Lottia gigantea
9644630	gi|676479442|ref|XP_009061749.1|	3.27734e-49	hypothetical protein LOTGIDRAFT_166436 [Lottia gigantea
9649568	gi|405967683|gb|EKC32819.1|	3.28782e-162	Ras GTPase-activating protein 1 [Crassostrea gigas]
9616016	gi|405967691|gb|EKC32825.1|	3.28913e-83	Sentrin-specific protease 1 [Crassostrea gigas]
9639846	gi|524881030|ref|XP_005097171.1|	3.293e-33	PREDICTED: protein SCAF8-like [Aplysia californica
9652904	gi|405950058|gb|EKC18065.1|	3.30138e-99	Serine/threonine-protein kinase ULK2 [Crassostrea gigas]
9651370	gi|405976866|gb|EKC41345.1|	3.30279e-74	Ataxin-1 [Crassostrea gigas]
9634318	gi|405970131|gb|EKC35063.1|	3.30708e-121	Sialin [Crassostrea gigas]
9617718	gi|676486864|ref|XP_009064112.1|	3.31887e-17	hypothetical protein LOTGIDRAFT_167982 [Lottia gigantea]
9637416	gi|676472655|ref|XP_009059560.1|	3.33039e-178	hypothetical protein LOTGIDRAFT_124718 [Lottia gigantea]
9650222	gi|676481082|ref|XP_009062262.1|	3.33053e-144	hypothetical protein LOTGIDRAFT_210691 [Lottia gigantea]
9641376	gi|443693945|gb|ELT95205.1|	3.33273e-31	hypothetical protein CAPTEDRAFT_154663 [Capitella teleta
9579422	gi|260818627|ref|XP_002604484.1|	3.34089e-63	hypothetical protein BRAFLDRAFT_114510 [Branchiostoma floridae]
9640418	gi|405962511|gb|EKC28180.1|	3.36139e-174	Serine/threonine-protein kinase D1, partial [Crassostrea gigas
9603222	gi|405959695|gb|EKC25702.1|	3.36962e-106	Clathrin heavy chain 1 [Crassostrea gigas]
9644864	gi|405969995|gb|EKC34935.1|	3.3807e-110	Choline-phosphate cytidylyltransferase B [Crassostrea gigas
9653004	gi|524906263|ref|XP_005108462.1|	3.38126e-38	PREDICTED: uncharacterized protein LOC101850281 [Aplysia californica]
9640808	gi|405958558|gb|EKC24674.1|	3.40591e-81	Hemicentin-1 [Crassostrea gigas]
9642924	gi|607357226|gb|EZA51692.1|	3.40732e-13	Acetylcholine receptor subunit alpha-like protein [Cerapachys biro
9626078	gi|301773242|ref|XP_002922050.1|	3.42114e-91	PREDICTED: hypothetical protein LOC100473890 [Ailuropoda melanoleuca
9644840	gi|405967040|gb|EKC32254.1|	3.44214e-163	hypothetical protein CGI_10026244 [Crassostrea gigas]
9627288	gi|405969575|gb|EKC34537.1|	3.44603e-118	Putative palmitoyltransferase ZDHHC14 [Crassostrea gigas]
9636312	gi|405964550|gb|EKC30021.1|	3.45078e-108	Multiple PDZ domain protein [Crassostrea gigas
9641310	gi|676459084|ref|XP_009055186.1|	3.45185e-12	hypothetical protein LOTGIDRAFT_161572 [Lottia gigantea]
9643234	gi|405967472|gb|EKC32627.1|	3.49208e-114	Syntaxin-binding protein 5 [Crassostrea gigas
9578998	gi|524878049|ref|XP_005095717.1|	3.4976e-20	PREDICTED: la-related protein 6-like [Aplysia californica
9582069	gi|360127119|gb|AEV93606.1|	3.50579e-47	P-glycoprotein [Xiphophorus helleri
9651516	gi|676431406|ref|XP_009046241.1|	3.5114e-164	hypothetical protein LOTGIDRAFT_212290 [Lottia gigantea]

9598395	gi|405967934|gb|EKC33048.1|	3.51454e-30	Atrial natriuretic peptide-converting enzyme [Crassostrea gigas
9629342	gi|405969345|gb|EKC34321.1|	3.51464e-36	Ubiquitin-associated protein 2 [Crassostrea gigas]
9622856	gi|321459525|gb|EFX70577.1|	3.51562e-18	hypothetical protein DAPPUDRAFT_327936 [Daphnia pulex
9649530	gi|405973733|gb|EKC38427.1|	3.51645e-31	E3 ubiquitin-protein ligase TRIM33 [Crassostrea gigas
9591109	gi|405964818|gb|EKC30263.1|	3.52868e-12	Tripartite motif-containing protein 3 [Crassostrea gigas]
9643940	gi|405971528|gb|EKC36363.1|	3.5391e-101	Ras-related protein Rab-33B [Crassostrea gigas]
9587959	gi|676477793|ref|XP_009061219.1|	3.54888e-67	hypothetical protein LOTGIDRAFT_206942 [Lottia gigantea
9643168	gi|676430375|ref|XP_009045905.1|	3.56194e-34	hypothetical protein LOTGIDRAFT_237684 [Lottia gigantea]
9594019	gi|676488269|ref|XP_009064562.1|	3.56543e-103	hypothetical protein LOTGIDRAFT_207233 [Lottia gigantea]
9611896	gi|405973713|gb|EKC38408.1|	3.56764e-113	Serine/threonine-protein kinase PAK 3 [Crassostrea gigas]
9591235	gi|524872626|ref|XP_005093074.1|	3.5679e-89	PREDICTED: ras-specific guanine nucleotide-releasing factor RalGPS2-like, partial [Aplysia californica
9618526	gi|405977818|gb|EKC42251.1|	3.56824e-91	Guanine nucleotide exchange factor VAV2 [Crassostrea gigas
9642748	gi|405969938|gb|EKC34881.1|	3.56948e-47	AP1 subunit gamma-binding protein 1 [Crassostrea gigas
9640270	gi|524882644|ref|XP_005097963.1|	3.57316e-51	PREDICTED: ralBP1-associated Eps domain-containing protein 1-like isoform X2 [Aplysia californica
9630612	gi|405950254|gb|EKC18253.1|	3.57462e-148	Tyrosine-protein kinase PR2 [Crassostrea gigas]
9653288	gi|405976896|gb|EKC41374.1|	3.57806e-127	Transcription initiation factor TFIID subunit 4 [Crassostrea gigas
9600350	gi|676479023|ref|XP_009061615.1|	3.59638e-46	hypothetical protein LOTGIDRAFT_194163 [Lottia gigantea]
9619282	gi|405951392|gb|EKC19308.1|	3.59843e-38	E3 ubiquitin-protein ligase RNF19A [Crassostrea gigas
9613076	gi|524890619|ref|XP_005101853.1|	3.60579e-108	PREDICTED: protein transport protein Sec24C-like isoform X1 [Aplysia californica]
9621272	gi|676436854|ref|XP_009048016.1|	3.60686e-81	hypothetical protein LOTGIDRAFT_111786 [Lottia gigantea]
9645604	gi|405972960|gb|EKC37702.1|	3.61412e-54	Tenascin-X [Crassostrea gigas]
9631794	gi|699624049|ref|XP_009898675.1|	3.61433e-42	PREDICTED: dihydrolipoyllysine-residue acetyltransferase component of pyruvate dehydrogenase complex, mitochondrial [Picoides pubescen
9647222	gi|676422813|ref|XP_009044007.1|	3.61824e-127	hypothetical protein LOTGIDRAFT_181302 [Lottia gigantea]
9617588	gi|676459676|ref|XP_009055378.1|	3.61874e-64	hypothetical protein LOTGIDRAFT_104744, partial [Lottia gigantea]
9599319	gi|676473607|ref|XP_009059873.1|	3.61896e-35	hypothetical protein LOTGIDRAFT_204335 [Lottia gigantea]
9649870	gi|405973103|gb|EKC37835.1|	3.63358e-55	Ankyrin repeat and protein kinase domain-containing protein 1 [Crassostrea gigas
9648038	gi|676476700|ref|XP_009060875.1|	3.65442e-101	hypothetical protein LOTGIDRAFT_125925 [Lottia gigantea
9595685	gi|405968786|gb|EKC33819.1|	3.66054e-74	Succinyl-CoA ligase [GDP-forming] subunit beta, mitochondrial [Crassostrea gigas
9649170	gi|524874576|ref|XP_005094023.1|	3.68237e-94	PREDICTED: abhydrolase domain-containing protein 2-like [Aplysia californica
9602886	gi|524883984|ref|XP_005098617.1|	3.68305e-97	PREDICTED: sorting nexin-6-like isoform X1 [Aplysia californica
9599539	gi|676472676|ref|XP_009059567.1|	3.68317e-27	hypothetical protein LOTGIDRAFT_124564 [Lottia gigantea
9648312	gi|405964094|gb|EKC29616.1|	3.69895e-16	hypothetical protein CGI_10027404 [Crassostrea gigas]
9614796	gi|405957767|gb|EKC23953.1|	3.70004e-79	CDP-diacylglycerol--inositol 3-phosphatidyltransferase, partial [Crassostrea gigas
9615704	gi|482426321|emb|CCV01190.1|	3.70087e-103	ABCB/P-glycoprotein-like protein [Mytilus galloprovincialis
9615916	gi|676463053|ref|XP_009056472.1|	3.70466e-18	hypothetical protein LOTGIDRAFT_232908 [Lottia gigantea
9583405	gi|676480246|ref|XP_009061999.1|	3.7057e-52	hypothetical protein LOTGIDRAFT_229242 [Lottia gigantea]
9634854	gi|524881876|ref|XP_005097586.1|	3.70631e-144	PREDICTED: solute carrier family 12 member 9-like [Aplysia californica
9648566	gi|405952000|gb|EKC19860.1|	3.72421e-72	Ras association domain-containing protein 9 [Crassostrea gigas]
9646130	gi|676456689|ref|XP_009054404.1|	3.72605e-86	hypothetical protein LOTGIDRAFT_207958 [Lottia gigantea
9639078	gi|443726885|gb|ELU13881.1|	3.72968e-175	hypothetical protein CAPTEDRAFT_175923 [Capitella teleta
9631778	gi|676487419|ref|XP_009064287.1|	3.73158e-47	hypothetical protein LOTGIDRAFT_58640, partial [Lottia gigantea]
9619140	gi|405952378|gb|EKC20197.1|	3.7411e-104	Eukaryotic translation initiation factor 4 gamma 2 [Crassostrea gigas
9637860	gi|405950485|gb|EKC18471.1|	3.74217e-84	Tyrosine-protein kinase HTK16 [Crassostrea gigas]
9613460	gi|524884447|ref|XP_005098843.1|	3.74617e-12	PREDICTED: SH3 and PX domain-containing protein 2A-like [Aplysia californica
9643724	gi|405951692|gb|EKC19584.1|	3.74961e-87	Inter-alpha-trypsin inhibitor heavy chain H3 [Crassostrea gigas
9603168	gi|324120664|dbj|BAJ78723.1|	3.75156e-81	RNA polymerase II second largest subunit [Inocellia japonica
9570148	gi|405977643|gb|EKC42082.1|	3.75934e-11	CD63 antigen [Crassostrea gigas]
9637196	gi|405977415|gb|EKC41871.1|	3.77772e-57	Nuclear RNA export factor 1 [Crassostrea gigas]
9644252	gi|567768093|gb|AHC94802.1|	3.78027e-66	TRAF6 [Pinctada martensii]
9635584	gi|676455488|ref|XP_009054022.1|	3.79477e-51	hypothetical protein LOTGIDRAFT_117101, partial [Lottia gigantea
9652410	gi|524879059|ref|XP_005096209.1|	3.80123e-104	PREDICTED: uncharacterized protein ZC262.3-like isoform X1 [Aplysia californica]
9623398	gi|405964042|gb|EKC29564.1|	3.81273e-137	Coatomer subunit alpha [Crassostrea gigas
9574332	gi|524909232|ref|XP_005109665.1|	3.8187e-11	PREDICTED: mucin-12-like isoform X1 [Aplysia californica
9613358	gi|524875398|ref|XP_005094427.1|	3.81895e-25	PREDICTED: uncharacterized protein LOC101848876 [Aplysia californica]
9637166	gi|405974590|gb|EKC39224.1|	3.8197e-134	DENN domain-containing protein 5A [Crassostrea gigas
9585145	gi|744457|prf||2014371A	3.8219e-63	kinesin
9649166	gi|405950132|gb|EKC18136.1|	3.84685e-51	Beta-1,3-galactosyltransferase 1 [Crassostrea gigas]
9640004	gi|405951549|gb|EKC19452.1|	3.84944e-98	hypothetical protein CGI_10008502 [Crassostrea gigas]
9639066	gi|260817491|ref|XP_002603620.1|	3.86172e-55	hypothetical protein BRAFLDRAFT_93164 [Branchiostoma floridae
9615308	gi|405976491|gb|EKC40996.1|	3.87224e-28	PRKC apoptosis WT1 regulator protein [Crassostrea gigas]
9629110	gi|676481003|ref|XP_009062237.1|	3.87713e-136	hypothetical protein LOTGIDRAFT_107156 [Lottia gigantea
9649548	gi|676475408|ref|XP_009060464.1|	3.89381e-147	hypothetical protein LOTGIDRAFT_125375, partial [Lottia gigantea]
9652080	gi|405977266|gb|EKC41725.1|	3.89563e-79	hypothetical protein CGI_10028472 [Crassostrea gigas]
9647080	gi|390356209|ref|XP_003728730.1|	3.90479e-174	PREDICTED: LOW QUALITY PROTEIN: seali [Strongylocentrotus purpuratus
9621138	gi|405953450|gb|EKC21110.1|	3.9076e-35	Nuclear factor interleukin-3-regulated protein [Crassostrea gigas
9612934	gi|405957663|gb|EKC23860.1|	3.91083e-22	BAT2 domain-containing protein 1 [Crassostrea gigas
9611684	gi|405951867|gb|EKC19740.1|	3.91927e-19	Nuclear receptor ROR-alpha [Crassostrea gigas]
9644378	gi|405958979|gb|EKC25057.1|	3.93459e-180	DNA mismatch repair protein Msh6 [Crassostrea gigas]
9605082	gi|524886218|ref|XP_005099706.1|	3.94081e-41	PREDICTED: protein UBASH3A homolog [Aplysia californica
9651250	gi|405969212|gb|EKC34195.1|	3.94155e-88	Tudor domain-containing protein 7 [Crassostrea gigas

9651466	gi|602636720|ref|XP_007425212.1|	3.96135e-176	PREDICTED: roquin-1 isoform X2 [Python bivittatus
9595935	gi|571032608|gb|AHF21789.1|	3.96576e-28	suppressor of cytokine signalling-2 [Ruditapes philippinarun
9611044	gi|676429522|ref|XP_009045627.1|	3.96764e-16	hypothetical protein LOTGIDRAFT_171182 [Lottia gigantea]
9626588	gi|676466863|ref|XP_009057704.1|	3.96985e-111	hypothetical protein LOTGIDRAFT_178767 [Lottia gigantea]
9649802	gi|676467833|ref|XP_009058015.1|	3.97417e-179	hypothetical protein LOTGIDRAFT_105202 [Lottia gigantea]
9601796	gi|405970251|gb|EKC35177.1|	3.97738e-80	UPF0663 transmembrane protein C17orf28 [Crassostrea gigas]
9647668	gi|405971265|gb|EKC36111.1|	3.97857e-36	F-box only protein 25 [Crassostrea gigas]
9647404	gi|405963329|gb|EKC28912.1|	3.98932e-73	Tetraspanin-33 [Crassostrea gigas]
9651764	gi|524905595|ref|XP_005108174.1|	3.99052e-102	PREDICTED: hornerin-like isoform X1 [Aplysia californica
9646818	gi|676450548|ref|XP_009052414.1|	3.99418e-139	hypothetical protein LOTGIDRAFT_231720 [Lottia gigantea]
9604166	gi|405961346|gb|EKC27164.1|	3.99788e-70	High affinity cAMP-specific and IBMX-insensitive 3',5'-cyclic phosphodiesterase 8B [Crassostrea gigas
9645674	gi|676481001|ref|XP_009062236.1|	4.02837e-45	hypothetical protein LOTGIDRAFT_154791 [Lottia gigantea
9617018	gi|676464431|ref|XP_009056920.1|	4.03622e-84	hypothetical protein LOTGIDRAFT_120942 [Lottia gigantea]
9620058	gi|405951058|gb|EKC19003.1|	4.04627e-29	Sodium- and chloride-dependent glycine transporter 2 [Crassostrea gigas
9572938	gi|348505787|ref|XP_003440442.1|	4.05379e-53	PREDICTED: dnaJ homolog subfamily A member 2-like [Oreochromis niloticu
9642058	gi|405960231|gb|EKC26172.1|	4.05431e-173	Vigilin [Crassostrea gigas]
9645034	gi|405969351|gb|EKC34327.1|	4.07042e-87	Fidgetin-like protein 1 [Crassostrea gigas
9586109	gi|676474861|ref|XP_009060281.1|	4.08816e-20	hypothetical protein LOTGIDRAFT_229134 [Lottia gigantea
9651418	gi|156341935|ref|XP_001620823.1|	4.09458e-40	hypothetical protein NEMVEDRAFT_v1g248755 [Nematostella vectensis]
9645198	gi|524864217|ref|XP_005088946.1|	4.1004e-103	PREDICTED: polycystic kidney disease protein 1-like 2-like [Aplysia californica
9651486	gi|405952100|gb|EKC19948.1|	4.11151e-64	Tensin-4 [Crassostrea gigas]
9613576	gi|405977266|gb|EKC41725.1|	4.13228e-19	hypothetical protein CGI_10028472 [Crassostrea gigas]
9604420	gi|405964002|gb|EKC29530.1|	4.13577e-59	Protein FAM65C [Crassostrea gigas]
9635930	gi|524883658|ref|XP_005098456.1|	4.14076e-146	PREDICTED: alkyldihydroxyacetonephosphate synthase, peroxisomal-like [Aplysia californic
9601580	gi|405953233|gb|EKC20935.1|	4.14468e-53	Disintegrin and metalloproteinase domain-containing protein 10 [Crassostrea gigas
9626260	gi|676459495|ref|XP_009055319.1|	4.14614e-28	hypothetical protein LOTGIDRAFT_104845, partial [Lottia gigantea]
9568470	gi|391346812|ref|XP_003747662.1|	4.14781e-26	PREDICTED: ras association domain-containing protein 1-like [Metaseiulus occidentalis
9652268	gi|524889956|ref|XP_005101528.1|	4.16032e-132	PREDICTED: protein FAM135A-like [Aplysia californica
9649728	gi|405951963|gb|EKC19827.1|	4.16837e-88	UDP-galactose translocator [Crassostrea gigas]
9636036	gi|676461082|ref|XP_009055830.1|	4.1802e-130	hypothetical protein LOTGIDRAFT_119149, partial [Lottia gigantea]
9620774	gi|405978767|gb|EKC43130.1|	4.18052e-90	Uncharacterized protein C22orf9-like protein [Crassostrea gigas]
9634760	gi|405959418|gb|EKC25460.1|	4.18235e-55	Zinc finger homeobox protein 3 [Crassostrea gigas
9640752	gi|405971288|gb|EKC36134.1|	4.18595e-47	Transcription factor MafK [Crassostrea gigas]
9616012	gi|405956446|gb|EKC23049.1|	4.19954e-46	hypothetical protein CGI_10000765 [Crassostrea gigas]
9642032	gi|405957797|gb|EKC23980.1|	4.20207e-37	Ankyrin repeat domain-containing protein 17 [Crassostrea gigas
9622174	gi|524898642|ref|XP_005105767.1|	4.20428e-125	PREDICTED: tyrosine-protein phosphatase non-receptor type 4-like [Aplysia californica
9613388	gi|602654651|ref|XP_007433146.1|	4.23675e-48	PREDICTED: estradiol 17-beta-dehydrogenase 2 [Python bivittatus
9648364	gi|676484683|ref|XP_009063418.1|	4.23971e-137	hypothetical protein LOTGIDRAFT_130072, partial [Lottia gigantea
9651304	gi|405965756|gb|EKC31110.1|	4.29203e-58	Hairy/enhancer-of-split related with YRPW motif protein [Crassostrea gigas
9624282	gi|405964105|gb|EKC29627.1|	4.3074e-51	Liprin-beta-1 [Crassostrea gigas]
9597141	gi|405975439|gb|EKC40004.1|	4.30778e-86	Golgi SNAP receptor complex member 1 [Crassostrea gigas
9652494	gi|524912183|ref|XP_005110944.1|	4.3189e-79	PREDICTED: uncharacterized protein LOC101861007 isoform X1 [Aplysia californica]
9625036	gi|405977349|gb|EKC41806.1|	4.3197e-83	E3 ubiquitin-protein ligase NEDD4 [Crassostrea gigas
9614184	gi|524914691|ref|XP_005112157.1|	4.32411e-48	PREDICTED: TBC domain-containing protein kinase-like protein-like [Aplysia californica
9621096	gi|405954597|gb|EKC21994.1|	4.32803e-143	hypothetical protein CGI_10002990 [Crassostrea gigas]
9609716	gi|524887879|ref|XP_005100522.1|	4.3291e-68	PREDICTED: striatin-3-like isoform X3 [Aplysia californica]
9571586	gi|195019225|ref|XP_001984936.1|	4.3322e-63	GH14768 [Drosophila grimshawi] >gi|193898418|gb|EDV97284.1| GH14768 [Drosophila grimshaw
9619026	gi|405952904|gb|EKC20659.1|	4.34035e-52	Guanine nucleotide exchange factor DBS [Crassostrea gigas
9607096	gi|405968724|gb|EKC33770.1|	4.3464e-38	Carboxypeptidase D [Crassostrea gigas]
9619240	gi|524914134|ref|XP_005111886.1|	4.34806e-76	PREDICTED: inner nuclear membrane protein Man1-like [Aplysia californica
9626106	gi|405973316|gb|EKC38036.1|	4.34834e-94	Lysine-specific demethylase 5A [Crassostrea gigas]
9600484	gi|443690233|gb|ELT92419.1|	4.35568e-76	hypothetical protein CAPTEDRAFT_148966 [Capitella teleta
9653586	gi|405963867|gb|EKC29401.1|	4.35724e-65	hypothetical protein CGI_10005214 [Crassostrea gigas]
9643108	gi|405965792|gb|EKC31146.1|	4.36734e-125	Tubulin-specific chaperone D [Crassostrea gigas]
9633126	gi|405978367|gb|EKC42766.1|	4.36895e-147	CREB-binding protein [Crassostrea gigas
9581135	gi|573897847|ref|XP_006636658.1|	4.37094e-18	PREDICTED: Golgi reassembly-stacking protein 2-like [Lepisosteus oculatus
9580772	gi|260800756|ref|XP_002595263.1|	4.37094e-18	hypothetical protein BRAFLDRAFT_97213 [Branchiostoma floridae]
9647186	gi|524912688|ref|XP_005111186.1|	4.39127e-141	PREDICTED: uncharacterized protein LOC101861402 [Aplysia californica]
9650040	gi|443704393|gb|ELU01456.1|	4.39682e-149	hypothetical protein CAPTEDRAFT_92464 [Capitella teleta
9636994	gi|260830286|ref|XP_002610092.1|	4.43162e-50	hypothetical protein BRAFLDRAFT_125655 [Branchiostoma floridae]
9649358	gi|405961267|gb|EKC27099.1|	4.43369e-95	Wilms tumor protein 1-interacting-like protein [Crassostrea gigas
9649002	gi|676431419|ref|XP_009046245.1|	4.46488e-68	hypothetical protein LOTGIDRAFT_156721 [Lottia gigantea
9639148	gi|524883616|ref|XP_005098435.1|	4.46557e-144	PREDICTED: V-type proton ATPase 116 kDa subunit a isoform 1-like isoform X3 [Aplysia californica
9637778	gi|405954517|gb|EKC21934.1|	4.47035e-146	Brefeldin A-inhibited guanine nucleotide-exchange protein 1 [Crassostrea gigas
9639582	gi|524897931|ref|XP_005105420.1|	4.4865e-176	PREDICTED: ATP-binding cassette sub-family E member 1-like isoform X3 [Aplysia californica
9631140	gi|524881030|ref|XP_005097171.1|	4.4878e-21	PREDICTED: protein SCAF8-like [Aplysia californica
9636436	gi|524893419|ref|XP_005103225.1|	4.48915e-23	PREDICTED: tripartite motif-containing protein 2-like [Aplysia californica
9623100	gi|676476506|ref|XP_009060815.1|	4.5004e-114	hypothetical protein LOTGIDRAFT_126136, partial [Lottia gigantea]
9641582	gi|405972571|gb|EKC37333.1|	4.51719e-44	Cartilage matrix protein [Crassostrea gigas]
9630820	gi|405974952|gb|EKC39559.1|	4.52609e-66	SET and MYND domain-containing protein 5 [Crassostrea gigas

9589507	gi|405973427|gb|EKC38144.1|	4.54681e-41	ADP-ribosylation factor-binding protein GGA3 [Crassostrea gigas
9630270	gi|405963423|gb|EKC28997.1|	4.55109e-79	Williams-Beuren syndrome chromosomal region 14 protein [Crassostrea gigas
9625812	gi|405959322|gb|EKC25373.1|	4.58037e-96	Pre-mRNA-splicing factor 18 [Crassostrea gigas]
9569442	gi|166406785|gb|ABY87356.1|	4.58483e-17	hypothetical protein [Haliotis diversicolor
9649562	gi|676436888|ref|XP_009048027.1|	4.59146e-39	hypothetical protein LOTGIDRAFT_157573 [Lottia gigantea]
9609830	gi|405976018|gb|EKC40542.1|	4.59159e-86	DnaJ-like protein subfamily C member 7 [Crassostrea gigas
9641652	gi|565412058|dbj|BAO20820.1|	4.60147e-121	GABA transporter1 [Bathymodiolus septemdierum
9605152	gi|676421273|ref|XP_009043798.1|	4.60826e-122	hypothetical protein LOTGIDRAFT_227913 [Lottia gigantea]
9650644	gi|405945264|gb|EKC17247.1|	4.60916e-27	Kelch-like protein 24 [Crassostrea gigas]
9647224	gi|405975315|gb|EKC39889.1|	4.63047e-82	Suppressor of cytokine signaling 5 [Crassostrea gigas
9639170	gi|405975054|gb|EKC39650.1|	4.63187e-133	transport protein Sec24B [Crassostrea gigas]
9639088	gi|405957344|gb|EKC23562.1|	4.6554e-101	G/T mismatch-specific thymine DNA glycosylase [Crassostrea gigas
9639366	gi|405967643|gb|EKC32780.1|	4.66067e-16	Bromodomain testis-specific protein [Crassostrea gigas]
9595667	gi|676475445|ref|XP_009060476.1|	4.66293e-45	hypothetical protein LOTGIDRAFT_165220 [Lottia gigantea]
9592623	gi|405978198|gb|EKC42608.1|	4.70553e-15	canopy-like protein 2 [Crassostrea gigas]
9588681	gi|405978194|gb|EKC42604.1|	4.71862e-75	Putative sulfite oxidase, mitochondrial [Crassostrea gigas
9627010	gi|645013650|ref|XP_008205204.1|	4.73514e-11	PREDICTED: death-associated protein kinase related-like isoform X1 [Nasonia vitripennis
9625290	gi|405951297|gb|EKC19222.1|	4.7377e-45	Sperm-associated antigen 1 [Crassostrea gigas]
9595125	gi|676470913|ref|XP_009059007.1|	4.75597e-34	hypothetical protein LOTGIDRAFT_123506 [Lottia gigantea]
9641040	gi|405951377|gb|EKC19295.1|	4.77447e-107	hypothetical protein CGI_10008895 [Crassostrea gigas]
9650206	gi|405972865|gb|EKC37612.1|	4.7805e-42	Metal-response element-binding transcription factor 2 [Crassostrea gigas
9647234	gi|405970647|gb|EKC35536.1|	4.78181e-95	hypothetical protein CGI_10020303 [Crassostrea gigas]
9630296	gi|524909345|ref|XP_005109720.1|	4.78412e-137	PREDICTED: serine/threonine-protein kinase TAO1-like isoform X5 [Aplysia californica
9639168	gi|676449453|ref|XP_009052059.1|	4.80436e-122	hypothetical protein LOTGIDRAFT_115058 [Lottia gigantea]
9643818	gi|405962937|gb|EKC28565.1|	4.80771e-58	Mitogen-activated protein kinase kinase kinase 13-B [Crassostrea gigas
9645358	gi|405959735|gb|EKC25735.1|	4.81122e-168	Protein pelota [Crassostrea gigas]
9573130	gi|498968865|ref|XP_004526420.1|	4.84445e-14	PREDICTED: uncharacterized protein LOC101449230 isoform X1 [Ceratitis capitata]
9650832	gi|156364755|ref|XP_001626511.1|	4.85041e-64	predicted protein [Nematostella vectensis]
9640210	gi|443688808|gb|ELT91395.1|	4.85173e-32	hypothetical protein CAPTEDRAFT_228118 [Capitella teleta
9644260	gi|676480483|ref|XP_009062074.1|	4.85768e-122	hypothetical protein LOTGIDRAFT_107273 [Lottia gigantea
9643252	gi|405977912|gb|EKC42339.1|	4.87793e-124	Proton-coupled folate transporter [Crassostrea gigas]
9652718	gi|405963355|gb|EKC28938.1|	4.91019e-89	INO80 complex subunit D [Crassostrea gigas]
9641568	gi|405957283|gb|EKC23507.1|	4.91391e-99	RNA-binding protein MEX3C [Crassostrea gigas]
9612754	gi|148887771|gb|ABR15461.1|	4.92432e-33	HSF [Haliotis asinina]
9593793	gi|405962262|gb|EKC27956.1|	4.93342e-73	Ubiquitin carboxyl-terminal hydrolase 2 [Crassostrea gigas
9579894	gi|3746336|gb|AAC63909.1|	4.95618e-75	sarco/endoplasmic reticulum-type Ca-2+ATPase [Placopecten magellanicus
9589723	gi|676480302|ref|XP_009062017.1|	4.96913e-80	hypothetical protein LOTGIDRAFT_210457 [Lottia gigantea]
9651866	gi|405950210|gb|EKC18211.1|	5.00038e-76	hypothetical protein CGI_10014585 [Crassostrea gigas]
9636912	gi|632962448|ref|XP_007897319.1|	5.01185e-70	PREDICTED: E3 ubiquitin-protein ligase HACE1 isoform X1 [Callorhinchus mil
9650588	gi|405963970|gb|EKC29501.1|	5.03594e-42	hypothetical protein CGI_10025510 [Crassostrea gigas]
9642788	gi|405952630|gb|EKC20418.1|	5.04199e-134	Protein neuralized [Crassostrea gigas]
9581411	gi|676465457|ref|XP_009057250.1|	5.05218e-62	hypothetical protein LOTGIDRAFT_217098 [Lottia gigantea
9647650	gi|676421727|ref|XP_009043845.1|	5.05279e-124	hypothetical protein LOTGIDRAFT_208293 [Lottia gigantea]
9639994	gi|524898493|ref|XP_005105695.1|	5.06262e-74	PREDICTED: tyrosine-protein phosphatase Lar-like [Aplysia californica
9645886	gi|524916507|ref|XP_005113030.1|	5.07693e-112	PREDICTED: small heat shock protein p36-like [Aplysia californica
9614930	gi|676473339|ref|XP_009059786.1|	5.0826e-20	hypothetical protein LOTGIDRAFT_165019 [Lottia gigantea]
9575644	gi|548401787|ref|XP_005738790.1|	5.10022e-40	PREDICTED: calmodulin-like [Pundamilia nyerere
9617270	gi|405967821|gb|EKC32948.1|	5.11768e-71	Cholesterol 7-alpha-monooxygenase [Crassostrea gigas]
9643810	gi|405960322|gb|EKC26253.1|	5.12896e-169	Segment polarity protein dishevelled-like protein DVL-3 [Crassostrea gigas
9598389	gi|405965007|gb|EKC30438.1|	5.13139e-106	DNA-directed RNA polymerase II subunit RPB1 [Crassostrea gigas
9600948	gi|405970443|gb|EKC35345.1|	5.1407e-16	hypothetical protein CGI_10012484 [Crassostrea gigas]
9644364	gi|499045993|ref|XP_004573577.1|	5.14153e-63	PREDICTED: zinc finger protein 26-like [Maylandia zebra
9642712	gi|524894883|ref|XP_005103932.1|	5.14828e-38	PREDICTED: fibroblast growth factor receptor substrate 2-like [Aplysia californica
9645864	gi|405972025|gb|EKC36822.1|	5.16652e-157	Nuclear receptor subfamily 5 group A member 2 [Crassostrea gigas
9623310	gi|676433267|ref|XP_009046844.1|	5.17702e-118	hypothetical protein LOTGIDRAFT_199704 [Lottia gigantea]
9578734	gi|513219867|ref|XP_004947509.1|	5.17821e-48	PREDICTED: ubiquitin conjugation factor E4 B isoform X3 [Gallus gallu
9650500	gi|524896931|ref|XP_005104935.1|	5.1784e-132	PREDICTED: latrophilin-3-like [Aplysia californica
9619980	gi|676461082|ref|XP_009055830.1|	5.19104e-61	hypothetical protein LOTGIDRAFT_119149, partial [Lottia gigantea]
9641662	gi|405950385|gb|EKC18377.1|	5.19201e-165	Programmed cell death 6-interacting protein [Crassostrea gigas
9569954	gi|679008176|gb|KFW08024.1|	5.20935e-37	Integrin beta-3, partial [Eurypyga helias
9646732	gi|288551189|gb|ADC53123.1|	5.2235e-107	IL-1 receptor associated kinase 4 [Haliotis diversicolor]
9650082	gi|405968488|gb|EKC33555.1|	5.23825e-134	E3 ubiquitin-protein ligase UBR5 [Crassostrea gigas
9650124	gi|405953432|gb|EKC21095.1|	5.24718e-33	Pecanex-like protein 1 [Crassostrea gigas]
9636544	gi|325296731|ref|NP_001191601.1|	5.25461e-72	ELAV 2-like protein [Aplysia californica]
9646652	gi|524881856|ref|XP_005097577.1|	5.26663e-51	PREDICTED: uncharacterized protein LOC101863813 [Aplysia californica]
9648080	gi|405966260|gb|EKC31567.1|	5.27763e-116	Ubiquitin carboxyl-terminal hydrolase 20 [Crassostrea gigas
9588629	gi|676488684|ref|XP_009064694.1|	5.2779e-34	hypothetical protein LOTGIDRAFT_131897 [Lottia gigantea]
9594995	gi|405974458|gb|EKC39101.1|	5.28278e-17	E3 ubiquitin-protein ligase MIB2 [Crassostrea gigas
9604000	gi|676442556|ref|XP_009049839.1|	5.30686e-14	hypothetical protein LOTGIDRAFT_113430 [Lottia gigantea]
9649516	gi|93277256|gb|ABF06445.1|	5.31437e-96	AE-like protein [Doryteuthis pealeii

9595647	gi|524885969|ref|XP_005099585.1|	5.33507e-41	PREDICTED: transmembrane protein 214-B-like isoform X1 [Aplysia californica
9652110	gi|260826804|ref|XP_002608355.1|	5.34883e-36	hypothetical protein BRAFLDRAFT_91314 [Branchiostoma floridae]
9572282	gi|524889326|ref|XP_005101222.1|	5.36997e-13	PREDICTED: nuclear receptor coactivator 6-like [Aplysia californica
9612640	gi|405965509|gb|EKC30878.1|	5.38716e-32	DnaJ-like protein subfamily B member 9 [Crassostrea gigas
9643816	gi|405970329|gb|EKC35244.1|	5.3871e-126	Mediator of RNA polymerase II transcription subunit 14 [Crassostrea gigas
9626306	gi|676447856|ref|XP_009051545.1|	5.40281e-60	hypothetical protein LOTGIDRAFT_103971, partial [Lottia gigantea]
9574950	gi|524916601|ref|XP_005113074.1|	5.41282e-74	PREDICTED: uncharacterized protein LOC101849429 isoform X1 [Aplysia californica]
9644250	gi|646722013|gb|KDR23157.1|	5.44606e-73	Hyccin [Zootermopsis nevadensis]
9628200	gi|405962261|gb|EKC27955.1|	5.44828e-34	hypothetical protein CGI_10018418 [Crassostrea gigas]
9639664	gi|405951336|gb|EKC19257.1|	5.48453e-73	Potassium voltage-gated channel protein Shaw [Crassostrea gigas
9630262	gi|405976693|gb|EKC41191.1|	5.48645e-125	Coiled-coil and C2 domain-containing protein 1-like protein [Crassostrea gigas
9642644	gi|676471548|ref|XP_009059211.1|	5.49097e-149	hypothetical protein LOTGIDRAFT_218210 [Lottia gigantea]
9591783	gi|524902656|ref|XP_005107557.1|	5.50823e-37	PREDICTED: ras-related C3 botulinum toxin substrate 1-like [Aplysia californica
9622222	gi|676460273|ref|XP_009055569.1|	5.51649e-109	hypothetical protein LOTGIDRAFT_209470, partial [Lottia gigantea]
9633588	gi|524877064|ref|XP_005095239.1|	5.51659e-89	PREDICTED: protein transport protein Sec31A-like [Aplysia californica
9611454	gi|405952098|gb|EKC19946.1|	5.53337e-58	Ubiquitin carboxyl-terminal hydrolase 5 [Crassostrea gigas
9642492	gi|405978367|gb|EKC42766.1|	5.54168e-93	CREB-binding protein [Crassostrea gigas]
9647778	gi|405963150|gb|EKC28748.1|	5.5547e-70	Protein toll [Crassostrea gigas]
9636666	gi|405968489|gb|EKC33556.1|	5.57577e-114	Solute carrier organic anion transporter family member 4A1 [Crassostrea gigas
9576160	gi|675872642|ref|XP_009021881.1|	5.58531e-63	hypothetical protein HELRODRAFT_187263 [Helobdella robusta
9640360	gi|405962532|gb|EKC28198.1|	5.58615e-20	Neuropeptide Y receptor type 4 [Crassostrea gigas]
9623320	gi|405960204|gb|EKC26145.1|	5.59568e-80	Hypoxia up-regulated protein 1 [Crassostrea gigas
9615066	gi|676436704|ref|XP_009047968.1|	5.60646e-27	hypothetical protein LOTGIDRAFT_230663 [Lottia gigantea]
9633018	gi|405961962|gb|EKC27689.1|	5.62567e-62	Vam6/Vps39-like protein [Crassostrea gigas]
9650144	gi|676426988|ref|XP_009044801.1|	5.62998e-27	hypothetical protein LOTGIDRAFT_170715 [Lottia gigantea]
9601044	gi|405964042|gb|EKC29564.1|	5.63508e-95	Coatomer subunit alpha [Crassostrea gigas
9608134	gi|340369048|ref|XP_003383061.1|	5.6361e-73	PREDICTED: protein DD3-3-like [Amphimedon queenslandica
9592169	gi|521032522|gb|EPQ14307.1|	5.63632e-21	Carboxypeptidase D [Myotis brandtii
9640196	gi|405967256|gb|EKC32438.1|	5.66876e-57	Glypican-6 [Crassostrea gigas]
9638542	gi|405954670|gb|EKC22046.1|	5.67691e-19	hypothetical protein CGI_10002867 [Crassostrea gigas]
9622124	gi|676467231|ref|XP_009057822.1|	5.68226e-122	hypothetical protein LOTGIDRAFT_182444 [Lottia gigantea]
9600176	gi|405964008|gb|EKC29536.1|	5.68869e-76	Homeodomain-interacting protein kinase 2 [Crassostrea gigas
9652738	gi|405975811|gb|EKC40356.1|	5.69052e-61	Latrophilin-2 [Crassostrea gigas]
9652212	gi|585701837|ref|XP_006822956.1|	5.69374e-150	PREDICTED: uncharacterized protein LOC100371179 [Saccoglossus kowalevskii
9620382	gi|405966044|gb|EKC31369.1|	5.69436e-31	Collagen alpha-1(XII) chain [Crassostrea gigas
9646808	gi|405952727|gb|EKC20505.1|	5.70418e-61	Cartilage matrix protein [Crassostrea gigas]
9646284	gi|405974819|gb|EKC39432.1|	5.71892e-143	Serine/threonine-protein kinase RIO2 [Crassostrea gigas]
9649426	gi|524895322|ref|XP_005104146.1|	5.73501e-42	PREDICTED: lysine-specific demethylase 7-like [Aplysia californica
9630776	gi|405978628|gb|EKC43005.1|	5.74448e-29	Apelin receptor [Crassostrea gigas]
9580008	gi|657527505|ref|XP_008287784.1|	5.76342e-15	PREDICTED: forkhead box protein J3-like [Stegastes partitus
9619710	gi|126697446|gb|ABO26680.1|	5.76812e-52	RAB protein [Haliotis discus discus]
9621212	gi|405952266|gb|EKC20098.1|	5.77181e-71	Protein hu-li tai shao [Crassostrea gigas]
9625100	gi|405964247|gb|EKC29753.1|	5.78962e-19	Putative E3 ubiquitin-protein ligase HERC2 [Crassostrea gigas
9621484	gi|676477793|ref|XP_009061219.1|	5.79258e-87	hypothetical protein LOTGIDRAFT_206942 [Lottia gigantea]
9618952	gi|676449793|ref|XP_009052169.1|	5.81198e-81	hypothetical protein LOTGIDRAFT_143418 [Lottia gigantea]
9633978	gi|405960149|gb|EKC26094.1|	5.81383e-78	Blastula protease 10 [Crassostrea gigas]
9647046	gi|676427677|ref|XP_009045025.1|	5.83655e-45	hypothetical protein LOTGIDRAFT_176352, partial [Lottia gigantea]
9627056	gi|676429041|ref|XP_009045471.1|	5.83909e-94	hypothetical protein LOTGIDRAFT_206027 [Lottia gigantea]
9653170	gi|405958783|gb|EKC24875.1|	5.84361e-84	Nuclear respiratory factor 1 [Crassostrea gigas]
9651016	gi|405977956|gb|EKC42377.1|	5.84369e-65	hypothetical protein CGI_10005750 [Crassostrea gigas]
9584257	gi|646709832|gb|KDR15521.1|	5.85002e-53	F-actin-capping protein subunit alpha [Zootermopsis nevadensis
9604946	gi|405967893|gb|EKC33012.1|	5.86151e-53	Separin [Crassostrea gigas]
9627412	gi|676465439|ref|XP_009057244.1|	5.86442e-126	hypothetical protein LOTGIDRAFT_233250 [Lottia gigantea]
9623740	gi|524884315|ref|XP_005098779.1|	5.87591e-53	PREDICTED: dual specificity mitogen-activated protein kinase kinase 4-like isoform X4 [Aplysia californica
9650764	gi|405950417|gb|EKC18408.1|	5.8774e-62	Signal-induced proliferation-associated 1-like protein 1 [Crassostrea gigas
9645380	gi|405954164|gb|EKC21680.1|	5.88451e-49	hypothetical protein CGI_10003543 [Crassostrea gigas]
9571870	gi|542234517|ref|XP_005455382.1|	5.89603e-36	PREDICTED: dehydrodolichyl diphosphate synthase-like isoform X1 [Oreochromis niloticus
9617710	gi|676435759|ref|XP_009047661.1|	5.89606e-107	hypothetical protein LOTGIDRAFT_139282, partial [Lottia gigantea]
9636270	gi|524873805|ref|XP_005093645.1|	5.91351e-92	PREDICTED: huntingtin isoform X1 [Aplysia californica
9634740	gi|405966671|gb|EKC31928.1|	5.91455e-126	Lateral signaling target protein 2-like protein [Crassostrea gigas
9612516	gi|676436058|ref|XP_009047758.1|	5.91491e-55	hypothetical protein LOTGIDRAFT_139521, partial [Lottia gigantea]
9644414	gi|405962840|gb|EKC28483.1|	5.93913e-152	Amyloid beta A4 precursor protein-binding family B member 2 [Crassostrea gigas
9601522	gi|405964907|gb|EKC30346.1|	5.9406e-28	Mediator of RNA polymerase II transcription subunit 26 [Crassostrea gigas
9649074	gi|676494997|ref|XP_009066746.1|	5.94835e-137	hypothetical protein LOTGIDRAFT_197880 [Lottia gigantea
9617794	gi|405952378|gb|EKC20197.1|	5.96483e-67	Eukaryotic translation initiation factor 4 gamma 2 [Crassostrea gigas
9576494	gi|676421818|ref|XP_009043866.1|	5.96739e-65	hypothetical protein LOTGIDRAFT_149167 [Lottia gigantea]
9650548	gi|729054702|ref|WP_033935734.1|	5.97159e-11	hypothetical protein, partial [Lactobacillus mucosae
9637128	gi|405950131|gb|EKC18135.1|	5.97565e-71	Inverted formin-2 [Crassostrea gigas]
9633142	gi|405953738|gb|EKC21341.1|	5.98756e-96	SHC-transforming protein 1 [Crassostrea gigas]
9604970	gi|676483448|ref|XP_009063028.1|	5.99578e-90	hypothetical protein LOTGIDRAFT_220783 [Lottia gigantea]

9634914	gi|524881630|ref|XP_005097465.1|	6.02803e-78	PREDICTED: sphingomyelin phosphodiesterase 2-like [Aplysia californica
9639798	gi|676468340|ref|XP_009058178.1|	6.02928e-120	hypothetical protein LOTGIDRAFT_205708 [Lottia gigantea
9648068	gi|405976930|gb|EKC41408.1|	6.05205e-96	Transmembrane protein C2orf18 [Crassostrea gigas]
9618872	gi|524909234|ref|XP_005109666.1|	6.07335e-14	PREDICTED: mucin-12-like isoform X2 [Aplysia californica
9600124	gi|677548062|gb|KFR08970.1|	6.07785e-78	putative ATP-dependent RNA helicase DDX47, partial [Nipponia nippo
9609572	gi|524900139|ref|XP_005106493.1|	6.08293e-46	PREDICTED: protein PRRC2C-like isoform X2 [Aplysia californica
9640644	gi|524890010|ref|XP_005101555.1|	6.1304e-99	PREDICTED: kelch-like protein diablo-like [Aplysia californica
9646522	gi|676439421|ref|XP_009048847.1|	6.13101e-84	hypothetical protein LOTGIDRAFT_158021 [Lottia gigantea
9601094	gi|676446627|ref|XP_009051142.1|	6.13331e-33	hypothetical protein LOTGIDRAFT_226642 [Lottia gigantea]
9647974	gi|676441757|ref|XP_009049593.1|	6.13506e-16	hypothetical protein LOTGIDRAFT_238733 [Lottia gigantea]
9648772	gi|524865348|ref|XP_005089504.1|	6.13513e-147	PREDICTED: galactocerebrosidase-like isoform X2 [Aplysia californica]
9650312	gi|405964329|gb|EKC29826.1|	6.15327e-138	Low-density lipoprotein receptor-related protein 4 [Crassostrea gigas
9651560	gi|405964008|gb|EKC29536.1|	6.15908e-100	Homeodomain-interacting protein kinase 2 [Crassostrea gigas
9607224	gi|676494423|ref|XP_009066557.1|	6.16081e-101	hypothetical protein LOTGIDRAFT_108853 [Lottia gigantea]
9627890	gi|676438002|ref|XP_009048386.1|	6.17739e-131	hypothetical protein LOTGIDRAFT_238422 [Lottia gigantea]
9637760	gi|405951123|gb|EKC19063.1|	6.18452e-47	Serine/threonine-protein kinase 10 [Crassostrea gigas]
9644340	gi|676420281|ref|XP_009043661.1|	6.19823e-109	hypothetical protein LOTGIDRAFT_208058 [Lottia gigantea]
9642412	gi|405963514|gb|EKC29079.1|	6.23756e-121	Group XV phospholipase A2 [Crassostrea gigas]
9638878	gi|405965170|gb|EKC30576.1|	6.23886e-98	dead ringer-like protein [Crassostrea gigas]
9653018	gi|405964100|gb|EKC29622.1|	6.24271e-24	Methylcytosine dioxygenase TET1 [Crassostrea gigas
9612346	gi|524875692|ref|XP_005094570.1|	6.25231e-57	PREDICTED: uncharacterized protein LOC101862252, partial [Aplysia californica
9639718	gi|675855046|ref|XP_009013083.1|	6.28634e-77	hypothetical protein HELRODRAFT_74072, partial [Helobdella robusta
9645392	gi|524871901|ref|XP_005092721.1|	6.34221e-136	PREDICTED: major facilitator superfamily domain-containing protein 10-like isoform X2 [Aplysia californic
9588249	gi|676463053|ref|XP_009056472.1|	6.35215e-16	hypothetical protein LOTGIDRAFT_232908 [Lottia gigantea]
9645976	gi|676483309|ref|XP_009062983.1|	6.36947e-155	hypothetical protein LOTGIDRAFT_220749 [Lottia gigantea]
9649120	gi|405975339|gb|EKC39912.1|	6.3714e-30	hypothetical protein CGI_10007764 [Crassostrea gigas]
9636174	gi|405964105|gb|EKC29627.1|	6.4168e-139	Liprin-beta-1 [Crassostrea gigas]
9620880	gi|405970092|gb|EKC35027.1|	6.44376e-123	26S proteasome non-ATPase regulatory subunit 2 [Crassostrea gigas
9630724	gi|405969898|gb|EKC34842.1|	6.45171e-89	Tight junction protein ZO-1, partial [Crassostrea gigas
9641162	gi|641664535|ref|XP_008183043.1|	6.46077e-11	PREDICTED: tigger transposable element-derived protein 6-like [Acyrthosiphon pisur
9634140	gi|676489406|ref|XP_009064924.1|	6.46178e-77	hypothetical protein LOTGIDRAFT_132462 [Lottia gigantea]
9627756	gi|676457877|ref|XP_009054793.1|	6.46919e-64	hypothetical protein LOTGIDRAFT_161209 [Lottia gigantea]
9646278	gi|405975474|gb|EKC40037.1|	6.48287e-171	spinster-like protein 1 [Crassostrea gigas]
9651214	gi|405974583|gb|EKC39217.1|	6.50045e-176	Phosphatidylinositol-3,4,5-trisphosphate 5-phosphatase 2A [Crassostrea gigas
9591477	gi|676431082|ref|XP_009046134.1|	6.50668e-38	hypothetical protein LOTGIDRAFT_237816 [Lottia gigantea]
9610040	gi|405960502|gb|EKC26423.1|	6.51191e-32	Leucine-rich repeat-containing protein 58 [Crassostrea gigas]
9590749	gi|405961471|gb|EKC27269.1|	6.56284e-59	Putative serine carboxypeptidase CPVL [Crassostrea gigas]
9610744	gi|676460671|ref|XP_009055700.1|	6.56955e-80	hypothetical protein LOTGIDRAFT_215894 [Lottia gigantea]
9648424	gi|405971665|gb|EKC36490.1|	6.57129e-138	Vitamin D3 receptor [Crassostrea gigas]
9624812	gi|405971473|gb|EKC36308.1|	6.58473e-100	Cytoplasmic dynein 1 light intermediate chain 1 [Crassostrea gigas
9607482	gi|665808258|ref|XP_008552442.1|	6.59028e-26	PREDICTED: transcription factor HES-1-A [Microplitis demolitor
9642720	gi|524908092|ref|XP_005109153.1|	6.60026e-102	PREDICTED: myotubularin-related protein 10-like [Aplysia californica
9636374	gi|676439650|ref|XP_009048921.1|	6.64621e-51	hypothetical protein LOTGIDRAFT_238569 [Lottia gigantea]
9651686	gi|524882366|ref|XP_005097827.1|	6.65433e-33	PREDICTED: synaptojanin-1-like [Aplysia californica
9646648	gi|568251510|gb|ETN61034.1|	6.68964e-147	calcium binding protein 39 [Anopheles darling
9645274	gi|405959275|gb|EKC25328.1|	6.70869e-37	Serine/threonine-protein phosphatase 4 regulatory subunit 3 [Crassostrea gigas
9628688	gi|676438297|ref|XP_009048483.1|	6.71591e-48	hypothetical protein LOTGIDRAFT_200654 [Lottia gigantea]
9641694	gi|405974500|gb|EKC39140.1|	6.76527e-173	Eukaryotic peptide chain release factor GTP-binding subunit ERF3B [Crassostrea gigas
9643374	gi|405972945|gb|EKC37688.1|	6.79285e-41	DNA-binding protein D-ETS-3 [Crassostrea gigas]
9625496	gi|524869507|ref|XP_005091546.1|	6.81135e-84	PREDICTED: probable phospholipid-transporting ATPase IF-like, partial [Aplysia californic
9650836	gi|405953746|gb|EKC21348.1|	6.81174e-159	Ankyrin-2 [Crassostrea gigas]
9645000	gi|524915840|ref|XP_005112708.1|	6.81441e-26	PREDICTED: deformed epidermal autoregulatory factor 1-like, partial [Aplysia californica
9612614	gi|676465268|ref|XP_009057189.1|	6.81478e-27	hypothetical protein LOTGIDRAFT_121283 [Lottia gigantea]
9612440	gi|242019237|ref|XP_002430069.1|	6.81478e-27	class B secretin-like G-protein coupled receptor GPRcir1, putative [Pediculus humanus corporis
9636548	gi|676478195|ref|XP_009061354.1|	6.81515e-96	hypothetical protein LOTGIDRAFT_234809 [Lottia gigantea
9612462	gi|440896903|gb|ELR48704.1|	6.8306e-19	hypothetical protein M91_18454, partial [Bos mutus
9646154	gi|405952357|gb|EKC20179.1|	6.84104e-72	Protein daughter of sevenless [Crassostrea gigas]
9640182	gi|599127531|gb|AHN53440.1|	6.88154e-79	DDE superfamily endonuclease [Nuttalliella namaqua
9596437	gi|405961919|gb|EKC27652.1|	6.89327e-20	Oxysterol-binding protein-related protein 6 [Crassostrea gigas
9598299	gi|111073721|dbj|BAF02549.1|	6.92427e-55	amino acid transporter [Crassostrea gigas]
9607360	gi|405963352|gb|EKC28935.1|	6.94381e-76	Signal transducing adapter molecule 2 [Crassostrea gigas
9651378	gi|674058575|ref|XP_008835865.1|	6.98325e-24	PREDICTED: zinc finger protein 91-like isoform X2 [Nannospalax galil
9587823	gi|405953210|gb|EKC20915.1|	6.99283e-39	Ras-specific guanine nucleotide-releasing factor RalGPS1 [Crassostrea gigas]
9648494	gi|676448333|ref|XP_009051698.1|	7.00816e-47	hypothetical protein LOTGIDRAFT_104167 [Lottia gigantea]
9571268	gi|524894858|ref|XP_005103920.1|	7.03142e-13	PREDICTED: cytoplasmic dynein 1 intermediate chain 2-like isoform X6 [Aplysia californica
9636504	gi|405976422|gb|EKC40928.1|	7.05258e-93	Proteasome activator complex subunit 4 [Crassostrea gigas
9636712	gi|524911052|ref|XP_005110397.1|	7.054e-125	PREDICTED: translocating chain-associated membrane protein 1-like 1-like isoform X1 [Aplysia californica
9633024	gi|676455691|ref|XP_009054088.1|	7.08356e-73	hypothetical protein LOTGIDRAFT_239451 [Lottia gigantea
9612418	gi|646714439|gb|KDR18401.1|	7.09757e-117	GTP-binding protein 128up [Zootermopsis nevadensis
9648228	gi|524882884|ref|XP_005098078.1|	7.10141e-169	PREDICTED: heat shock 70 kDa protein 13-like [Aplysia californica

9633820	gi|22090632|dbj|BAC06836.1|	7.11888e-52	Se-cadherin, partial [Saccostrea echinata]
9597625	gi|405973719|gb|EKC38414.1|	7.11928e-20	Vasculin-like protein 1 [Crassostrea gigas]
9625322	gi|405952090|gb|EKC19940.1|	7.12899e-17	hypothetical protein CGI_10007269 [Crassostrea gigas]
9629232	gi|676422246|ref|XP_009043933.1|	7.13119e-13	hypothetical protein LOTGIDRAFT_228002 [Lottia gigantea]
9653172	gi|405973814|gb|EKC38506.1|	7.16674e-74	E3 SUMO-protein ligase RanBP2 [Crassostrea gigas]
9628764	gi|221132199|ref|XP_002155458.1|	7.17266e-42	PREDICTED: transmembrane prolyl 4-hydroxylase-like [Hydra vulgaris
9645744	gi|443714549|gb|ELU06913.1|	7.18108e-143	hypothetical protein CAPTEDRAFT_6632 [Capitella teleta
9653306	gi|524867064|ref|XP_005090345.1|	7.18856e-168	PREDICTED: TBC1 domain family member 14-like isoform X3 [Aplysia californica
9618794	gi|405966142|gb|EKC31460.1|	7.20366e-116	TBC1 domain family member 4 [Crassostrea gigas
9642676	gi|405977682|gb|EKC42118.1|	7.21331e-141	Chloride channel protein D [Crassostrea gigas
9606112	gi|405964853|gb|EKC30295.1|	7.23077e-38	Brain protein 16 [Crassostrea gigas]
9614588	gi|405977962|gb|EKC42382.1|	7.23297e-35	Sphingosine-1-phosphate phosphatase 2 [Crassostrea gigas
9644430	gi|405977594|gb|EKC42037.1|	7.27695e-142	hypothetical protein CGI_10028226 [Crassostrea gigas]
9643920	gi|676485010|ref|XP_009063523.1|	7.32156e-115	hypothetical protein LOTGIDRAFT_195725, partial [Lottia gigantea
9569252	gi|6323259|ref|NP_013330.1|	7.32157e-23	Rho family GTPase CDC42 [Saccharomyces cerevisiae S288c]
9643170	gi|676450371|ref|XP_009052357.1|	7.32447e-80	hypothetical protein LOTGIDRAFT_159606 [Lottia gigantea]
9627872	gi|646709550|gb|KDR15350.1|	7.33106e-116	F-box/SPRY domain-containing protein 1 [Zootermopsis nevadensis
9610674	gi|676459486|ref|XP_009055316.1|	7.33118e-47	hypothetical protein LOTGIDRAFT_104663, partial [Lottia gigantea]
9581799	gi|443724550|gb|ELU12510.1|	7.36333e-29	hypothetical protein CAPTEDRAFT_228200 [Capitella teleta
9596663	gi|676429774|ref|XP_009045710.1|	7.36482e-22	hypothetical protein LOTGIDRAFT_180090 [Lottia gigantea]
9639860	gi|405963556|gb|EKC29119.1|	7.36613e-134	dpy-19-like protein 1 [Crassostrea gigas]
9632618	gi|405976530|gb|EKC41034.1|	7.39245e-99	Solute carrier family 23 member 1 [Crassostrea gigas]
9650334	gi|405950535|gb|EKC18517.1|	7.46314e-176	F-box/LRR-repeat protein 7 [Crassostrea gigas]
9623704	gi|524879161|ref|XP_005096259.1|	7.48494e-24	PREDICTED: protein DD3-3-like [Aplysia californica]
9643928	gi|676454037|ref|XP_009053549.1|	7.50666e-144	hypothetical protein LOTGIDRAFT_116428 [Lottia gigantea]
9617148	gi|524909153|ref|XP_005109627.1|	7.50678e-131	PREDICTED: lissencephaly-1 homolog [Aplysia californica
9617294	gi|405953328|gb|EKC21012.1|	7.52421e-123	Protein kinase C delta type [Crassostrea gigas
9603752	gi|524882370|ref|XP_005097829.1|	7.5434e-21	PREDICTED: uncharacterized protein LOC101861888 [Aplysia californica]
9641870	gi|617455595|ref|XP_007570163.1|	7.54957e-63	PREDICTED: E3 ubiquitin-protein ligase MIB2-like [Poecilia formosa
9643570	gi|676483885|ref|XP_009063167.1|	7.56404e-165	hypothetical protein LOTGIDRAFT_220887 [Lottia gigantea
9643030	gi|524913397|ref|XP_005111529.1|	7.57669e-170	PREDICTED: kelch-like ECH-associated protein 1-like [Aplysia californica
9620834	gi|242013973|ref|XP_002427673.1|	7.58068e-23	Signal transducer and activator of transcription 5B, putative [Pediculus humanus corporis
9619322	gi|676466854|ref|XP_009057701.1|	7.58656e-113	hypothetical protein LOTGIDRAFT_233411 [Lottia gigantea]
9649260	gi|260809994|ref|XP_002599789.1|	7.59858e-172	hypothetical protein BRAFLDRAFT_276757 [Branchiostoma floridae]
9627038	gi|676450666|ref|XP_009052453.1|	7.60842e-102	hypothetical protein LOTGIDRAFT_231740 [Lottia gigantea]
9581155	gi|676444868|ref|XP_009050569.1|	7.60881e-63	hypothetical protein LOTGIDRAFT_187185 [Lottia gigantea]
9644512	gi|405957680|gb|EKC23874.1|	7.62136e-99	Inter-alpha-trypsin inhibitor heavy chain H3 [Crassostrea gigas
9584195	gi|564254039|ref|XP_006266015.1|	7.64037e-53	PREDICTED: importin subunit alpha-5-like [Alligator mississippiensis
9641610	gi|676458636|ref|XP_009055040.1|	7.66249e-76	hypothetical protein LOTGIDRAFT_232452 [Lottia gigantea]
9642090	gi|405964043|gb|EKC29565.1|	7.66802e-47	R3H domain-containing protein 2 [Crassostrea gigas
9652092	gi|676429401|ref|XP_009045588.1|	7.68019e-160	hypothetical protein LOTGIDRAFT_136948, partial [Lottia gigantea]
9630850	gi|405975349|gb|EKC39919.1|	7.75623e-50	hypothetical protein CGI_10006642 [Crassostrea gigas]
9622090	gi|676443858|ref|XP_009050240.1|	7.77905e-47	hypothetical protein LOTGIDRAFT_226424 [Lottia gigantea]
9569768	gi|405963464|gb|EKC29034.1|	7.82054e-17	A disintegrin and metalloproteinase with thrombospondin motifs 16 [Crassostrea giga
9617600	gi|405961241|gb|EKC27074.1|	7.8447e-43	Protein BTG3 [Crassostrea gigas]
9643376	gi|405963082|gb|EKC28686.1|	7.85839e-114	Palmitoyltransferase ZDHHC3 [Crassostrea gigas]
9633718	gi|405974577|gb|EKC39211.1|	7.8656e-35	Proton-coupled amino acid transporter 4 [Crassostrea gigas
9581619	gi|405966827|gb|EKC32064.1|	7.86704e-31	Contactin [Crassostrea gigas]
9643320	gi|405955397|gb|EKC22530.1|	7.86852e-98	Protein PRRC1-A [Crassostrea gigas]
9653112	gi|348507723|ref|XP_003441405.1|	7.88846e-86	PREDICTED: inositol polyphosphate 1-phosphatase-like isoform X1 [Oreochromis niloticus
9640412	gi|405958474|gb|EKC24601.1|	7.91273e-99	Sprouty-related, EVH1 domain-containing protein 2 [Crassostrea gigas
9603450	gi|676447333|ref|XP_009051374.1|	7.92964e-79	hypothetical protein LOTGIDRAFT_203023 [Lottia gigantea]
9623370	gi|405960743|gb|EKC26631.1|	7.93593e-26	NEDD4-like E3 ubiquitin-protein ligase WWP1 [Crassostrea gigas]
9647468	gi|405950058|gb|EKC18065.1|	7.94861e-130	Serine/threonine-protein kinase ULK2 [Crassostrea gigas]
9580234	gi|677442616|gb|KFQ38449.1|	7.96792e-49	Importin-5, partial [Mesitornis unicolor
9623500	gi|405967592|gb|EKC32733.1|	7.96849e-127	Transcription elongation factor SPT6 [Crassostrea gigas]
9643060	gi|405974154|gb|EKC38822.1|	7.96897e-111	Solute carrier family 35 member B1 [Crassostrea gigas
9641650	gi|524916005|ref|XP_005112786.1|	8.01448e-49	PREDICTED: mucin-19-like [Aplysia californica
9578522	gi|524917081|ref|XP_005113304.1|	8.04698e-33	PREDICTED: serine/threonine-protein kinase pakG-like isoform X2 [Aplysia californica
9651352	gi|524906470|ref|XP_005108533.1|	8.04705e-105	PREDICTED: archaemetzincin-2-like isoform X1 [Aplysia californica]
9635824	gi|405962690|gb|EKC28341.1|	8.06062e-134	Transcription initiation factor IIB [Crassostrea gigas]
9652412	gi|405967934|gb|EKC33048.1|	8.07008e-155	Atrial natriuretic peptide-converting enzyme [Crassostrea gigas
9645040	gi|676477563|ref|XP_009061149.1|	8.0751e-152	hypothetical protein LOTGIDRAFT_126569, partial [Lottia gigantea]
9618510	gi|321477468|gb|EFX88427.1|	8.09808e-27	guanine nucleotide binding protein, alpha stimulating activity polypeptide [Daphnia pul
9576544	gi|512851710|ref|XP_004887364.1|	8.12148e-38	PREDICTED: WD repeat-containing protein 11 isoform X1 [Heterocephalus glaber
9600610	gi|632946998|ref|XP_007888838.1|	8.13148e-46	PREDICTED: integrin beta-2 isoform X2 [Callorhinchus mili
9602042	gi|443733929|gb|ELU18108.1|	8.16643e-65	hypothetical protein CAPTEDRAFT_219441 [Capitella teleta
9588765	gi|524916043|ref|XP_005112804.1|	8.18048e-19	PREDICTED: piggyBac transposable element-derived protein 4-like [Aplysia californica
9598207	gi|405970853|gb|EKC35720.1|	8.19838e-48	Pleckstrin-like protein domain-containing family M member 2 [Crassostrea gigas
9585877	gi|607365006|gb|EZA59208.1|	8.19925e-37	Copper-transporting ATPase [Cerapachys biroi]

9651298	gi|676440737|ref|XP_009049272.1|	8.22244e-118	hypothetical protein LOTGIDRAFT_158312 [Lottia gigantea]
9643396	gi|269854573|gb|ACZ51335.1|	8.22701e-71	leucine-rich repeat-like protein [Biomphalaria glabrata
9641434	gi|405965411|gb|EKC30788.1|	8.23519e-147	E3 SUMO-protein ligase PIAS1 [Crassostrea gigas]
9577256	gi|676447868|ref|XP_009051549.1|	8.25281e-70	hypothetical protein LOTGIDRAFT_103711, partial [Lottia gigantea]
9645280	gi|676423465|ref|XP_009044109.1|	8.25358e-128	hypothetical protein LOTGIDRAFT_152421 [Lottia gigantea]
9573092	gi|524900018|ref|XP_005106434.1|	8.26337e-14	PREDICTED: microphthalmia-associated transcription factor-like isoform X3 [Aplysia californica
9615492	gi|405974511|gb|EKC39150.1|	8.26383e-95	Calpain-9 [Crassostrea gigas]
9577020	gi|443734794|gb|ELU18651.1|	8.27197e-62	hypothetical protein CAPTEDRAFT_73288, partial [Capitella teleta
9649464	gi|405961346|gb|EKC27164.1|	8.27286e-174	High affinity cAMP-specific and IBMX-insensitive 3',5'-cyclic phosphodiesterase 8B [Crassostrea gigas
9627432	gi|524905794|ref|XP_005108259.1|	8.27857e-88	PREDICTED: dentin sialophosphoprotein-like [Aplysia californica
9602018	gi|405970135|gb|EKC35067.1|	8.31483e-118	Protein argonaute-2 [Crassostrea gigas]
9630502	gi|405965494|gb|EKC30863.1|	8.32528e-89	DnaJ-like protein subfamily B member 11 [Crassostrea gigas
9601192	gi|405950802|gb|EKC18765.1|	8.32796e-14	Cyclin-dependent kinase-like 5 [Crassostrea gigas
9651674	gi|405967897|gb|EKC33016.1|	8.34672e-52	Eukaryotic translation initiation factor 4E transporter [Crassostrea gigas
9646856	gi|405953478|gb|EKC21133.1|	8.36973e-129	Leucine-rich repeat-containing protein 6 [Crassostrea gigas]
9596145	gi|405951983|gb|EKC19845.1|	8.38745e-34	Serine/threonine-protein phosphatase 1 regulatory subunit 10 [Crassostrea gigas
9639184	gi|676439421|ref|XP_009048847.1|	8.40301e-50	hypothetical protein LOTGIDRAFT_158021 [Lottia gigantea
9620570	gi|676453270|ref|XP_009053301.1|	8.40395e-99	hypothetical protein LOTGIDRAFT_214738 [Lottia gigantea]
9598789	gi|405970746|gb|EKC35622.1|	8.40409e-16	Kelch-like protein 29 [Crassostrea gigas]
9581323	gi|147906033|ref|NP_001087367.1|	8.40521e-33	leukocyte receptor cluster (LRC) member 9 [Xenopus laevis]
9645396	gi|390339094|ref|XP_003724927.1|	8.41876e-80	PREDICTED: uncharacterized protein LOC100890479 [Strongylocentrotus purpuratus
9645882	gi|405964507|gb|EKC29983.1|	8.4464e-83	Sphingosine-1-phosphate phosphatase 2 [Crassostrea gigas
9580672	gi|597762380|ref|XP_007245160.1|	8.45164e-46	PREDICTED: U2 snRNP-associated SURP motif-containing protein-like isoform X2 [Astyanax mexicanus
9625622	gi|676477136|ref|XP_009061016.1|	8.48224e-18	hypothetical protein LOTGIDRAFT_165742 [Lottia gigantea]
9597789	gi|405951336|gb|EKC19257.1|	8.484e-45	Potassium voltage-gated channel protein Shaw [Crassostrea gigas
9618328	gi|646706872|gb|KDR13891.1|	8.49563e-61	Metabotropic glutamate receptor 3, partial [Zootermopsis nevadensis
9649304	gi|524906499|ref|XP_005108547.1|	8.51537e-139	PREDICTED: tau-tubulin kinase 1-like [Aplysia californica
9631618	gi|405975658|gb|EKC40212.1|	8.53345e-52	Sphingolipid delta(4)-desaturase DES1 [Crassostrea gigas
9647252	gi|405963792|gb|EKC29338.1|	8.55896e-44	hypothetical protein CGI_10010418 [Crassostrea gigas]
9610242	gi|405966738|gb|EKC31981.1|	8.56276e-48	Eukaryotic translation initiation factor 4 gamma 3 [Crassostrea gigas
9619510	gi|405957421|gb|EKC23632.1|	8.57943e-64	hypothetical protein CGI_10009013 [Crassostrea gigas]
9636770	gi|585713585|ref|XP_006825118.1|	8.5877e-22	PREDICTED: uncharacterized protein LOC102808082 [Saccoglossus kowalevskii
9641718	gi|405972339|gb|EKC37112.1|	8.59445e-51	sprouty-like protein 2 [Crassostrea gigas]
9619470	gi|405974286|gb|EKC38942.1|	8.62465e-16	Hemicentin-1 [Crassostrea gigas]
9646826	gi|405958520|gb|EKC24642.1|	8.65858e-118	Putative protein tag-53 [Crassostrea gigas]
9619418	gi|405978553|gb|EKC42933.1|	8.68015e-109	WD repeat and FYVE domain-containing protein 2 [Crassostrea gigas
9624424	gi|405966142|gb|EKC31460.1|	8.69608e-36	TBC1 domain family member 4 [Crassostrea gigas
9652456	gi|676452634|ref|XP_009053094.1|	8.71058e-157	hypothetical protein LOTGIDRAFT_174957 [Lottia gigantea]
9634268	gi|405971018|gb|EKC35876.1|	8.73845e-90	hypothetical protein CGI_10016017 [Crassostrea gigas]
9643298	gi|405977908|gb|EKC42335.1|	8.75129e-65	PPPDE peptidase domain-containing protein 1 [Crassostrea gigas
9647114	gi|524865134|ref|XP_005089399.1|	8.76199e-158	PREDICTED: WD repeat-containing protein 11-like [Aplysia californica
9609958	gi|405959962|gb|EKC25931.1|	8.78072e-37	hypothetical protein CGI_10009560 [Crassostrea gigas]
9599645	gi|676425791|ref|XP_009044416.1|	8.7869e-21	hypothetical protein LOTGIDRAFT_229985 [Lottia gigantea]
9608824	gi|405974457|gb|EKC39100.1|	8.79878e-74	Collagen type IV alpha-3-binding protein [Crassostrea gigas
9641408	gi|524913601|ref|XP_005111630.1|	8.80634e-133	PREDICTED: phosphatidylinositol 4-phosphate 5-kinase type-1 gamma-like [Aplysia californic
9631828	gi|524885681|ref|XP_005099445.1|	8.80938e-97	PREDICTED: nucleolysin TIA-1-like isoform X7 [Aplysia californica
9610456	gi|676451291|ref|XP_009052657.1|	8.86107e-45	hypothetical protein LOTGIDRAFT_207912 [Lottia gigantea]
9570958	gi|307195801|gb|EFN77615.1|	8.87417e-16	MLX-interacting protein [Harpegnathos saltator
9609866	gi|676425888|ref|XP_009044447.1|	8.91958e-98	hypothetical protein LOTGIDRAFT_230007 [Lottia gigantea]
9629522	gi|524889909|ref|XP_005101506.1|	8.93189e-64	PREDICTED: E3 ubiquitin-protein ligase NEDD4-like [Aplysia californica
9584569	gi|675369816|gb|KFM62718.1|	8.94038e-49	Ran-specific GTPase-activating protein, partial [Stegodyphus mimosarum
9641922	gi|405965484|gb|EKC30853.1|	8.95071e-64	Interleukin-1 receptor-associated kinase 1 [Crassostrea gigas]
9648226	gi|405974733|gb|EKC39357.1|	8.98819e-47	Low-density lipoprotein receptor-related protein 4 [Crassostrea gigas
9647696	gi|676472322|ref|XP_009059455.1|	9.00483e-105	hypothetical protein LOTGIDRAFT_218462 [Lottia gigantea]
9624984	gi|405976478|gb|EKC40983.1|	9.00713e-81	Solute carrier family 28 member 3, partial [Crassostrea gigas
9640888	gi|405961685|gb|EKC27450.1|	9.03949e-66	Solute carrier family 12 member 2 [Crassostrea gigas]
9648898	gi|676427838|ref|XP_009045078.1|	9.05923e-130	hypothetical protein LOTGIDRAFT_136520, partial [Lottia gigantea]
9623792	gi|676462888|ref|XP_009056416.1|	9.07109e-78	hypothetical protein LOTGIDRAFT_162200 [Lottia gigantea]
9631564	gi|524892110|ref|XP_005102586.1|	9.09609e-62	PREDICTED: golgin-45-like [Aplysia californica
9640586	gi|405966878|gb|EKC32110.1|	9.10375e-103	SEC23-interacting protein [Crassostrea gigas]
9640974	gi|405974895|gb|EKC39507.1|	9.13278e-159	Son of sevenless-like protein 2 [Crassostrea gigas]
9639634	gi|405959704|gb|EKC25711.1|	9.1457e-36	Inositol-pentakisphosphate 2-kinase [Crassostrea gigas]
9649798	gi|405978684|gb|EKC43054.1|	9.20752e-152	Collagen alpha-1(IV) chain [Crassostrea gigas]
9649866	gi|405975643|gb|EKC40197.1|	9.27806e-160	Solute carrier family 2, facilitated glucose transporter member 1 [Crassostrea gigas
9625288	gi|405960037|gb|EKC25989.1|	9.28918e-25	Serine/threonine-protein phosphatase 6 regulatory subunit 3 [Crassostrea gigas
9581551	gi|524914673|ref|XP_005112148.1|	9.29305e-32	PREDICTED: uncharacterized protein LOC101853426 isoform X1 [Aplysia californica]
9588219	gi|322783659|gb|EFZ10997.1|	9.29596e-84	hypothetical protein SINV_01057 [Solenopsis invicta
9650966	gi|401834505|gb|AFQ23184.1|	9.29825e-172	TGF-beta type 1 receptor [Azumapecten farreri]
9586973	gi|405976018|gb|EKC40542.1|	9.30125e-20	DnaJ-like protein subfamily C member 7 [Crassostrea gigas
9639862	gi|405959846|gb|EKC25831.1|	9.30165e-20	hypothetical protein CGI_10008361 [Crassostrea gigas]

9647546	gi|405976920|gb|EKC41398.1|	9.32767e-171	Lysine-specific demethylase 2B [Crassostrea gigas]
9623448	gi|405977643|gb|EKC42082.1|	9.37443e-27	CD63 antigen [Crassostrea gigas]
9615922	gi|405961390|gb|EKC27200.1|	9.37732e-38	Smaug-like protein 1 [Crassostrea gigas]
9587715	gi|405967749|gb|EKC32880.1|	9.38556e-60	Catenin alpha-2 [Crassostrea gigas]
9648320	gi|676433451|ref|XP_009046904.1|	9.40638e-20	hypothetical protein LOTGIDRAFT_172073 [Lottia gigantea]
9647688	gi|405952379|gb|EKC20198.1|	9.42072e-171	Receptor-type tyrosine-protein phosphatase R [Crassostrea gigas]
9642224	gi|405950935|gb|EKC18889.1|	9.43765e-37	Wee1-like protein kinase [Crassostrea gigas]
9583607	gi|730368333|gb|KHJ40388.1|	9.44865e-11	TNFR/NGFR cysteine-rich region, partial [Trichuris suis]
9571562	gi|524871230|ref|XP_005092391.1|	9.45692e-18	PREDICTED: beta-hexosaminidase subunit beta-like, partial [Aplysia californica
9636396	gi|405970148|gb|EKC35080.1|	9.46448e-98	Putative protein arginine N-methyltransferase 6.1 [Crassostrea gigas
9602390	gi|676421053|ref|XP_009043769.1|	9.47452e-13	hypothetical protein LOTGIDRAFT_227883 [Lottia gigantea]
9632540	gi|524870876|ref|XP_005092218.1|	9.49567e-22	PREDICTED: uncharacterized protein LOC101864367 isoform X1 [Aplysia californica]
9650336	gi|405971143|gb|EKC35997.1|	9.51199e-30	Ubiquitin carboxyl-terminal hydrolase 10 [Crassostrea gigas
9653240	gi|676449926|ref|XP_009052212.1|	9.54262e-130	hypothetical protein LOTGIDRAFT_143498 [Lottia gigantea]
9644570	gi|524881713|ref|XP_005097506.1|	9.54507e-150	PREDICTED: exocyst complex component 1-like [Aplysia californica
9621482	gi|524915789|ref|XP_005112685.1|	9.54801e-90	PREDICTED: elongation factor Tu, mitochondrial-like [Aplysia californica
9595071	gi|524864134|ref|XP_005088905.1|	9.56075e-43	PREDICTED: uncharacterized protein LOC101862840 [Aplysia californica]
9589909	gi|524888069|ref|XP_005100611.1|	9.59955e-63	PREDICTED: uncharacterized protein LOC101856271 [Aplysia californica]
9643694	gi|260808745|ref|XP_002599167.1|	9.6018e-67	hypothetical protein BRAFLDRAFT_68754 [Branchiostoma floridae]
9646264	gi|405963537|gb|EKC29101.1|	9.60313e-82	Solute carrier family 12 member 9 [Crassostrea gigas]
9611564	gi|241739707|ref|XP_002405143.1|	9.6547e-95	endosomal membrane protein EMP70, putative [Ixodes scapularis
9653416	gi|405962832|gb|EKC28475.1|	9.67425e-36	AF4/FMR2 family member 4 [Crassostrea gigas]
9648844	gi|405963516|gb|EKC29081.1|	9.68242e-108	Ras-responsive element-binding protein 1, partial [Crassostrea gigas
9626062	gi|676461482|ref|XP_009055958.1|	9.68604e-70	hypothetical protein LOTGIDRAFT_119445 [Lottia gigantea]
9639680	gi|524864217|ref|XP_005088946.1|	9.69843e-78	PREDICTED: polycystic kidney disease protein 1-like 2-like [Aplysia californica
9603702	gi|405964531|gb|EKC30006.1|	9.71583e-69	Sodium- and chloride-dependent glycine transporter 2 [Crassostrea gigas
9635314	gi|405969211|gb|EKC34194.1|	9.72863e-108	Calcium-binding mitochondrial carrier protein Aralar1 [Crassostrea gigas
9578874	gi|675853156|ref|XP_009012138.1|	9.74306e-55	hypothetical protein HELRODRAFT_104956 [Helobdella robusta]
9583999	gi|525027218|ref|XP_005061040.1|	9.75517e-16	PREDICTED: CDC42 small effector protein 2 isoform X1 [Ficedula albicollis
9633560	gi|443689881|gb|ELT92172.1|	9.78298e-70	hypothetical protein CAPTEDRAFT_182891 [Capitella teleta
9601584	gi|405973446|gb|EKC38161.1|	9.81937e-71	Bardet-Biedl syndrome 1 protein [Crassostrea gigas
9626214	gi|685284579|ref|XP_009133195.1|	9.86314e-147	PREDICTED: actin-1-like [Brassica rapa]
9632676	gi|676473936|ref|XP_009059982.1|	9.88138e-19	hypothetical protein LOTGIDRAFT_153999 [Lottia gigantea]
9641666	gi|405969694|gb|EKC34648.1|	9.88297e-132	Serine/threonine-protein phosphatase 4 regulatory subunit 1 [Crassostrea gigas
9622000	gi|405963886|gb|EKC29418.1|	9.92684e-127	Protein roadkill [Crassostrea gigas]
9646172	gi|403310251|emb|CCJ09600.1|	9.94741e-47	Nacrein-like 3 protein, partial [Patella vulgata
9649324	gi|405977199|gb|EKC41661.1|	9.94834e-63	capicua-like protein [Crassostrea gigas]
9628378	gi|524868201|ref|XP_005090905.1|	9.95216e-28	PREDICTED: piggyBac transposable element-derived protein 4-like [Aplysia californica
