# Supplementary material for: Biodiversity in marine invertebrate responses to acute warming revealed by a comparative multi‐omics approach
Source: Glob Chang Biol. 2016 Jun 17;23(1):318–30. doi: 10.1111/gcb.13357 (PMC6849730; doi:10.1111/gcb.13357)
Supplement: Supplementary file 8 — Data S1. Detail on PCA and PLS‐DA analyses. [file GCB-23-318-s008.pdf]

Supplemental material S1: Detail on PCA and PLS-DA analyses

Methods are described in the main paper. Analyses were carried out on 11 different tissues from 6 Antarctic marine invertebrate species (Table 1).

Table 1: Sample codes that were used while carrying out NMR analysis

Animal	Tissue	Code used
Laternula elliptica	Siphon	LeS
	Mantle	LeC
	Foot	LeF
	Gill	LeG
	Digestive gland	LeD
Liothyrella uva	Whole animal	Lu
Paraceradocus miersi	Whole animal	Pm
Aequiyoldia eightsii	Foot	AeF
	Rest of the animal	AeR
Marseniopsis mollis	Whole animal	Mm
Cucumaria georgiana	Whole animal	Cg

At the end of the code was an additional letter either “C” (control) or “T” (treated).

The 220 pJRES spectra were split according to controls and treated tissue of each species as shown in Table 2.

Table 2: The PCA models produced

Animal	Tissue	PCA
Laternula elliptica	Siphon	LeSC vs LeST
	Mantle	LeMC vs LeMT
	Foot	LeFC vs LeFT
	Gill	LeGC vs LeGT
	Digestive gland	LeDC vs LeDT
Liothyrella uva	Whole animal	LuC vs LuT
Paraceradocus miersi	Whole animal	PmC vs PmT
Aequiyoldia eightsii	Foot	AeFC vs AeFT
	Rest of the animal	AeRC vs AeRT
Marseniopsis mollis	Whole animal	MmC vs MmT
Cucumaria georgiana	Whole animal	CgC vs CgT

The PCA models of these groups were constructed, with the number of components chosen automatically by the PLS Toolbox algorithm. The classes to be separated were based on controls and treatment groups of these animal tissues. To help interpret the PCA model, the principal component (PC) scores were tested using an in-house developed matlab script (pca_scores_test_v12.m), which performs a T-test on the scores for each PC. These scores were not corrected for multiple testing at this preliminary stage.

<u>**Results**</u>

LeSC vs. LeST

A 3 PC PCA model was constructed for *Laternula elliptica* siphon tissue (**Figure 1-2)**. The percentages of variance of PCs 1-3 were 30.34, 17.09 and 13.52% respectively.

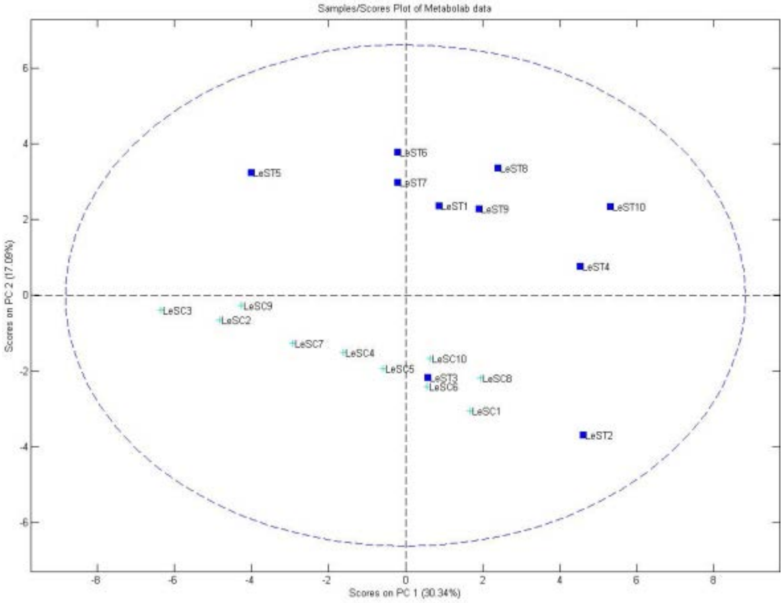

Figure 1: LeSC vs. LeST, PC1 against PC2

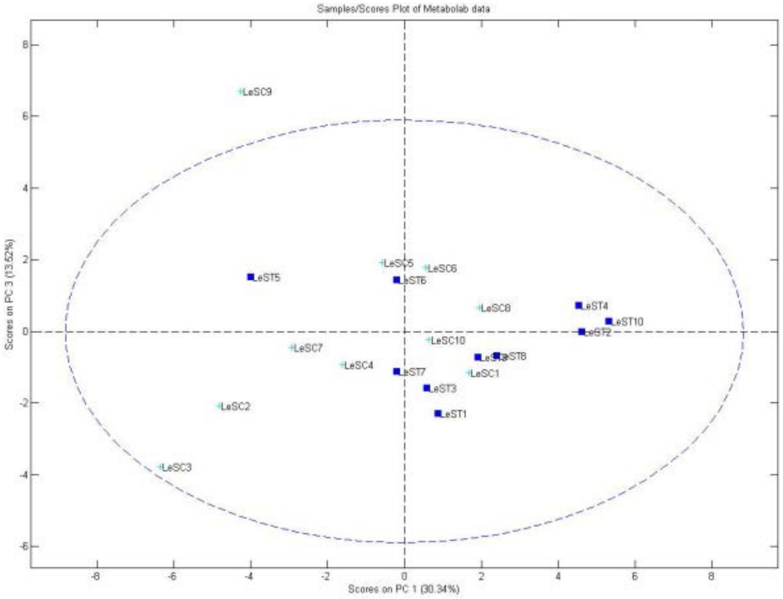

Figure 2: LeSC vs. LeST, PC1 against PC3

The PC scores results were subjected to Students t-test:

PC1 p-value = 0.0241 (30.34% of variance)

PC2 p-value = 0.00198 (17.09% of variance)

PC3 p-value = 0.627 (13.52% of variance)

The t-test for the PC2 scores data confirmed there was a significant separation between the control and treated samples. Also, the PC1 scores data was potentially significant, however, when corrected this may not be significant. The PC2 scores data was further investigated by identifying the metabolites from the loadings plot, **Figure 3**.

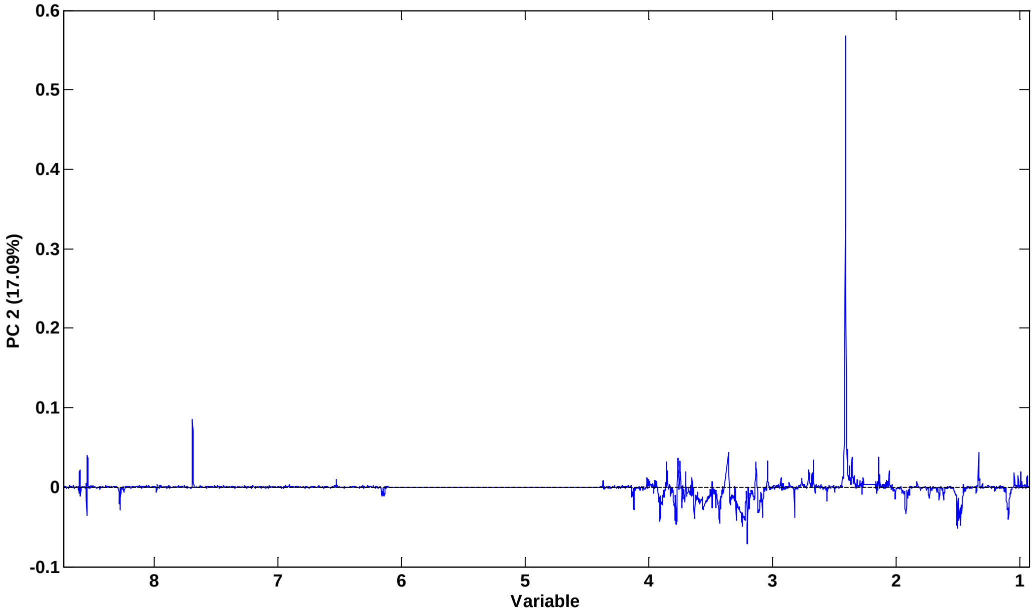

Figure 3: PC2 loadings plot

The first 25 highest loadings were used to identify the metabolites that were causing separation between the control and treated samples of *L. elliptica* siphon tissues seen for PC2, **Table 3**.

Table 3: Metabolites identified from the first 25 highest loadings

δ_H	Metabolite
1.33	Lactate
1.50	Alanine
2.14	Methionine/glutamate*
2.41	Succinate
3.20	Carnitine
3.24	Arginine
3.42	Taurine
3.77	Glutamate

*Overlap of signals

Table 4: Fold change and adjusted p-value for the metabolites

Metabolite	P-value	Fold change (T/C)
Lactate	0.14	1.27
Alanine	0.85	1.03
Succinate	0.08	5.42

Carnitine	0.85	1.05
Arginine	0.85	0.97
Taurine	0.49	0.82
Glutamate	0.85	1.08

A two tailed t-test, at 0.05, was performed on the peak areas of the metabolites identified in **Figure 4**. These peak areas were fitted and measured using the Chenomx software and normalized to the TMSP reference peak within each sample. The P values were adjusted for multiple testing using Benjamini-Hochberg's method (this procedure was used throughout this study for the two tailed t-test). From the adjusted p-value (Benjamini-Hochberg), the seven metabolites showed no significant differences between the control and treated samples of *L. elliptica* siphon tissues, **Table 4**.

PLS-DA was optimised to produce a model using 3 LVs and 45 bins. The permutation testing of the PLS-DA model using all the variables (100 permutations), produced an average class error of 15.01% which showed a significant separation of the data, P value close to 0. The permutation testing of the PLS-DA model, using reduced number of variables (40, 100 permutations), produced an average class error of 8.15% , which showed significant separation of the reduced number of variables, P value close to 0. This model using reduced number of variables was investigated further by identifying the metabolites in **Figure 4**

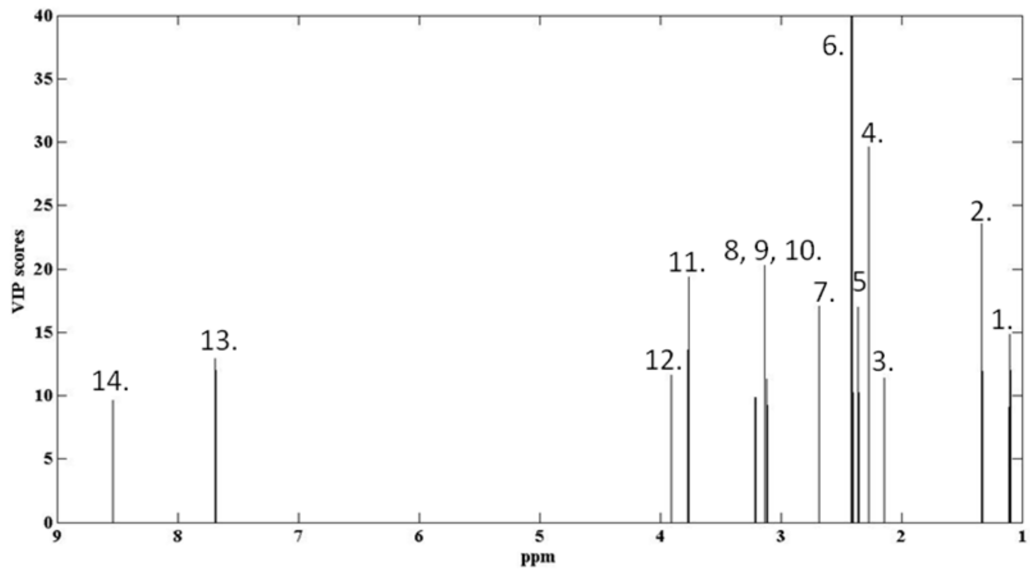

Figure 4: VIP scores for 45 forward selected bins in a 3 LV model of LeSC vs. LeST. The metabolites identified are (1) unknown metabolite, 1.10 ppm, (2) lactate, (3) methionine, (4) acetoacetate, (5) glutamate, (6) succinate, (7) aspartate, (8) malonate, (9) dimethyl sulfone, (10) carnitine, (11) glutamate, (12) betaine, (13) unknown metabolite, 7.68 ppm and (14) homarine

The VIP scores showed succinate had the highest score of 608, which suggests that this metabolite contributes mainly to the separation of the control and treated samples of *L. elliptica*

siphon tissues (for visualisation purposes the VIP scores range was alternated, **Figure 4**). Metabolite 3 and 11 were overlapping signals and to identify them all the signals for each of the metabolite were checked to see if they also have a VIP score. This aided in the tentative identification for metabolite 3 and 11. The resonance at 7.68 ppm was seen as a weak intensity signal in the ^{1}H NMR spectra and the resonance at 1.10 ppm is an unknown metabolite.

A two tailed t-test, at 0.05, was performed on the metabolites identified in **Figure 4**. From the adjusted p-value (Benjamini-Hochberg), the metabolites showed no significant differences between the control and treated samples of *L. elliptica* siphon tissues, **Table 5**.

Table 5: Metabolites identified from the reduced bins

Peak ID	P value	Fold change (T/C)
Acetoaceate	0.52	0.65
Aspartate	0.43	1.34
Betaine	0.1	1.48
Carnitine	0.85	1.07
Dimethyl sulfone	0.77	0.69
Glutamate	0.52	0.41
Homarine	0.1	1.48
Lactate	0.29	1.21
Malonate	0.97	1.01
Methionine	0.1	1.47
Succinate	0.12	3.13

LeMC vs. LeMT

A 3 PC PCA model was constructed for *Laternula elliptica* mantle tissue (**Figures 5-6**). The percentages of variance of PCs 1-3 were 31.50, 15.59 and 12.02% respectively.

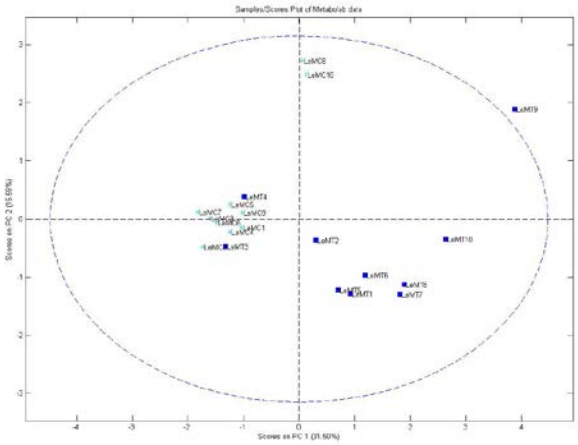

Figure 5: LeMC vs LeMT, PC1 against PC2

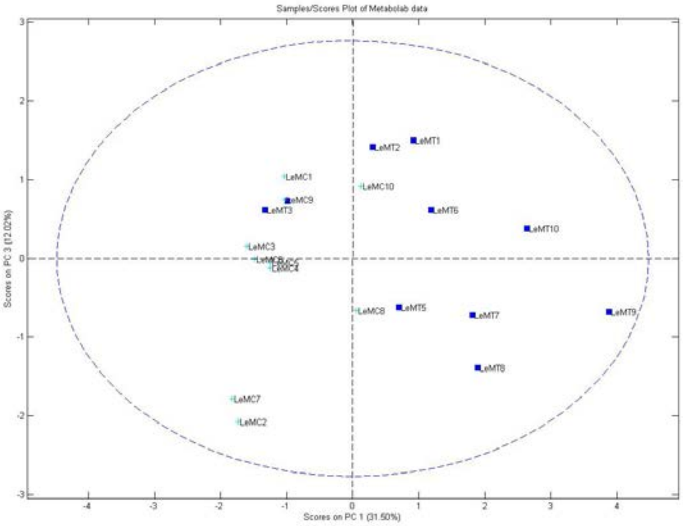

Figure 6: LeMC vs LeMT, PC1 against PC3

The PC scores results were subjected to Students t-test:
PC1 p-value = 0.0007 (31.50% of variance)
PC2 p-value = 0.060 (15.59% of variance)
PC3 p-value = 0.432 (12.02% of variance)
The t-test for the PC1 scores data confirmed there was a significant separation between the control and treated samples of *L. elliptica* mantle tissues. This was further investigated by identifying metabolites from the PC1 loadings plot, **Figure 7**.

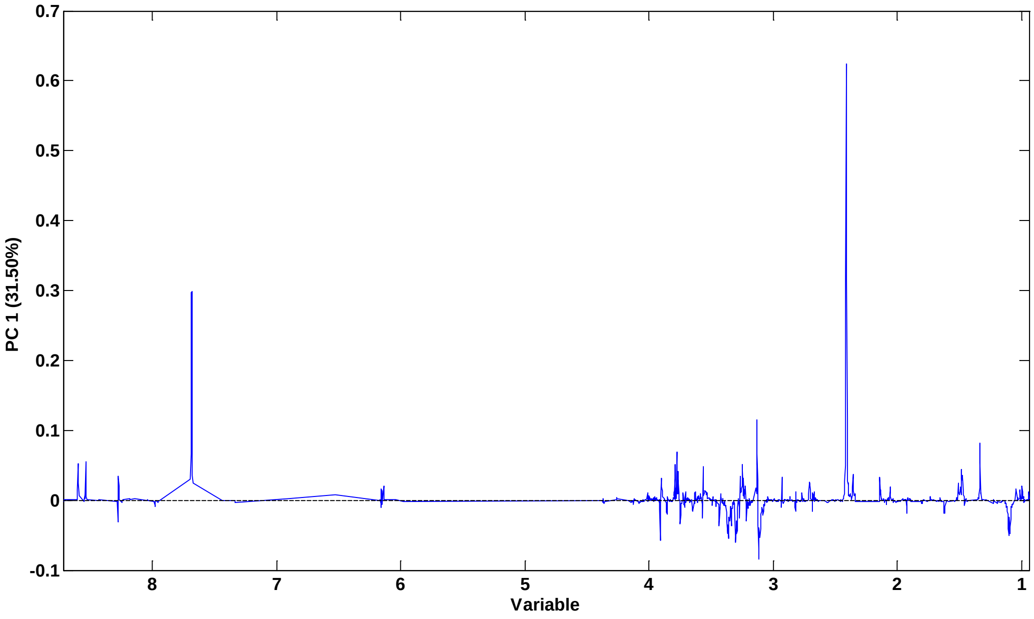

Figure 7: PC1 loadings plot

The first 25 highest loadings were used to identify the metabolites that were causing separation between the control and treated samples of *L. elliptica* mantle tissues samples seen for PC1 loadings plot, **Table 6**.

Table 6: Metabolites identified from the first 25 highest loadings

δ_H	Metabolite
1.33	Lactate
2.41	Succinate
3.11	Malonate
3.77	Glutamate
3.91	Betaine
8.53	Homarine
8.60	AMP

Table 7: Fold change and adjusted p-value for the metabolites

Metabolite	P-value	Fold change (T/C)
**Lactate**	**0.01**	**1.42**
Betaine	0.065	1.03
**Succinate**	**0**	**14.63**
Arginine	0.51	1.12
Malonate	0.16	0.82

A two tailed t-test, at 0.05, was performed on the metabolites identified, **Table 6**. From the adjusted p-value (Benjamini-Hochberg), succinate and lactate were found to be significantly different between the control and treated samples of *L. elliptica* mantle tissues, **Table 7**.

PLS-DA was optimised to produce a model using 2 LVs and 40 bins. The permutation testing of the PLS-DA model using all the variables (1000 permutations), produced an average class error of 12.74%, which showed significant separation the data, P value of 0.2. The permutation testing of the PLS-DA model using the reduced number of variables (40, 1000 permutations), produced an average class error of 10.66%, which showed significant separation of the reduced number of variables, P value close to 0. The model using reduced number of variables was further investigated by identifying the metabolites in **Figure 8**.

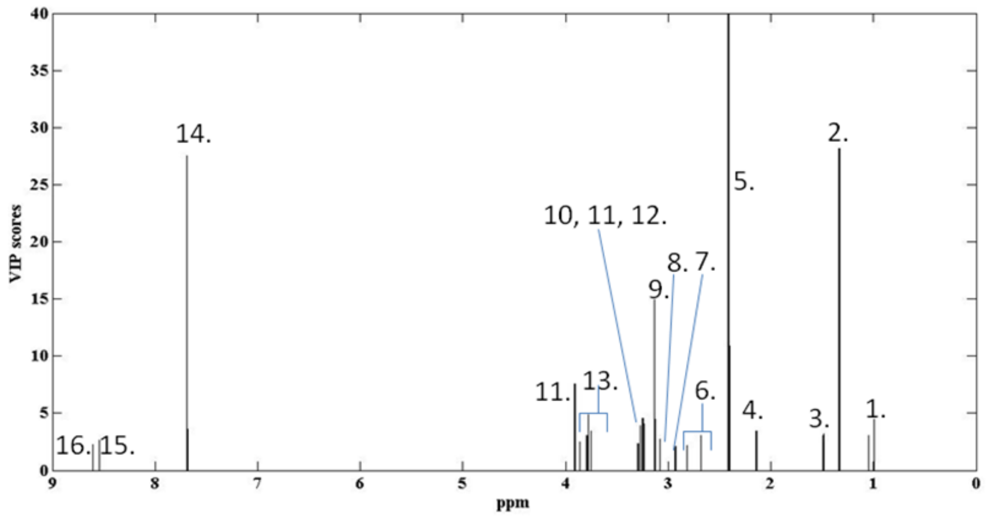

Figure 8: VIP values for 40 forward selected bins in a 2 LV model of LeMC vs. LeMT. The metabolites identified are (1) valine, (2) lactate, (3) alanine, (4) methionine, (5) succinate, (6) aspartate, (7) dimethylglycine, (8) unknown metabolite, (9) dimethyl sulfone, (10) carniite, (11) betaine, (12) arginine, (13) unknown metabolite, (14) unknown metabolite, (15) homarine and (16) unknown metabolite

The VIP scores showed that succinate had the highest score of 353, which suggested that this contributes mainly to the separation of the control and treated samples of *L. elliptica* mantle tissues (for visualisation purposes the VIP scores range was alternated, **Figure 8**). Metabolites 8, 13, 14 and 16 were not identified either due to complex overlapping signals or the signal intensity being weak.

A two tailed t-test, at 0.05, was performed on the metabolites identified in **Figure 8**. From the adjusted p-value (Benjamini-Hochberg), lactate, succinate and valine were found to be significantly different between the control and treated samples of *L. elliptica* mantle tissues, **Table 8**.

Table 8: Metabolites identified from the VIP scores

Peak ID	P-value	Fold change (T/C)
Alanine	0.56	1.07
Arginine	0.53	1.12
Aspartate	0.13	0.73
Betaine	0.78	1.03
Carnitine	1	1
Dimethyl glycine	0.53	2
Dimethyl sulfone	0.52	1.14
Homarine	0.52	0.57

**Lactate**	**0.01**	**1.42**
Methionine	0.8	1.05
**Succinate**	**0**	**14.63**
**Valine**	**0.01**	**1.6**

LeFC vs. LeFT

A 5 PC PCA model was constructed for *Laternula elliptica* foot tissue (**Figure 9-12**). The percentages of variance of PCs 1-5 were 38.09, 16.33, 9.35, 6.50 and 5.54% respectively.

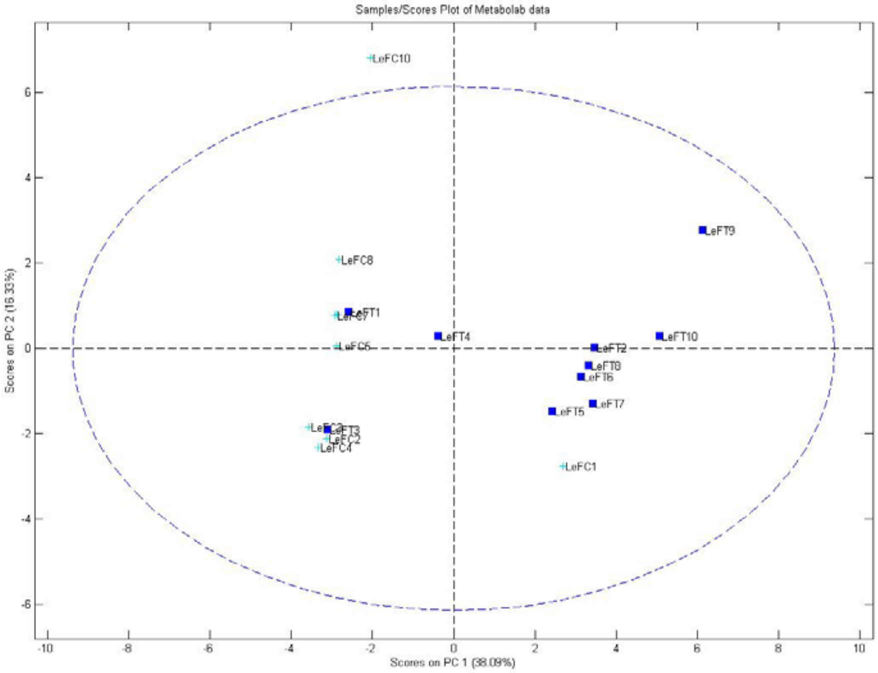

Figure 9: LeFC vs. LeFT, PC1 against PC2

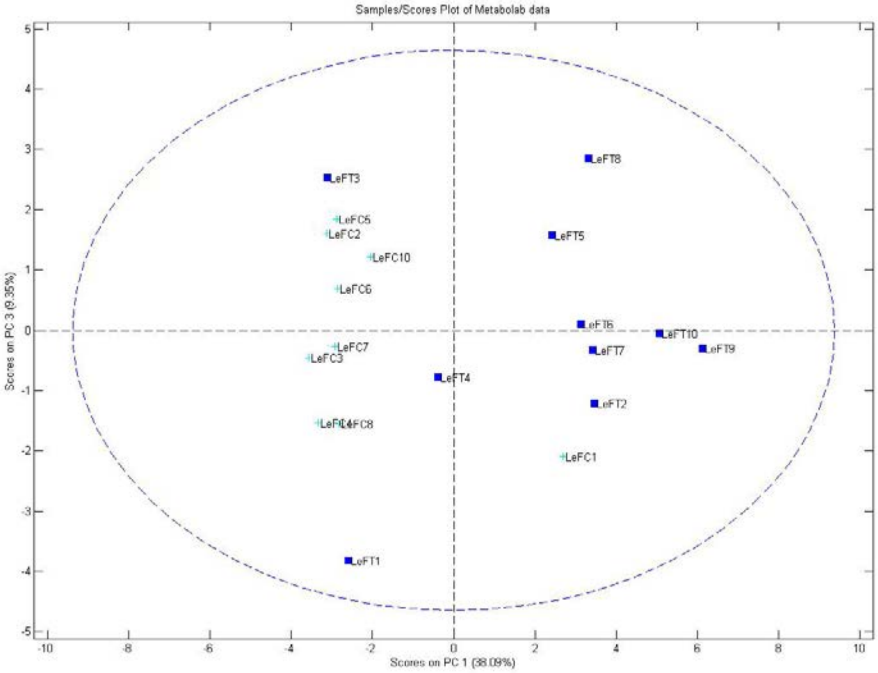

Figure 10: LeFC vs. LeFT, PC1 against PC3

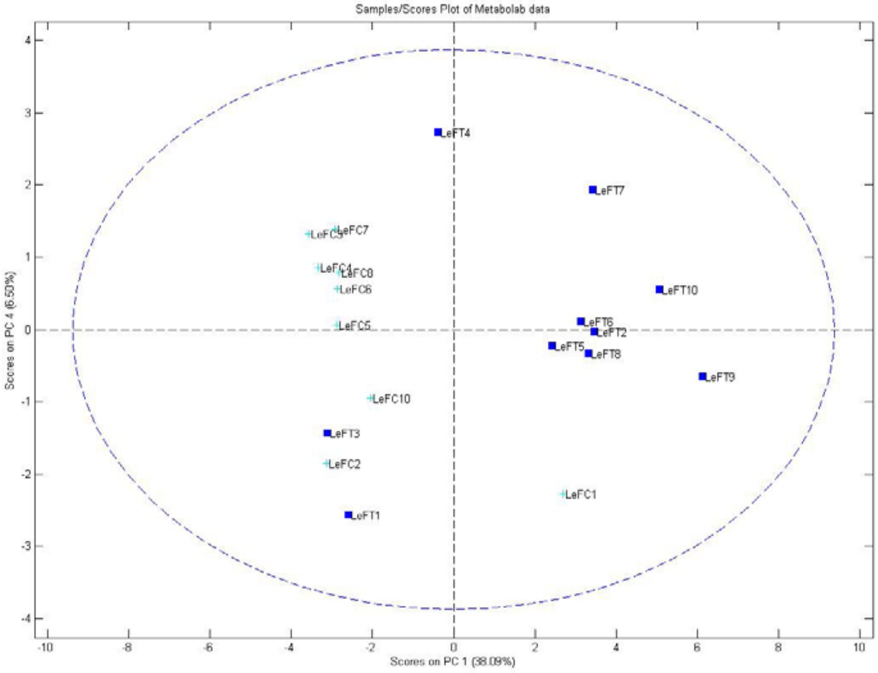

Figure 11: LeFC vs. LeFT, PC1 against PC4

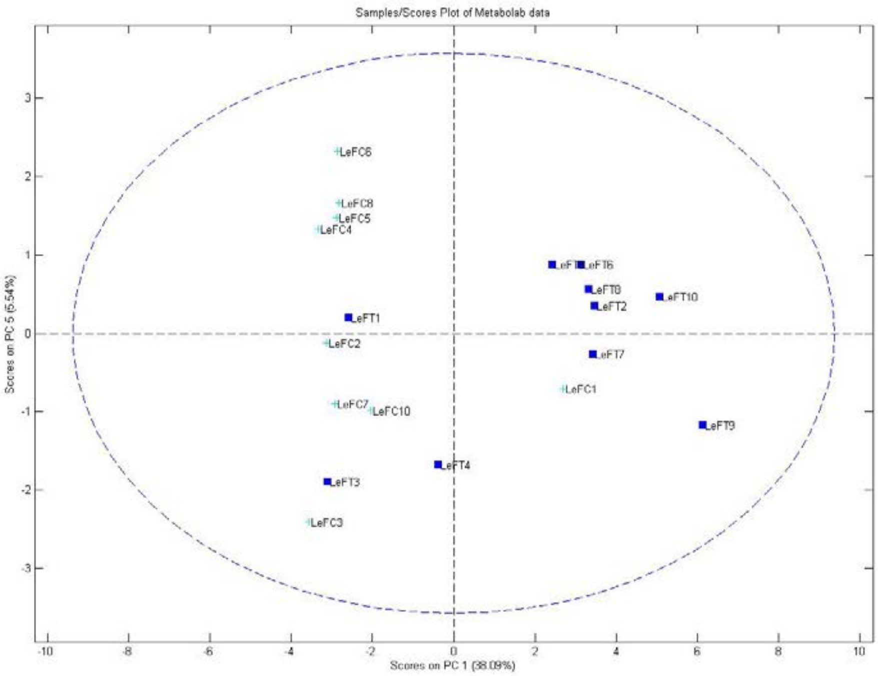

Figure 12: LeFC vs. LeFT, PC1 against PC5

The PC scores results were subjected to Students t-test:
PC1 p-value = 0.002 (38.09% of variance)
PC2 p-value = 0.774 (16.33% of variance)
PC3 p-value = 0.886 (9.35% of variance)
PC4 p-value = 0.979 (6.50% of variance)
PC5 p-value = 0.571 (5.54% of variance)
The t-test for the PC 1 scores data confirmed there was a significant separation between the control and treated samples of *L. elliptica* foot tissues. This was further investigated by identifying metabolites from the loadings plot, **Figure 13**.

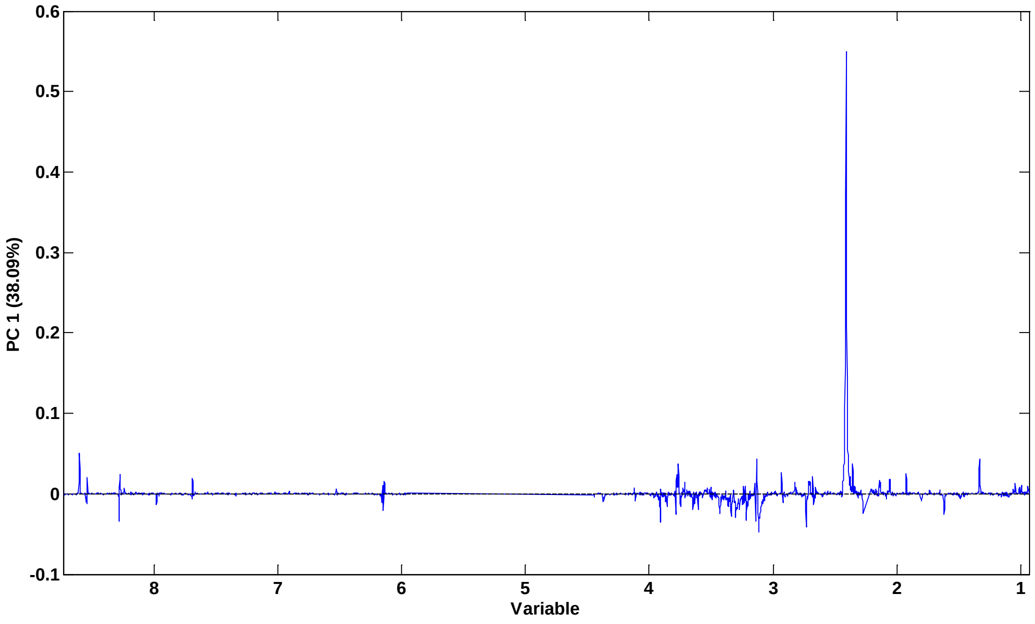

Figure 13: PC1 loadings plot

The first 25 highest loadings were used to identify the metabolites that were causing separation between the control and treated samples of *L. elliptica* foot tissues seen for PC1 loadings plot, **Table 9**.

Table 9: Metabolites identified from the first 25 highest loadings

δ_H	Metabolite
2.41	Succinate
3.11	Malonate
3.13	Dimethyl sulfone
1.33	lactate
3.76	Arginine
3.90	Betaine

Table 10: Fold change and adjusted p-value for the metabolites

Metabolite	P-value	Fold change (T/C)
**Succinate**	**0**	**25.84**
Malonate	0.98	1.01
Lactate	0.28	1.32
Arginine	0.63	0.83
Betaine	0.20	0.89

A two tailed t-test, at 0.05, was performed on the metabolite identified, **Table 9**. From the adjusted p-value (Benjamini-Hochberg), succinate was found to be significantly different between the control and treated samples of *L. elliptica* foot tissue, **Table 10**.

PLS-DA was optimised to produce a model using 1 LV and 100 bins. T he permutation testing of the PLS-DA model using all the variables (100 permutations), produced an average class error of 22.92%, which showed a significant separation of the data, P value of 0.01. The permutation testing of the PLS-DA model using reduced number of variables (100, 100 permutations), produced an average class error of 20.03% , which showed a significant separation of the reduced number of variables, P values close to 0. The model using reduced number of variables was further reinvestigated by identifying the metabolites in **Figure 14**.

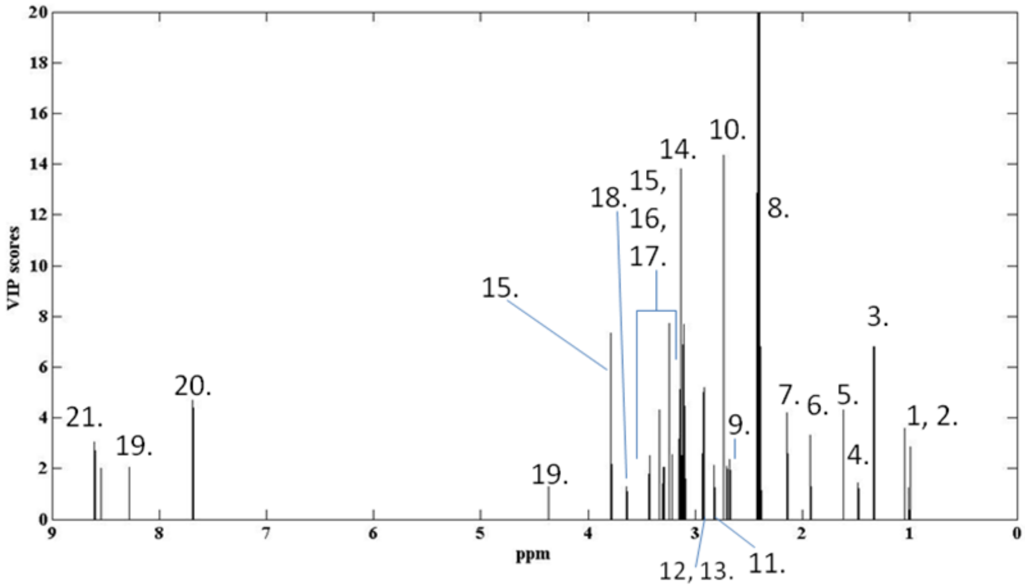

Figure 14: VIP values for 100 forward selected bins in a 1 LV model of LeFC vs. LeFT. The metabolites identified are (1) valine, (2) isoleucine, (3) lactate, (4) alanine, (5) unknown metabolite, (6) acetate, (7) methionine, (8) succinate, (9) aspartate, (10) unknown metabolite, (11) methylguanidine, (12) unknown metabolite, (13) dimethylglycine, (14) malonate, (15) arginine, (16) unknown metabolite, (17) taurine, (18) unknown metabolite, (19) homaraine, (20) unknown metabolite and (21) unknown metabolite

The VIP scores shows that succinate had the highest score of 852, which suggests that this contributes majorly to the separation of the control and treated samples of *L. elliptica* foot tissue (for visualisation purposes the VIP scores range was alternated, **Figure 14**). Metabolites 5, 10, 12, 16, 18, 20 and 21 were not identified either due to complex overlapping signals or the signal intensity being weak. To identify these further separations of the samples, using SPE methods, is required.

A two tailed t-test, at 0.05, was performed on the metabolites identified in **Figure 14**. From the adjusted p-value (Benjamini-Hochberg), acetate was found to be significantly different between the control and treated samples of *L. elliptica* mantle tissues, **Table 11**.

Table 11: Metabolites identified from the VIP scores

Peak ID	P-value	Fold change (T/C)
Acetate	0.66	0.8
Alanine	0.91	1.03
Arginine	0.73	0.83
Aspartate	0.66	0.82
Carnitine	0.66	1.86
Dimethyl sulfone	0.73	1.09
Dimethylglycine	0.65	0.34
Homarine	0.66	0.46
Isoleucine	0.98	0.97
Lactate	0.66	1.32
Malonate	0.98	1.01
Methionine	0.73	0.64
Methyl guanidine	0.66	0.53
**Succinate**	**0.01**	**25.84**
Taurine	0.67	0.93
Valine	0.58	1.65

LeGC vs. LeGT

A 5 PC PCA model was constructed for *Laternula elliptica* gill tissue (**Figure 15-16**). The percentages of variance of PCs 1-3 were 40.93, 6.88 and 11.99% respectively.

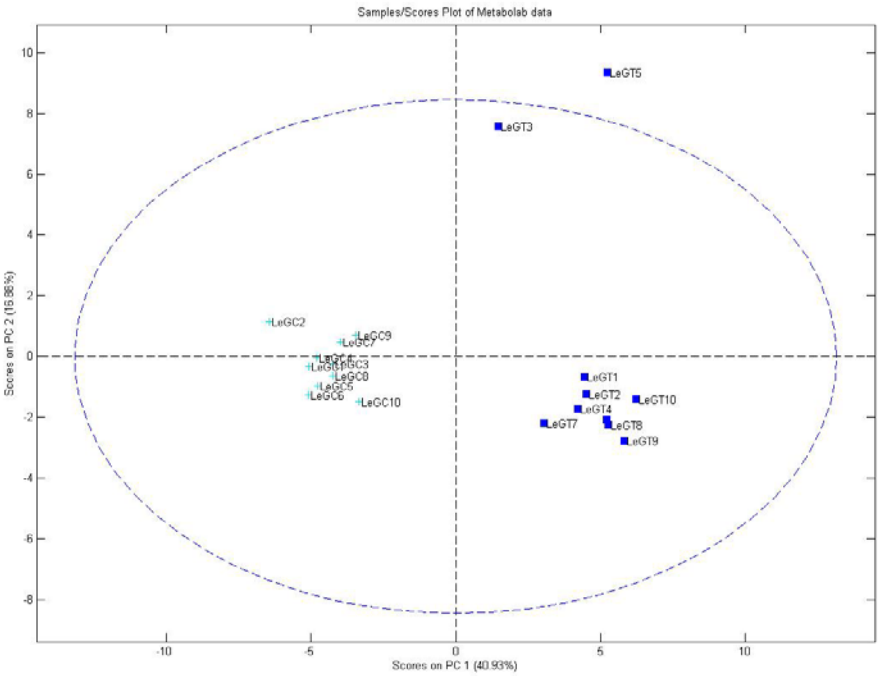

Figure 15: LeGC vs. LeGT, PC1 against PC2

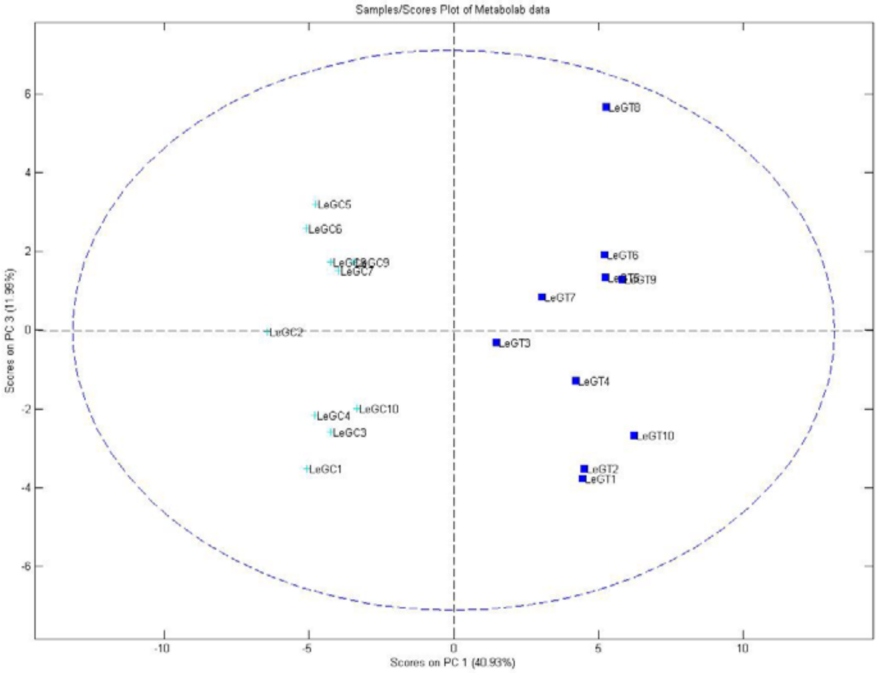

Figure 16: LeGC vs. LeGT, PC1 against PC3

The PC scores results were subjected to Students t-test:
PC1 p-value = 1.273e-12 (40.93% of variance)
PC2 p-value = 0.714 (6.88% of variance)
PC3 p-value = 0.935 (11.99% of variance)
The t-test for the PC 1 scores data confirmed there was a significant separation between the control and treated samples of *L. elliptica* gill tissues. This was further investigated by identifying metabolites from the loadings plot, **Figure 17**.

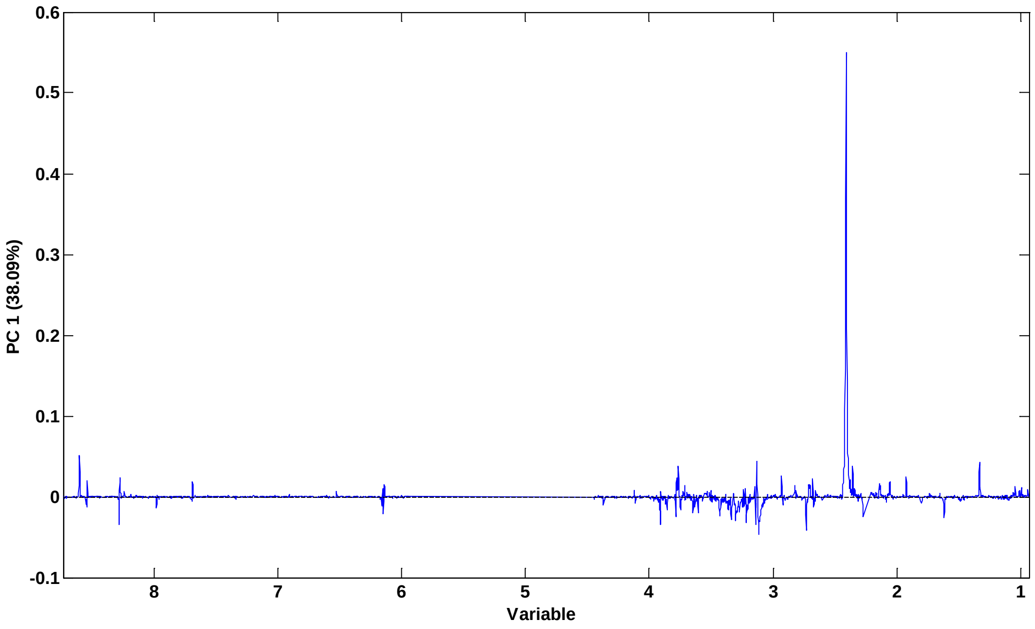

Figure 17: PC1 loadings plot

The first 25 highest loadings were used to identify the metabolites that were causing separation between the control and treated samples of *L. elliptica* gill tissues seen in PC1 loadings plot, **Table 12**.

Table 12: Metabolites identified from the first 25 highest loadings

δ_H	Metabolite
1.48	Alanine
1.33	Lactate
2.41	Succinate

Table 13: Fold change and adjusted p-value for the metabolites

Metabolite	P-value	Fold change (T/C)
Alanine	0	2.37
Lactate	0	4.15
Succinate	0	86.57

A two t-tailed test, at 0.05, was performed on the metabolites identified, **Table 12**. From the adjusted p-value (Benjamini-Hochberg), alanine, lactate and succinate was found to be significantly different between the control and treated samples of *L. elliptica* gill tissues, **Table 13**.

PLS-DA was optimised to produce a model using 3 LVs and 75 bins. The permutation testing of the PLS-DA model using all the variables (100 permutations), produced an average class error of 0.15%, which shows a significant separation of the data, P value close to 0. The permutation

testing of the PLS-DA model using reduced number of variables (75, 100 permutations), produced an average class error of 0.5%, which showed a significant separation of the reduced number of variables, P value close to 0. The model using reduced number of variables was further investigated by identifying the metabolites in **Figure 18**.

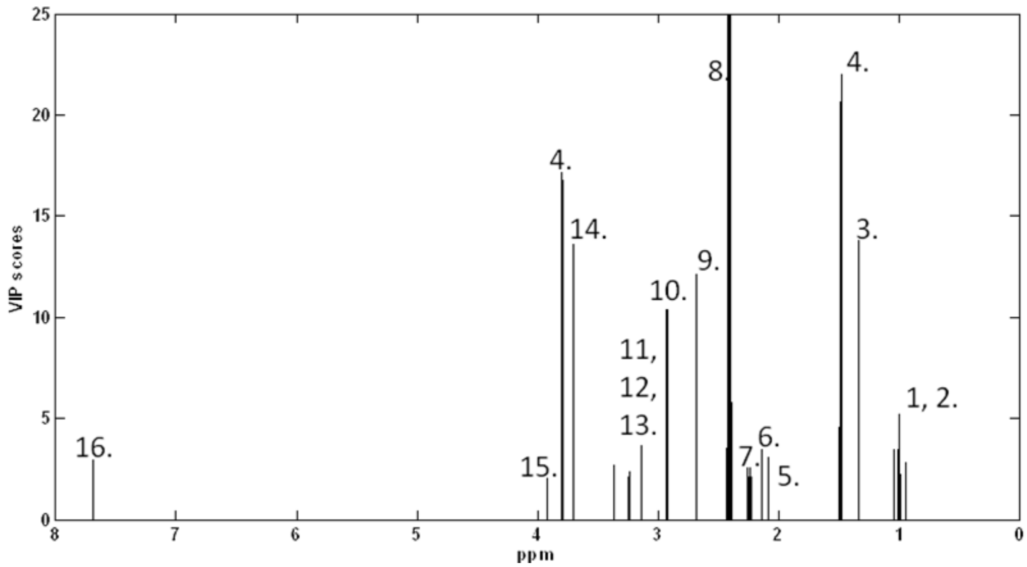

Figure 18: VIP values for 75 forward selected bins in a 3 LV model of LeGC vs. LeGT. The metabolites identified are (1) isoleucine, (2) valine, (3) lactate, (4) alanine, (5) unknown metabolite, (6) methionine, (7) acetone (8) succinate, (9) aspartate, (10) dimethyl sulfone, (11) unknown metabolite, (12) methanol, (13) unknown metabolite (14) unknown metabolite, (15) betanine and (16) unknown metabolite

The VIP scores showed that succinate had the highest score of 385, which suggests that this contributes mainly to the separation of the control and treated samples of *L elliptica* gill tissue (for visualisation purposes the VIP scores range was alternated, **Figure 18**). Methanol was used during the extraction of the samples, which means the methanol was not completely removed during the drying stage and when processing the data. Metabolites 5, 11, 13, 14 and 16 were not identified either due to complex overlapping signals or the signal intensity being weak. To identify these further separations of the samples, using SPE methods, is required.

A two tailed, t-test, at 0.05, was performed on the metabolites identified in **Figure 8**. From the adjusted p-value (Benjamini-Hochberg), acetone, alanine, asparatate, lacetate, succinate and valine were found to be significantly different between the control and treated samples of *L. elliptica* mantle tissues, **Table 13**.

Table 13: **Metabolites identified from the VIP scores**

Peak ID	P-value	Fold change (T/C)
Acetone	0.04	0.61
Alanine	0	2.45
Aspartate	0.01	0.55

Dimethylsulfone	0.27	0.79
Isoleucine	0.43	1.98
**Lactate**	**0**	**4.07**
Methionine	0.27	2.98
**Succinate**	**0**	**94.73**
**Valine**	**0**	**3.84**

LeDC vs. LeDT

A 2 PC PCA model was constructed for *Laternula elliptica* digestive gland tissue (**Figure 19**). The percentages of variance of PCs 1-2 were 53.15 and 11.59% respectively.

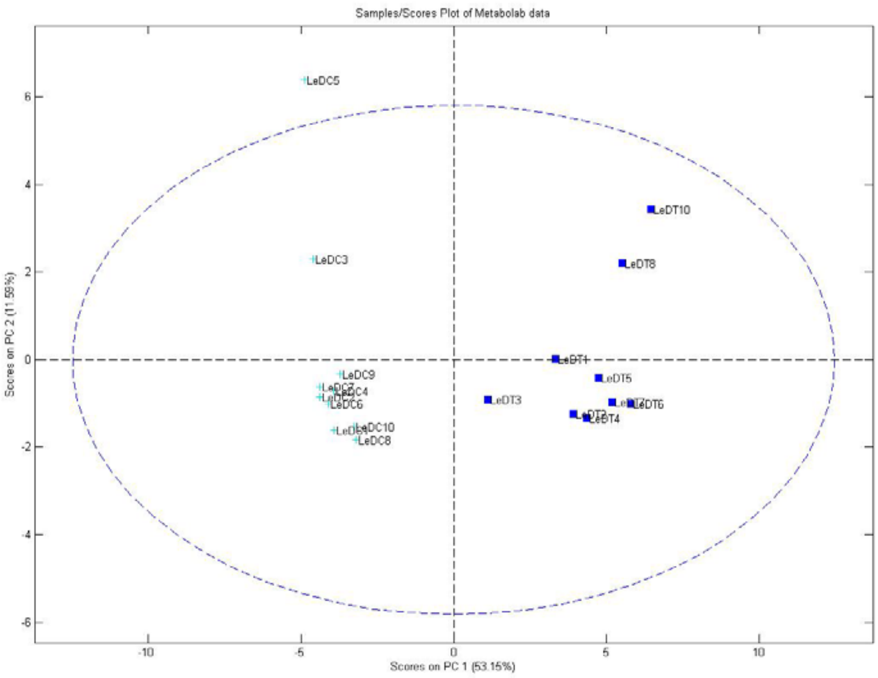

Figure 19: LeDC vs. LeDT, PC1 against PC2

The PC scores results were subjects to Students t-test:

PC1 p-value = 1.073e-11 (53.15% of variance)

PC2 p-value = 0.965 (11.59% of variance)

The t-test for the PC 1 scores data confirms there is a significant separation between the control and treated samples of *L. elliptica* digestive gland tissues. This was further investigated by identifying metabolites from the loadings plot, **Figure 20**.

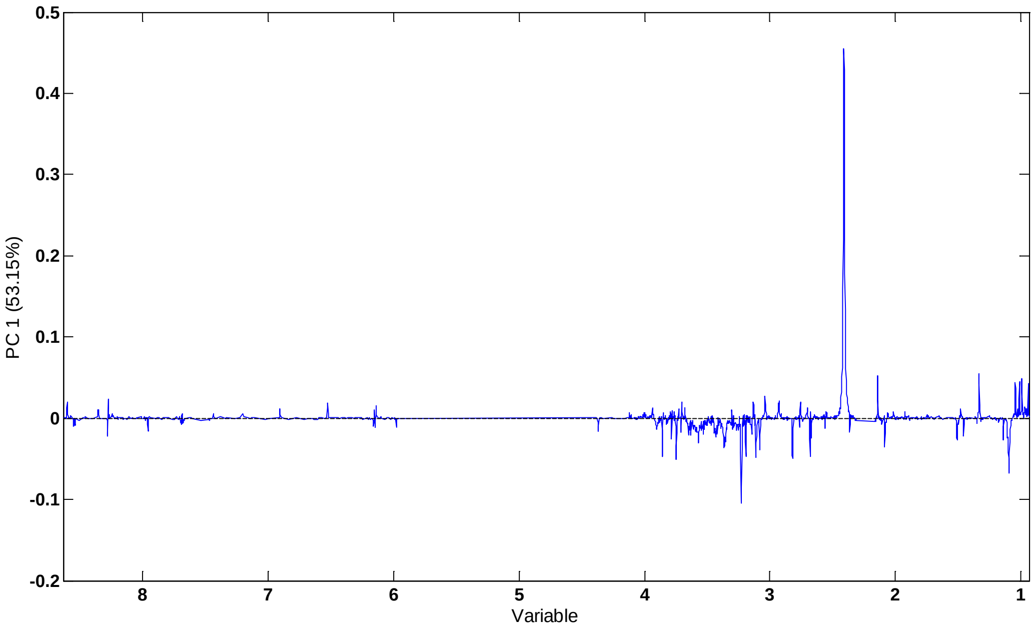

Figure 20: PC1 loadings plot

The first 25 highest loadings were used to identify the metabolites that were causing separation between the control and treated samples of *L. elliptica* digestive gland tissues seen in the PC1 loadings plot, **Table 14**.

Table 14: Metabolites identified from the first 25 highest loadings

δ_H	Metabolite
3.22	Carnitine
1.33	Lactate
2.41	Succinate
3.74	Arginine*

*Overlapping signals

Table 15: Fold change and adjusted p-value for the metabolites

Metabolite	p-value	Fold change (T/C)
Carnitine	0.18	0.75
**Lactate**	**0**	**2.13**
**Succinate**	**0**	**72.91**

A two t-tailed test, at 0.05, was performed on the metabolites identified, **Table 14**. From the adjusted p-value (Benjamini-Hochberg), lactate and succinate was found to be significantly different between the control and treated samples of *L. elliptica* digestive gland tissues for, **Table 15**.

PLS-DA was optimised to produce a model using 3 LVs and 75 bins. The permutation testing of the PLS-DA model using all the variables (100 permutations), produced an average class error of

0.15%, which shows a significant separation of the data, P value close to 0. The permutation testing of the PLS-DA model using the reduced number of variables (75, 100 permutations), produced an average class error of 0.5%, which shows a significant separation of the reduced number of variables, P value close to 0. The model using reduced number of variable was further investigated by identifying the metabolites in **Figure 21**.

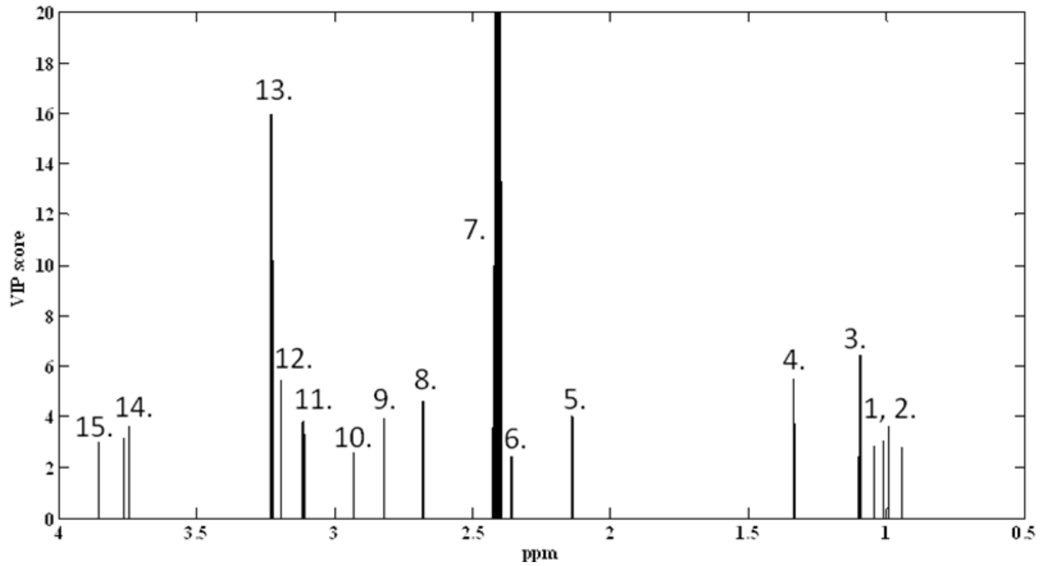

Figure 21: VIP values for 75 forward selected bins in a 3 LV model of LeDC vs. LeDT. The metabolites identified are (1) isoleucine, (2) valine, (3) 1.10ppm, unknow metabolite, (4) lactate, (5) methionine, (6) unknown metabolite, (7) succinate, (8) aspartate, (9) unknown metabolite, (10) dimethylglycine, (11) malonate, (12) β-alanine, (13) carnitine, (14) glutamate and (15) unknown metabolite

The VIP scores showed that succinate had the highest score of 307, which suggests that this contributes mainly to the separation of the control and treated samples of *L. elliptica* digestive gland tissue (for visualisation purposes the VIP scores range was alternated, **Figure 21**). Metabolites 3, 6, 9 and 15 were not identified either due to complex overlapping signals or the signal intensity being weak. To identify these further separations of the samples, using SPE methods, is required.

A two tailed t-test, at 0.05, was performed on the metabolites identified in **Figure 21**. From the adjusted p-value (Benjamini-Hochberg), lactate, succinate and valine were found to be significantly different between the control and treated samples of *L. elliptica* mantle tissues, **Table 16**.

Table 16: **Metabolites identified from the VIP scores**

Peak ID	P-value	Fold change (T/C)
Arginine	0.98	0.99
Beta-alanine	0.36	1.21
Carnitine	0.36	0.75
Dimethylglycine	0.59	0.58
Glutamate	0.66	1.11
Isoleucine	0.23	4.95
**Lactate**	**0**	**2.13**
Malonate	0.36	0.79
**Succinate**	**0**	**72.91**
**Valine**	**0**	**2.53**

LuC vs. LuT

A 3 PC PCA model was constructed for *Liothyrella uva* muscular tissue (**Figure 22, 23**). The percentages of variance of PCs 1-3 were 71.45, 8.90 and 6.68% respectively.

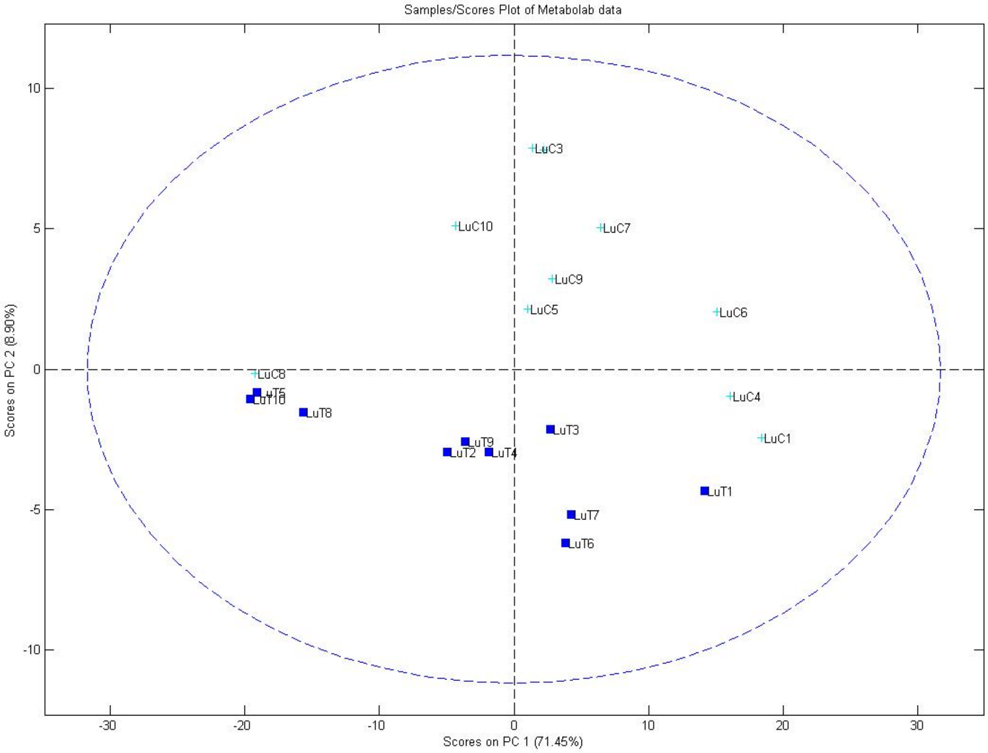

Figure 22: LuC vs. LuT, PC 1 against PC 2

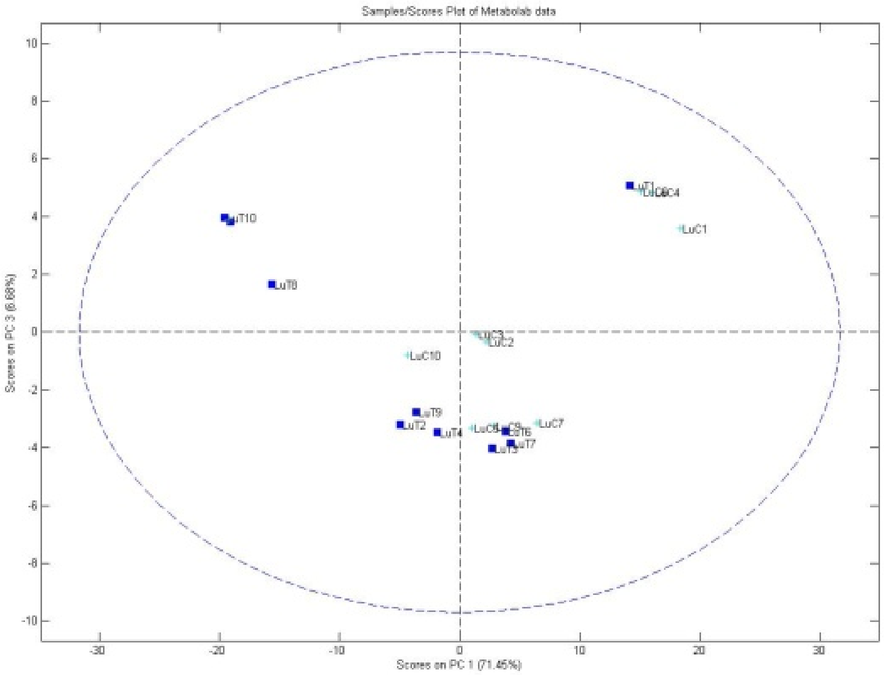

Figure 23: LuC vs. LuT, PC 1 against PC 3

The T-test p-value results for the 3 PC PCA models:
PC1 p-value = 0.127 (71.45% of variance)
PC2 p-value = 0.000157 (8.90% of variance)
PC3 p-value = 0.446 (6.68% of variance)
The t-test for the PC2 scores data confirmed there was a significant separation between the control and treated samples of *Liothyrella uva* tissues. This was further investigated by identifying metabolites form the loadings plot, **Figure 24**.

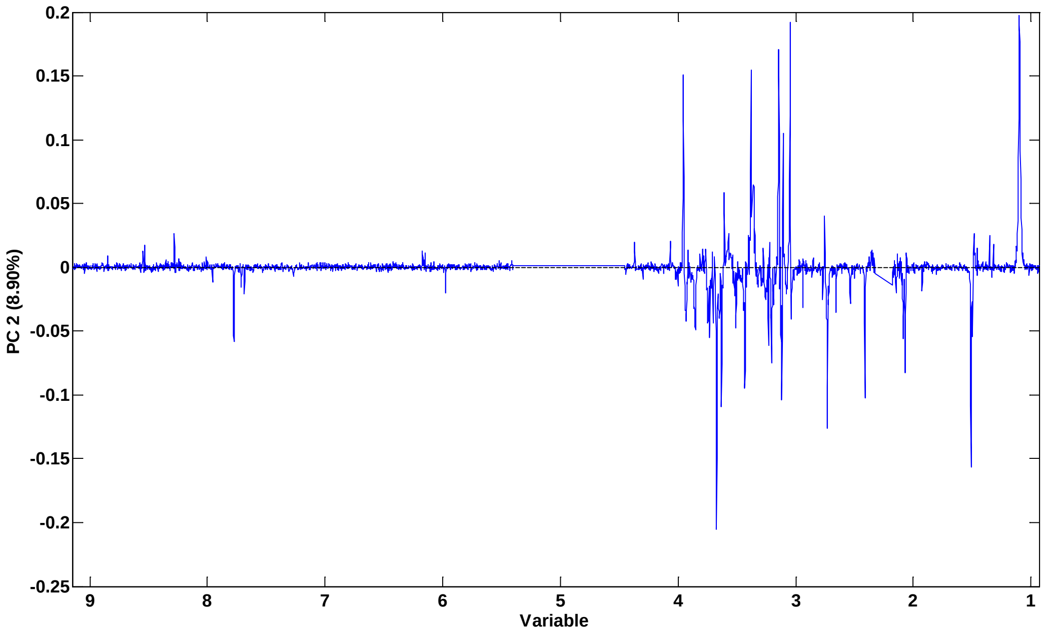

Figure 24: PC2 loadings plot

The first 25 highest loadings were used to identify the metabolites that were causing separation between the control and treated samples of *L. uva* tissues seen in PC2 loadings plot, **Table 17**.

Table 17: Metabolites identified from the first 25 highest loadings

δ_H	Metabolite
1.10	Unknow
1.50	Unknown
2.41	Succinate
2.73	Dimethylamine
3.04 & 3.95	Creatine phosphate
3.14	Dimethyl sulfone
3.63	Signal too small to ID
3.67	Ethylene glycol

Table 18: Fold change and adjusted p-value for the metabolites

Metabolite	P-value	Fold change (T/C)
Creatine phosphate	0.23	0.59
Dimethylamine	0.23	0.36
**Dimethyl sulfone**	**0.02**	**0.22**
Ethylene glycol	0.58	0.74

A two t-tailed test, at 0.05, was performed on the metabolites identified, **Table 17**. From the adjusted p-value (Benjamini-Hochberg), dimethyl sulfone was found to be significantly different between the control and treated samples of *L. uva* tissues, **Table 18**.

PLS-DA was optimised to produce a model using 4 LVs and 30 bins. The permutation testing of the PLS-DA model using all the variables (100 permutations), produced an average class error of 13.7%, which shows a significant separation of the data, P value close to 0. The permutation testing of the PLS-DA model using reduced number of variables (30, 100 permutations), produced an average class error of 16.75%, which shows a significant separation of the reduced number of variables, P value of 0.02. The model using reduced number of variables was further investigated by identifying the metabolites in **Figure 25**.

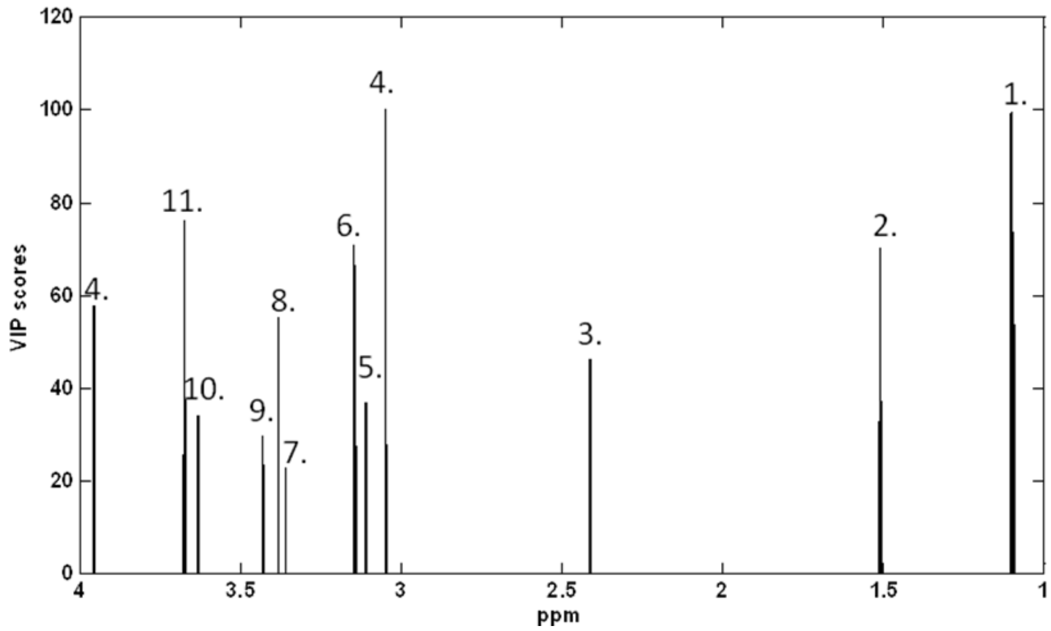

Figure 25: VIP values for 30 forward selected bins in a 4 LV model of LuC vs. LuT. The metabolites identified are (1) 1.10ppm, unknown metabolite, (2) unknown metabolite, (3) succinate, (4) creatine phosphate, (5) malonate, (6) dimethyl sulfone, (7) methanol, (8) unknown metabolite, (9) taurine, (10) unknown metabolite and (11) ethylene glycol

Figure 25 shows which metabolites contribute to the separation of the control and treated samples of *L. uva* tissues. Metabolites 1, 2, 8 and 10 were not identified either due to complex overlapping signals or the signal intensity being weak.

A two tailed t-test, at 0.05, was performed on the metabolites identified in **Figure 25**. From the adjusted p-value (Benjamini-Hochberg), dimethyl sulfone was found to be significantly different between the control and treated samples of *L. uva* mantle tissues, **Table 19**.

Table 19: Metabolites identified from the VIP scores

Peak ID	P-value	Fold change (T/C)
Creatine	0.84	1.72
**Dimethyl sulfone**	**0.02**	**0.16**
Ethylene glycol	0.9	0.9
Succinate	0.84	1.52
Taurine	0.9	0.94

PmC vs PmT
A 2 PC PCA model was constructed for *Paraceradocus miersi* whole animal tissue (**Figure 26**). The percentages of variance of PCs 1-2 were 20.22 and 17.52% respectively.

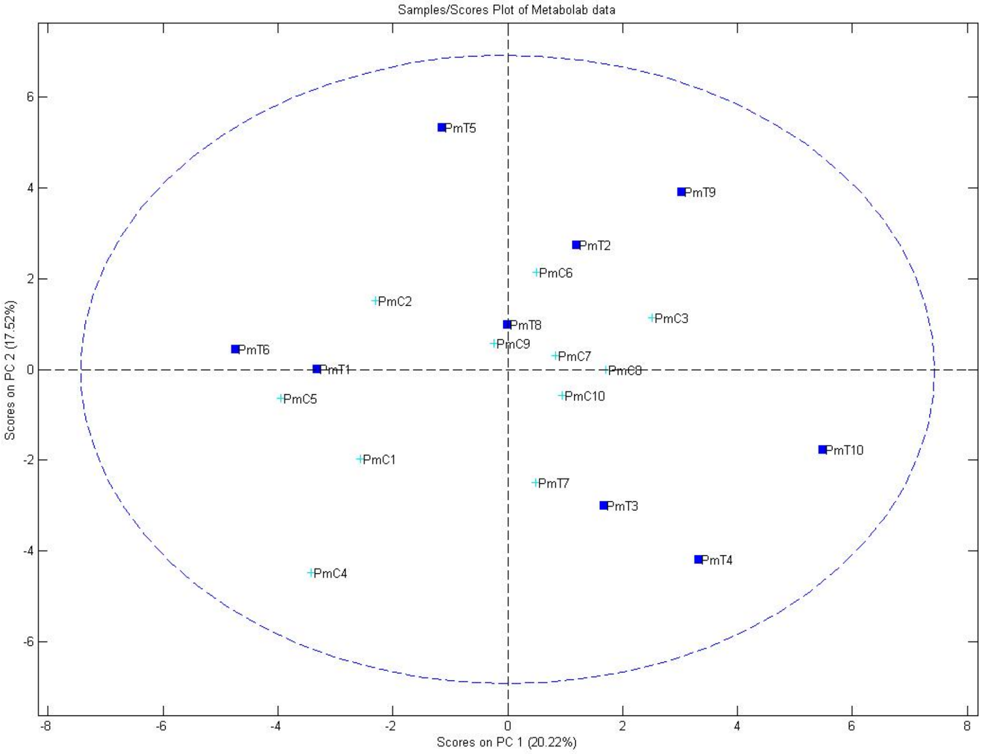

Figure 26: PmC vs. PmT, PC1 against PC2

The T-test p-value results for the 2 PC PCA models:
PC1 p-value = 0.378 (20.22% of variance)
PC2 p-value = 0.435 (17.52% of variance)
The t-test for the PC scores data showed no significant separation between the control and treated samples of the whole animal of *Paraceradocus miersi* tissues. Therefore, PLS-DA was used to force a separation in order to find some metabolic difference between the control and treated samples.

PLS-DA was optimised to produce a model using 3 LVs and 33 bins. The permutation testing of the PLS-DA model using all the variables (100 permutations), produced an average class error of

27.78%, P value of 0.04, which showed there was no significant separation between the control and treated samples. However, the PLS-DA was still continued to see if there were any metabolites which are important. The permutation testing of the PLS-DA model using reduced number of variables (33, 100 permutations), produced an average class error of 7.20%, which shows a significant separation of the reduced number of variables, P value close to 0. The model using reduced number of variables was further investigated by identifying the metabolites in **Figure 27**.

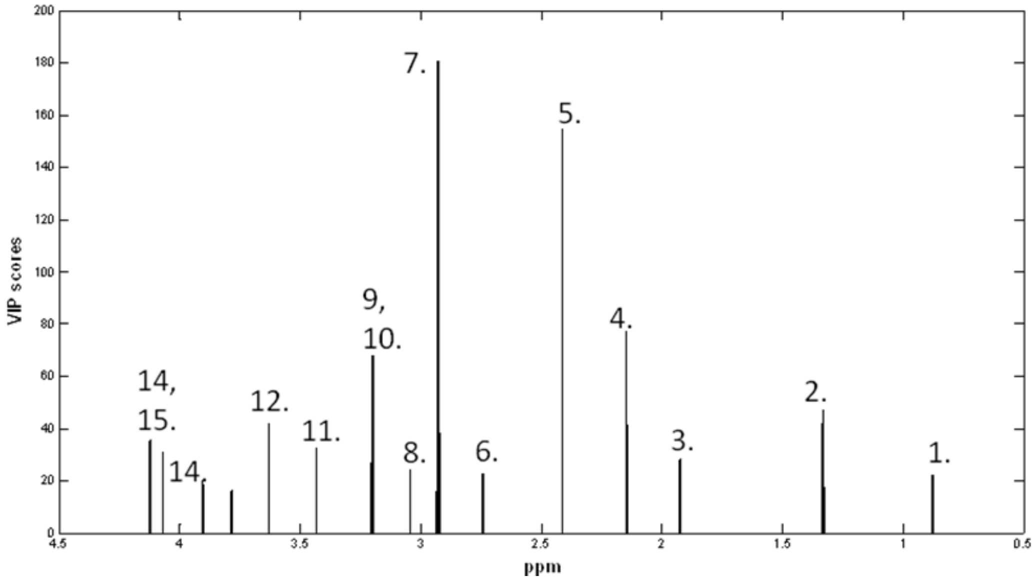

Figure 27: VIP values for 33 forward selected bins in a 3 LV model of PmC vs. PmT. The metabolites identified are (1) 2-hyrodxybutyrate, (2) lactate, (3) acetate, (4) methionine, (5) succinate, (6) sarcosine, (7) dimethylglycine, (8) creatine, (9) choline, (10) carnitine (11) taurine, (12) unknown metabolite, (13) unknown metabolite, (14) betaine and (15) ethylene glycol

Figure 27 shows which metabolites contribute to the separation of the control and treated samples of *P.miersi* tissues. Metabolites 12, 13 and 15 were not identified either due to complex overlapping signals or the signal intensity being weak.

A two tailed t-test, at 0.05, was performed on the metabolites identified in **Figure 27**. From the adjusted p-value (Benjamini-Hochberg), it was found that there were no metabolites which showed significant difference between the control and treated samples of *P.miersi* tissues, **Table 20**.

Table 20: **Metabolites identified from the VIP scores**

Peak ID	P-value	Fold change (T/C)
2-Hydroxybutyrate	0.54	1.31
Acetate	0.54	1.1
Betaine	0.93	0.99
Carnitine	0.65	0.88
Choline	0.45	1.42
Creatine	0.45	0.77
Dimethylglycine	0.45	1.66
Lactate	0.45	1.31
Methionine	0.45	0.72
Sarcosine	0.7	1.1
Succinate	0.65	1.06
Taurine	0.45	1.3

AeFC vs AeFT

A 2 PC PCA model was constructed for *Aequiyoldia eightsii* foot tissue (**Figure 28**). The percentages of variance of PCs 1-2 were 53.86 and 21.37 % respectively.

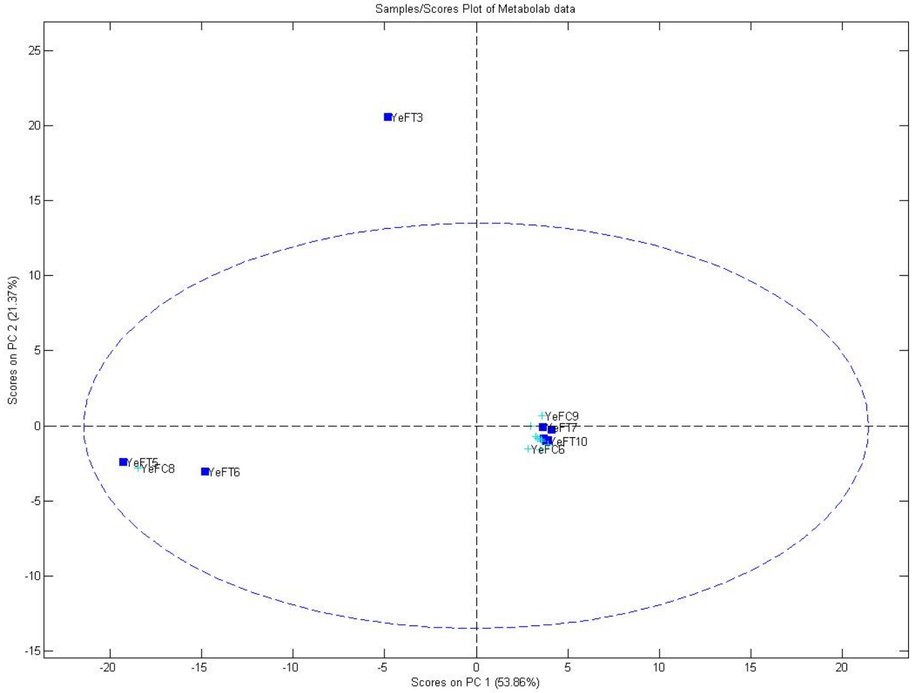

Figure 28: AeFC vs AeFT, against PC1 vs. PC2 (NB: annotated YeFC and YeFT in plot)

The T-test p-value results for the 2 PC PCA models:
PC1 p-value = 0.5027 (53.86% of variance)
PC2 p-value = 0.376 (21.37% of variance)

The t-test for the PC scores data showed no significant separation between the control and treated samples of *Aequiyoldia eightsii* foot tissues. Therefore, PLS-DA was used to force a separation in order to find some metabolic differences between the control and treated samples.

PLS-DA was optimised to produce a model using 2 LVs and 14 bins. The permutation testing of the PLS-DA model using all the variables (100 permutations), produced an average class error of 28.83%, P value of 0.034, which shows there is no significant separation between the control and treated samples. However, the PLS-DA was still continued to see if there were any metabolites which are important. The permutation testing of the PLS-DA model using reduced number of variables (14, 100 permutations), produced an average class error of 17.5%, which shows significant separation between the control and treated samples, P value close to 0. The model using reduced number of variables was further investigated by identifying the metabolites in **Figure 29.**

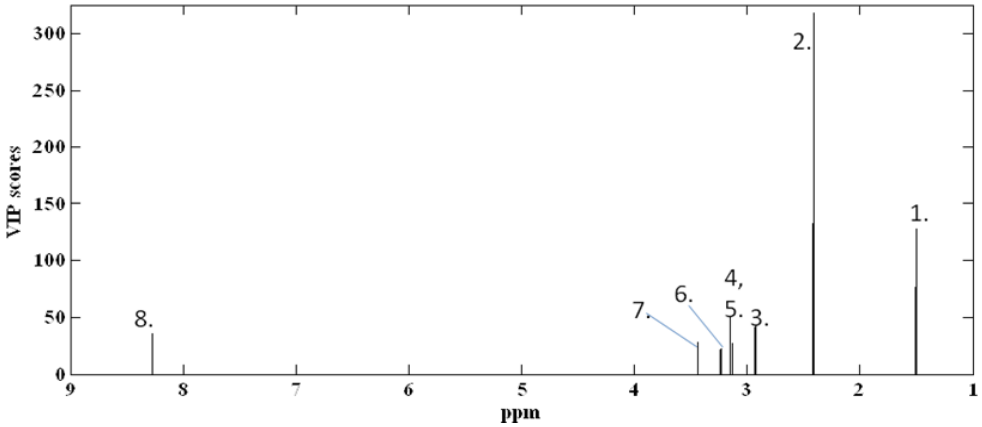

Figure 29: VIP values for 14 forward selected bins in a 2 LV model of AeFC vs. AeFT. The metabolites identified are (1) alanine, (2) succinate, (3) unknown metabolite, (4) dimethyl sulfone, (5) unknown metabolite, (6) carnitine, (7) taurine and (8) unknown metabolite.

Figure 29 shows which metabolites contribute to the separation of the control and treated samples of *A. eightsii* foot tissues. Metabolites 3, 5 and 8 were not identified either due to complex overlapping signals or the signal intensity being weak.

A two tailed t-test, at 0.05, was performed on the metabolites identified in **Figure 29**. From the adjusted p-value (Benjamini-Hochberg), alanine was found to be significantly different between the control and treated samples of foot tissue of *A. eightsii* foot tissues, **Table 21**.

Table 21: **Metabolites identified from the VIP scores**

Peak ID	P-value	Fold change (T/C)
**Alanine**	**0.01**	**0.69**
Carnitine	0.5	1.12
dimethyl sulfone	0.5	0.85
Succinate	0.12	2.39
Taurine	0.09	0.77

AeRC vs AeRT

A 6 PC PCA model was constructed for the remainder of the *Aequiyoldia eightsii* tissue (i.e. minus the foot tissue) (**Figure 30-34:** AeRC and AeRT denoted YeRC and YeRT in the plots). The percentages of variance of PCs 1-6 were 34.25, 18.85, 10.01, 7.03, 5.87 and 5.38% respectively.

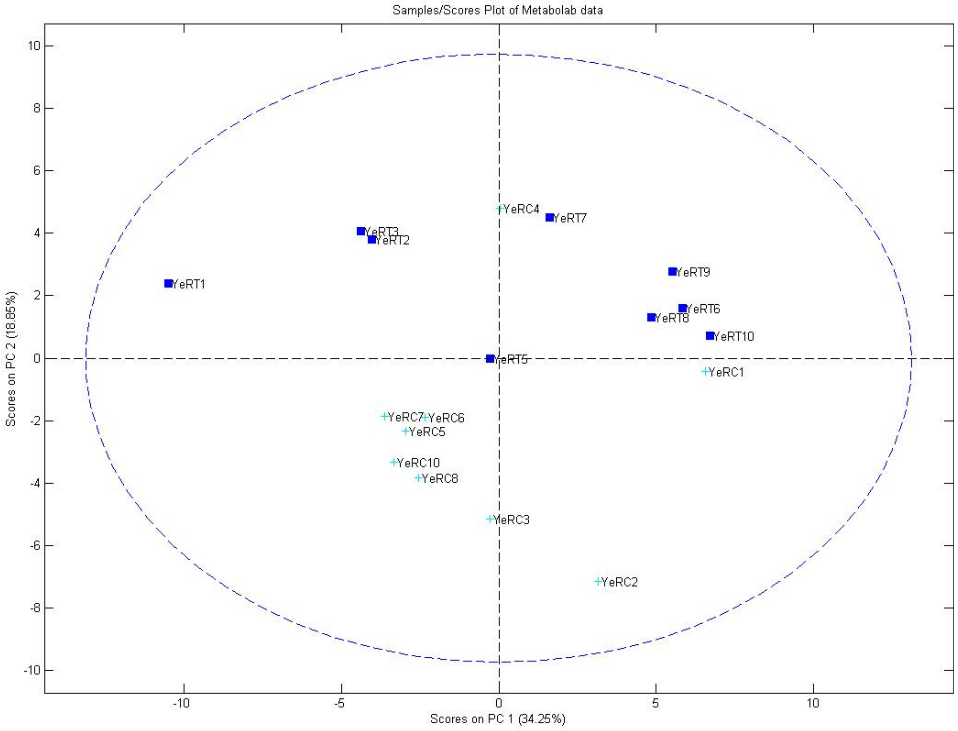

Figure 30: AeRC vs AeRT, against PC1 vs. PC2

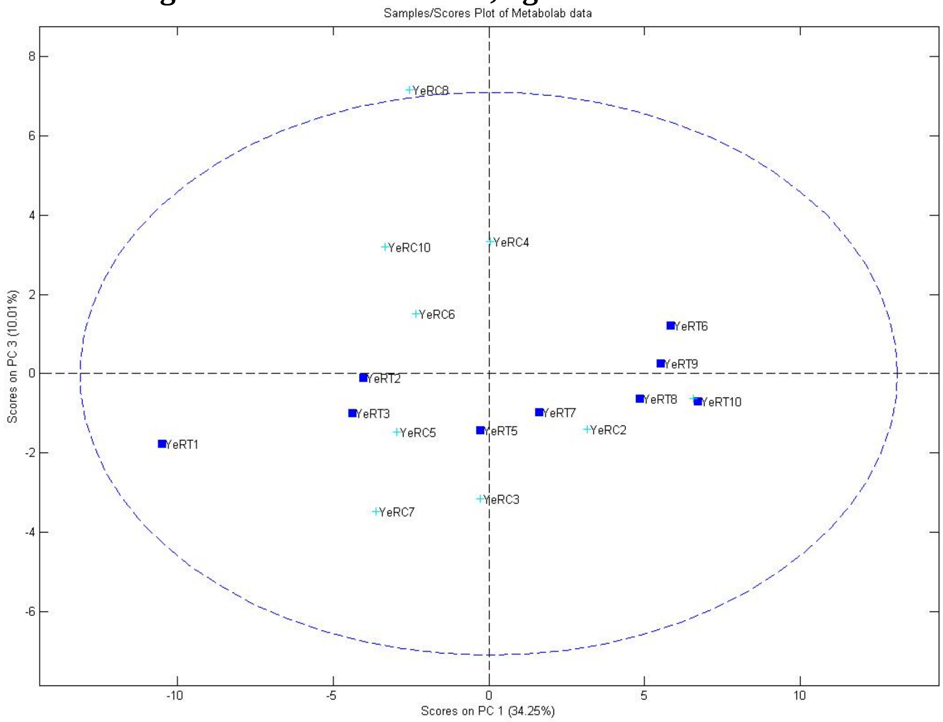

Figure 31: AeRC vs AeRT, against PC 1 vs. PC 3

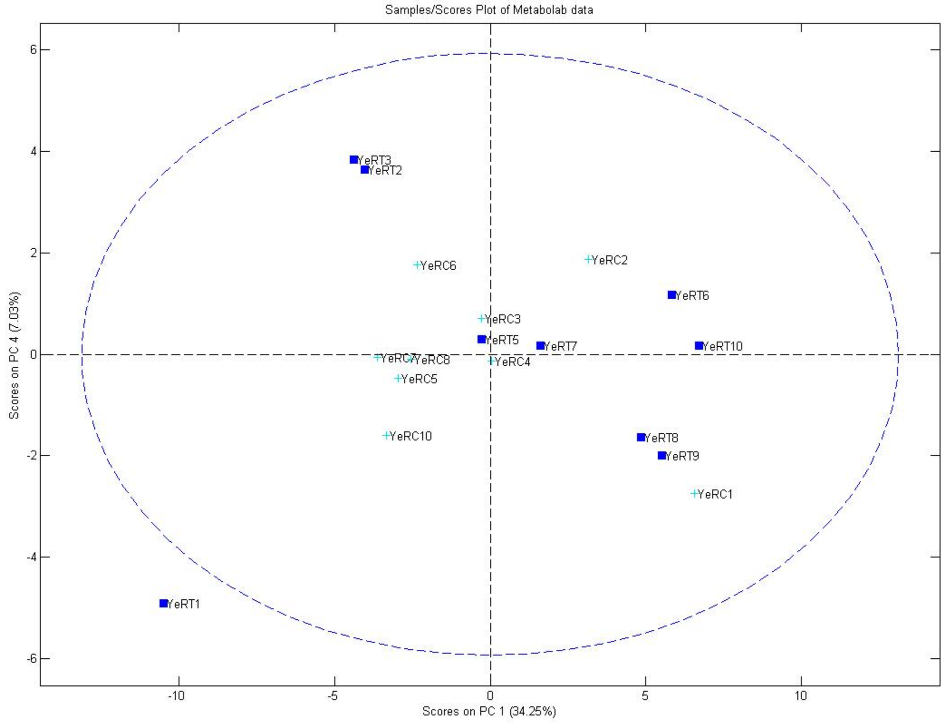

Figure 32: AeRC vs AeRT, against PC1 vs. PC4

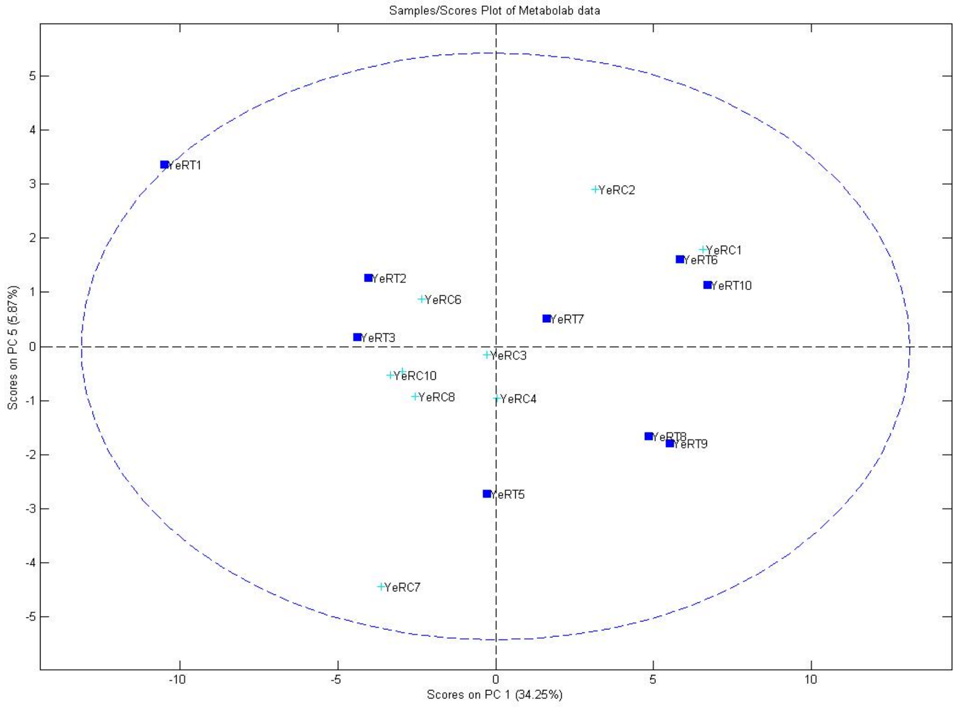

Figure 33: AeRC vs AeRT, against PC1 vs PC5

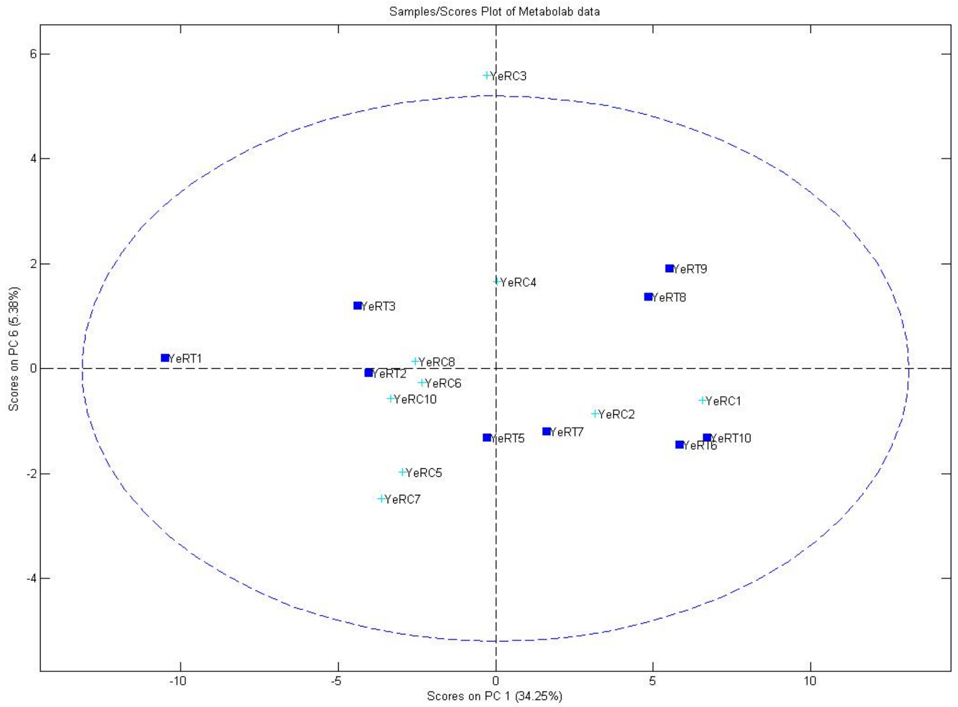

Figure 34: AeRC vs AeRT, against PC1 vs PC6

The T-test p-value results for the 6 PC PCA models:
PC1 p-value = 0.611 (34.25% of variance)
PC2 p-value = 0.00147 (18.85% of variance)
PC3 p-value = 0.364 (10.01% of variance)
PC4 p-value = 0.873 (7.03% of variance)
PC5 p-value = 0.661 (5.87% of variance)
PC6 p-value = 0.876 (5.38% of variance)
The t-test for the PC 2 scores data confirmed there was significant separation between the control and treated samples of *Aequiyoldia eightsii* tissues. This was further investigated by identifying metabolites from the loadings plot, **Figure 35**.

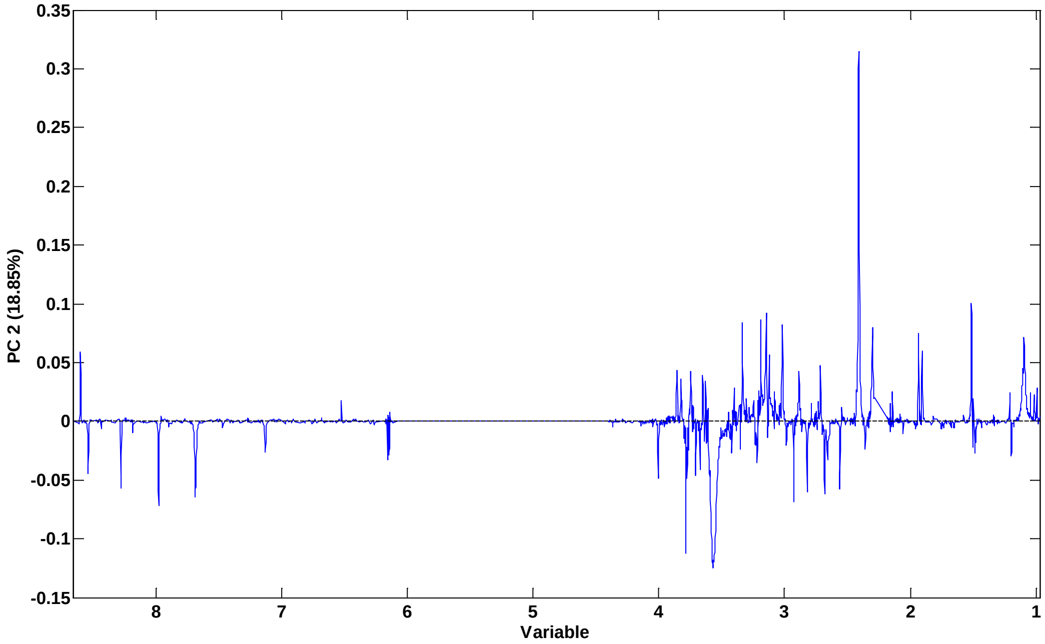

Figure 35: PC2 loadings plot

The first 25 highest loadings were used to identify the metabolites that were causing separation between the control and treated samples of *A. eightsii* tissue seen in PC2 loadings plot, **Table 22**.

Table 22: Metabolites identified from the first 25 highest loadings

δ_H	Metabolite
2.41	Succinate
3.57	Glycine

*Overlapping signals

Table 23: Fold change and adjusted p-value for the metabolites

Metabolite	P-value	Fold change (T/C)
**Succinate**	**0.01**	**7.91**
Glycine	0.85	1.05

A two tailed, t-test at 0.05, was performed on the metabolites identified, **Table 22**. From the adjusted p-value (Benjamini-Hochberg), succinate was found to be significantly different between the control and treated samples of *A. eightsii* foot tissues, **Table 23**.

PLS-DA was optimised to produce a model using 3 LVs and 75 bins. The permutation testing of the PLS-DA model using all the variables (100 permutations), produced an average class error of 0.15%, which shows a significant separation of the data, P values close to 0. The permutation testing of the PLS-DA model using reduced number of variables (75, 100 permutations), produced an average class error of 0.5%, which shows a significant separation of the reduced

number of variables, P value close to 0. The model using reduced number of variables was further investigated by identifying the metabolites in Figure 36.

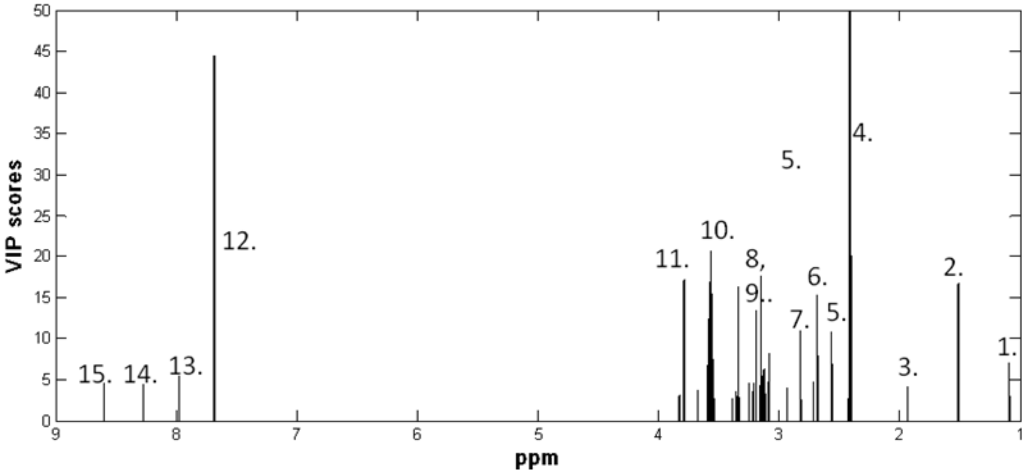

Figure 36: VIP values for 75 forward selected bins in a 3 LV model of AeRC vs. AeRT. The metabolites identified are (1) unknown metabolite, (2) unknown metbaolite, (3) unknown metabolite, (4) succinate, (5) aspartate, (6) dimethylamine, (7) dimethylglycine, (8) unknown metabolite, (9) malonate, (10) glycine, (11) anserine, (12) unknown metabolite, (13) homarine, (14) unknown metabolite and (15) unknown metabolite.

Figure 36 shows which metabolites contribute to the separation of the control and treated samples of *A. eightsii* tissues. Metabolites 1, 2, 3, 8, 12, 14 and 15 were not identified either due to complex overlapping signals or the signal intensity being weak.

A two tailed t-test, at 0.05, was performed on the metabolites identified in **Figure 36**. From the adjusted p-value (Benjamini-Hochberg), aspartate, dimethylamine and succinate were found to be significantly different between the control and treated samples of *A. eightsii* tissues, **Table 24**.

Table 24: **Metabolites identified from the VIP scores**

Peak ID	P-value	Fold change (T/C)
Anserine	0.94	1.02
**Aspartate**	**0.04**	**0.53**
**Dimethylamine**	**0.01**	**0.51**
Dimethylglycine	0.27	0.67
Glycine	0.94	1.05
Malonate	0.94	0.95
**Succinate**	**0.02**	**7.91**

MmC vs MmT

A 3 PC PCA model was constructed for *Marseniopsis mollis* whole animal tissue (**Figure 37, 38**). The percentages of variance of PCs 1-3 were 34.73, 16.56 and 12.73 % respectively.

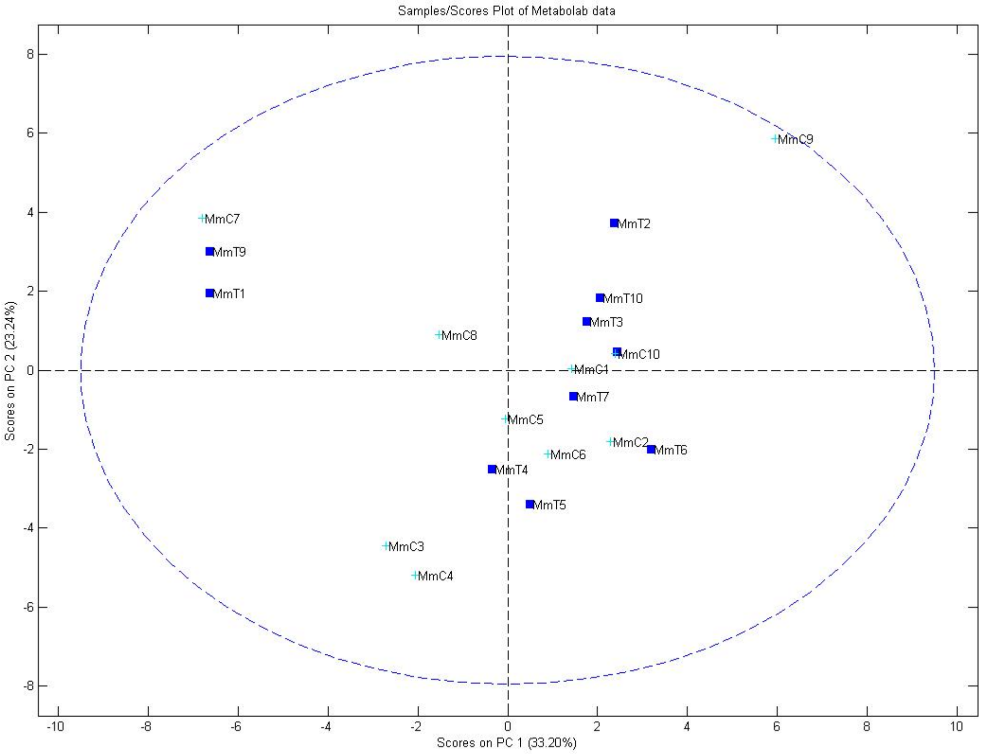

Figure 37: MmC vs MmT, PC1 against PC2

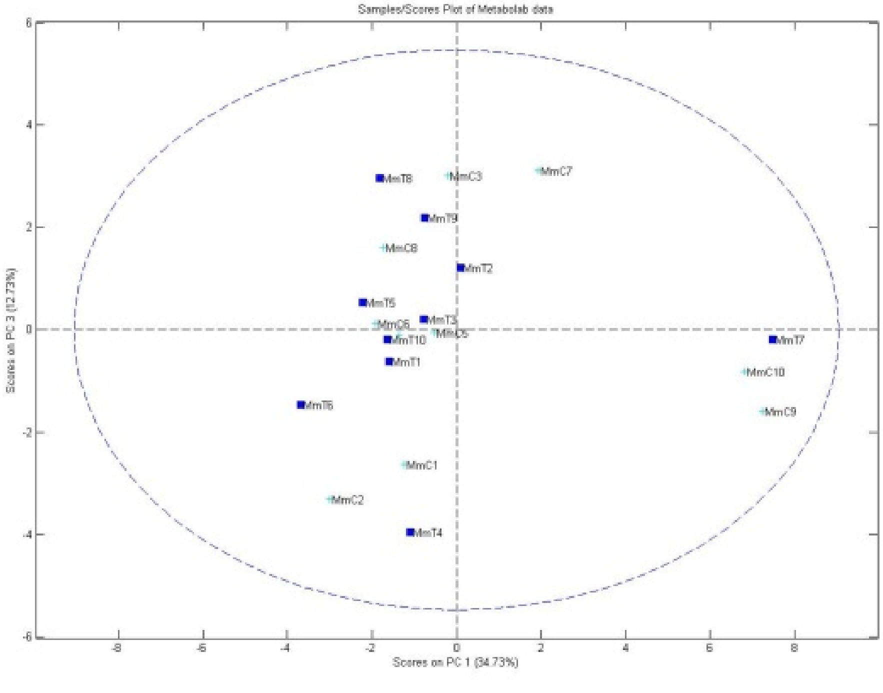

Figure 38: MmC vs MmT, PC1 against PC3

The T-test p-value results for the 3 PC PCA models:
PC1 p-value = 0.431 (34.73% of variance)
PC2 p-value = 0.0317 (16.56% of variance)
PC3 p-value = 0.884 (12.73% of variance)
The t-test for the PC scores data showed no significant separation between the control and treated samples of *Marseniopsis mollis* tissues. Therefore, PLS-DA was used to force a separation in order to find some metabolic difference between the control and treated samples.

PLS-DA was optimised to produce a model using 1 LV and 33 bins. The permutation testing of the PLS-DA model using all the variables (100 permutations), produced an average class error of 38.18% with a P value of 0.146, which shows there is no significant separation between the control and treated samples. However, the PLS-DA was still continued to see if there were any metabolites which are important. The permutation testing of the PLS-DA model using the reduced number of variables (33, 100 permutations), produced an average class error of 29.30%, which shows there is no significant separation between the control and treated samples, P value of 0.04. However, the metabolites in **Figure 39** were identified.

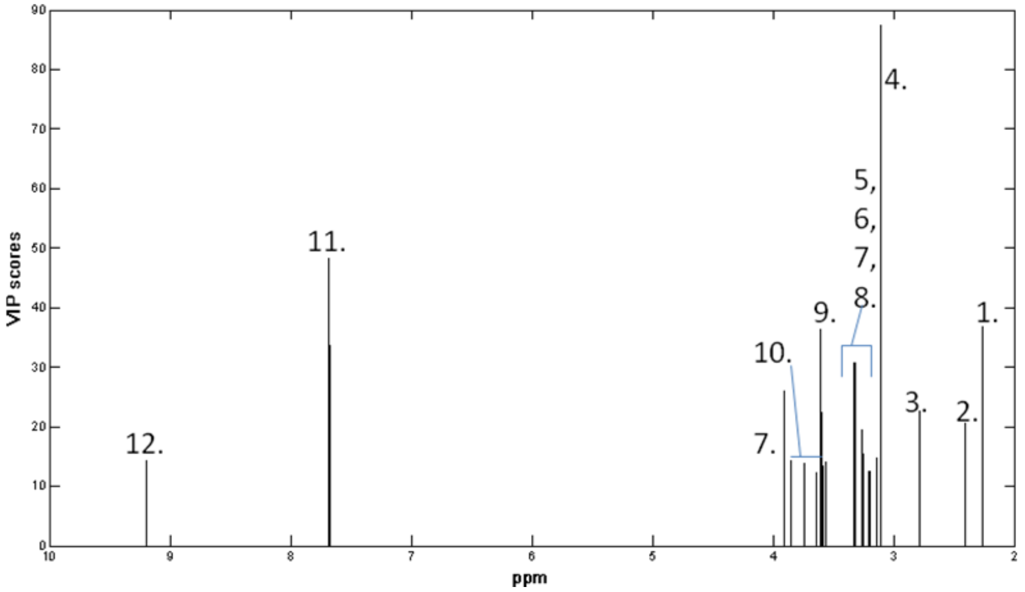

Figure 39: VIP values for 33 forward selected bins in a 1 LV model of MmC vs. MmT. The metabolites identified are (1) acetoacetate, (2) succinate, (3) unknown metabolite, (4) malonate, (5) dimethylsulfone, (6) carnitine, (7) betaine, (8) unknown metabolite, (9) glycine, (10) unknown metabolite, (11) unknown metabolite and (12) unknown metabolite.

Figure 39 shows which metabolites contribute to the separation of the control and treated samples of *M. mollis* tissues. Metabolites 1, 2, 3, 8, 12, 14 and 15 were not identified either due to complex overlapping signals or the signal intensity being weak.

A two tailed t-test, at 0.05, was performed on the metabolites identified in **Figure 39**. From the adjusted p-value (Benjamini-Hochberg), it was found that these metabolites did not show any significant difference between the control and treated samples of *M. mollis* tissues, **Table 25**.

Table 25: **Metabolites identified from the VIP scores**

Peak ID	P-value	Fold change (T/C)
Acetoacetate	0.63	0.79
Betaine	0.78	1.12
Carnitine	0.63	1.21
Dimethyl sulfone	0.96	1.05
Glycine	0.96	1.01
Malonate	0.96	1.01
Succinate	0.63	1.42

CgC vs CgT

A 2 PC PCA model was constructed for *Cucumaria georgiana* whole animal tissue (**Figure 40**). The percentages of variance of PCs 1-10 were 43.02 and 22.49% respectively.

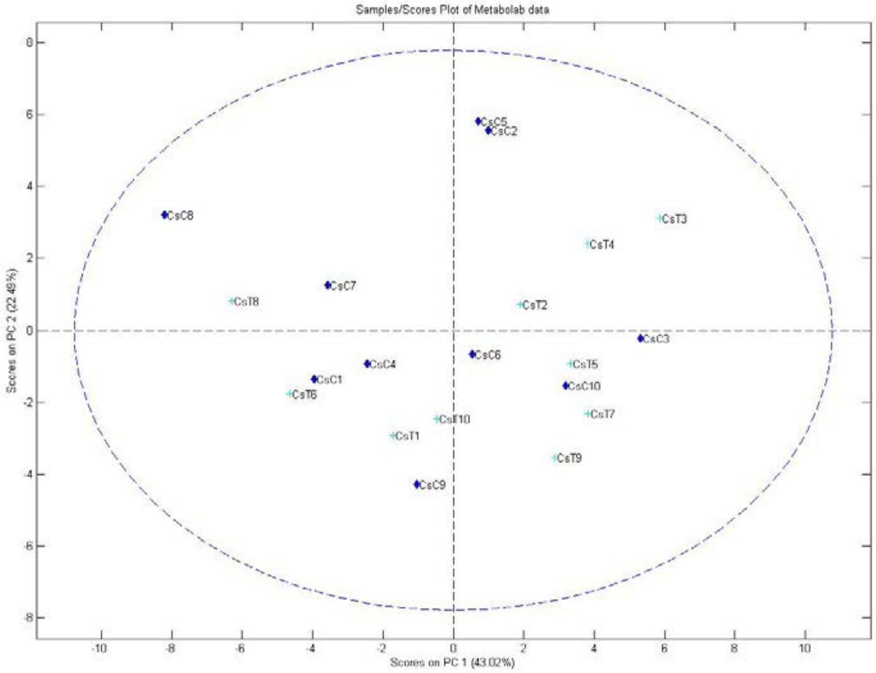

Figure 40: CgC vs CgT, PC1 against PC2, NB. Denoted CsC and CsT in plot)

The T-test p-value results for the 2 PC PCA models:
PC1 p-value = 0.347 (43.02% of variance)
PC2 p-value = 0.292 (22.49% of variance)

The t-test for the PC scores data showed no significant separation between the control and treated samples. Therefore, PLS-DA was used to force a separation in order to find some metabolic difference between the control and treated samples of *Cucumaria georgiana* tissues.

PLS-DA was optimised to produce a model using 1 LV and 54 bins. The permutation testing of the PLS-DA model using all the variables (100 permutations), produced an average class error of 48.10% , P value of 0.45, which shows there is no significant separation between the control and treated samples. However, the PLS-DA was still continued to see if there were any metabolites which are important. The permutation testing of the PLS-DA model using the reduced number of variables (54, 50 permutations), produced an average class error of 32.90%, P value of 0.02, which shows there is significant separation between the control and treated samples for 54 variables. The model using reduced number of variables was further investigated by identifying the metabolites in **Figure 41**.

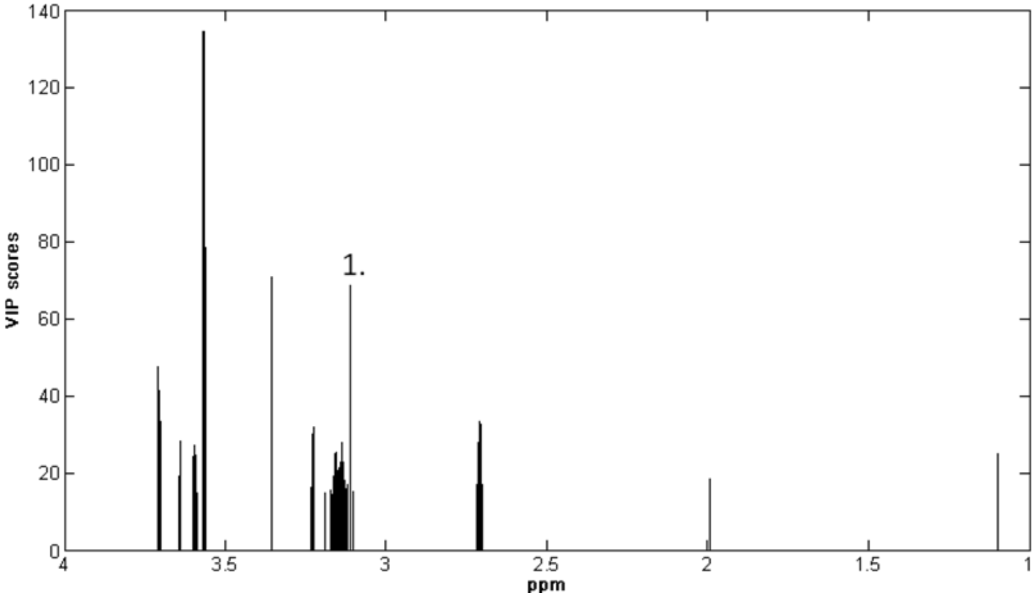

Figure 41: VIP values for 33 forward selected bins in a 1 LV model of CgC vs. CgT. The metabolite identified was (1) malonate

The only metabolite that was identifiable was malonate. There other signals in **Figure 41** were found to be either due to complex overlapping signals or the signal intensity being weak. The high P value (0.45) suggested that there was no significant separation of the control and treated samples. However, further investigations of the reduced variables did suggest this showed significant separation. When considering the high P-value of the model it seems the reduced variables selected, to cause this significant separation, is of small and/or overlapped signals. Therefore, to identify these signals further work will need to be considered.

Table 26: **Metabolites identified from the VIP scores**

Peak ID	P-value	Fold change (T/C)
Malonate	0.41	1.13

A two tailed t-test, at 0.05, was performed on the metabolites identified in **Figure 41**. From the adjusted p-value (Benjamini-Hochberg), it was found that these metabolites did not show any significant difference between the control and treated samples of *Cucumaria georgiana* tissues, **Table 26**.

Summary

The PCA analysis showed that there were PC scores that gave significant separations between the control and treated samples for LeS, LeM, LeF, LeG, LeD, Lu and AeR tissues. However, Pm, AeF, Mm and Cg did not show any PC scores which showed significant separations.

PLS-DA was carried out for the various animal tissues. From the results, various metabolites were identified and tested for their significance. There are many metabolites which were not identified due to the intensity of the signal being weak or overlapped complex signals. To investigate these metabolites advanced chromatographic separations and NMR techniques are required.
