# Supplementary material for: Biodiversity in marine invertebrate responses to acute warming revealed by a comparative multi‐omics approach
Source: Glob Chang Biol. 2016 Jun 17;23(1):318–30. doi: 10.1111/gcb.13357 (PMC6849730; doi:10.1111/gcb.13357)
Supplement: Supplementary file 9 — Data S2. LC‐MS statistics and signal annotations. [file GCB-23-318-s009.pdf]

**Table ...: LC-MS statistics and signal annotations**

**Columns:** *M/Z* and Intensity: *m/z* values and average intensities from polar extracts peaklist
RT: retention times [min]
PC4: 4th principal component loadings (PCA), p=0.0005, 5 % of variance; the 25 highest absolute values ae marked in green.
LV1: 1st latent variable weightings (PLS-DA)
VarImp: PLS-DA Variable Importance vector calculated from all 4 LVs, forward selected signals (26) in green
Adj.P: adjusted p-value (q value) from t-test after 5 % FDR correction, remaining significant values in green
Change: fold-change of treated compared to control
Formula: assigned empirical molecular formula (na - no assignment possible with standard settings)
Ion form: adduct
Calc. *m/z* : calculated putative *m/z* value
Mass error: calculated difference of calcuated and experimental *m/z* value in ppm;
MIPack match in KEGG Compound database
Comment

| *M/Z* | Intensity | RT | PC4 | LV1 | VarImp | ADJ_P | Change | Formula | Ion form | Calc. *m/z* | Mass error | Annotation | Comment |
|---|---|---|---|---|---|---|---|---|---|---|---|---|---|
| 132.10167 | 162873 | 1.86 | 0.0877 | 0.0987 | 10.1123 | 0.001375 | 2.377678 | C6H13NO2 | [M+H]+ | 132.10191 | -1.78 | Leucine / Isoleucine | KEGG |
| 141.01561 | 255221 | 1.48 | 0.0857 | 0.1033 | 10.5191 | 0.04847 | 2.366205 | C4H6O4 | [M+Na]+ | 141.01583 | -1.55 | Succinate | KEGG |
| 150.05860 | 215699 | 1.01 | 0.0756 | 0.0664 | 4.6269 | 0.154816 | 1.72893 | C5H11NO2S | [M+H]+ | 150.05833 | 1.82 | Methionine | KEGG |
| 157.01082 | 111541 | 0.68 | 0.0803 | 0.0859 | 7.7559 | 0.002013 | 2.153009 | C4H6O5 | [M+Na]+ | 157.01075 | 0.47 | no ID | KEGG: malate |
| 166.08491 | 708440 | 3.34 | 0.0740 | 0.0721 | 5.4683 | 0.011213 | 1.879873 | C3H12N5OP | [M+H]+ | 166.08522 | -1.89 | no ID | |
| 188.07071 | 146995 | 3.59 | 0.0719 | 0.0893 | 8.1436 | 0.07195 | 2.055938 | C11H9NO2 | [M+H]+ | 188.07061 | 0.56 | no ID | |
| 193.00191 | 483880 | 1.50 | 0.0862 | 0.1562 | 25.6129 | 0.001147 | 3.212724 | C4H9O6 | [M+Ca-H]+ | 193.00196 | -0.24 | Succinate | Succinate+2H2O+Ca |
| 205.09693 | 619275 | 3.61 | 0.0710 | 0.0840 | 7.1846 | 0.042603 | 1.964373 | C11H12N2O2 | [M+H]+ | 205.09715 | -1.09 | Tryptophan | KEGG |
| 208.99659 | 80820 | 0.70 | 0.0435 | 0.0501 | 2.6695 | 0.01481 | 1.59169 | C4H9O7Ca? | | | | no ID | |
| 211.01328 | 58514 | 1.49 | 0.0450 | 0.0792 | 6.1626 | 0.002922 | 2.083923 | | | | | no ID | |
| 218.13862 | 3257084 | 2.75 | 0.0924 | 0.1042 | 10.8626 | 0.016891 | 2.081101 | C10H19NO4 | [M+H]+ | 218.13869 | -0.30 | O-Propanoylcarnitine | KEGG |
| 219.13388 | 549995 | 0.53 | -0.0886 | -0.0496 | 2.9989 | 0.558585 | 0.580995 | C9H18N2O4 | [M+H]+ | 219.13393 | -0.25 | D-Lysopine | KEGG |
| 219.14196 | 302608 | 2.73 | 0.0618 | 0.0827 | 6.8690 | 0.023085 | 1.878246 | C9(13C)H19NO4 | [M+H]+ | 219.14204 | -0.37 | O-Propanoylcarnitine | 13C isotope |
| 220.99687 | 854792 | 1.49 | 0.2249 | 0.3105 | 100.1379 | 0 | 12.76707 | C5H9O7 | [M+Ca-H]+ | 220.99687 | 0.00 | Succinate | Succinate+H2O+HCOOH+Ca |
| 232.15435 | 977486 | 3.43 | 0.0776 | 0.0980 | 9.5533 | 0.318281 | 1.974108 | C11H21NO4 | [M+H]+ | 232.15434 | 0.06 | O-Butanoylcarnitine | KEGG |
| 232.92812 | 196810 | 1.13 | -0.0535 | -0.0822 | 6.7995 | 0.005271 | 0.476004 | | | | | no ID | |
| 234.06485 | 33300 | 1.86 | 0.0345 | 0.0545 | 3.1842 | 0.02275 | 1.902291 | C7H15N3OS | [M+2Na-H]+ | 234.06475 | 0.43 | no ID | |
| 236.99916 | 255115 | 0.71 | 0.0609 | 0.0776 | 6.1677 | 0.002922 | 2.027368 | | | | | no ID | |
| 250.93869 | 926090 | 1.12 | -0.0787 | -0.0907 | 8.5581 | 0.002922 | 0.443268 | | | | | no ID | |
| 255.08393 | 746442 | 0.60 | -0.1222 | -0.1298 | 19.8320 | 0.624629 | 0.452282 | | | | 0.34 | no ID | |
| 259.05967 | 168691 | 0.53 | -0.0728 | -0.0881 | 7.7640 | 0.000357 | 0.453089 | C6H14N2O7S | [M+H]+ | 259.05945 | 0.84 | no ID | most likely of 5 possible formulae |
| 277.04050 | 487159 | 1.48 | 0.0810 | 0.1156 | 13.2647 | 0.011213 | 2.522515 | C8H13O9 | [M+Mg-H]+ | 277.04045 | 0.18 | Succinate | Succinate dimer + H2O+Mg |
| 285.00432 | 89169 | 4.13 | 0.0742 | 0.0893 | 8.2623 | 0.111732 | 2.425002 | | | | | no ID | |
| 291.05591 | 77872 | 1.48 | 0.0789 | 0.1104 | 12.0093 | 0.011213 | 2.874975 | C9H15O9 | [M+Mg-H]+ | 291.05610 | -0.65 | Succinate | Succinate dimer + MeOH+Mg |
| 291.12951 | 78762 | 0.56 | 0.0644 | 0.1186 | 14.2234 | 0.000003 | 2.971644 | | | | -0.70 | no ID | inorganic |
| 293.01799 | 404742 | 1.48 | 0.0707 | 0.1023 | 10.2317 | 0.040036 | 2.219963 | C8H13O9 | [M+Ca-H]+ | 293.01800 | -0.03 | Succinate | Succinate dimer + H2O+Ca |
| 298.28539 | 77043 | 4.20 | 0.0455 | 0.0888 | 8.4893 | 0.494761 | 2.909165 | C17H35N3O | [M+H]+ | 298.28529 | 0.34 | no ID | |
| 306.99636 | 63771 | 0.69 | 0.0612 | 0.0874 | 7.8503 | 0.011213 | 2.364833 | | | | | no ID | |
| 322.11555 | 65945 | 0.52 | 0.0282 | 0.0367 | 1.3482 | 0.023085 | 1.400742 | | | | | no ID | |
| 348.14823 | 74895 | 1.08 | -0.0856 | -0.0832 | 6.7830 | 0.738011 | 0.393728 | | | | | no ID | 13C isotope |
| 367.04374 | 95951 | 0.84 | 0.0801 | 0.0429 | 2.0408 | 0.7146 | 1.614695 | | | | | no ID | |
| 373.35379 | 561658 | 3.50 | 0.1252 | 0.1374 | 22.1960 | 0.21384 | 2.769798 | C20H44N4O2 | [M+H]+ | 373.35370 | 0.23 | no ID | |
| 374.35716 | 119964 | 3.49 | 0.0741 | 0.0939 | 9.7406 | 0.317718 | 2.329491 | C19(13C)H44N4O2 | [M+H]+ | 374.35705 | 0.29 | no ID | 13C isotope |
| 377.05659 | 627826 | 1.48 | 0.0933 | 0.1022 | 10.7482 | 0.133245 | 2.892241 | C12H17O12 | [M+Mg-H]+ | 377.05649 | 0.25 | Succinate | Succinate trimer +Mg |
| 393.03408 | 2841349 | 1.48 | 0.0623 | 0.0938 | 8.3994 | 0.451667 | 1.859447 | C12H17O12? | [M+Ca-H]+ | 393.03404 | 0.09 | Succinate | Succinate trimer +Ca |
| 394.03730 | 376423 | 1.48 | 0.0720 | 0.0705 | 4.7736 | 0.386538 | 1.976187 | C11(13C)H17O12 | [M+Ca-H]+ | 394.03739 | -0.24 | Succinate | 13C isotope |
| 406.05623 | 38142 | 0.52 | -0.0439 | -0.0542 | 2.8558 | 0.022416 | 0.531614 | | | | | no ID | |
| 414.89191 | 238990 | 1.11 | -0.0619 | -0.0642 | 4.1268 | 0.023085 | 0.55247 | | | | | no ID | |
| 464.87449 | 366826 | 1.10 | -0.0578 | -0.0860 | 7.5412 | 0.04847 | 0.44803 | | | | | no ID | |
| 565.24376 | 477281 | 3.96 | 0.1241 | 0.1519 | 25.1998 | 0.887384 | 1.712459 | | | | | no ID | |
| 619.39023 | 294341 | 4.61 | -0.0778 | -0.0452 | 2.1501 | 0.979405 | 0.900532 | | | | | no ID | |
| 745.82075 | 135997 | 4.48 | 0.0742 | -0.0046 | 1.2614 | 0.942697 | 0.833276 | | | | | no ID | |
| 803.03410 | 565432 | 3.89 | -0.0057 | -0.0946 | 9.0070 | 0.507877 | 0.432126 | | | | | no ID | |
| 803.36789 | 433231 | 3.89 | 0.0035 | -0.0855 | 7.5743 | 0.525726 | 0.442321 | | | | | no ID | |
